# DEPDC1B promotes development of cholangiocarcinoma through enhancing the stability of CDK1 and regulating malignant phenotypes

**DOI:** 10.3389/fonc.2022.842205

**Published:** 2022-12-06

**Authors:** Zhenhai Zhang, Xinxing Wang, Peihua Nie, Yejun Qin, Junping Shi, Shifeng Xu

**Affiliations:** ^1^ Department of Hepatobiliary Surgery, Shandong Provincial Hospital Affiliated to Shandong First Medical University, Jinan, Shandong, China; ^2^ Department of Ophthalmology and Otorhinolaryngology, Shandong Provincial Third hospital, Jinan, Shandong, China; ^3^ Department of Pathology, Shandong Provincial Hospital Affiliated to Shandong First Medical University, Jinan, Shandong, China; ^4^ Medical Department, OrigiMed, Shanghai, China

**Keywords:** cholangiocarcinoma, DEPDC1B, CDK1, tumor promotor, ubiquitination

## Abstract

Cholangiocarcinoma (CCA) is the second most common primary tumor of the hepatobiliary system. At present, the therapeutic efficiency of cholangiocarcinoma is fairly low and the prognosis is poor. The root cause is that the molecular mechanism of the occurrence and development of CCA is largely unclear. This work intended to clarify the role of DEP domain-containing protein 1B (DEPDC1B) in the progress of CCA through cellular biology research strategies and further clarify the molecular mechanism of CCA. Clinical tissue-related detection showed that the expression level of DEPDC1B in tumor tissues was significantly higher than that in normal tissues and was positively correlated with tumor grade. Knockdown of the endogenous DEPDC1B of CCA cells can significantly inhibit cell proliferation and migration, while promoting cell apoptosis and blocking the cell cycle. DEPDC1B overexpression induced the opposite effects. Studies in animal models also showed that the downregulation of DEPDC1B can reduce the tumorigenicity of CCA cells. In addition, through gene profiling analysis and molecular biology studies, we found that CDK1 may be an important downstream mediator of DEPDC1B, the protein stability of which was significantly decreased through the ubiquitin–proteasome system in DEPDC1B knockdown cells. Moreover, knockdown of CDK1 can weaken the promotion of CCA caused by DEPDC1B overexpression. In summary, our research showed that DEPDC1B plays an important role in the development of CCA and its targeted inhibition may become one of the important methods to inhibit the progress of CCA.

## Introduction

Cholangiocarcinoma (CCA) is the most common invasive malignancy, originating from the biliary system and involving the intrahepatic, perihepatic, and distal bile ducts (1–3). Due to the heterogeneity and origin of CCA, it can be simply divided into intrahepatic CCA (iCCA) and extrahepatic CCA (eCCA) according to the location (4). Globally, the incidence of iCCA showed an annual growth trend of 4%, whereas the incidence of eCCA remained largely unchanged (5). Although surgery is the preferred treatment for all disease subtypes, only a small number of patients (approximately 35%) can undergo effective surgical excision (2, 6). For patients with advanced or unresectable CCA, the standard treatment options available are gemcitabine and cisplatin (7). However, the treatment effectiveness of CCA is limited, and the median overall survival is usually less than 1 year (8). Fortunately, the genetic pattern of each subtype of CCA was gradually determined with the development of comprehensive exome and transcriptome sequencing (9). Therefore, a deeper understanding of the molecular mechanism of cholangiocarcinoma is an indispensable part of the establishment of accurate medical treatment. In particular, promising molecular targets for precision medicine deserve further exploration.

DEPDC1B (DEP domain-containing protein 1B) is a 61-kDa protein encoded by 529 amino acids on human chromosome 5q12, containing an N-terminal DEP domain and a C-terminal Rho-GAP (GTPase-activated protein)-like domain (10). The DEP domain is a spherical domain of about 90 amino acids, which mediates the localization of the cell membrane and the determination of cell polarity (11, 12). The Rho-GAP domain is involved in transduction of the Rho GTPase signal and regulation of cell growth, cell movement, cell differentiation, cytoskeleton reorganization, and cell cycle (13). DEPDC1B has an unstable expression in the procession of cell cycle and accumulates to the peak in the G2 phase, whose function is similar to that of cell-cycle regulator cyclin B (10). Moreover, accumulating evidence demonstrated that DEPDC1B is overexpressed in diverse types of cancers such as non-small cell lung cancer, oral cancer, prostate cancer, soft tissue sarcoma, cervical cancer, and malignant melanoma (14–19). For example, knockdown of DEPDC1B inhibits the cellular behavior of malignant melanoma by slowing cell proliferation and inducing apoptosis (19). Analogously, downregulation of DEPDC1B can hinder the progression of glioblastoma (20). In addition, DEPDC1B promotes the migration and invasion of pancreatic cancer through the Rac1/PAK1-LIMK1-Cofilin1 signal pathway (21). Furthermore, DEPDC1B is recognized as a key regulator of mouse and human myoblast proliferation (22). On the other hand, DEPDC1B is identified as a new diagnostic and prognostic biomarker of hepatocellular carcinoma and prostate cancer, which has significant clinical value (23, 24). However, the biological function and potential mechanism of DEPDC1B in CCA is still a mystery.

In this study, the level of DEPDC1B in CCA was detected through immunohistochemistry analysis, showing that 1) the expression of DEPDC1B in CCA tissues is observably higher than in normal tissues and 2) CCA tissues with a more advanced malignant grade tend to express a higher DEPDC1B level. More evidence proving the promotion of CCA by DEPDC1B was provided by further biological studies, which displayed that DEPDC1B depletion could disturb the proliferation and migration capability of CCA cells while inducing cell apoptosis by regulating apoptosis- or epithelial–mesenchymal transition (EMT)-related proteins. Moreover, the inhibited tumorigenicity of CCA cells by DEPDC1B knockdown was also manifested *in vitro* by colony formation assay and *in vivo* by mouse xenograft models. The exploration of downstream mechanisms further recognized the involvement of CDK1, which is a critical regulatory factor in cell cycle and a well-known tumor promotor, in DEPDC1B-induced promotion of CCA. These results indicated the essential role of DEPDC1B in the development of CCA, which may act as an effective therapeutic target in the development of targeted drugs against CCA.

## Material and methods

### Cell lines and cell culture

Human cholangiocarcinoma cell lines HCCC-9810, QBC939, HUCCT1, and RBE were purchased from Hangzhou Bena Technology. Cells were cultured in 90% RPMI-1640 containing 10% FBS. All cells were grown in an incubator at 37°C with 5% CO_2_.

### Immunohistochemistry analysis and Ki-67 immunostaining

CCA tissue microarray (TMA, Cat. #GA802, Xian Alenabio Co., Ltd.) images of 78 spots were taken from 73 patients with cholangiocarcinoma, including 41 patients with eCCA, 27 patients with iCCA, and five patients with intrahepatic bile duct tissue. Related information and written informed consent were also provided. The experiment was approved by the Ethics Committee of Shandong Provincial Hospital. For immunohistochemistry (IHC) staining, sections were incubated with primary anti-DEPDC1B (Cat. #bs-14356R, BIOSS) at 4°C overnight. A secondary antibody was added and incubated for 2 h at room temperature. The tissue microarrays were stained with diaminobenzidine and examined under a microscope with ×200 and ×400 objective. IHC scoring of specimens was classified into four categories based on the sum of the staining intensity (varied from weak to strong) and staining extent scores, graded as 0 (0%), 1 (1%–25%), 2 (26%–50%), 3 (51%–75%), or 4 (76%–100%).

For Ki-67 immunostaining, slides were incubated with antibody to Ki-67 (Cat. #Ab16667, Abcam) and the secondary antibody and stained with hematoxylin and eosin (Cat. #BA4041, BA4022, Baso). Stained slides were examined with a microscope. All antibodies used in IHC are listed in **Table S1**.

### Human apoptosis antibody array

Detection of related genes in the human apoptosis signaling pathway was performed using Human Apoptosis Antibody Array (R&D Systems) following the manufacturer’s instructions. Briefly, the transfected HCCC-9810 were collected, washed, and then lysed with lysis buffer and total proteins were extracted. Protein concentrations were measured using the BCA Protein Assay Kit (HyClone-Pierce). Each array antibody membrane was blocked, then incubated with protein samples (0.5 mg/ml) overnight at 4°C and continuously incubated with an HRP-linked streptavidin conjugate for 1 h. Enhanced chemiluminescence (ECL) (Amersham) was used for visualizing and signaling the spots.

### Construction of stable knockdown and overexpressed cells and transfection

Full-length human DEPDC1B and SMURF1 complementary DNA was amplified by PCR and cloned into a lentivirus vector (Shanghai Biosciences) for gene overexpression. Lentiviruses expressing shDEPDC1B or shCDK1 and shCtrl or empty vectors were purchased from Shanghai Biosciences, Co., Ltd. Designed sequences for DEPDC1B and CDK1 are detailed in **Table S2**. HCCC-9810 and QBC939 were chosen to establish stable DEPDC1B knockdown models, whereas HUCCT1 cells were used for stable DEPDC1B or CDK1 knockdown, stable DEPDC1B overexpression, and CDK1 knockdown experiments. Cells (2 × 10^5^) at 80% confluence were seeded into six-well dishes, and RNA-expressing BR-V-108 vectors (1 × 10^8^ TU/ml) were added for transfection using Lipofectamine 2000 Reagent (Thermo Fisher Scientific). After culturing for 72 h, cell infection efficiency was evaluated by microscopic fluorescence.

### RNA extraction and RT-qPCR

RNA from experiment cells were extracted using TRIzol Reagent (QIAGEN), and the quality of total RNA was evaluated using a NanoDrop 2000 spectrophotometer (Thermo Fisher Scientific) according to the manufacturer’s instructions. Total RNA (2.0 μg) was reverse transcribed into cDNA, and quantitative real-time PCR with related primers (shown in **Table S3**) was conducted using SYBR Green Master Mix Kit (Vazyme) on the Applied Biosystems 7500 Real-Time PCR system. GAPDH was utilized as internal control. A relative quantitative analysis in gene expression data was performed using the 2^−ΔΔCt^ method.

### Western blotting assay

For Western blotting (WB) assay, each experiment group’s cells were lysed in ice-cold IP lysis buffer and the total proteins were collected. The BCA Protein Assay Kit (HyClone-Pierce) was applied for protein concentrations detection. Equal amounts of proteins (20 µg) were separated by 10% SDS-PAGE (Invitrogen) and transferred onto PVDF membranes. After blocking with TBST solution containing 5% non−fat milk, the membranes were incubated with specific primary antibodies (**Table S1**) and fluorescently conjugated secondary antibodies, followed by the detection with enhanced chemiluminescence (ECL) (Amersham). The quantification of blots was performed by ImageJ and shown in the following corresponding blots as protein/GAPDH.

For Co-IP, cell lysate was prepared as WB assay, and 1.0–1.2 mg proteins were incubated with normal rabbit IgG (as control) for 2 h, followed by 2 h of incubation with 20-μl protein A/G beads. The cleared protein antibody bead complex was incubated at 100°C for 10 min. Then, the proteins in the immunocomplex were separated by 10% SDS-PAGE as WB assay and used for immunoblotting to identify interacting proteins.

### Flow cytometry for apoptosis and cell cycle

Apoptosis and cell cycle were detected using the Annexin V-APC Apoptosis Detection Kit (eBioscience, Cat. #88-8007) and Cell Cycle PI Staining Reagent (Sigma, Cat. #P4170), respectively. Lentivirus transfected cells were inoculated in six-well plates in triplicate and cultured for 5 days until cell confluence reached 85%. After being trypsinized, cells were harvested and stained with 5 μl Annexin V-APC for 15 min in the dark. For cell-cycle detection, cells were collected and stained with propidium Iodide. Apoptosis analysis and cell-cycle distribution were measured using FACSCalibur (BD Biosciences).

### Cell proliferation assay

Cell viability was measured using the MTT assay kit (Genview, Cat #JT343). Cells (2,000 cells/well) in the exponential growth phase were seeded into a 96-well plate. After incubation for 1, 2, 3, 4, and 5 days at 37°C, cells were stained with 20 μl MTT reagent (5 mg/ml) and OD490 values were measured by a microplate reader (Tecan).

### Celigo cell counting assay

Transfected HUCCT1 cells were cultured for 72 h, and then the cells were seeded into 96-well plates (2,000 cells/well) and further cultured for 5 days. A Celigo image cytometer (Nexcelom Bioscience) was applied for cell counting for 5 consecutive days, and a cell proliferation curve was drawn.

### Wound healing assay

Lentivirus transfected cells were seeded at 5 × 10^4^ cells per well onto 96-well dishes until cell confluence reached 90%. Scratches were made by a 96-wounding replicator (V&P Scientific). Images were captured at 0 and 24 h by a fluorescence microscope and analyzed using ImageJ software for the cell migration rate.

### Transwell assay

Migration assays were performed using 24-well plates inserted using an 8-μm-pore-size Transwell filter insert (Cat. #3422, Corning). Transfected HUCCT1 cells (5 × 10^4^ cells/well) were harvested and seeded in the 24-well plate and cultured for 24 h. Medium supplemented with 30% FBS was added in the lower chamber. Non-metastatic cells were removed with a cotton swab, cells within the membrane were fixed with 4% formaldehyde and stained with Giemsa, and the migration ability of cells was analyzed.

### GeneChip analysis

Gene expression profiling analysis was completed with Affymetrix GeneChip PrimeView Human Gene Expression Array according to the manufacturer’s instruction. Total RNA was extracted using the RNeasy Kit (Sigma). The concentration and values of A260/A280 of total RNA were determined using the NanoDrop 2000 (Thermo Fisher Scientific). The RNA integrity number (RIN) was evaluated with Agilent 2100 and Agilent RNA 6000 Nano Kit (Agilent). Raw data statistical significance assessment was accomplished using a Welch t-test with Benjamini–Hochberg FDR (|fold change| ≥ 2.0 and FDR < 0.05 as significant). Significant difference analysis and functional analysis based on Ingenuity Pathway Analysis (IPA) (Qiagen) were executed.

### *In vivo* tumorigenicity assay

Our animal study was reviewed and approved by the Ethics Committee of Shandong Provincial Hospital and performed at the Animal Center of Provincial Hospital Affiliated to Shandong First Medical University. Four-week-old BALB/c female nude mice were purchased from Beijing Vital River Laboratory Animal Technology Co., Ltd., and housed at 22°C with a 12-h light/dark cycle-controlled condition. For *in vivo* tumor formation, 0.2 ml stably expressing shDEPDC1B or shCtrl HUCCT1 cell suspension with a density of 2 × 10^7^ cells/ml was subcutaneously injected into 20 mice (randomly divided into shDEPDC1B group and shCtrl group). The growth of tumor was monitored, and tumor volume was recorded around 1 month later using L and W (L represents the longest dimension and W means the dimension perpendicular to length) and calculated as π/6×L×W^2^. Tumor volume was calculated two to three times weekly. Pentobarbital sodium was used as the anesthetics. Bioluminescence imaging by the IVIS Spectrum Imaging System (Perkin Elmer) was performed. Then, mice were sacrificed and the tumor tissues were removed for Ki-67 immunostaining.

### Statistical analysis

Data are expressed as the mean ± SD, and all statistical analysis was performed using SPSS 17.0 (IBM) or GraphPad Prism 6.01 (GraphPad Software). *P*-values were determined using Student’s T-test or one-way ANOVA. The DEPDC1B expression difference between cholangiocarcinoma tissues and adjacent normal tissues was analyzed with rank-sum test analysis. The relation of DEPDC1B expression and tumor characteristics in cholangiocarcinoma patients was analyzed with Mann–Whitney U analysis and Spearman rank correlation analysis. *P* < 0.05 was considered statistically significant.

## Results

### DEPDC1B was upregulated in CCA tissues and highly expressed in CCA cells

**Figure 1:**
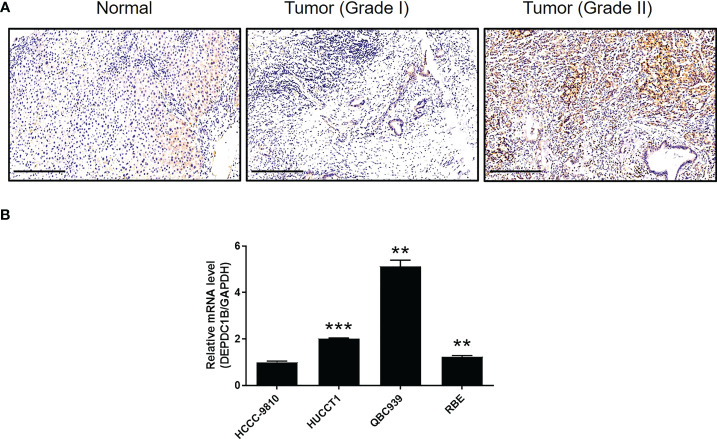
DEPDC1B was upregulated in CCA tissues and expressed in CCA cells. **(A)** The expression level of DEPDC1B was detected by IHC analysis in CCA tissues and normal tissues (scale bar = 250 μm). **(B)** The mRNA expression of DEPDC1B in HCCC-9810, HUCCT1, QBC939, and RBE cell lines was detected by qPCR. Data were shown as mean ± SD (n = 3). **P < 0.01, ***P < 0.001.

We aimed to explore the role of DEPDC1B in CCA. First, IHC analysis was used to reveal differences in the expression of DEPDC1B between CCA tissues and normal tissues; these analyses indicated the upregulated expression of DEPDC1B in CCA (**Figure 1A**). The statistical analysis of expression data collected from 73 CCA tissues and five normal tissues also highlighted the significantly higher expression levels of DEPDC1B in CCA (*P* < 0.001, **Table 1**). A correlation analysis between DEPDC1B expression and the clinical parameters of patients with CCA revealed that DEPDC1B expression was significantly upregulated in patients with a more advanced tumor grade (*P* < 0.05, **Figure 1A** and **Table 2**); these findings were further verified by Spearman rank correlation analysis (**Table S4**). Collectively, the higher expression levels of DEPDC1B in more serious cases of CCA suggested the potential role of DEPDC1B in the promotion of CCA. In addition, the endogenous expression of DEPDC1B in CCA cell lines including HCCC-9810, HUCCT1, QBC939, and RBE was detected by qPCR, exhibiting the expression of DEPDC1B in all cell lines (**Figure 1B**).

**Table 1:** Expression patterns of DEPDC1B in CCA tissues and normal tissues revealed in immunohistochemistry analysis.

DEPDC1B expression	Tumor tissue		Normal tissue	
	Cases	Percentage	Cases	Percentage
Low	31	42.5%	5	100%
High	42	57.5%	0	–

P < 0.001.

**Table 2:** Relationship between DEPDC1B expression and tumor characteristics in patients with CCA.

Features	No. of patients	DEPDC1B expression		*P* value
		Low	High	
All patients	73	31	42	
Age (years)				0.381
<59	35	13	22	
≥59	38	18	20	
Gender				0.593
Male	38	15	23	
Female	35	16	19	
Grade				<0.001
I	10	9	1	
II	37	18	19	
III	22	3	19	
Lymphatic metastasis (N)				0.652
N0	57	25	32	
N1	16	6	10	
T Infiltrate				0.073
T1	6	4	2	
T2	34	16	18	
T3	30	11	19	
T4	3	0	3	

### The depletion of DEPDC1B inhibited the development of CCA *in vitro*

**Figure 2:**
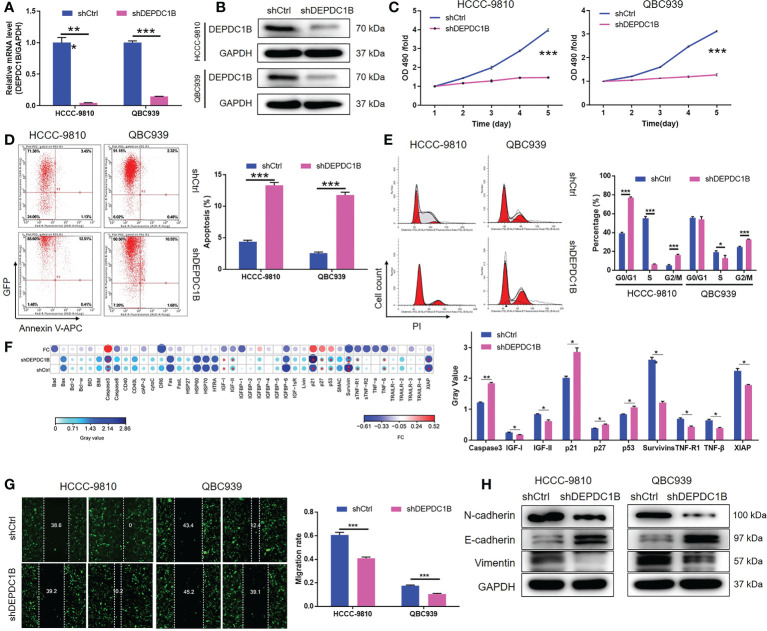
DEPDC1B knockdown inhibited CCA development *in vitro*. **(A, B)** Cell models with or without DEPDC1B knockdown were constructed by transfecting shDEPDC1B or shCtrl. The knockdown efficiency of DEPDC1B in HCCC-9810 and QBC939 cells was assessed by qPCR **(A)** and Western blotting **(B)**. **(C)** MTT assay was employed to show the effects of DEPDC1B on cell proliferation of HCCC-9810 and QBC939 cells. **(D)** Flow cytometry was performed to detect the cell apoptosis of HCCC-9810 and QBC939 cells with or without DEPDC1B knockdown. **(E)** Cell-cycle distribution was estimated in HCCC-9810 and QBC939 cells with or without DEPDC1B knockdown. **(F)** Human Apoptosis Antibody Array was performed to detect and compare the expression of apoptosis-related proteins in HCCC-9810 cells with or without DEPDC1B knockdown. **(G)** The effects of DEPDC1B on the cell migration ability of HCCC-9810 and QBC939 cells were evaluated by wound healing assay. **(H)** Western blotting was performed to show the effects of DEPDC1B knockdown on expression of EMT-related proteins. The representative images were selected from at least three independent experiments. Data were shown as mean ± SD (n = 3). *P < 0.05, **P < 0.01, ***P < 0.001.

We constructed a cell model of DEPDC1B deficiency by designing lentiviral vectors to silence DEPDC1B and thus allow us to investigate the role of this protein in CCA. Fluorescence signals were observed in >80% of cells, thus providing evidence of a successful transfection (**Figure S1**). Moreover, the significant downregulation of DEPDC1B mRNA and protein levels was demonstrated by qPCR (*P* < 0.001) and Western blotting (**Figures 2A, B**), respectively, thus confirming the successful knockdown of DEPDC1B in both HCCC-9810 and QBC939 cells. MTT assays further showed that cells in which DEPDC1B had been depleted (shDEPDC1B) grew significantly slower than those without DEPDC1B depletion (shCtrl) (*P* < 0.001, **Figure 2C**). Apoptosis is another key factor in cell proliferation; we used flow cytometry to determine cell apoptosis in CCA cells with or without DEPDC1B knockdown. As expected, cells with DEPDC1B deficiency exhibited a significantly larger population of apoptotic cells than the shCtrl group (*P* < 0.001, **Figure 2D**). Analysis of the cell-cycle distribution demonstrated that the downregulation of DEPDC1B in HCCC-9810 and QBC939 cells led to cell-cycle arrest in the G2 phase (*P* < 0.001, **Figure 2E**). In order to further investigate the mechanism underlying the ability of DEPDC1B to regulate cell apoptosis, we performed an antibody array to identify apoptosis-related proteins that exhibit interactions with DEPDC1B. We demonstrated that DEPDC1B depletion induced the upregulated expression of Caspase3, p21, p27, and p53 and the downregulation of IGF-I, IGF-II, Survivin, sTNF-R1, TNF-β, and XIAP (**Figure 2F**). We also used wound-healing assays to demonstrate the reduced mobility of HCCC-9810 and QBC939 cells in the shDEPDC1B group (*P* < 0.001, **Figure 2G**); this reduction was associated with the upregulation of E-cadherin and the downregulation of N-cadherin and Vimentin (**Figure 2H**). Collectively, these data suggested that DEPDC1B plays a vital role in the development of CCA by regulating cell proliferation and cell apoptosis. Moreover, CCA cell migration was influenced by DEPDC1B through the regulation of epithelial–mesenchymal transition-related proteins.

### DEPDC1B knockdown suppressed tumor growth *in vivo*

**Figure 3:**
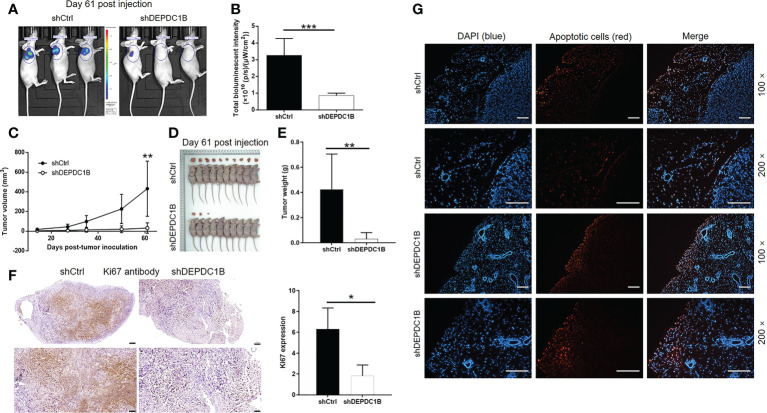
DEPDC1B knockdown inhibited CCA development *in vivo*. **(A, B)** *In vivo* imaging was performed to evaluate the tumor burden in mice of shDEPDC1B and shCtrl groups at day 61 post tumor inoculation. **(C)** Fourteen days post injection of HUCCT1 cells with or without DEPDC1B knockdown, the volume of tumors formed in mice was measured and calculated at indicated time intervals. **(D, E)** Mice were sacrificed at day 61 post injection, and the tumors were removed for collecting photos **(D)** and weighing **(E)**. **(F)** The expression of Ki67 in sections of xenografts was detected by IHC analysis (scale bar = 100 μm). **(G)** TUNEL analysis was performed in xenografts of shDEPDC1B and shCtrl groups (scale bar = 200 μm). Data were shown as mean ± SD (n = 3). *P < 0.05, **P < 0.01, ***P < 0.001.

We constructed and maintained a mouse model by injecting HUCCT1 cells, which possessed the highest efficiency in tumorigenesis *in vivo*, with or without a DEPDC1B knockdown vector. *In vivo* bioluminescence imaging revealed a significantly weaker total bioluminescence intensity, as well as a smaller tumor burden, in the shDEPDC1B group (*P* < 0.001, **Figures 3A, B**). Moreover, the smaller volume and weight of the solid tumors in the shDEPDC1B group also suggested that tumor growth slowed down when DEPDC1B was silenced (*P* < 0.01, **Figures 3C–E**). Furthermore, we observed a lower Ki67 index, as well as lower proliferative activity, in the tumors removed from mice in the shDEPDC1B group (**Figure 3F**). Furthermore, TUNEL analysis suggested that apoptosis of xenografts was much stronger in shDEPDC1B groups than in the control group (**Figure 3G**).

### DEPDC1B may regulate CCA by targeting CDK1

**Figure 4:**
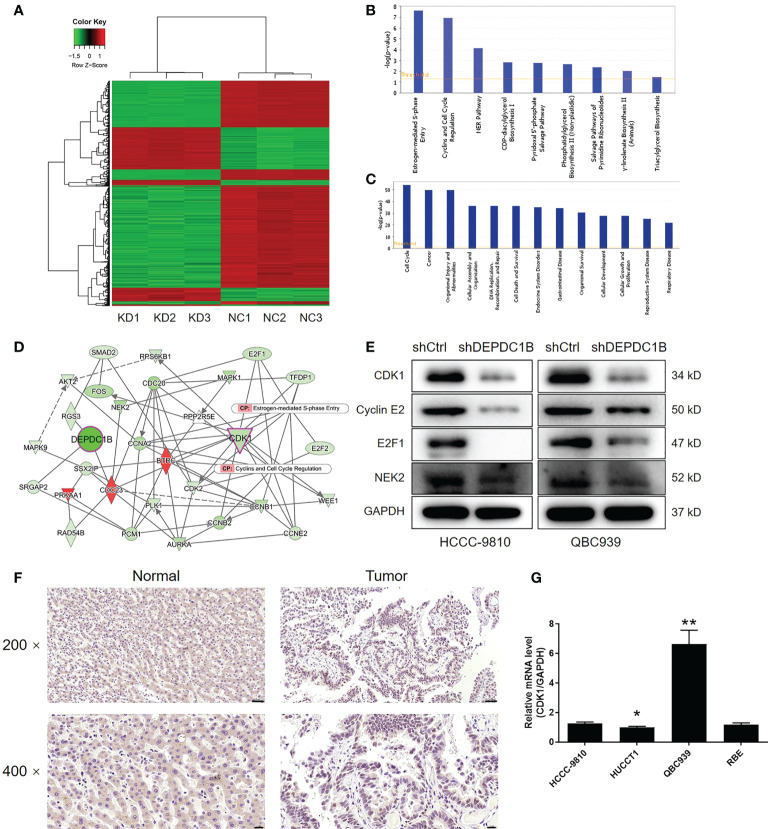
The exploration and verification of downstream underlying DEPDC1B-induced regulation of CCA. **(A)** A PrimeView Human Gene Expression Array was performed to identify the differentially expressed genes (DEGs) between the shDEPDC1B and shCtrl groups of HCCC-9810 cells. **(B, C)** The enrichment of DEGs in the canonical signaling pathway **(B)** and IPA disease and function **(C)** was analyzed by IPA. **(D)** A DEPDC1B-associated interaction network constructed by IPA revealed the potential linkage between DEPDC1B and CDK1. **(E)** Western blotting was used to detect the expression of several selected DEGs in HCCC-9810 and QBC939 cells with or without DEPDC1B knockdown. **(F)** The expression of CDK1 in CCA tissues and normal tissues was evaluated by IHC analysis (scale bar = 50 μm for ×200, 20 μm for ×400). **(G)** The mRNA expression of CDK1 in HUCCT1, QBC939, RBE. and HCCC-9810 cell lines was detected by qPCR. The representative images were selected from at least three independent experiments. Data were shown as mean ± SD (n = 3). *P < 0.05, **P < 0.01.

Given the clear regulatory role of DEPDC1B in CCA, we next attempted to identify the mechanisms underlying this effect. We conducted 3 v 3 RNA-seq to identify differentially expressed genes (DEGs) between cells in the shDEPDC1B group and the shCtrl group of HCCC-9810 cells. Based on a threshold of simultaneous |fold change| ≥ 2 and a FDR < 0.05 (the *P* value after Benjamini–Hochberg analysis), we identified 319 DEGs that were upregulated in shDEPDC1B cells compared with shCtrl cells and 828 DEGs that were downregulated (**Figure S2A**, **Figure 4A**). The enrichment of all 1,147 DEGs in canonical signaling pathways, or IPA disease and function, was assessed by IPA (**Figures 4B, C**). Based on bioinformatics and IPA of the DEPDC1B-associated interaction network built on the known interaction between related molecules (**Figures 4B–E**), several DEGs with the highest fold change in expression were subjected to verification by qPCR and Western blotting in HCCC-9810 and QBC939 cells (**Figures S2B**, **4D** and **S2C**). Of these, CDK1, a key member of the cyclin and cell-cycle regulation pathway, was considered to be a promising candidate as the target for DEPDC1B. Notably, the expression of CDK1 showed a similar expression pattern as DEPDC1B in CCA tissues in that higher expression levels were observed in CCA tissues than in normal tissues (**Figure 4F**). The abundant expression of CDK1 in CCA cells was also demonstrated by qPCR (**Figure 4G**). Collectively, these data indicated that CDK1 may represent a potential target for DEPDC1B during the regulation of CCA.

### DEPDC1B may regulate CDK1 expression through the ubiquitin–proteasome system

**Figure 5:**
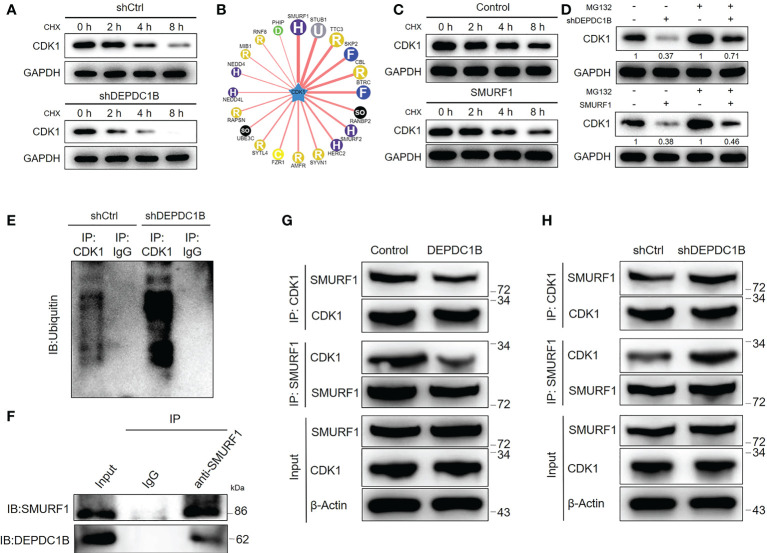
DEPDC1B regulates CDK1 expression through the ubiquitin–proteasome system. **(A)** The protein level of CDK1 was detected in shCtrl and shDEPDC1B HUCCT1 cells upon the treatment of cyclohexane (CHX) for indicated times. **(B)** The UbiBrowser online tool was used for predicting the E3 ligase of CDK1. **(C)** The protein level of CDK1 was detected in HUCCT1 cells with or without SMURF1 overexpression upon the treatment of cyclohexane (CHX) for indicated times. **(D)** The regulatory effects of the CDK1 protein level by DEPDC1B knockdown or SMURF1 overexpression were evaluated with or without treatment of proteasome inhibitor MG132. **(E)** An immunoprecipitation assay was performed for assessing the ubiquitination modification of CDK1 in shCtrl and shDEPDC1B HUCCT1 cells. **(F)** The interaction between DEPDC1B and SMURF1 was detected and verified by a co-immunoprecipitation assay using the IgG or SMURF1 antibody for obtaining a protein complex. **(G, H)** Total lysates of CCA cells with or without DEPDC1B overexpression **(G)** or DEPDC1B knockdown **(H)** were subjected to immunoprecipitation using anti-CDK1 or anti-SMURF1, followed by Western blotting analysis using indicated antibodies. All groups of cells were treated with MG132 to inhibit the function of proteasome.

Although the mechanism by which DEPDC1B regulates CDK1 on the RNA level is still unclear, we unexpectedly found that DEPDC1B knockdown could distinctly decrease the protein stability of CDK1 in HUCCT1 cells (**Figure 5A**), in which the treatment of cycloheximide (CHX) blocked the biosynthesis of proteins. Based on these, we proposed that DEPDC1B may regulate the protein level of CDK1 through the ubiquitin–proteasome system, which plays an important role in the degradation of endogenous proteins. Through the application of UbiBrowser (http://ubibrowser.ncpsb.org.cn/ubibrowser/home/index), CDK1 was predicted as a substrate of E3 ligase SMURF1 (**Figure 5B**). Indeed, the overexpression of SMURF1 could also decline the protein stability of CDK1 (**Figure 5C**). Moreover, the regulatory effects of DEPDC1B knockdown and SMURF1 overexpression on CDK1 protein level could be partially eliminated by the treatment of MG132, a proteasome inhibitor, indicating the potential involvement of the ubiquitin–proteasome system (**Figure 5D**). Accordingly, we revealed that knockdown of DEPDC1B could actually enhance the ubiquitination of CDK1 (**Figure 5E**). Therefore, we believed that DEPDC1B could upregulate the expression of CDK1 through inhibiting its ubiquitination as well as enhancing its protein stability, which may be resulted from the interaction between DEPDC1B and SMURF1 (**Figure 5F**). In addition, we also made some attempts to explore the underlying mechanism by which DEPDC1B regulates the SMURF1-mediated ubiquitination of CDK1. On the one hand, it was demonstrated that DEPDC1B knockdown/overexpression could downregulate/upregulate the mRNA and protein levels of SMURF1 in HUCCT1 cells (**Figure S3**). On the other hand, a series of immunoprecipitation assays showed that overexpression of DEPDC1B could apparently prevent the interaction between SMURF1 and CDK1 (**Figure 5G**), which was promoted by DEPDC1B knockdown (**Figure 5H**). Collectively, we believe that DEPDC1B may regulate the protein level of CDK1 through influencing SMURF1-mediated protein ubiquitination.

### CDK1 knockdown blocked the development of CCA *in vitro*

In order to identify the role of CDK1 in the development of CCA, we carried out a range of functional experiments following the construction of HUCCT1 cells in which CDK1 had been knocked down by lentivirus-delivered shCDK1–3 (**Figure S4**). Transfection and knockdown efficiencies were evaluated by a combination of fluorescence imaging, qPCR, and Western blotting (*P* < 0.001, **Figures S5** and **S6A**). Celigo cell counting assays indicated that the growth of HUCCT1 cells transfected with shCDK1 slowed down significantly whereas cells that had been transfected with shCtrl grew normally (*P* < 0.01, **Figure S6B**). We also counted the number of colonies formed by cells after 14 days of culture; there was a significant difference between the shCDK1 group (fewer colonies) and the shCtrl group (more colonies) (*P* < 0.001, **Figure S6C**). The effects of CDK1 knockdown on cell apoptosis were also similar to those induced by DEPDC1B knockdown; the proportion of apoptotic cells in the shCDK1 group increased by approximately 14-fold (*P* < 0.001, **Figure S6D**). Finally, wound-healing assays and Transwell assays showed that HUCCT1 cells in which CDK1 had been knocked down were significantly less motile (*P* < 0.001, **Figures S6E, F**). In summary, CDK1 exhibited similar regulatory effects on the development of CCA with DEPDC1B; however, the association between CDK1 and DEPDC1B remains unclear.

### CDK1 knockdown alleviated the regulatory effects of DEPDC1B overexpression on CCA

**Figure 6:**
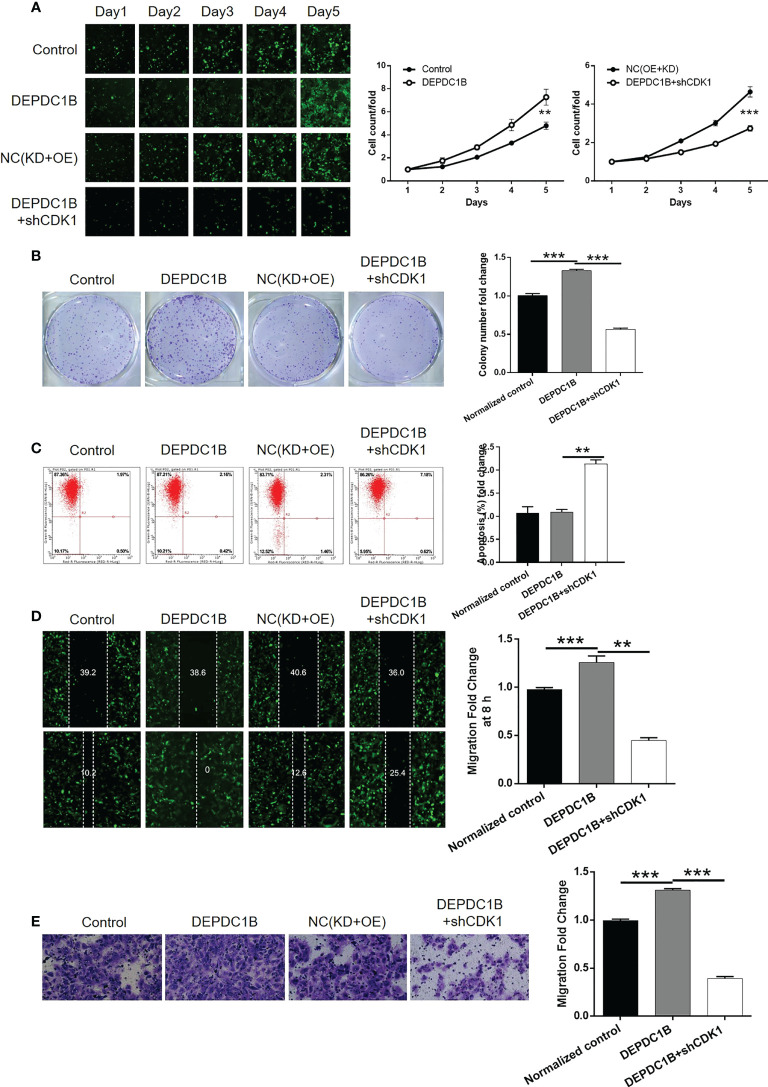
Knockdown of CDK1 attenuated the effects of CCA cells by DEPDC1B overexpression. HUCCT1 cells transfected with control plasmids, DEPDC1B overexpression plasmids, NC(OE+KD), and simultaneous DEPDC1B overexpression plasmids, and shCDK1 were subjected to the detection of cell proliferation by Celigo cell counting assay **(A)**, colony formation **(B)**, cell apoptosis by flow cytometry **(C)**, cell migration by wound healing assay **(D)**, and cell migration by Transwell assay **(E)**. The representative images were selected from at least three independent experiments. Data were shown as mean ± SD (n = 3). **P < 0.01, ***P < 0.001.

Next, we constructed HUCCT1 cells that overexpressed DEPDC1B and cells that overexpressed DEPDC1B but in which CDK1 had been knocked down. These cell groups were then used to investigate the synergistic effects of DEPDC1B and CDK1 on CCA. The transfection of HUCCT1 cells by DEPDC1B overexpression plasmids (**Figure S7A**) led to a significant upregulation of DEPDC1B (*P* < 0.001, **Figures S7B, C**) and a significantly faster rate of cell proliferation (*P* < 0.001, **Figure 6A**). We also found that the overexpression of DEPDC1B promoted the formation of colonies (*P* < 0.001, **Figure 6B**). Interestingly, we failed to observe the expected inhibition of cell apoptosis; this may have been attributed to the low apoptosis rate in the shCtrl group (**Figure 6C**). Furthermore, the overexpression of DEPDC1B promoted cell motility in HUCCT1 cells, as demonstrated by wound healing and Transwell assays (*P* < 0.001, **Figures 6D, E**). A comparative analysis of results produced by the DEPDC1B group (DEPDC1B overexpression) and the DEPDC1B+shCDK1 group (DEPDC1B overexpression + CDK1 knockdown) demonstrated that the effects of DEPDC1B overexpression on cell proliferation, colony formation, cell apoptosis, and cell migration could all be alleviated or even reversed by the knockdown of CDK1 (*P* < 0.001, **Figures S8** and **6A–E**). These results clearly indicate the effects of DEPDC1B overexpression on CCA and suggest that CDK1 may be involved.

## Discussion

Herein, we presented the first study reporting the role of DEPDC1B in CCA. In this study, it was found that DEPDC1B expression was higher in CCA tissues than in normal tissues, or in CCA tissues with a more advanced tumor grade, showing the potential participation of DEPDC1B in CCA development. The role of DEPDC1B was also proved by the subsequent *in vitro* investigation of the cell phenotype, which indicated the inhibition of cell proliferation, cell migration, the facilitation of cell apoptosis, and the arrest of cell cycle in the G2 phase. The simulation of *in vivo* CCA growth in the xenograft animal models further demonstrate the DEPDC1B knockdown-induced suppression of tumor growth.

A human apoptosis antibody array was utilized to explore how DEPDC1B regulates cell apoptosis and identified a variety of apoptosis-related proteins that are changed by DEPDC1B. For example, the shDEPDC1B group of CCA cells expressed a much lower level of Caspase3, an essential participator in cell apoptosis (25). Similarly, the suppression of cell apoptosis of CCA by DEPDC1B knockdown may also result from the downregulation of Survivin, which is a new member of the anti-apoptotic protein family (26). Meanwhile, some other apoptosis-associated factors such as IGF-I and IGF-II were also regulated by DEPDC1B. Otherwise, it has been well documented that EMT, which is an important biological process in development, specifically participates in the metastasis of malignant tumors (27, 28). In this study, the upregulation of epithelial cell marker E-cadherin and the downregulation of stromal cell markers N-cadherin and Vimentin in the shDEPDC1B group of CCA cells suggested that DEPDC1B may promote CCA cell migration through affecting the EMT process.

Human cells have more than 13 cyclin−dependent kinases (CDKs) and more than 25 cyclins that form a variety of CDK–cyclin complexes at different stages of the cell cycle, which exert a specific role in the process of the cell cycle (29). In addition, cyclin−dependent kinase 1 (CDK1) is an important modulator for a variety of biological behaviors, including cytoskeleton recombination, chromosome segregation, and daughter cell formation and separation (30). CDK1 participates in the regulation of the G2 phase as part of the cyclin A complex. For example, CDK1 is involved in G2/M transformation through the formation of a complex with cyclin B (31). The inactivation of CDK1 induces G2/M arrest in the cell cycle and the production of polyploid cells (32). Importantly, CDK1 was identified to be upregulated in a variety of malignant tumors, such as colorectal cancer (33), prostate cancer (34), bladder cancer (35), ovarian cancer (36), and breast cancer (37). Additionally, a high expression of CDK1 predicts a poor prognosis of pancreatic ductal adenocarcinoma (38). Yamamura et al. put forward that expression of p-CDK1 may be a prognostic indicator of CCA and that the CDK pathway may be a therapeutic target of CCA treatment (39). Therefore, the expression of CDK1 is closely correlated with the development and progress of various cancers including CCA, which makes it a potential promising target for molecular targeted therapy. In this study, based on mechanistic research, CDK1 was proposed as the downstream target of DEPDC1B in the regulation of CCA, whose upregulation in CCA agreed with previous studies. More importantly, it was also clarified that the effects of DEPDC1B overexpression on CCA cell phenotypes, including cell proliferation, colony formation, apoptosis, and migration could be alleviated or even eliminated by the simultaneous knockdown of CDK1. All the above results suggested that CDK1 may mediate the DEPDC1B-induced regulation of CCA. Actually, CDK1 was also found to be a downstream effector in the DEPDC1B-related regulation of hepatocellular carcinoma (40). Furthermore, a previous report indicating the role of DEPDC1B in pancreatic cancer development through targeting the Akt/Gsk3b/Snail pathway also provides support for the alleviation of DEPDC1B overexpression-induced effects by CDK1 knockdown (41). More importantly, it has been previously indicated that CDK1 could be regulated by posttranslational modification such as ubiquitination (42). Herein, we also discovered that DEPDC1B may regulate the protein stability as well as protein level of CDK1 through influencing SMURF1-mediated CDK1 ubiquitination. However, the regulatory mechanism by which DEPDC1B regulates the mRNA level of CDK1 still needs to be explored and investigated.

In summary, the outcomes of this study provided a novel insight into the biological functions of DEPDC1B in the development and progression of CCA and preliminarily explored the underlying mechanism. Herein, DEPDC1B was identified as a tumor promotor in CCA, the knockdown of which could inhibit CCA development. Moreover, DEPDC1B formed a signal axis together with CDK1 to induce the promotion of CCA and could be considered a promising therapeutic target of CCA treatment.

## Data availability statement

The original contributions presented in the study are included in the article/**Supplementary Material**. Further inquiries can be directed to the corresponding author.

## Ethics statement

The experiment was approved by Ethics committee of Shandong Provincial Hospital. The patients/participants provided their written informed consent to participate in this study. This study was reviewed and approved by Ethics committee of Shandong Provincial Hospital.

## Author contributions

SX designed this program. SX, XW, and YQ operated the cell and animal experiments. PN and JS conducted the data collection and analysis. SX produced the manuscript which was checked by SX, JS, ZZ, and XW. All authors contributed to the article and approved the submitted version.

## Funding

This work was financially supported by the Jinan Clinical Medicine Science and Technology Innovation Plan (No. 201907073) and Natural Science Foundation of Shandong Province (No. ZR2022MH169).

## Conflict of interest

Author JS was employed by Origimed at the time of writing.

The remaining authors declare that the research was conducted in the absence of any commercial or financial relationships that could be construed as a potential conflict of interest.

## Publisher’s note

All claims expressed in this article are solely those of the authors and do not necessarily represent those of their affiliated organizations, or those of the publisher, the editors and the reviewers. Any product that may be evaluated in this article, or claim that may be made by its manufacturer, is not guaranteed or endorsed by the publisher.
