# Supplementary material for: DEPDC1B promotes development of cholangiocarcinoma through enhancing the stability of CDK1 and regulating malignant phenotypes
Source: Front Oncol. 2022 Dec 6;12:842205. doi: 10.3389/fonc.2022.842205 (PMC9769124; doi:10.3389/fonc.2022.842205)
Supplement: Supplementary file 1 [file DataSheet_1.zip › Original data 1/Figure 2D/QBC939/shCtrl-2.pdf]

Well Number: D11

Sample ID: D11

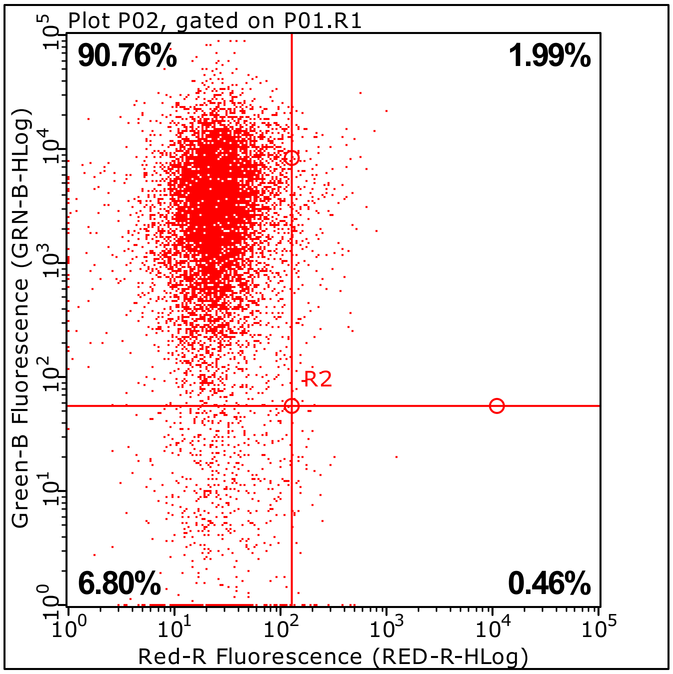

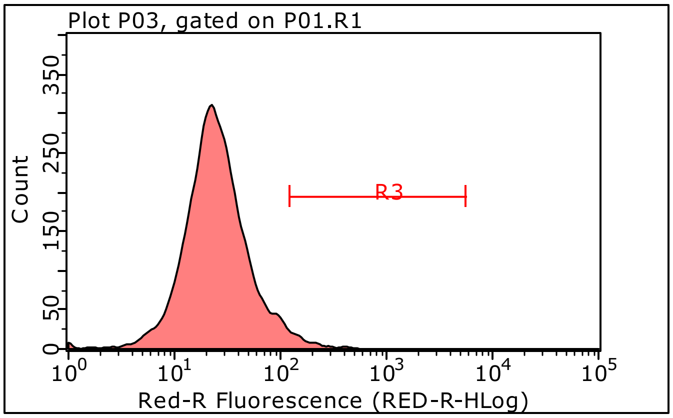

Well	Sample ID	Date	R2.Percent.UL Percent for R2 gated by P01.R1 (%)	R2.Percent.UR Percent for R2 gated by P01.R1 (%)	R2.Percent.LL Percent for R2 gated by P01.R1 (%)	R2.Percent.LR Percent for R2 gated by P01.R1 (%)
D11	D11	09.29.2018	90.76	1.99	6.80	0.46

Well	R3.Percent Percent for R3 gated by P01.R1 (%)
D11	2.45
