# Supplementary material for: DEPDC1B promotes development of cholangiocarcinoma through enhancing the stability of CDK1 and regulating malignant phenotypes
Source: Front Oncol. 2022 Dec 6;12:842205. doi: 10.3389/fonc.2022.842205 (PMC9769124; doi:10.3389/fonc.2022.842205)
Supplement: Supplementary file 1 [file DataSheet_1.zip › Original data 1/Figure 2D/QBC939/shDEPDC1B-2.pdf]

Well Number: E08

Sample ID: E08

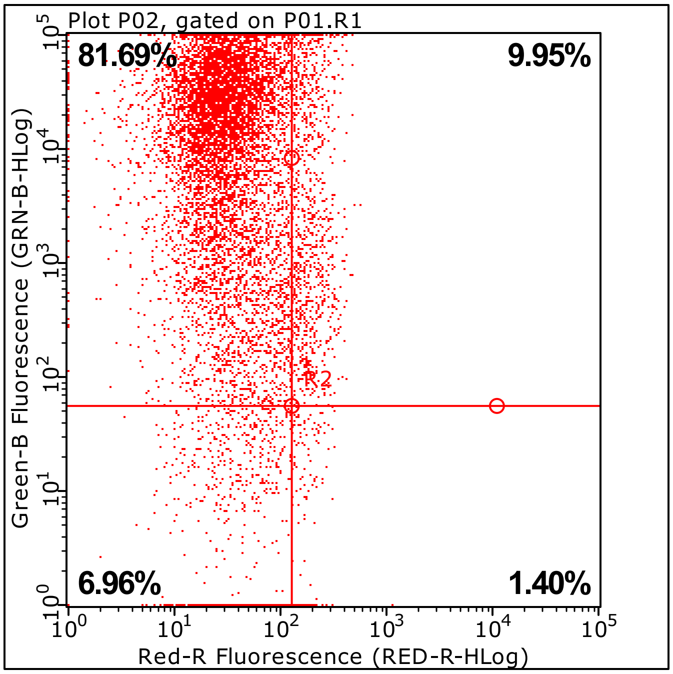


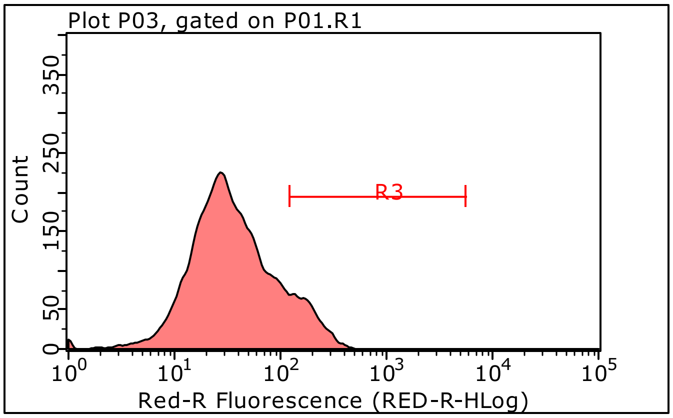

| Well | Sample ID | Date | R2.Percent.UL Percent for R2 gated by P01.R1 (%) | R2.Percent.UR Percent for R2 gated by P01.R1 (%) | R2.Percent.LL Percent for R2 gated by P01.R1 (%) | R2.Percent.LR Percent for R2 gated by P01.R1 (%) |
|---|---|---|---|---|---|---|
| E08 | E08 | 09.29.2018 | 81.69 | 9.95 | 6.96 | 1.40 |

| Well | R3.Percent Percent for R3 gated by P01.R1 (%) |
|---|---|
| E08 | 11.35 |
