# Supplementary material for: DEPDC1B promotes development of cholangiocarcinoma through enhancing the stability of CDK1 and regulating malignant phenotypes
Source: Front Oncol. 2022 Dec 6;12:842205. doi: 10.3389/fonc.2022.842205 (PMC9769124; doi:10.3389/fonc.2022.842205)
Supplement: Supplementary file 1 [file DataSheet_1.zip › Original data 1/Figure 6C/NC(OE+KD)-1.pdf]

Well Number: A07 Sample ID: A07
File Name: E:/wtw/20190808 HUCCT1 DW/2019-08-08_at_04-05-10pm.fcs

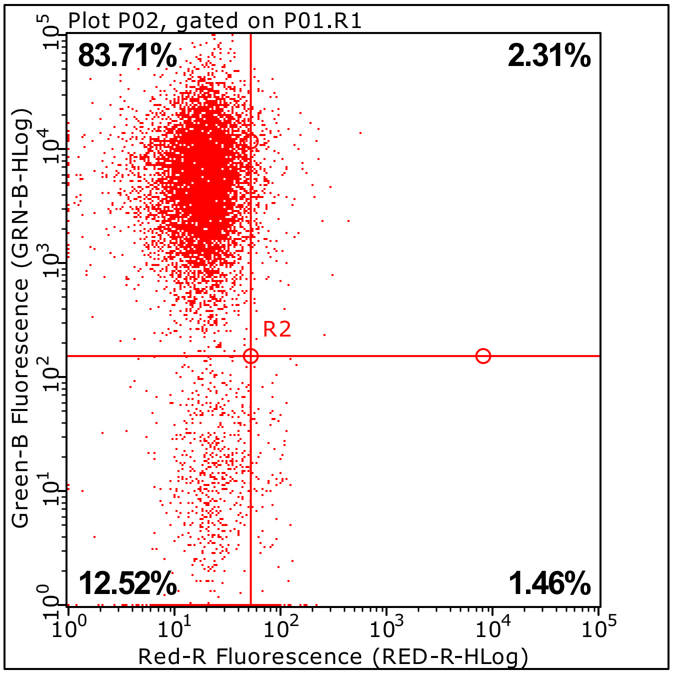

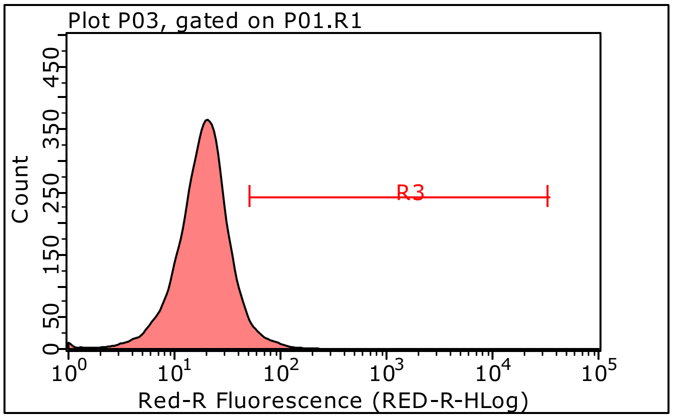

Well	Sample ID	Date	R2.Percent.UL Percent for R2 gated by P01.R1 (%)	R2.Percent.UR Percent for R2 gated by P01.R1 (%)	R2.Percent.LL Percent for R2 gated by P01.R1 (%)	R2.Percent.LR Percent for R2 gated by P01.R1 (%)
A07	A07	08.28.2019	83.71	2.31	12.52	1.46

Well	R3.Percent Percent for R3 gated by P01.R1 (%)
A07	3.78
