# Supplementary figures and images for: DEPDC1B promotes development of cholangiocarcinoma through enhancing the stability of CDK1 and regulating malignant phenotypes (part 2 of 2)
Source: Front Oncol. 2022 Dec 6;12:842205. doi: 10.3389/fonc.2022.842205 (PMC9769124; doi:10.3389/fonc.2022.842205)

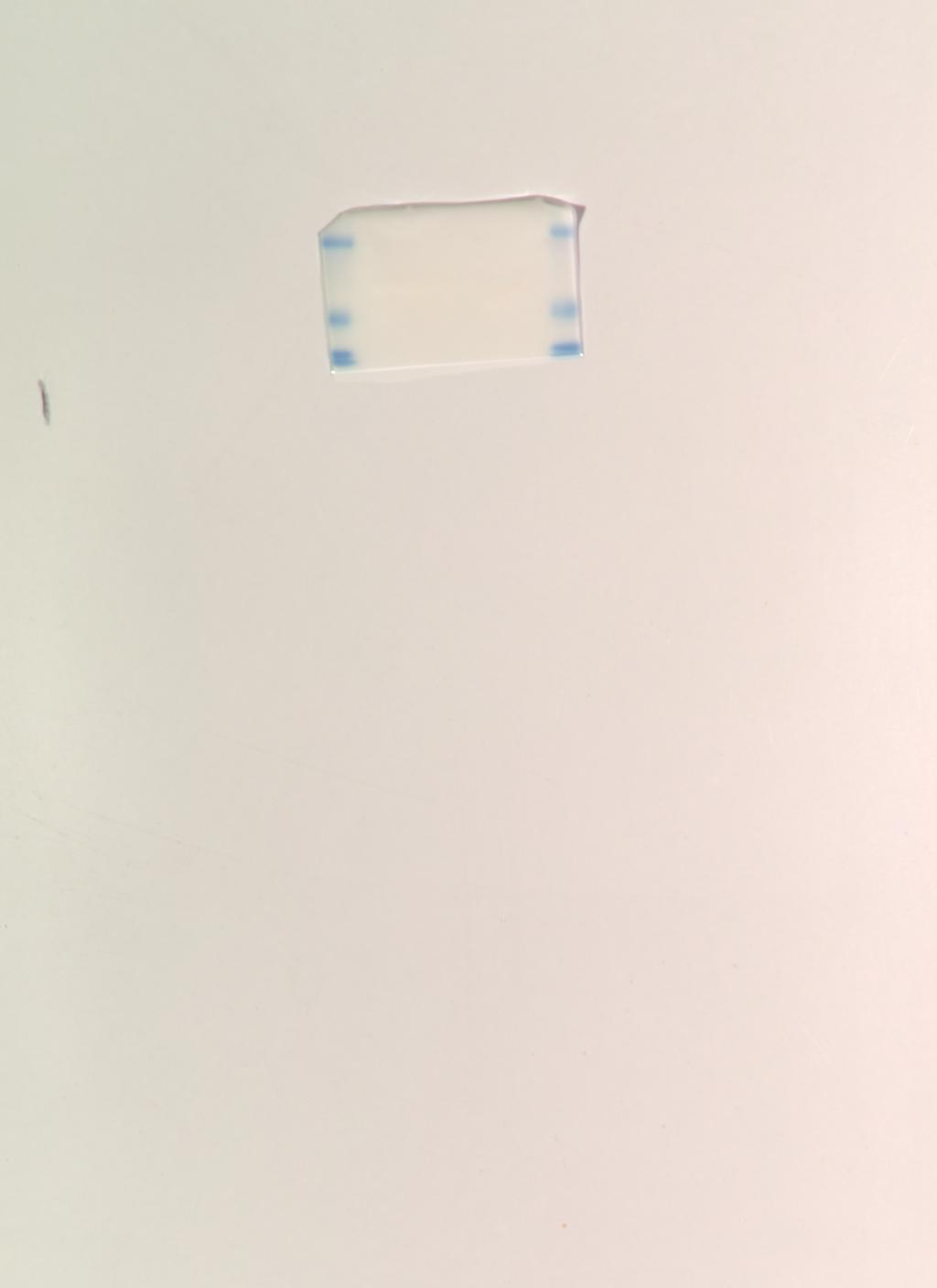

Supplement: Supplementary file 1 [file DataSheet_1.zip › Original data 1/Figure 5G/IP SMURF1/CDK1 M/CDK1 M.jpg]

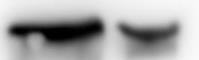

Supplement: Supplementary file 1 [file DataSheet_1.zip › Original data 1/Figure 5G/IP SMURF1/CDK1.jpg]

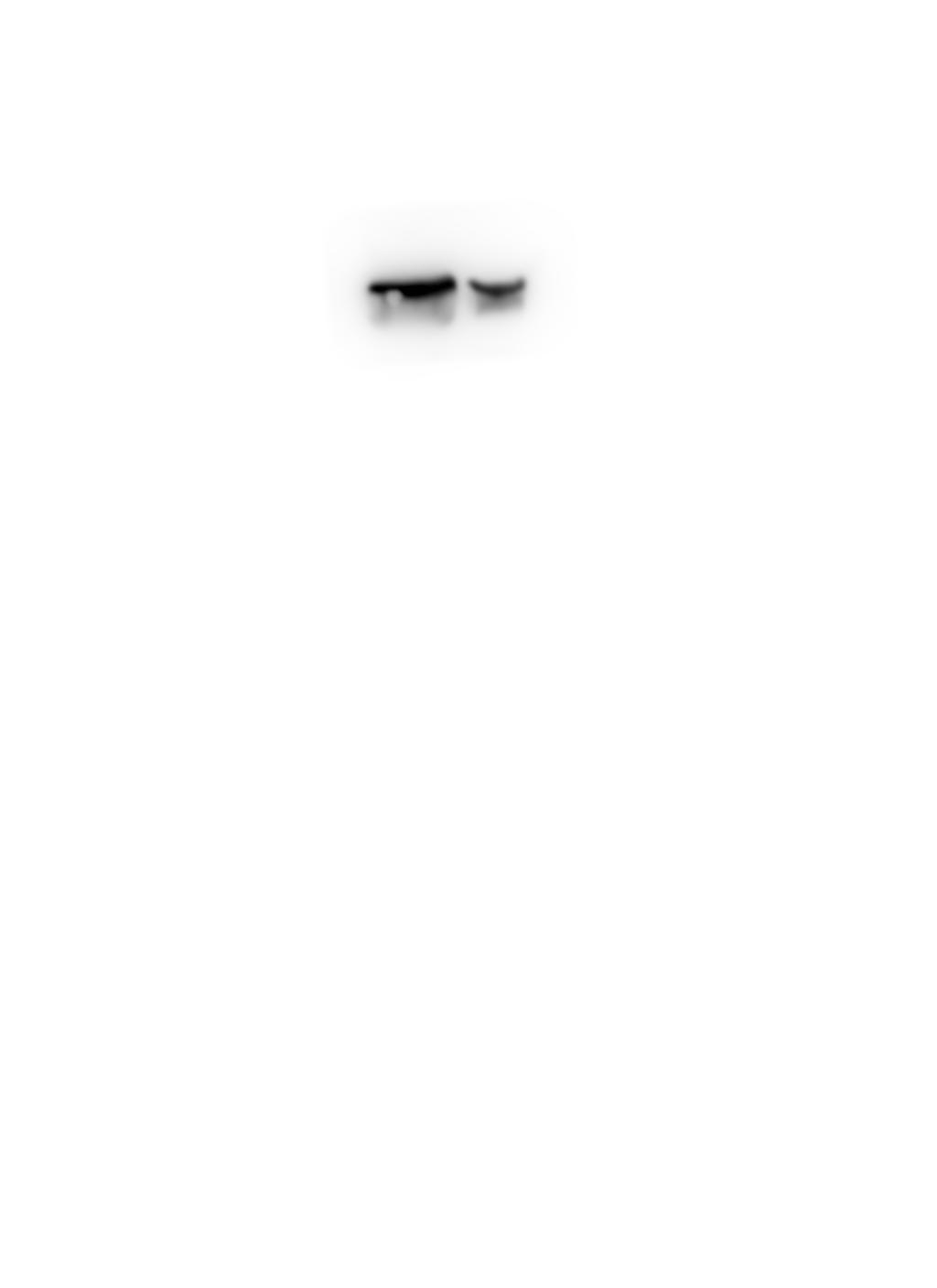

Supplement: Supplementary file 1 [file DataSheet_1.zip › Original data 1/Figure 5G/IP SMURF1/CDK1/CDK1.jpg]

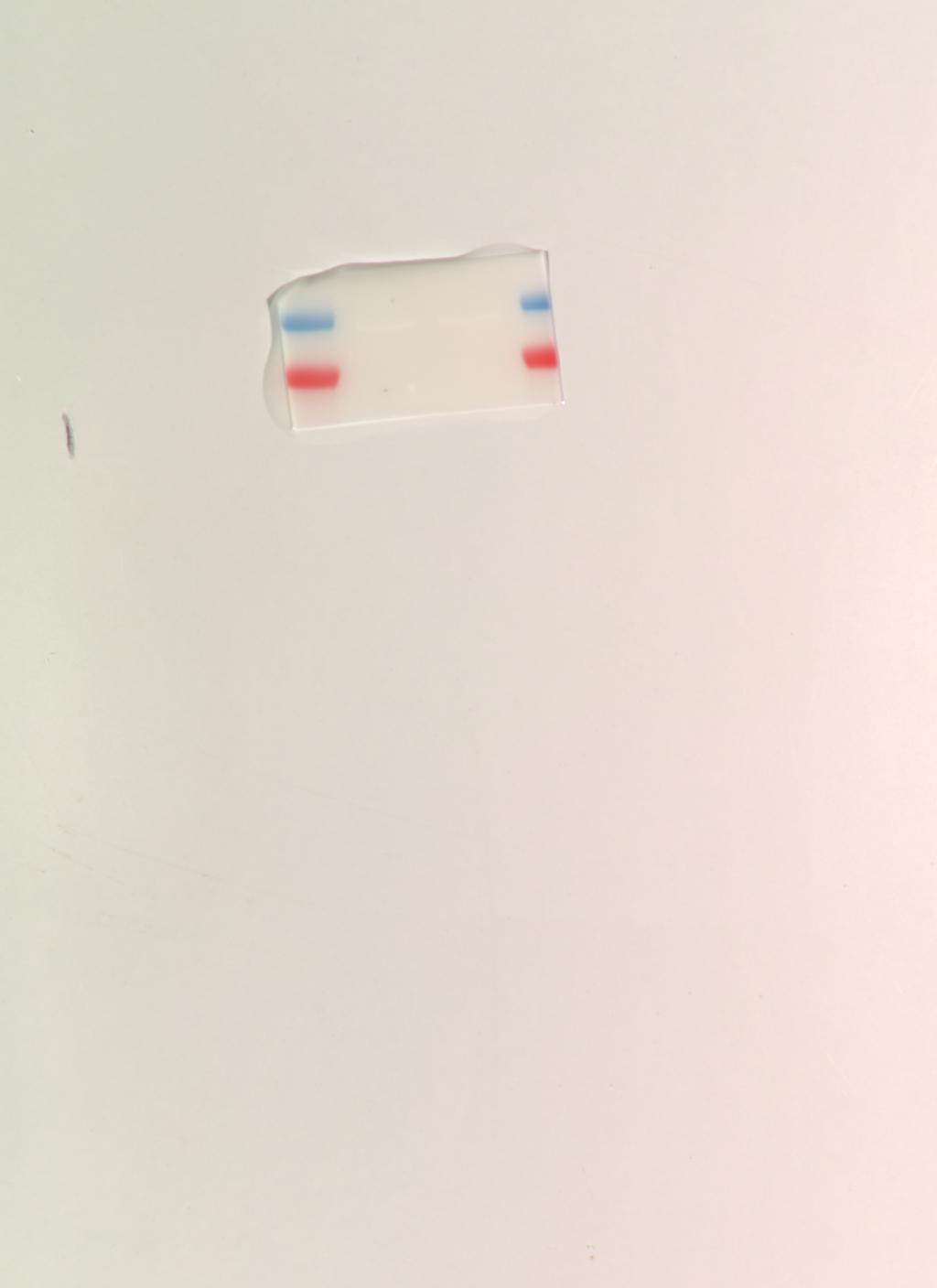

Supplement: Supplementary file 1 [file DataSheet_1.zip › Original data 1/Figure 5G/IP SMURF1/SMURF1 M/SMURF1 M.jpg]

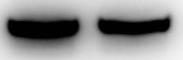

Supplement: Supplementary file 1 [file DataSheet_1.zip › Original data 1/Figure 5G/IP SMURF1/SMURF1.jpg]

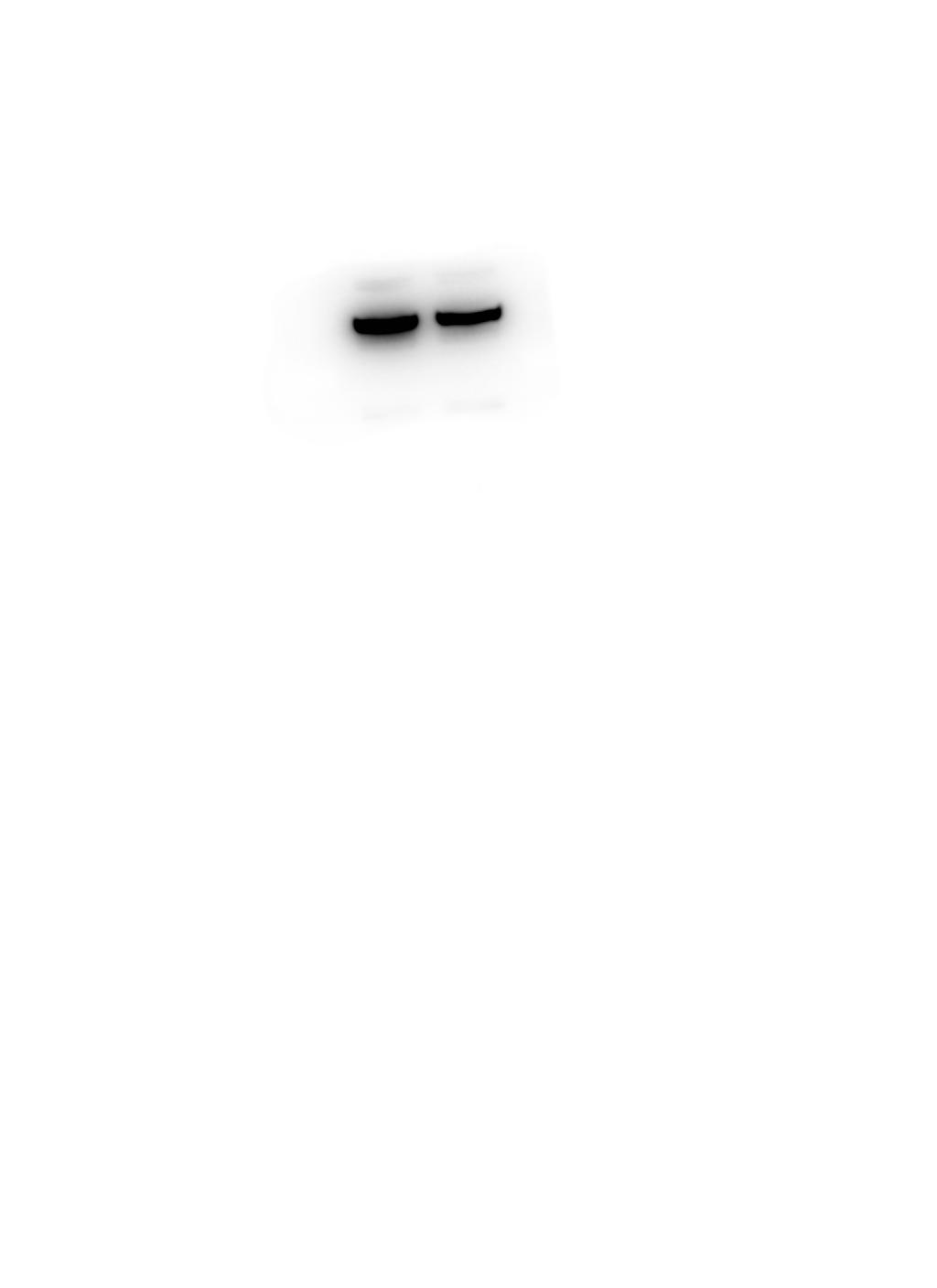

Supplement: Supplementary file 1 [file DataSheet_1.zip › Original data 1/Figure 5G/IP SMURF1/SMURF1/SMURF1.jpg]

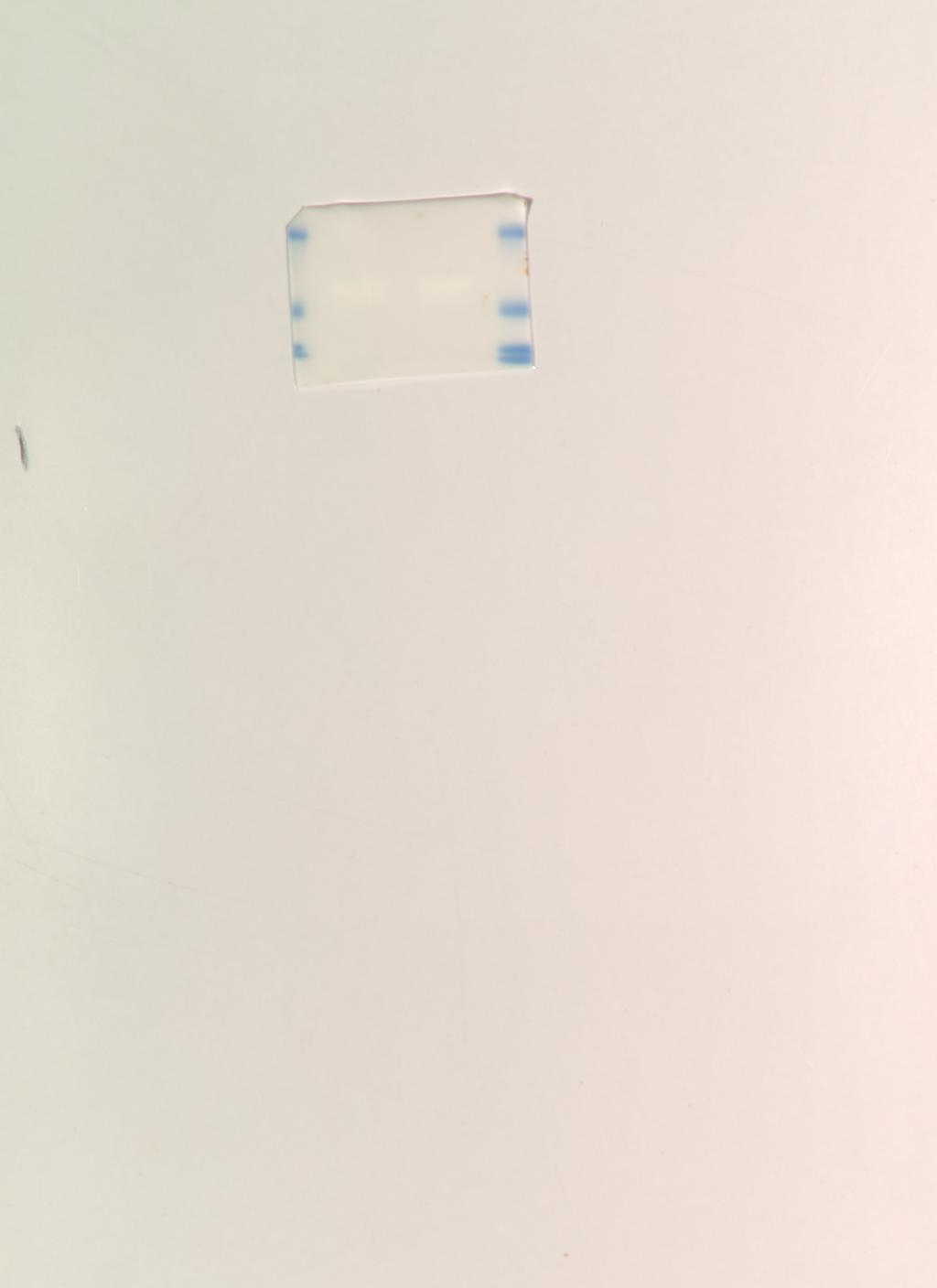

Supplement: Supplementary file 1 [file DataSheet_1.zip › Original data 1/Figure 5H/Input/CDK1 M/CDK1 M.jpg]

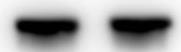

Supplement: Supplementary file 1 [file DataSheet_1.zip › Original data 1/Figure 5H/Input/CDK1.jpg]

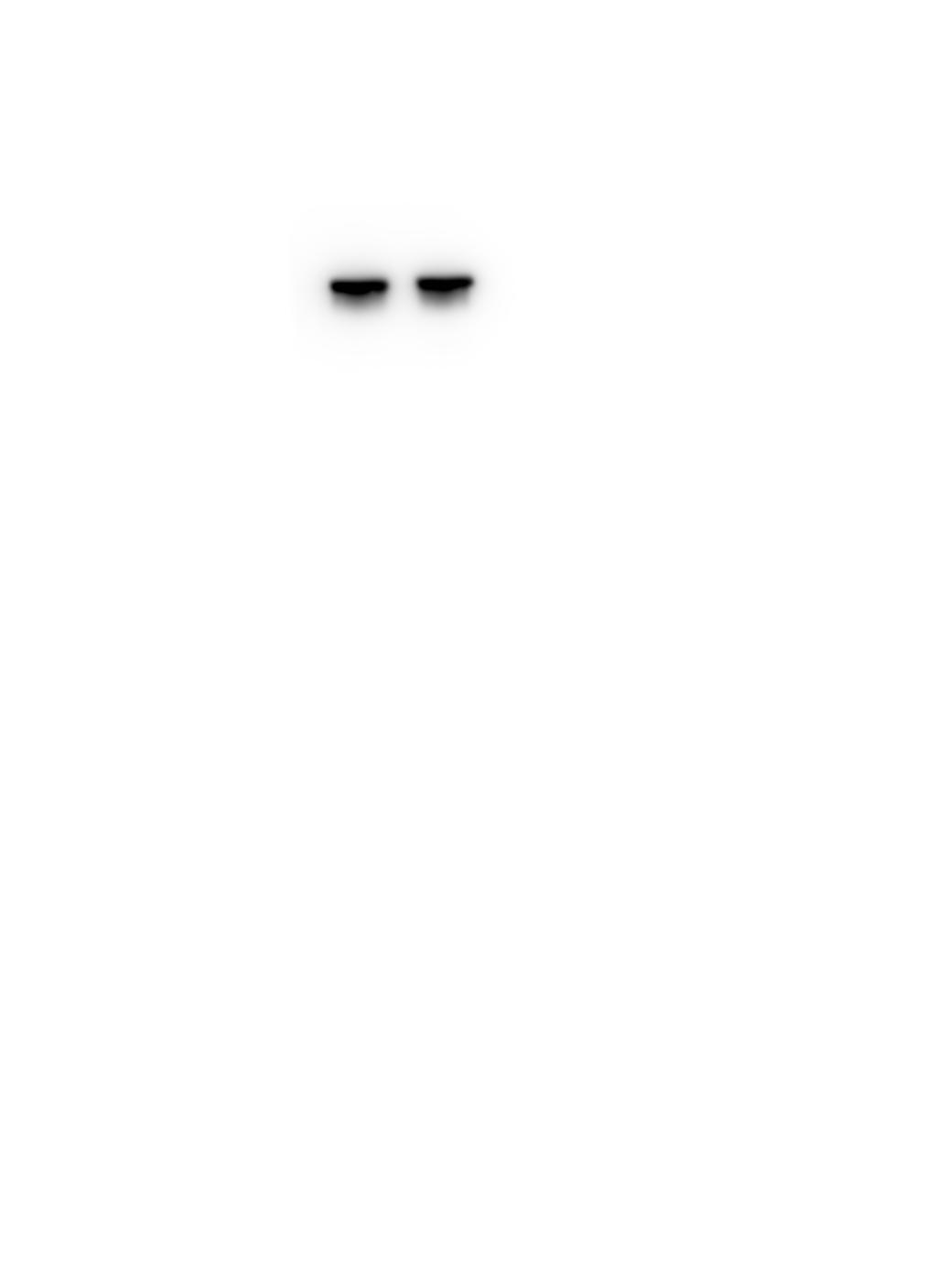

Supplement: Supplementary file 1 [file DataSheet_1.zip › Original data 1/Figure 5H/Input/CDK1/CDK1.jpg]

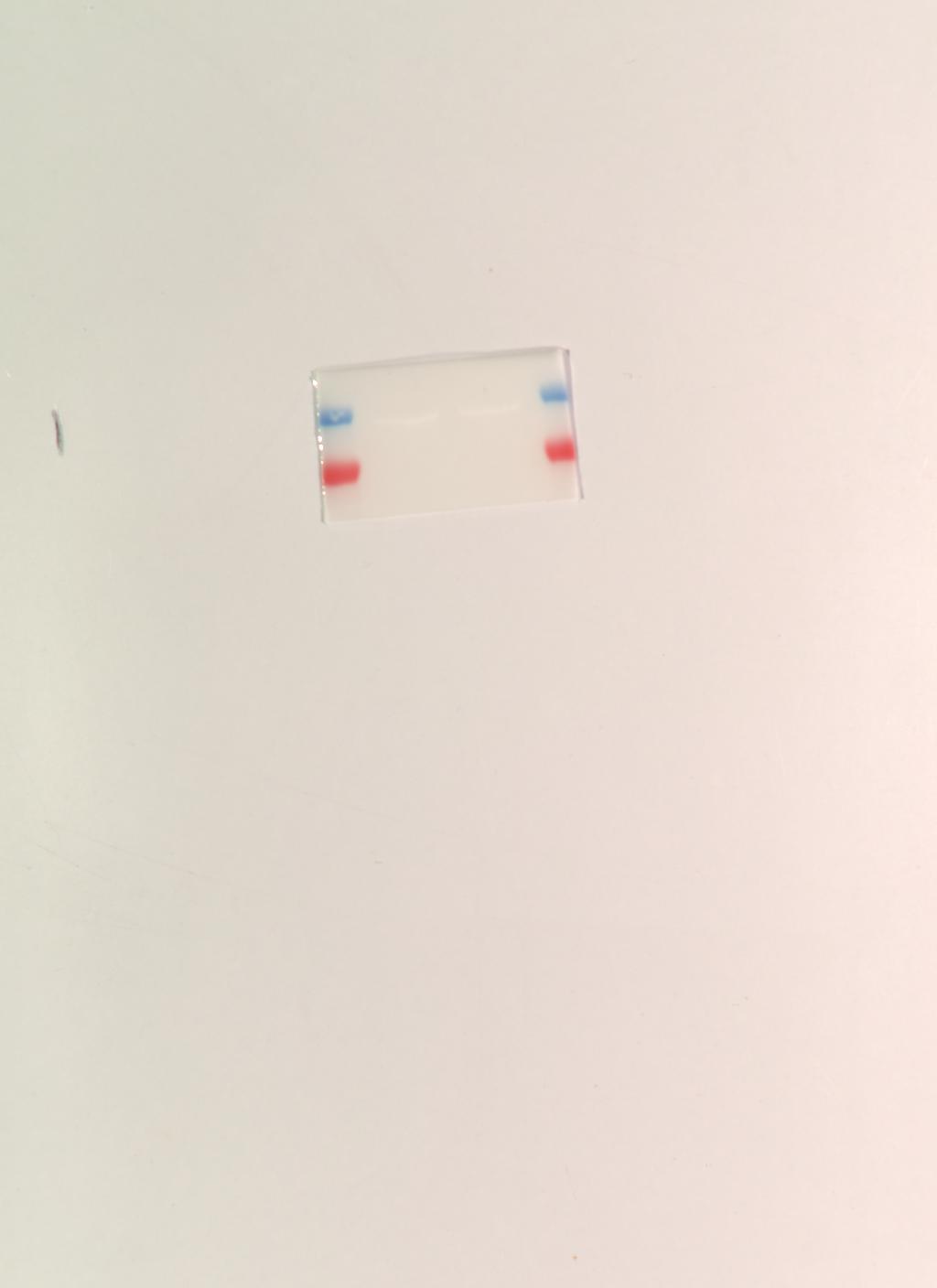

Supplement: Supplementary file 1 [file DataSheet_1.zip › Original data 1/Figure 5H/Input/SMURF1 M/SMURF1 M.jpg]

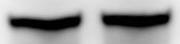

Supplement: Supplementary file 1 [file DataSheet_1.zip › Original data 1/Figure 5H/Input/SMURF1.jpg]

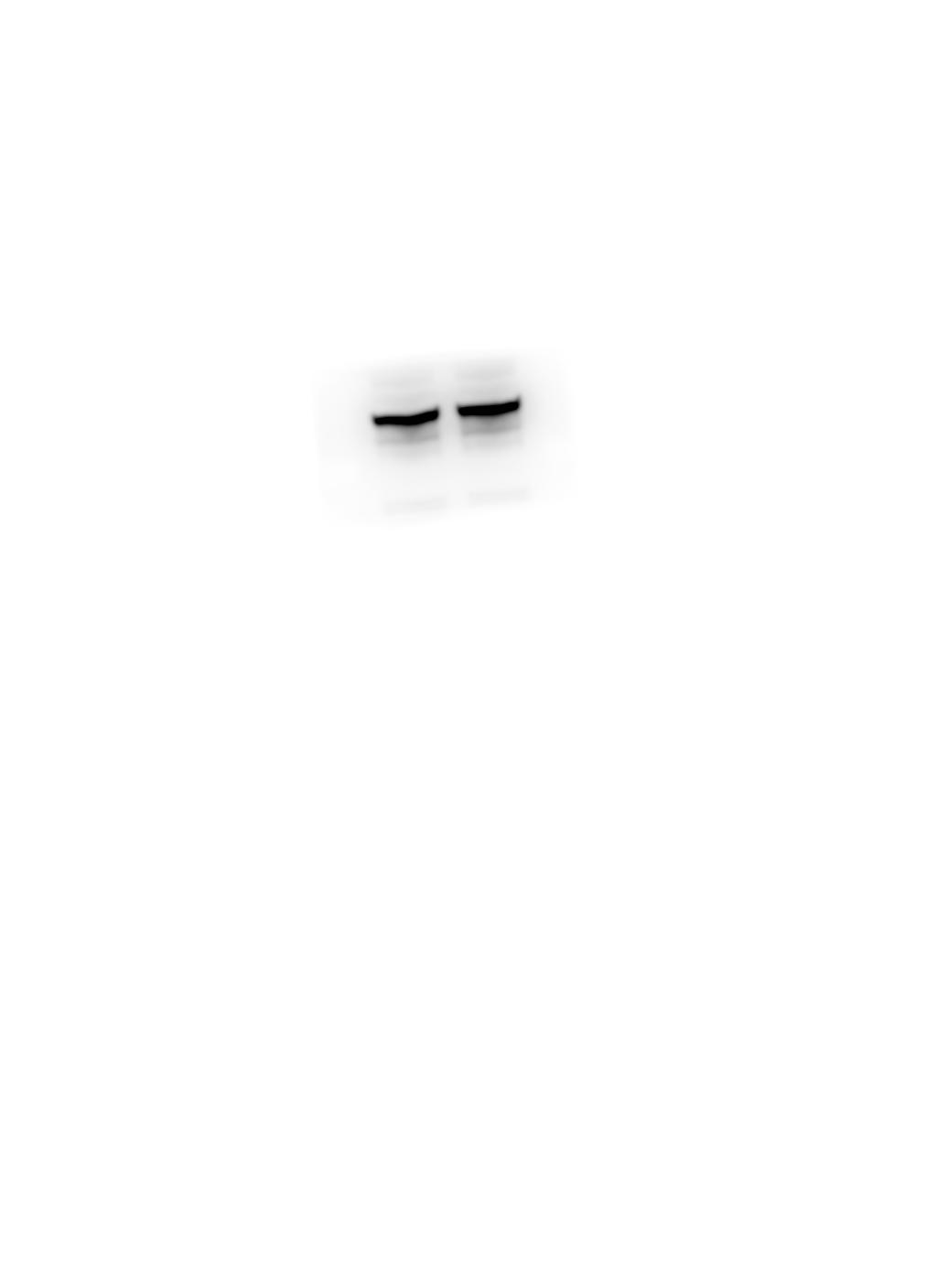

Supplement: Supplementary file 1 [file DataSheet_1.zip › Original data 1/Figure 5H/Input/SMURF1/SMURF1.jpg]

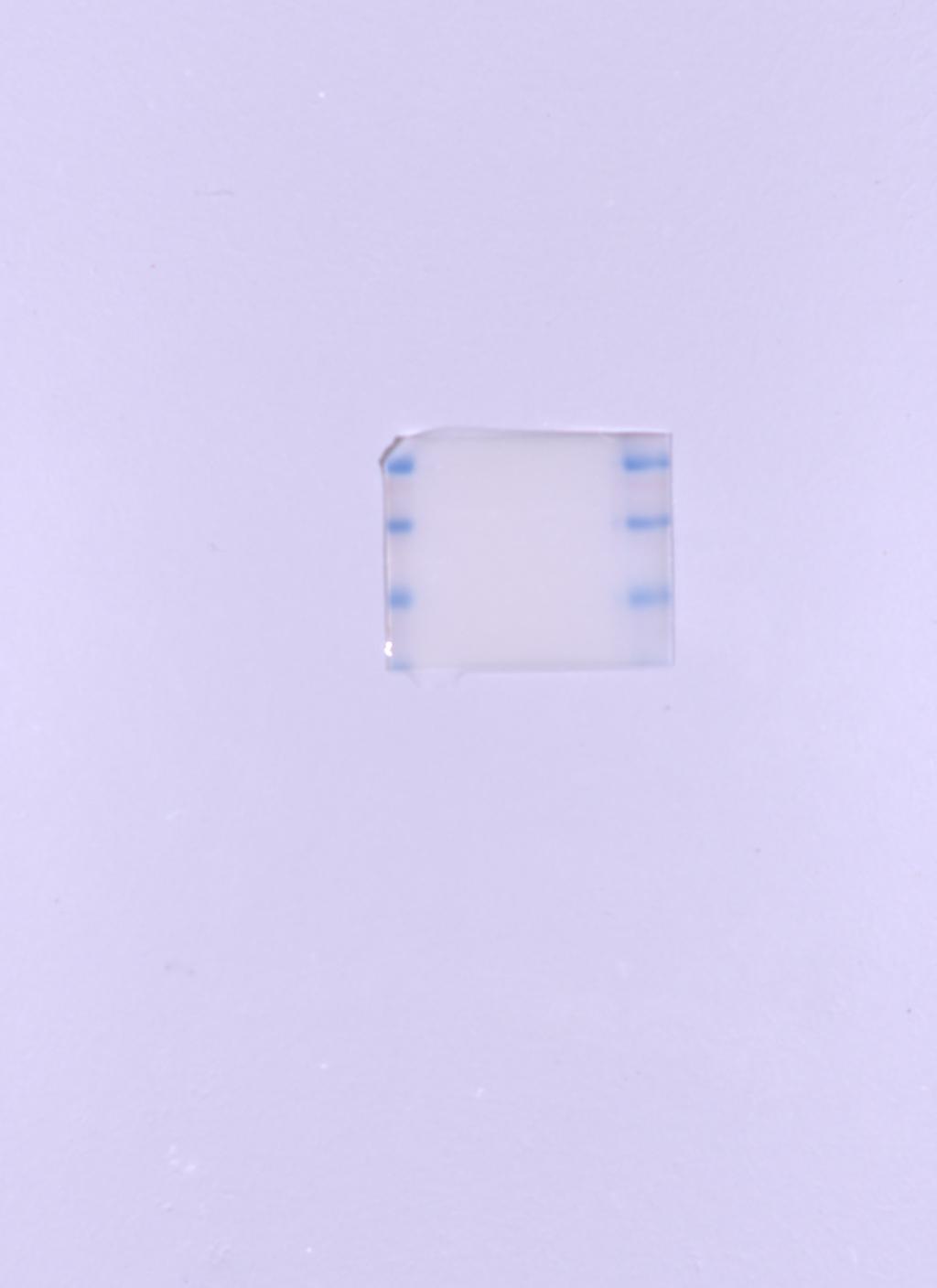

Supplement: Supplementary file 1 [file DataSheet_1.zip › Original data 1/Figure 5H/Input/β-Actin M/β-Actin M.jpg]

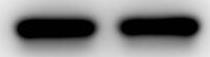

Supplement: Supplementary file 1 [file DataSheet_1.zip › Original data 1/Figure 5H/Input/β-Actin.jpg]

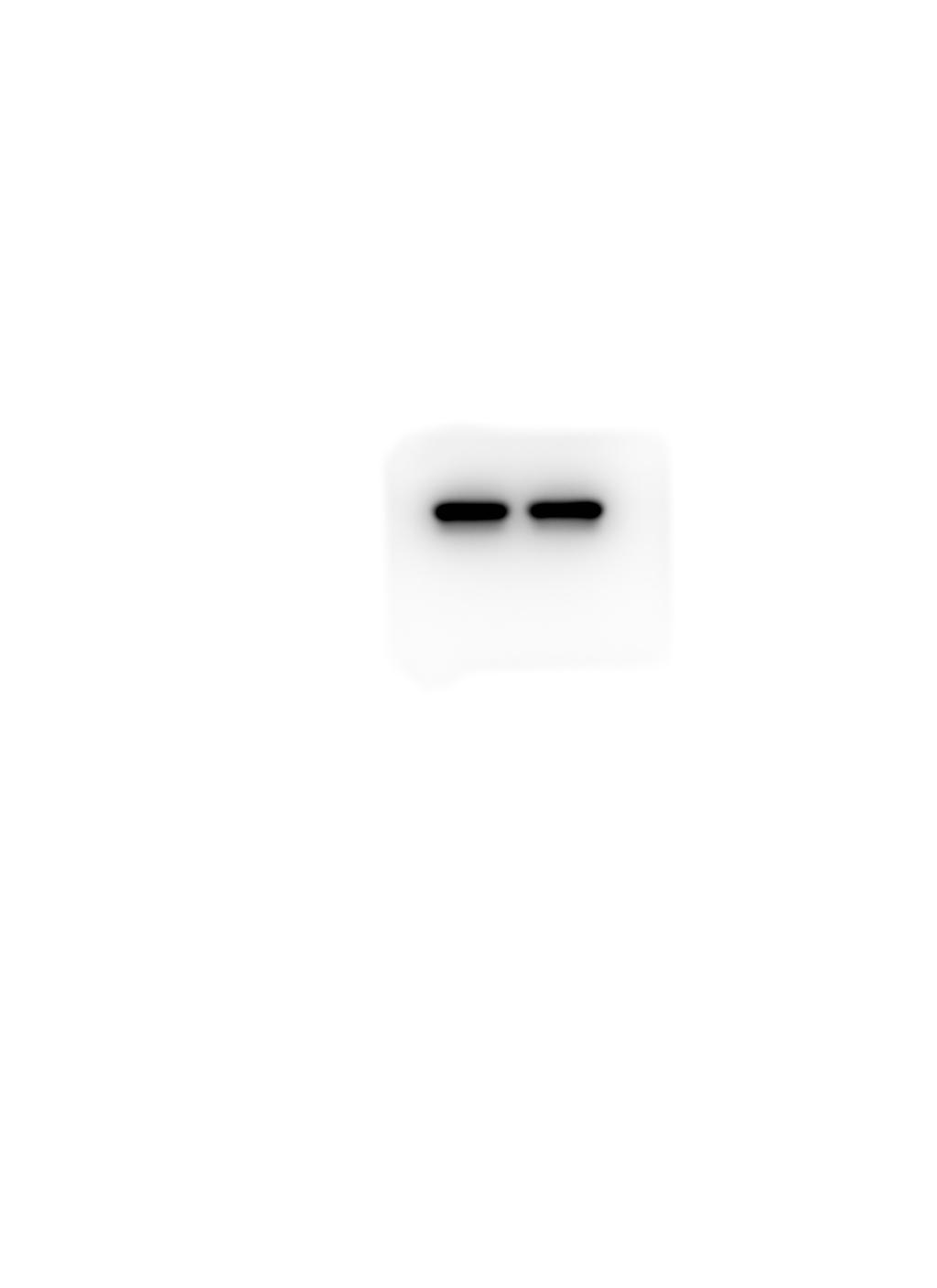

Supplement: Supplementary file 1 [file DataSheet_1.zip › Original data 1/Figure 5H/Input/β-Actin/β-Actin.jpg]

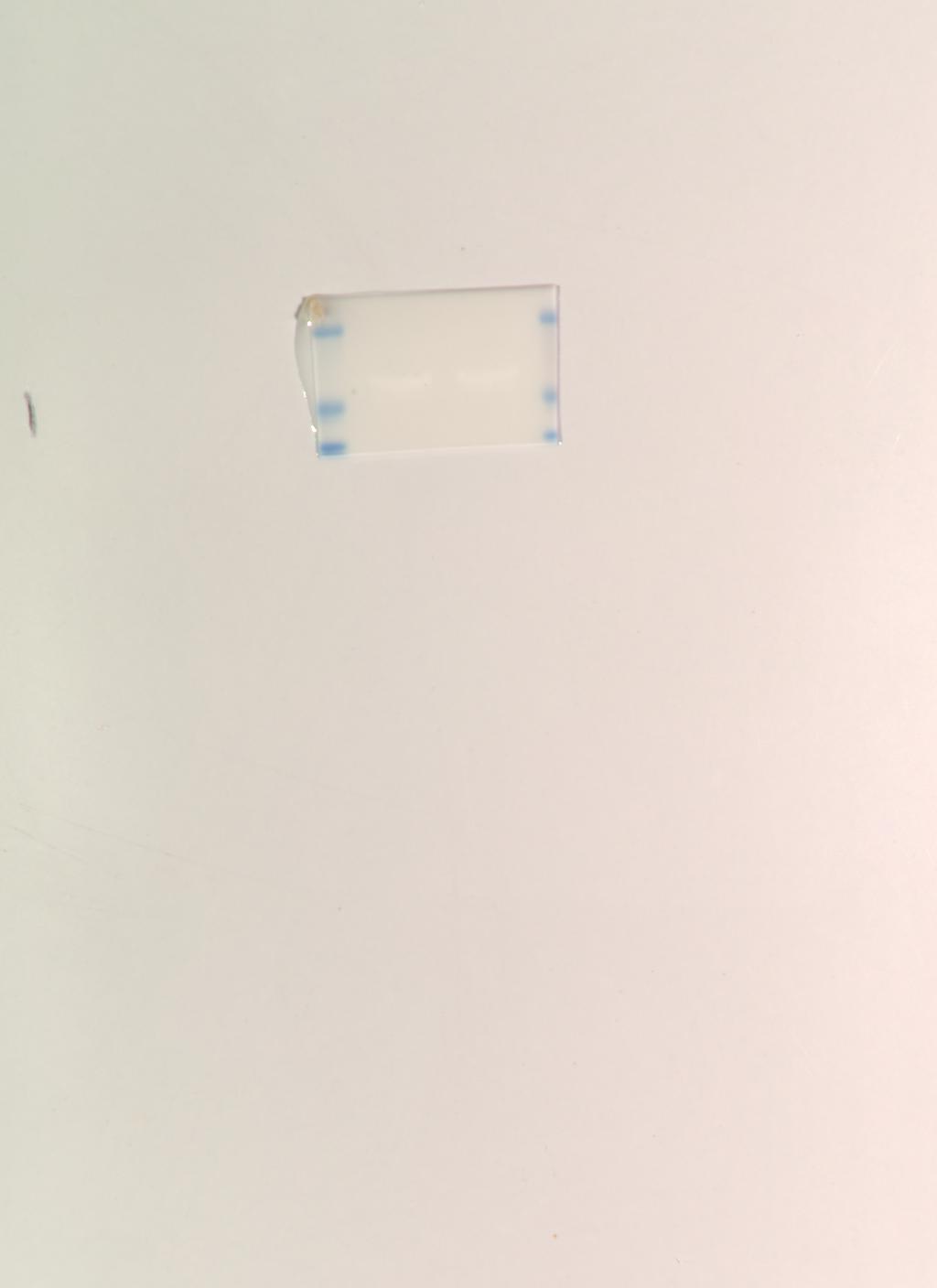

Supplement: Supplementary file 1 [file DataSheet_1.zip › Original data 1/Figure 5H/IP CDK1/CDK1 M/CDK1 M.jpg]

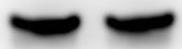

Supplement: Supplementary file 1 [file DataSheet_1.zip › Original data 1/Figure 5H/IP CDK1/CDK1.jpg]

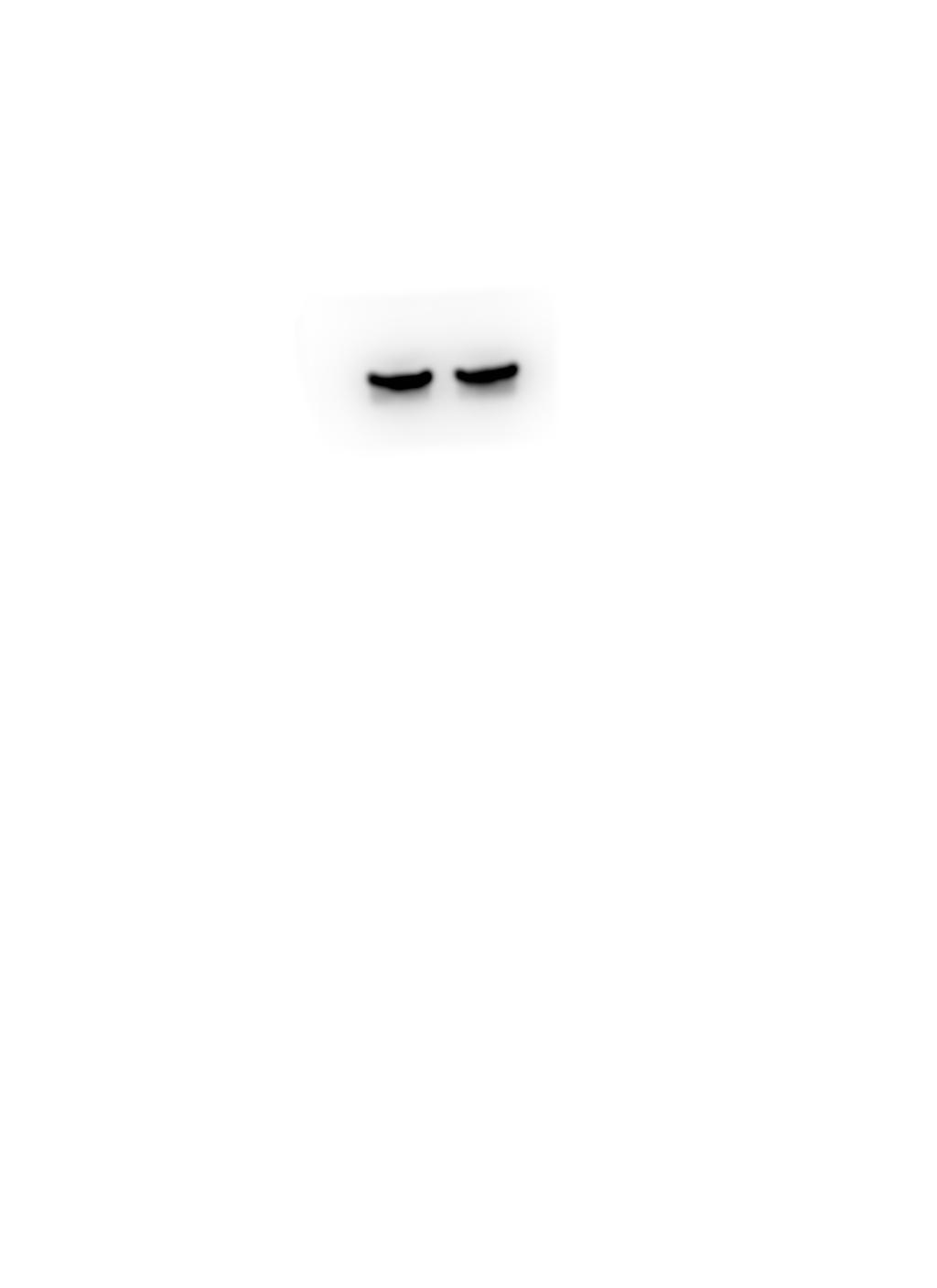

Supplement: Supplementary file 1 [file DataSheet_1.zip › Original data 1/Figure 5H/IP CDK1/CDK1/CDK1.jpg]

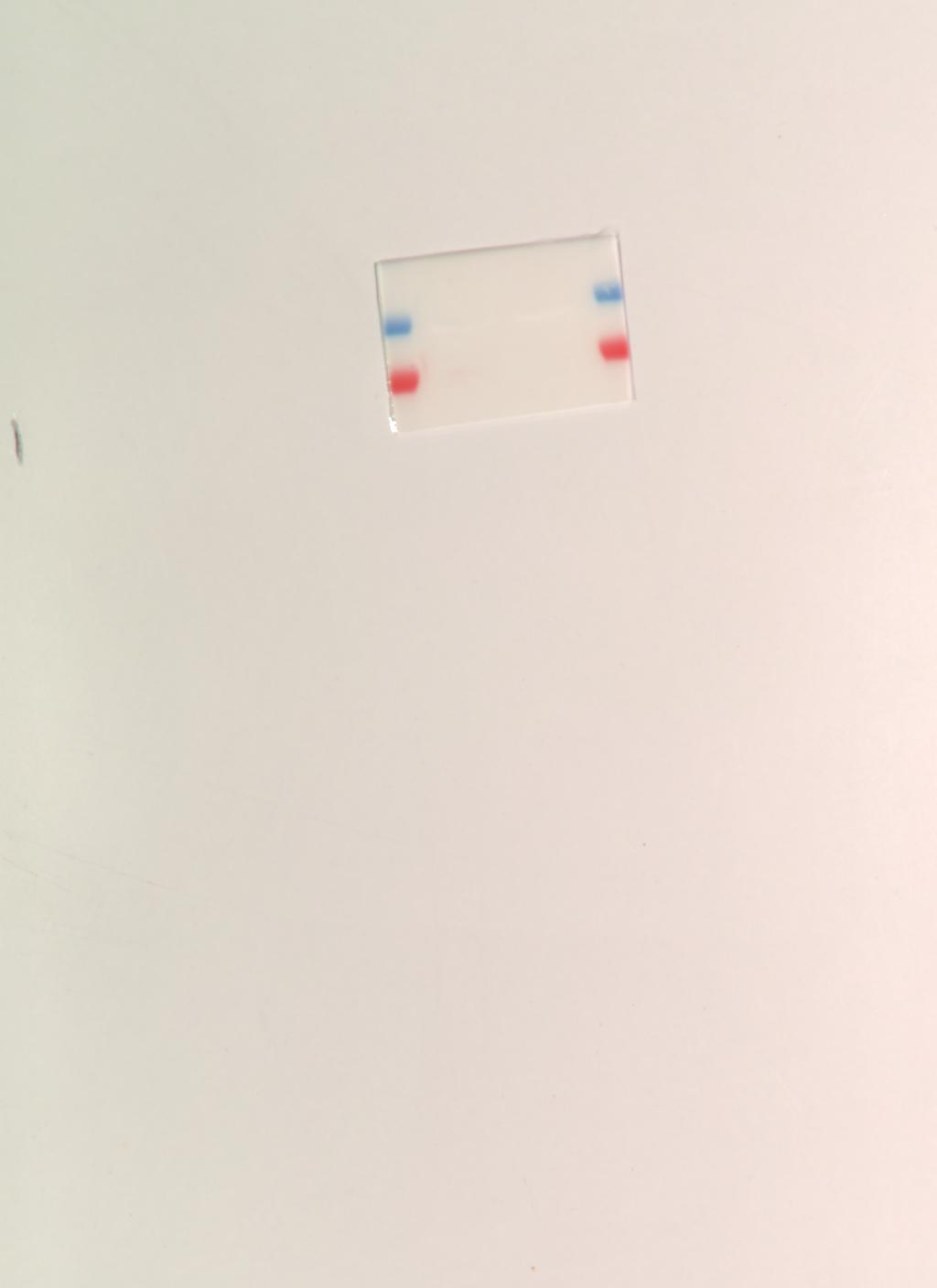

Supplement: Supplementary file 1 [file DataSheet_1.zip › Original data 1/Figure 5H/IP CDK1/SMURF1 M/SMURF1 M.jpg]

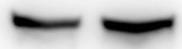

Supplement: Supplementary file 1 [file DataSheet_1.zip › Original data 1/Figure 5H/IP CDK1/SMURF1.jpg]

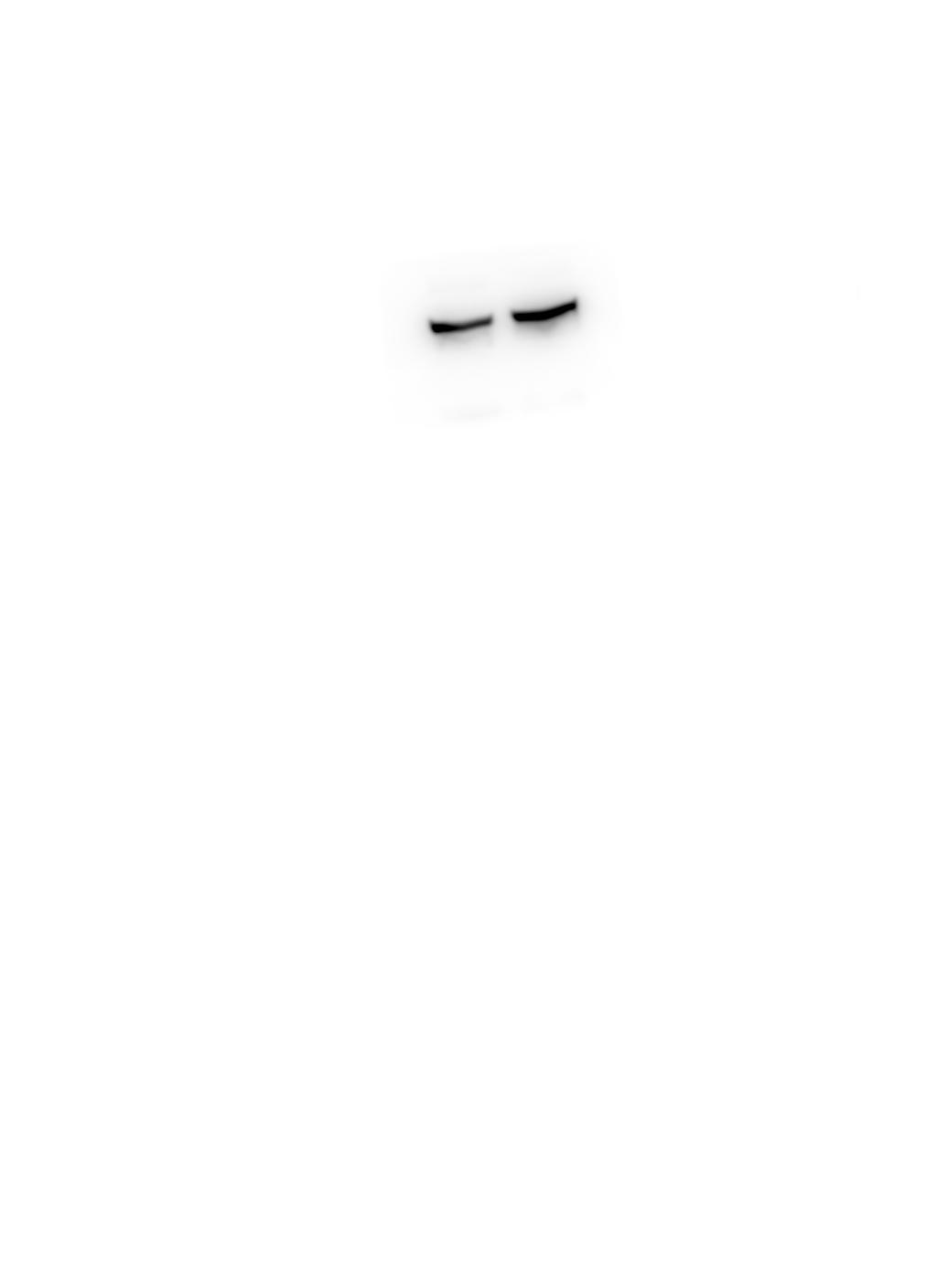

Supplement: Supplementary file 1 [file DataSheet_1.zip › Original data 1/Figure 5H/IP CDK1/SMURF1/SMURF1.jpg]

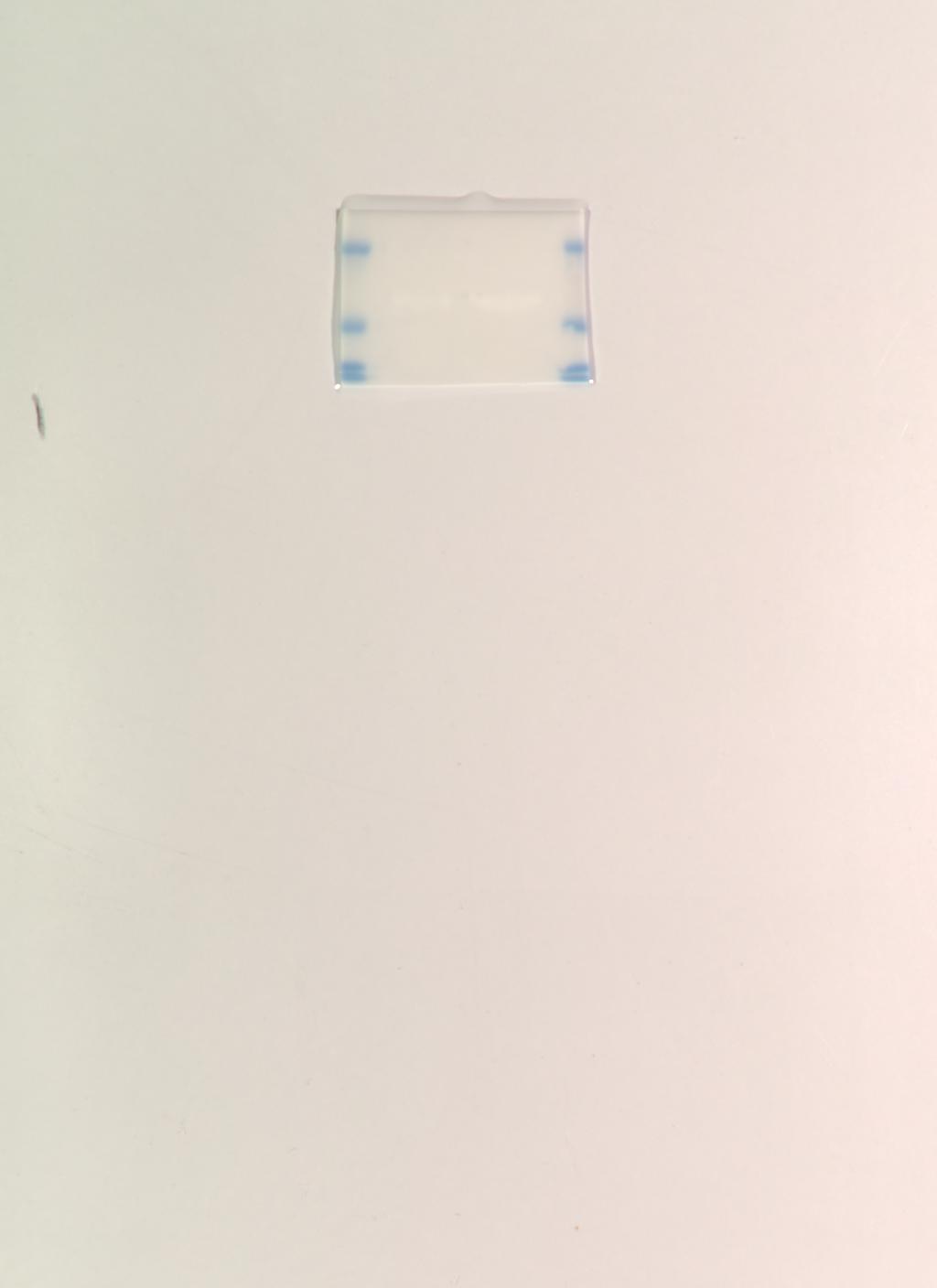

Supplement: Supplementary file 1 [file DataSheet_1.zip › Original data 1/Figure 5H/IP SMURF1/CDK1 M/CDK1 M.jpg]

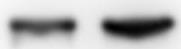

Supplement: Supplementary file 1 [file DataSheet_1.zip › Original data 1/Figure 5H/IP SMURF1/CDK1.jpg]

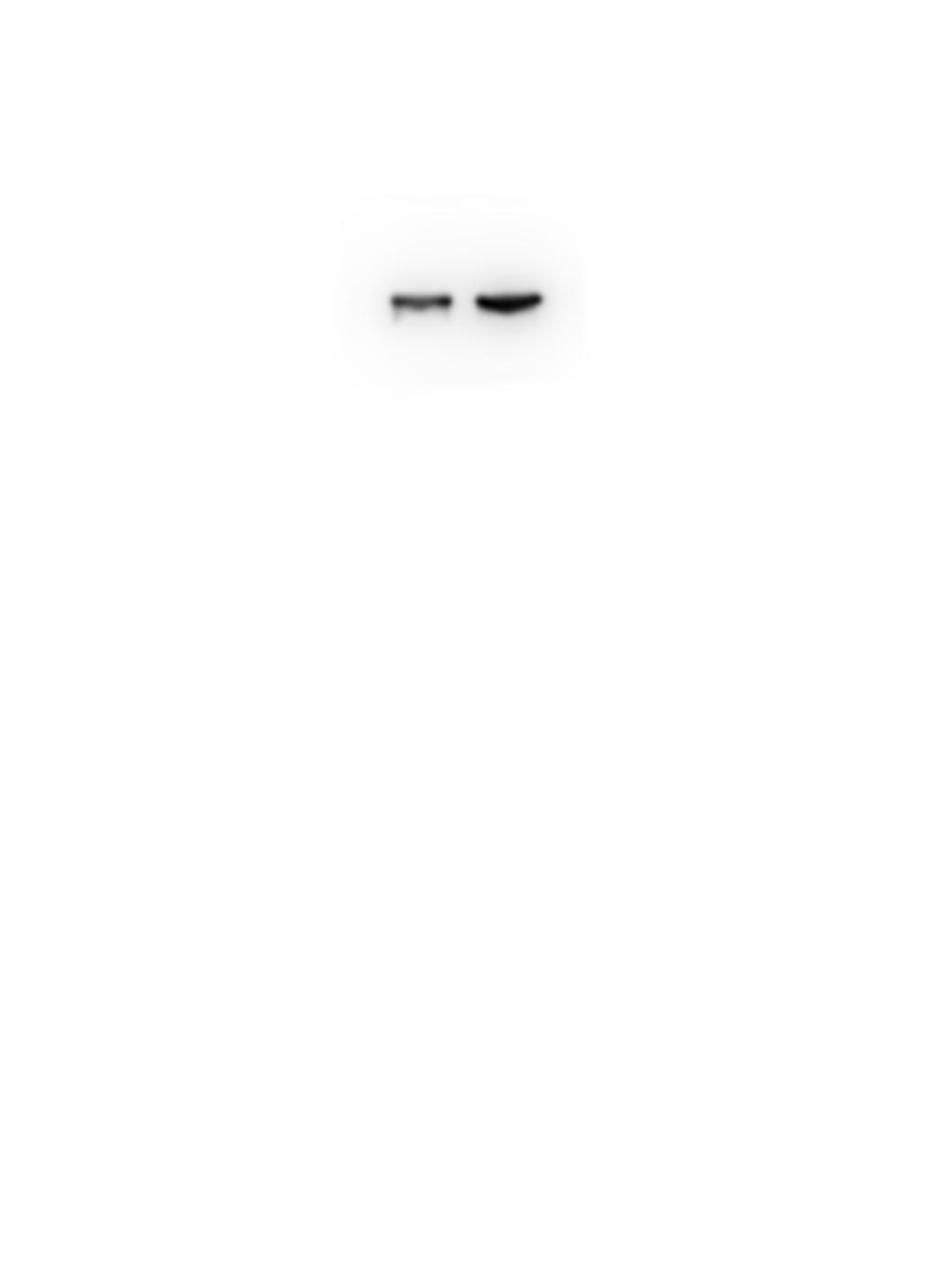

Supplement: Supplementary file 1 [file DataSheet_1.zip › Original data 1/Figure 5H/IP SMURF1/CDK1/CDK1.jpg]

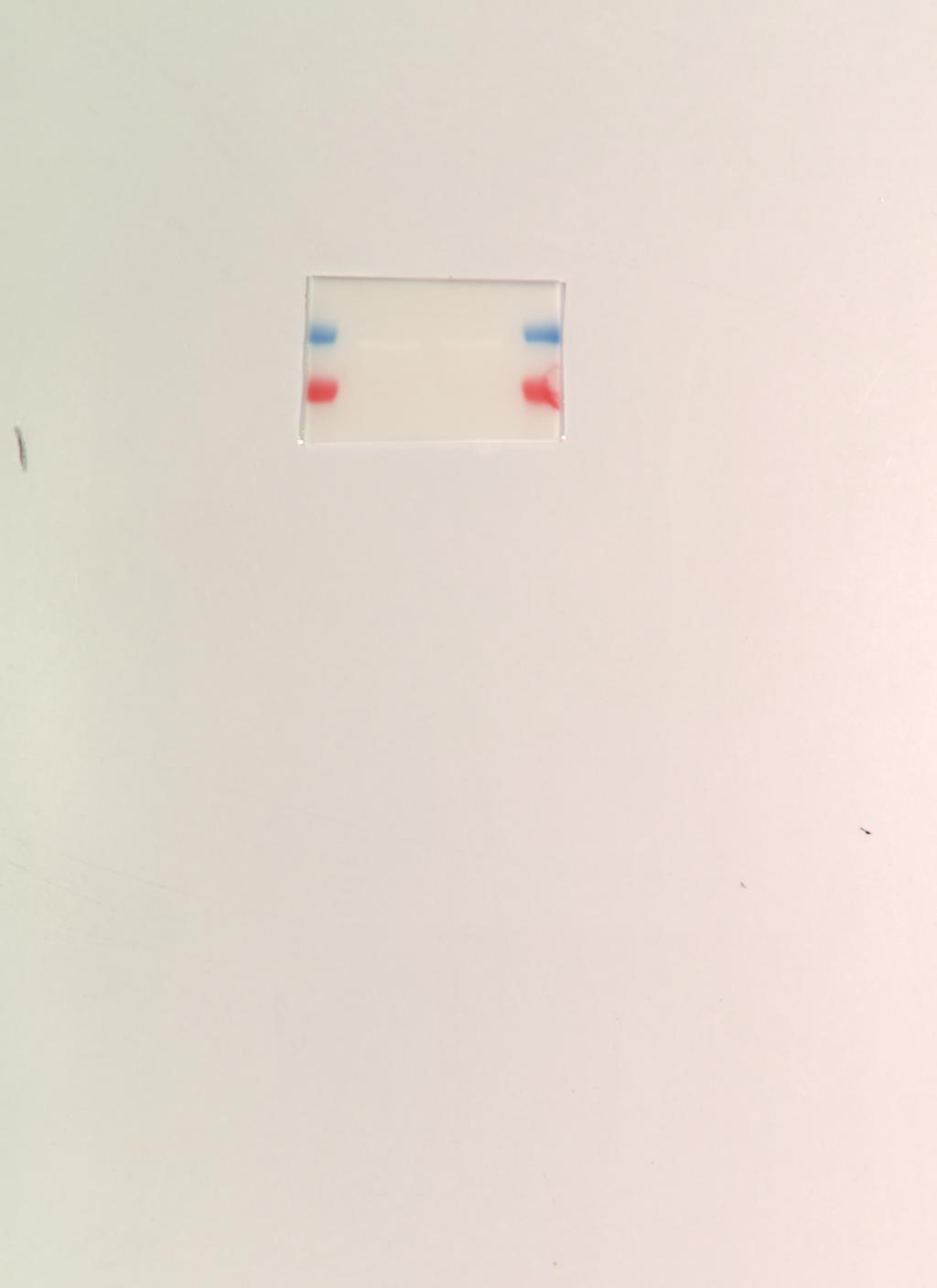

Supplement: Supplementary file 1 [file DataSheet_1.zip › Original data 1/Figure 5H/IP SMURF1/SMURF1 M/SMURF1 M.jpg]

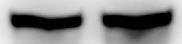

Supplement: Supplementary file 1 [file DataSheet_1.zip › Original data 1/Figure 5H/IP SMURF1/SMURF1.jpg]

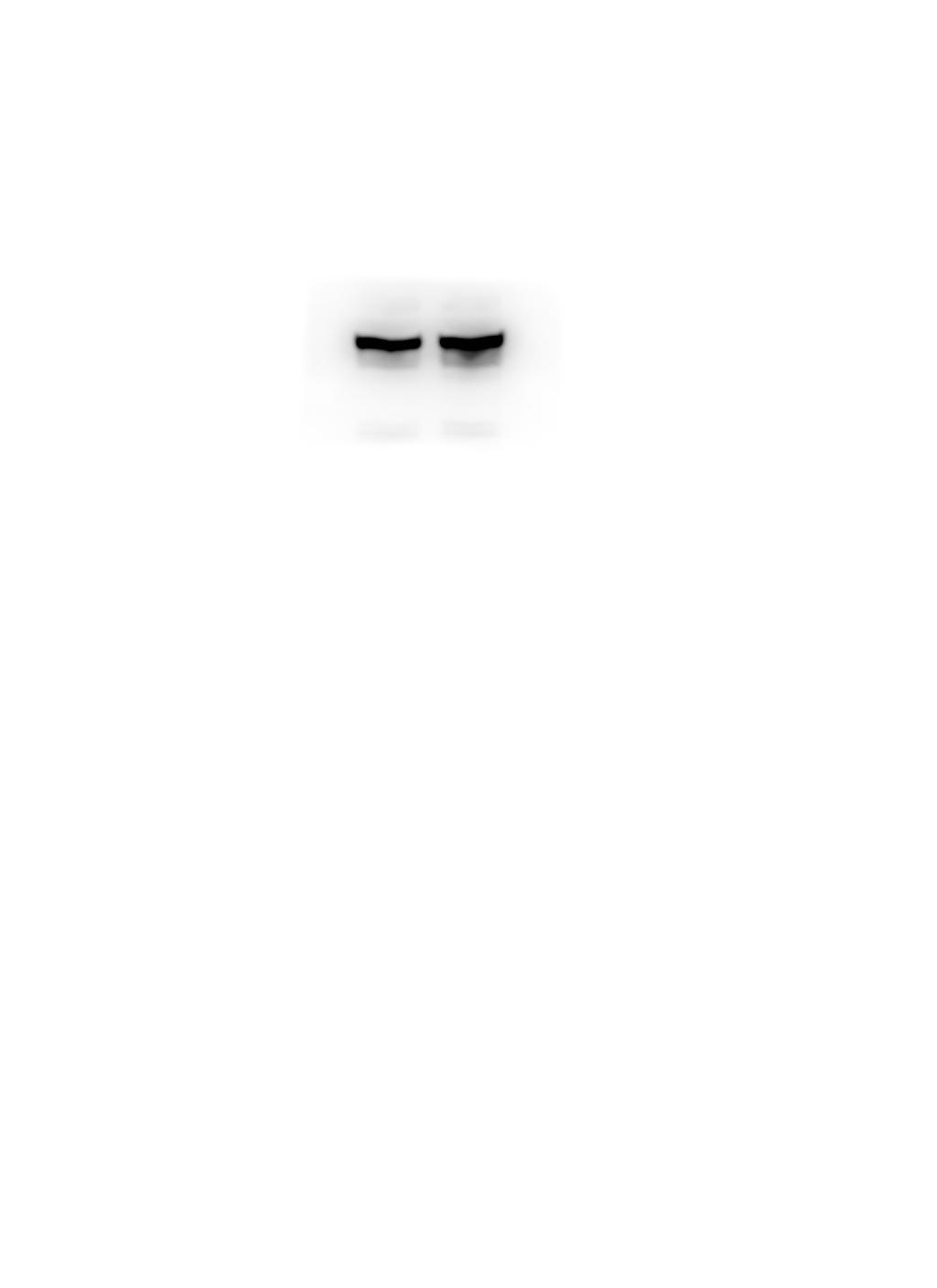

Supplement: Supplementary file 1 [file DataSheet_1.zip › Original data 1/Figure 5H/IP SMURF1/SMURF1/SMURF1.jpg]

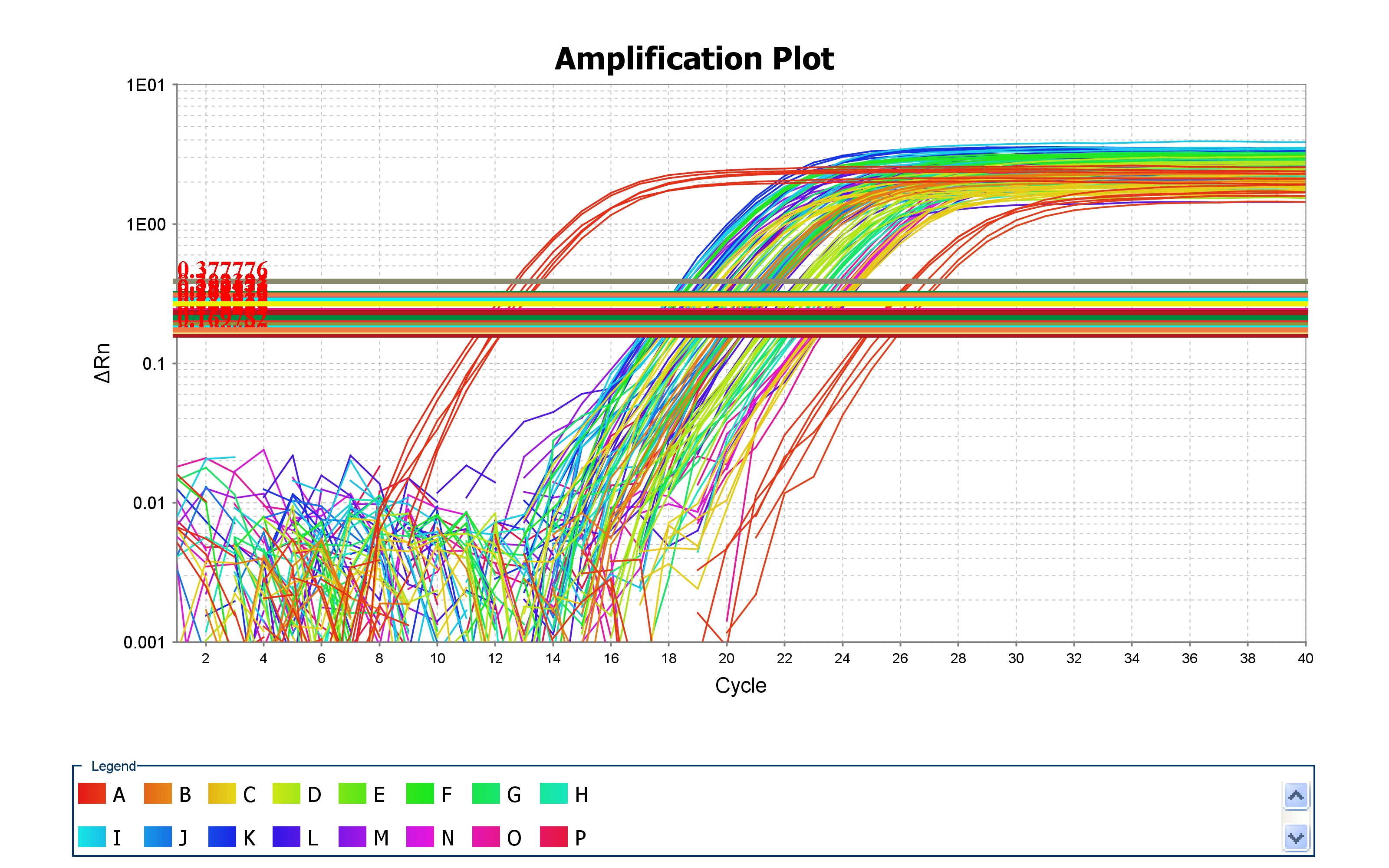

Supplement: Supplementary file 1 [file DataSheet_1.zip › Original data 1/Figure S2B/Amplification Plot.jpg]

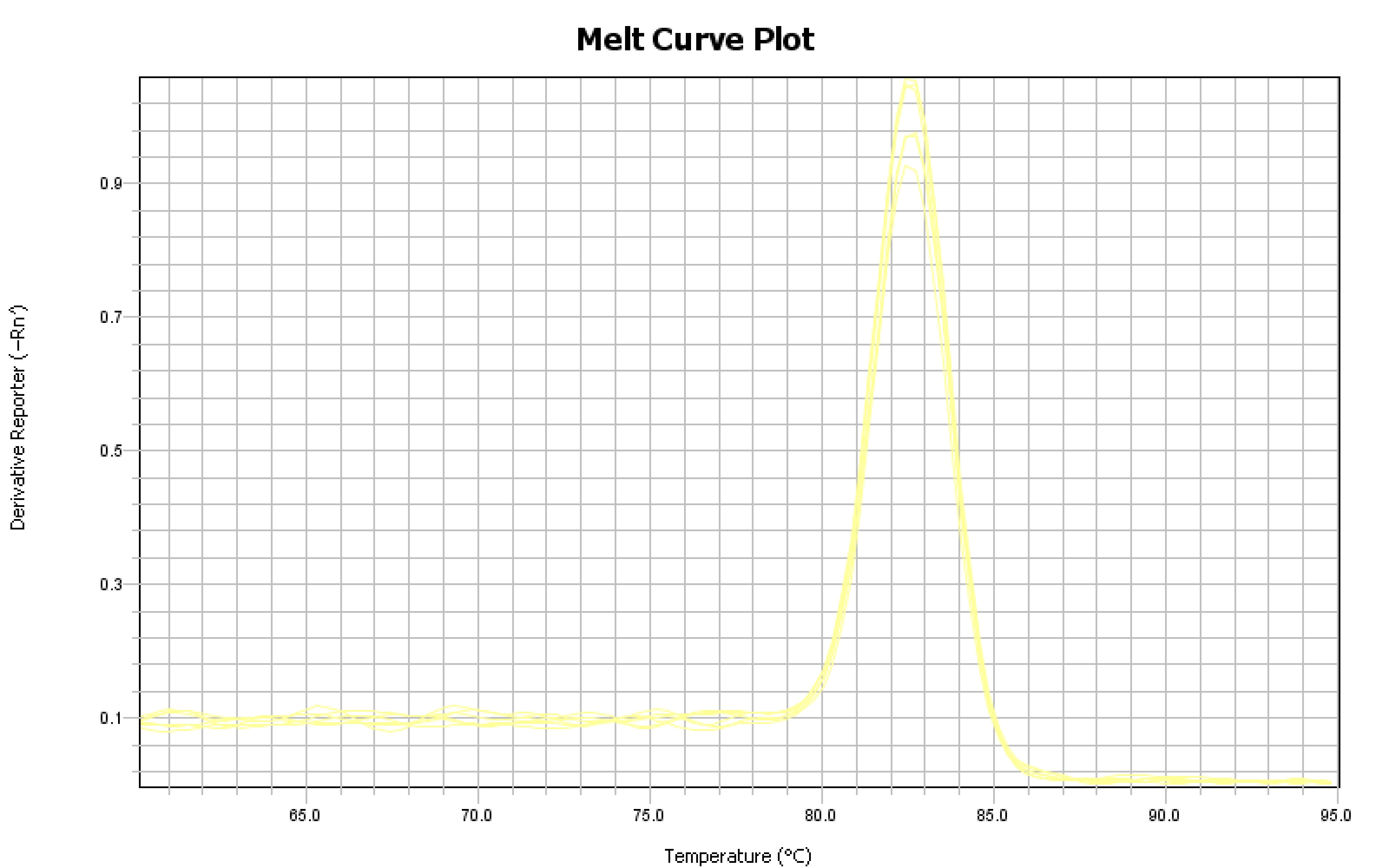

Supplement: Supplementary file 1 [file DataSheet_1.zip › Original data 1/Figure S2B/Melt Curve Plot H-AKT2.jpg]

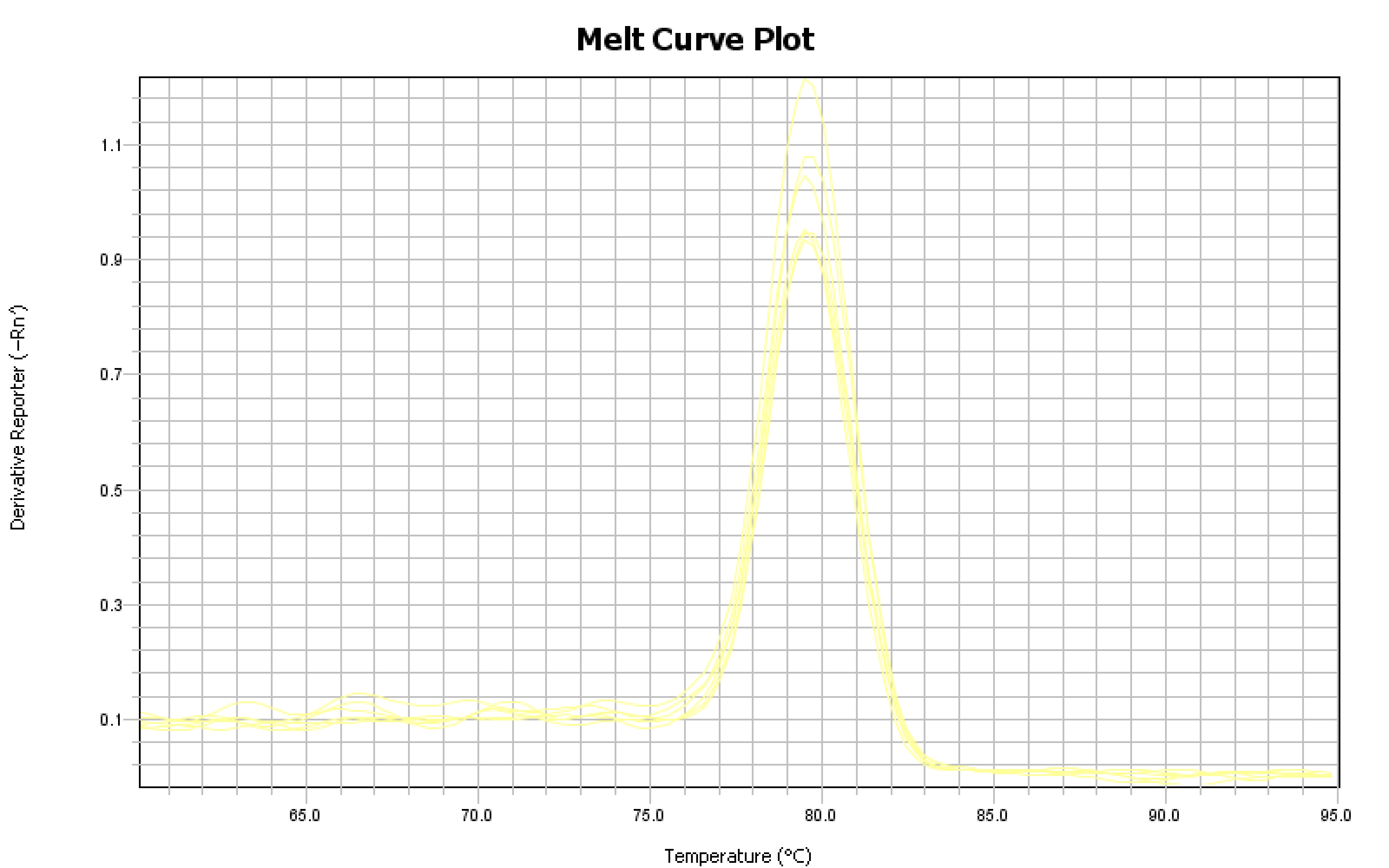

Supplement: Supplementary file 1 [file DataSheet_1.zip › Original data 1/Figure S2B/Melt Curve Plot H-BTRC.jpg]

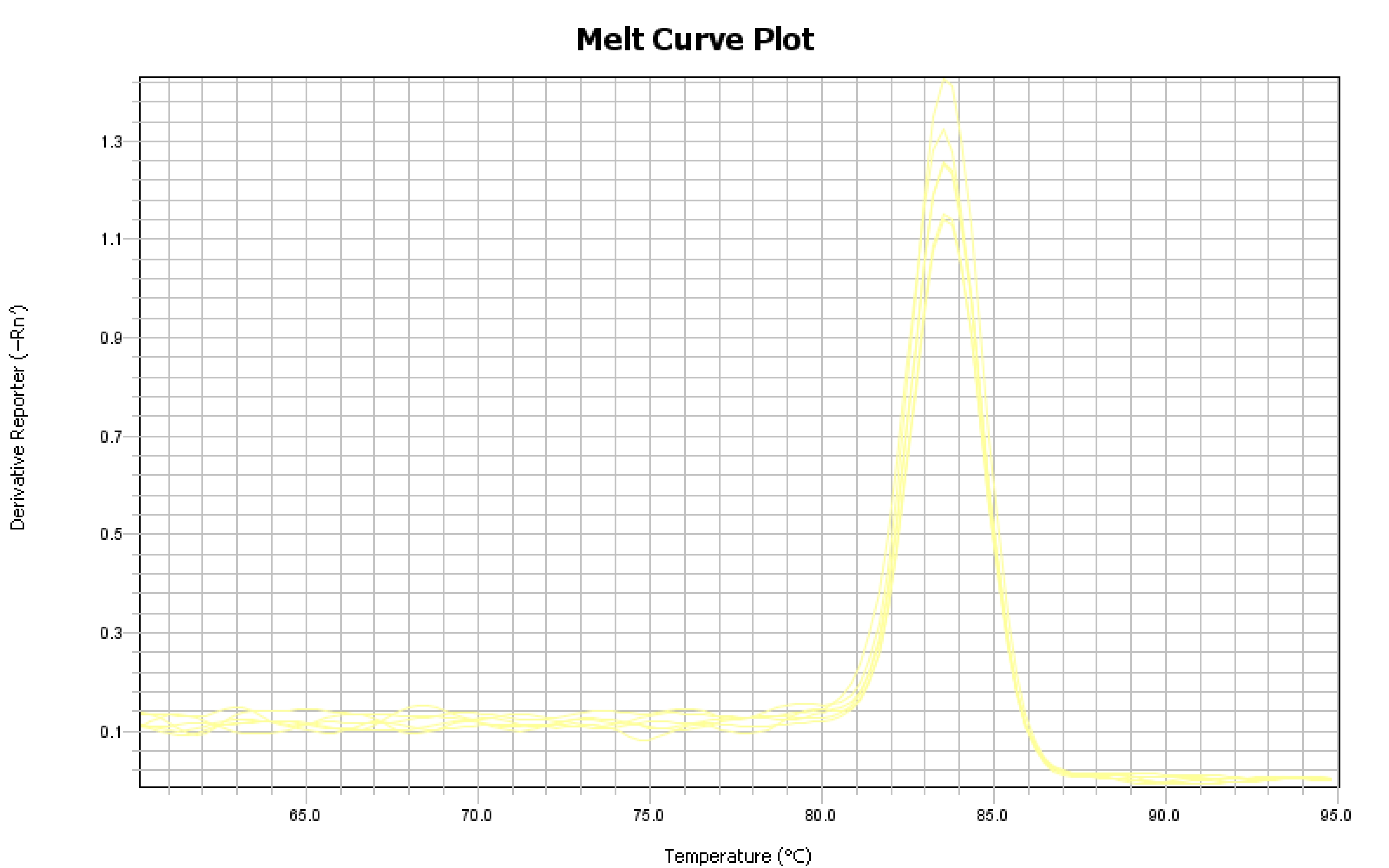

Supplement: Supplementary file 1 [file DataSheet_1.zip › Original data 1/Figure S2B/Melt Curve Plot H-CBY1.jpg]

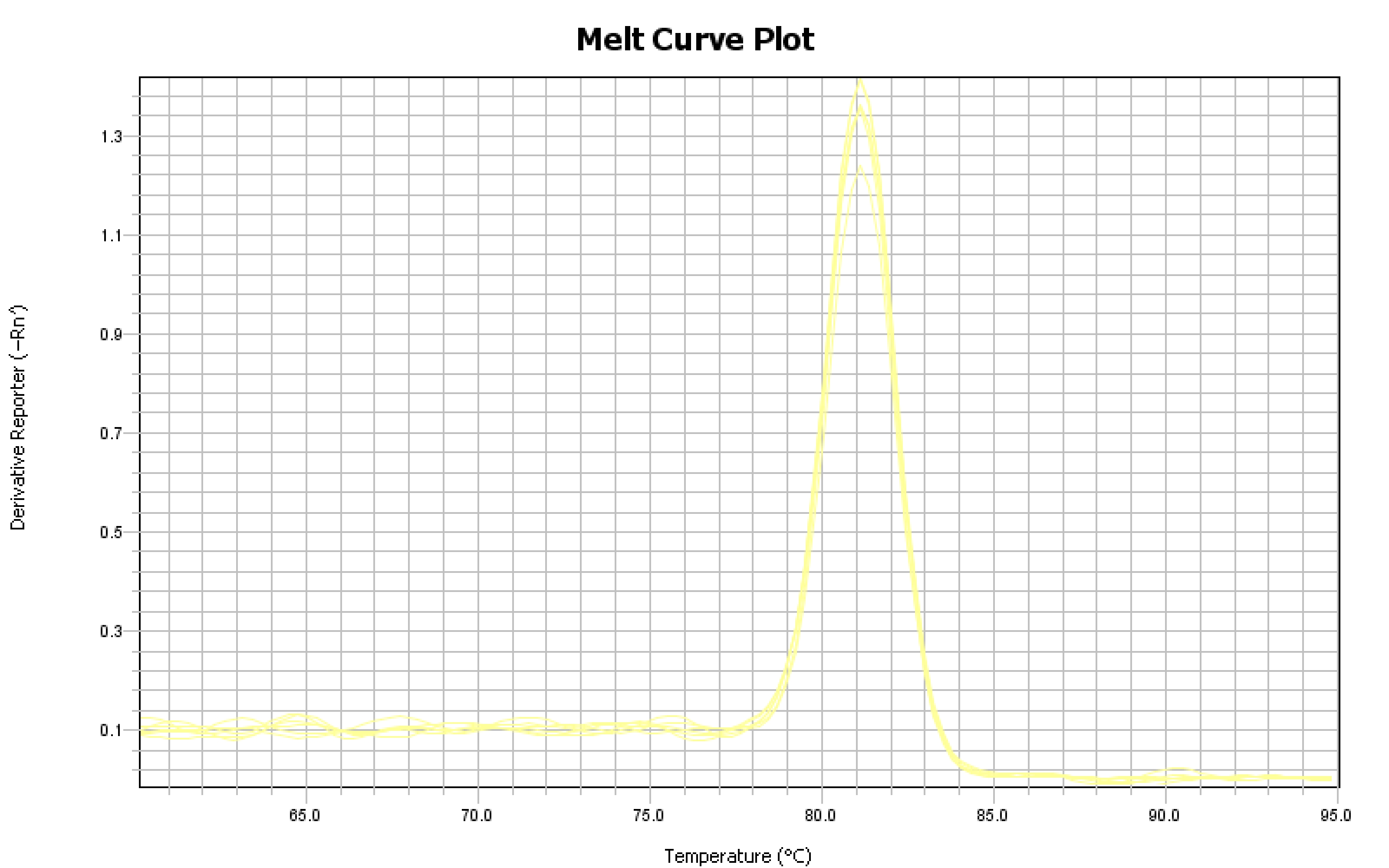

Supplement: Supplementary file 1 [file DataSheet_1.zip › Original data 1/Figure S2B/Melt Curve Plot H-CCNB2.jpg]

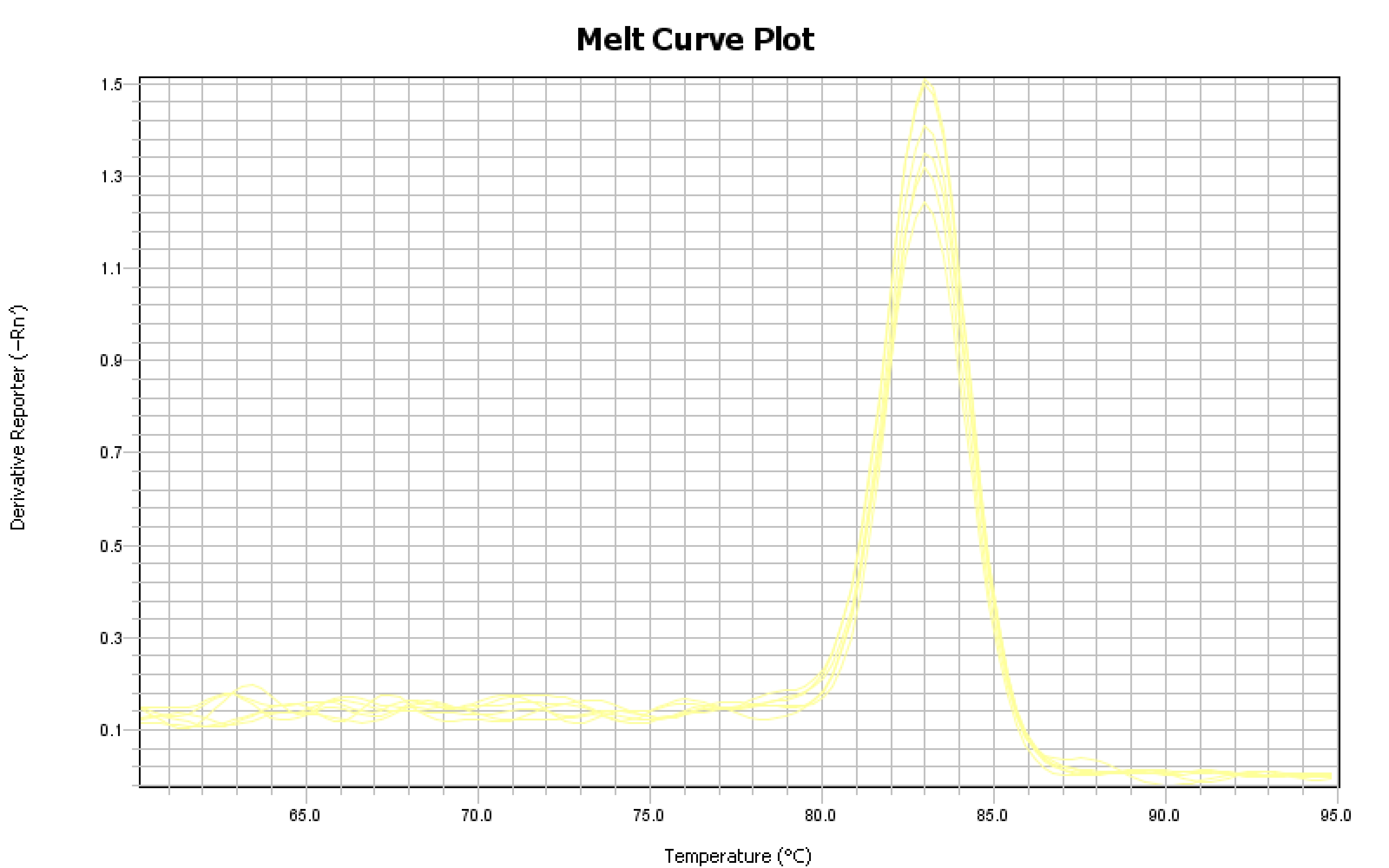

Supplement: Supplementary file 1 [file DataSheet_1.zip › Original data 1/Figure S2B/Melt Curve Plot H-CCNE2.jpg]

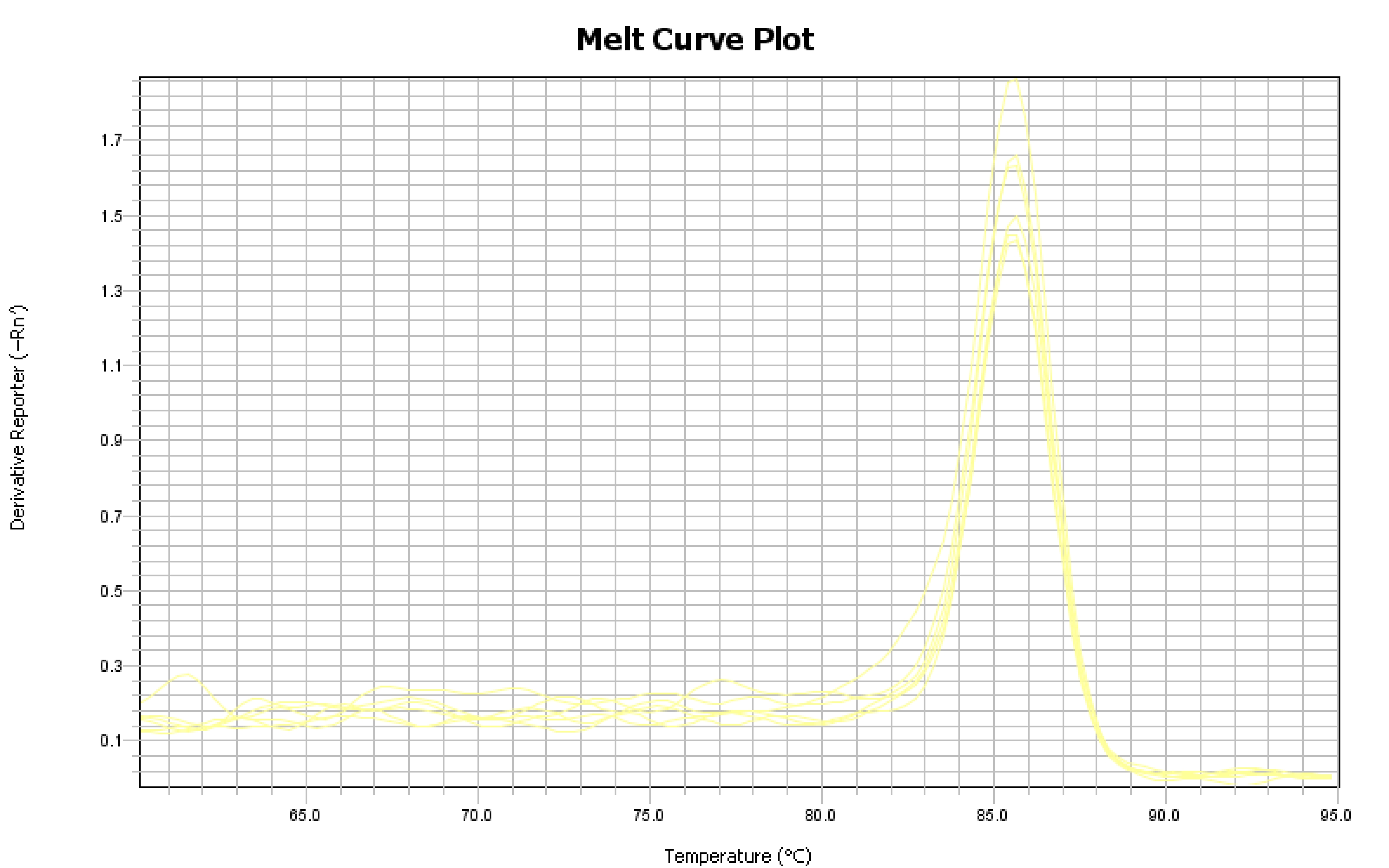

Supplement: Supplementary file 1 [file DataSheet_1.zip › Original data 1/Figure S2B/Melt Curve Plot H-CDC25B.jpg]

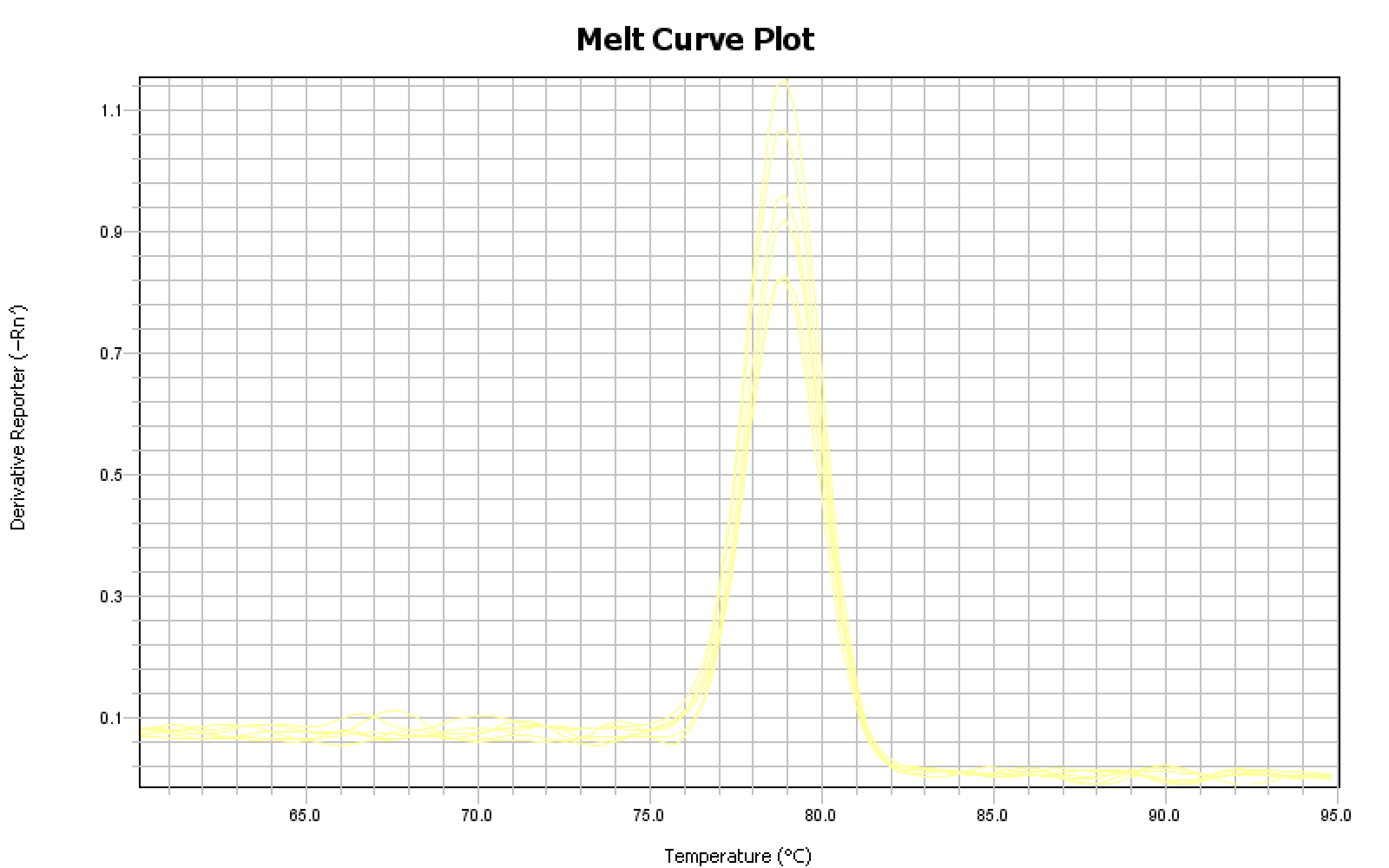

Supplement: Supplementary file 1 [file DataSheet_1.zip › Original data 1/Figure S2B/Melt Curve Plot H-CDC42.jpg]

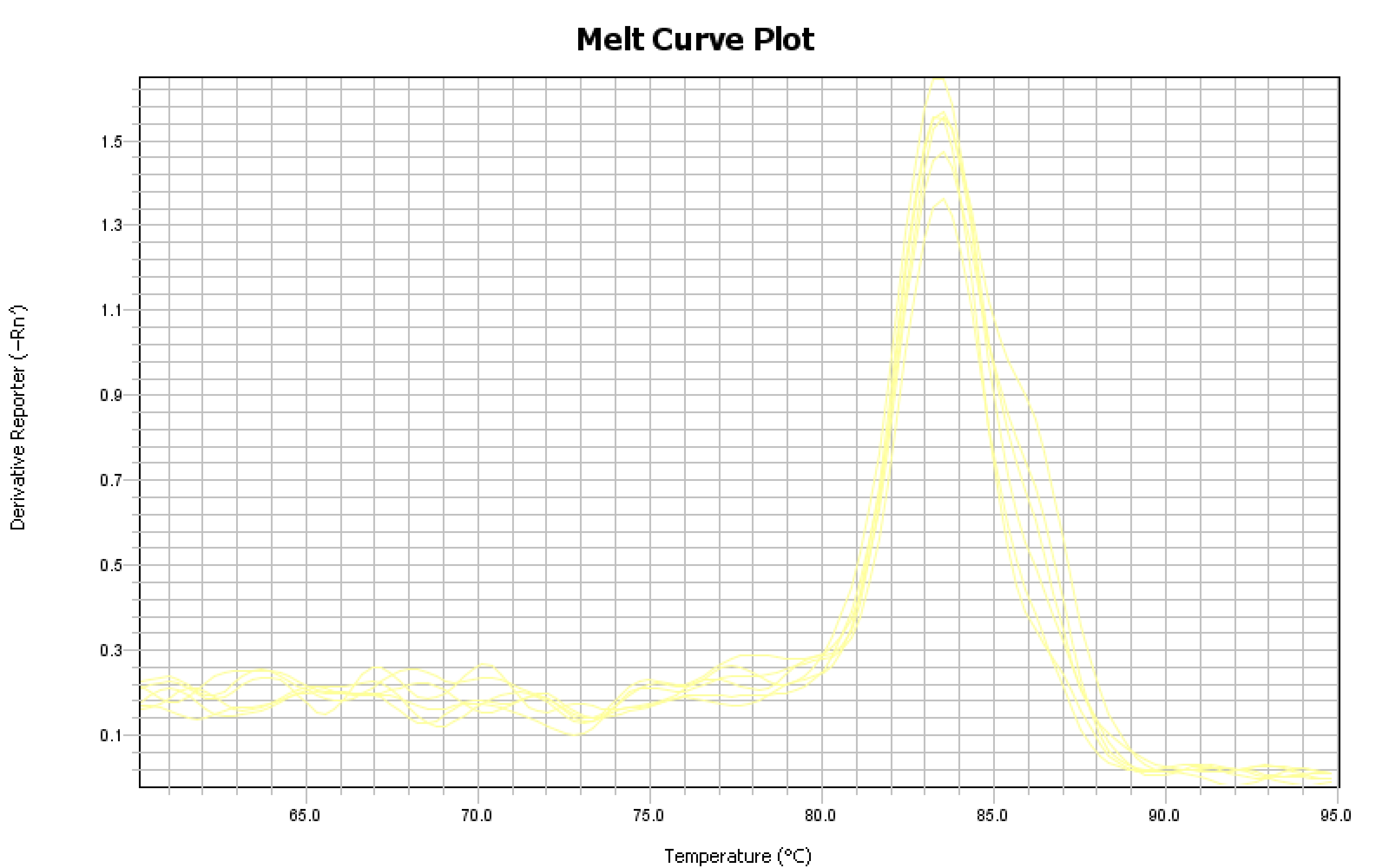

Supplement: Supplementary file 1 [file DataSheet_1.zip › Original data 1/Figure S2B/Melt Curve Plot H-CDK16.jpg]

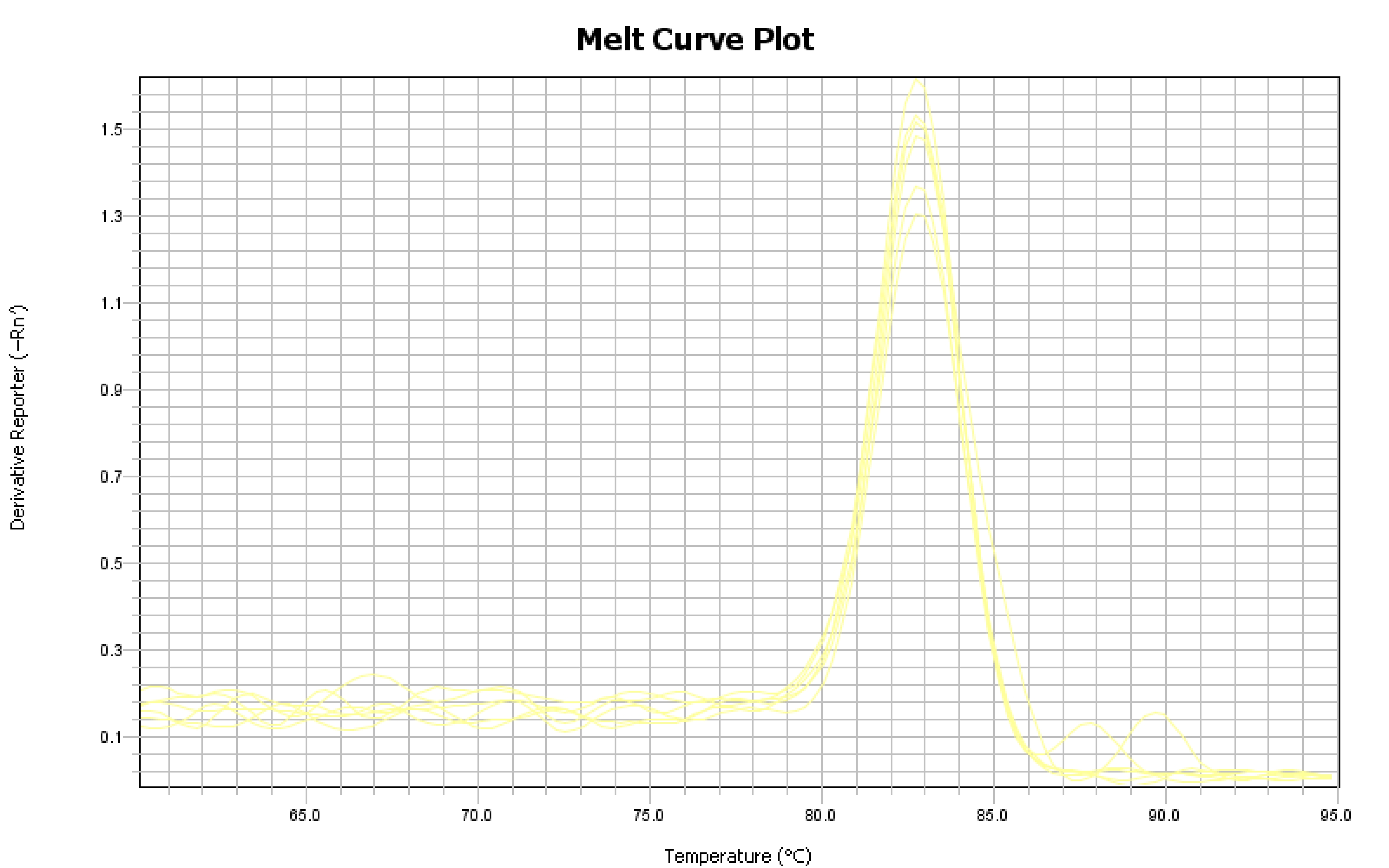

Supplement: Supplementary file 1 [file DataSheet_1.zip › Original data 1/Figure S2B/Melt Curve Plot H-CDK2.jpg]

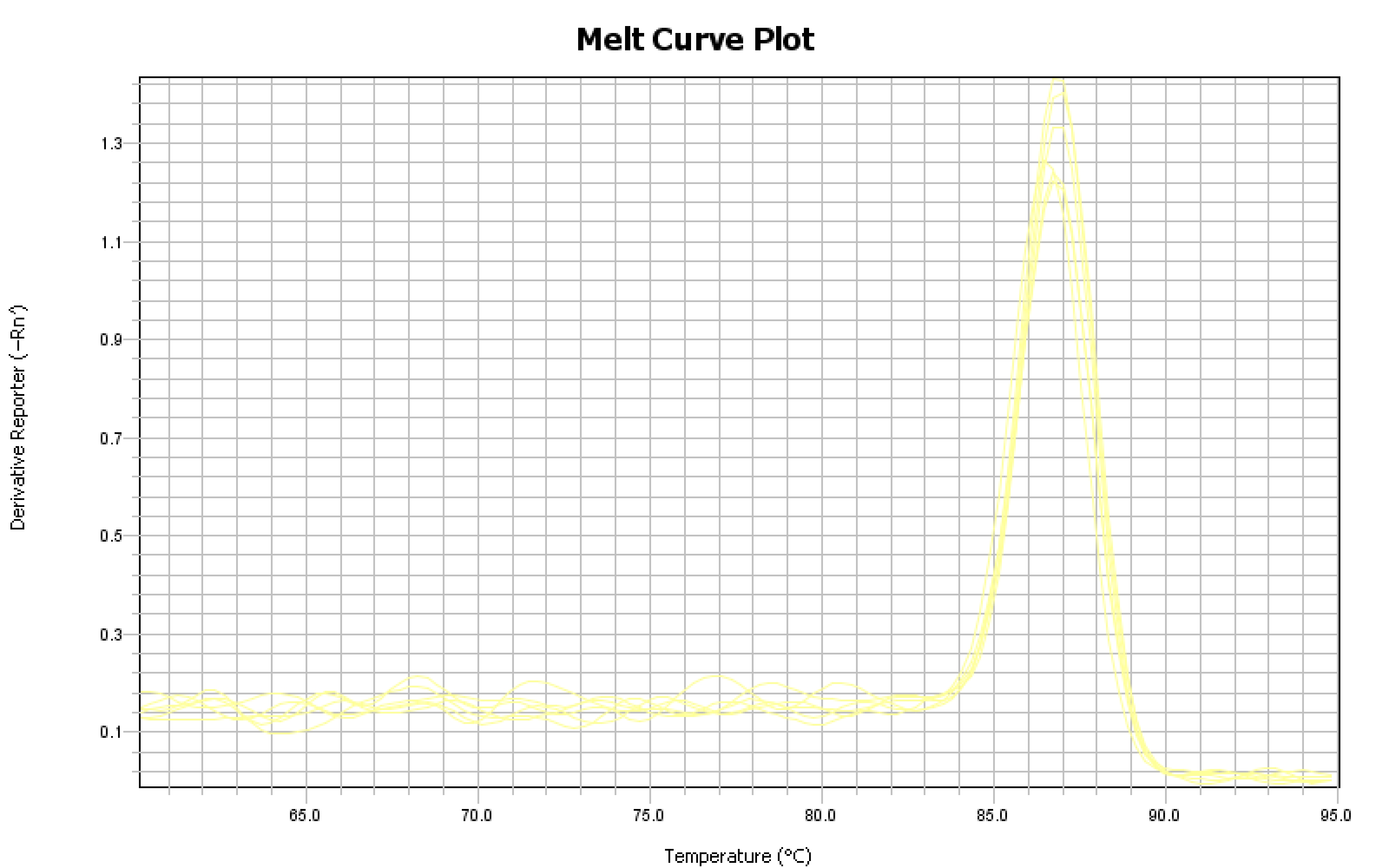

Supplement: Supplementary file 1 [file DataSheet_1.zip › Original data 1/Figure S2B/Melt Curve Plot H-E2F1.jpg]

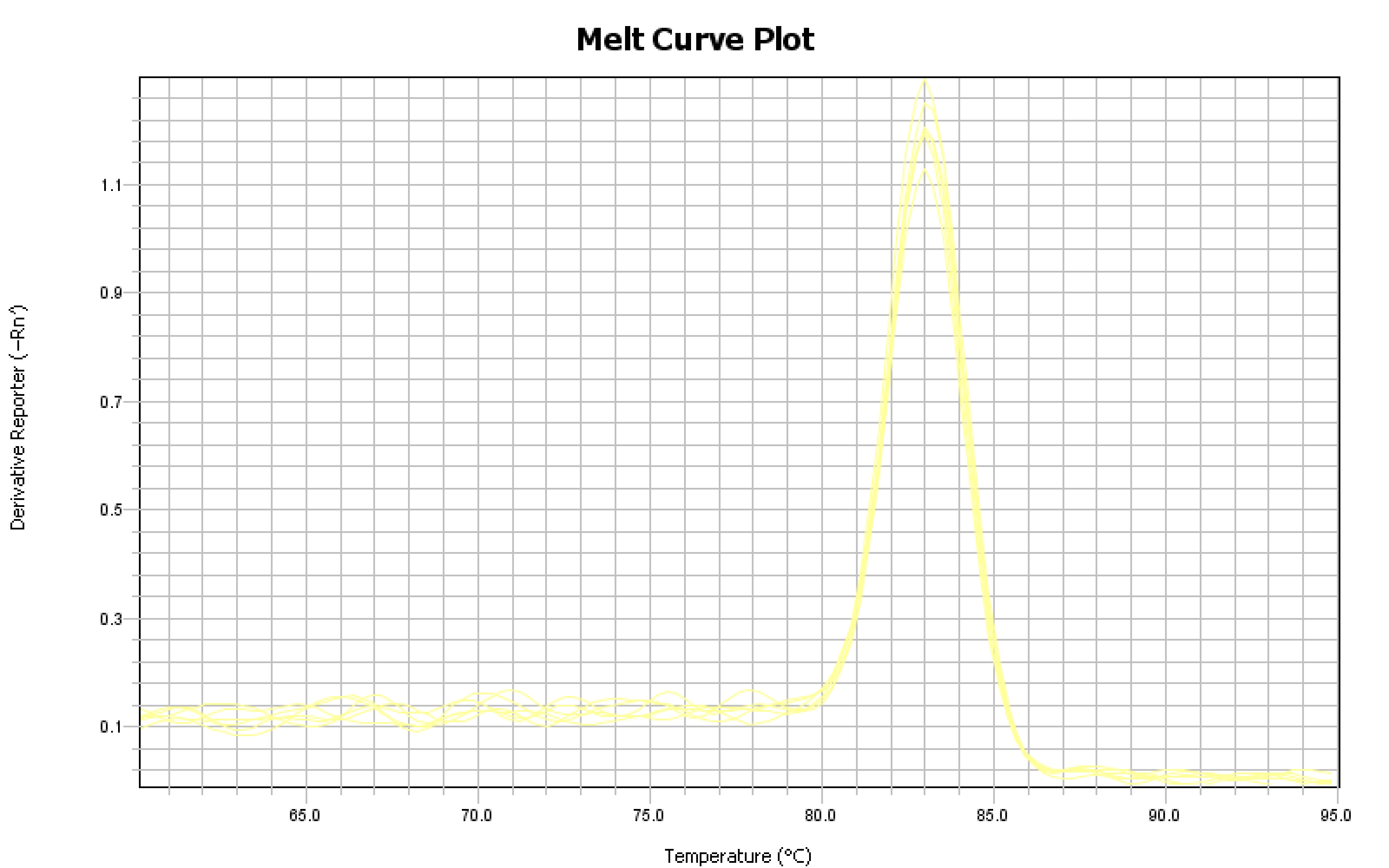

Supplement: Supplementary file 1 [file DataSheet_1.zip › Original data 1/Figure S2B/Melt Curve Plot H-FAM53C.jpg]

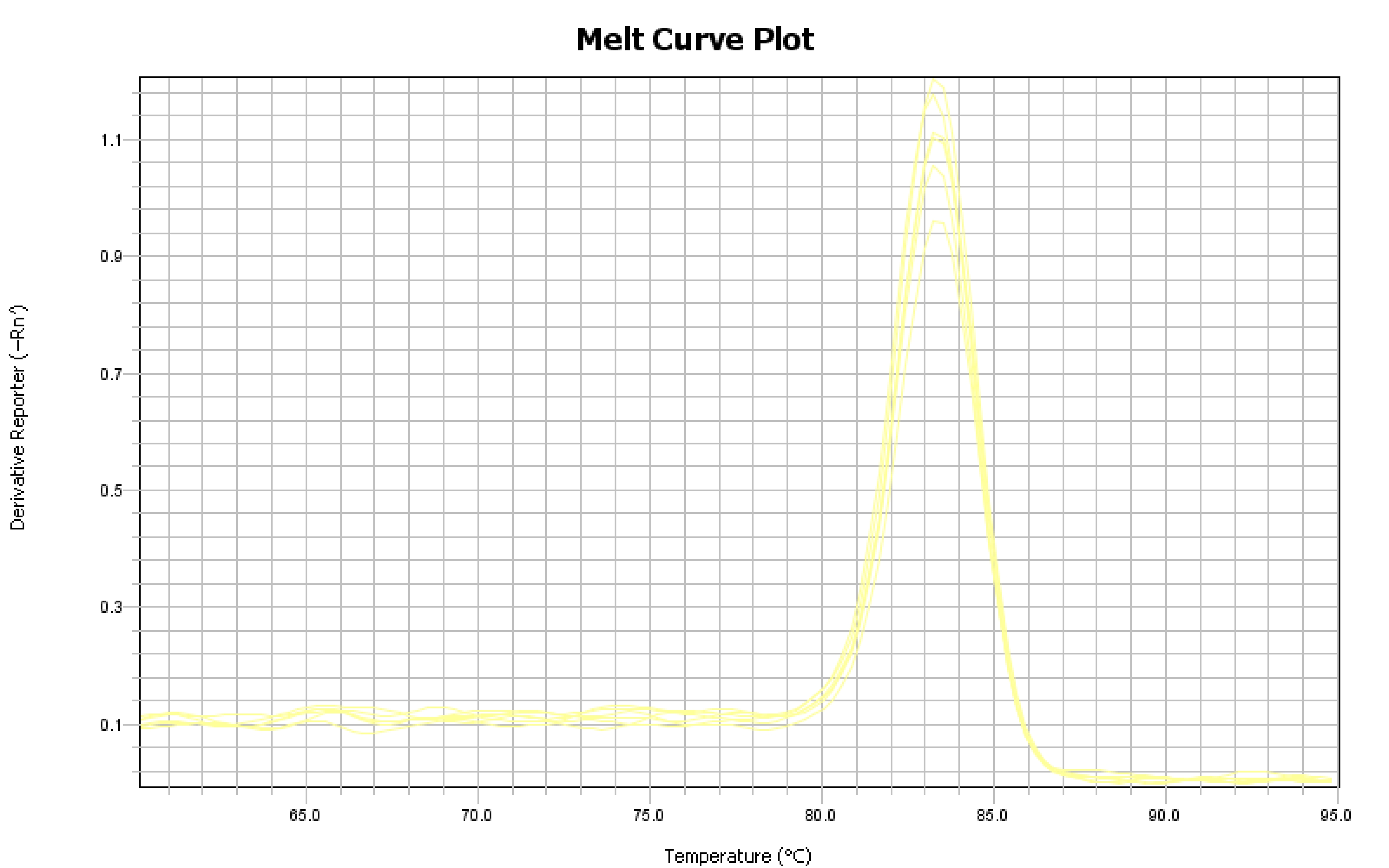

Supplement: Supplementary file 1 [file DataSheet_1.zip › Original data 1/Figure S2B/Melt Curve Plot H-GAPDH.jpg]

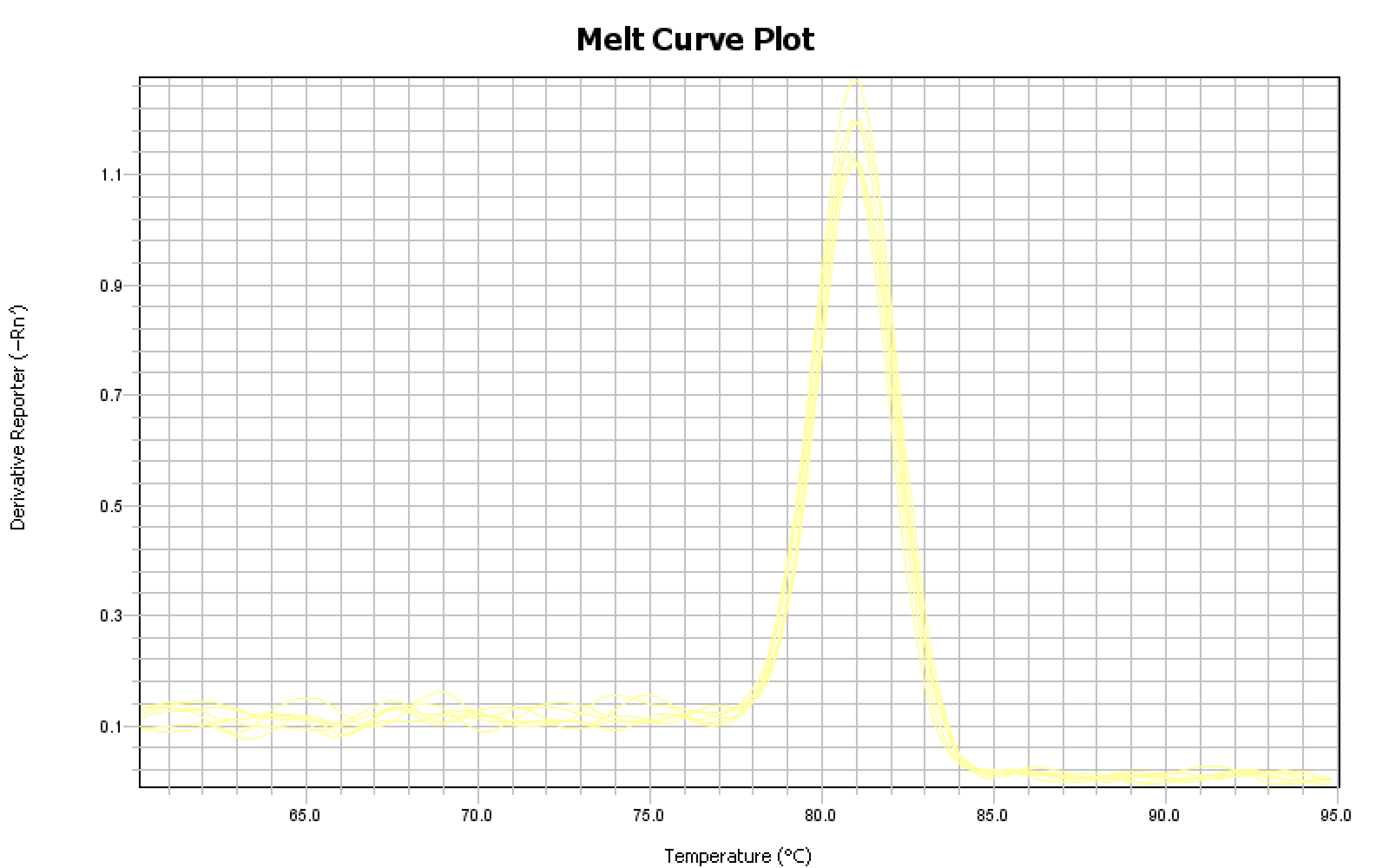

Supplement: Supplementary file 1 [file DataSheet_1.zip › Original data 1/Figure S2B/Melt Curve Plot H-KCTD3.jpg]

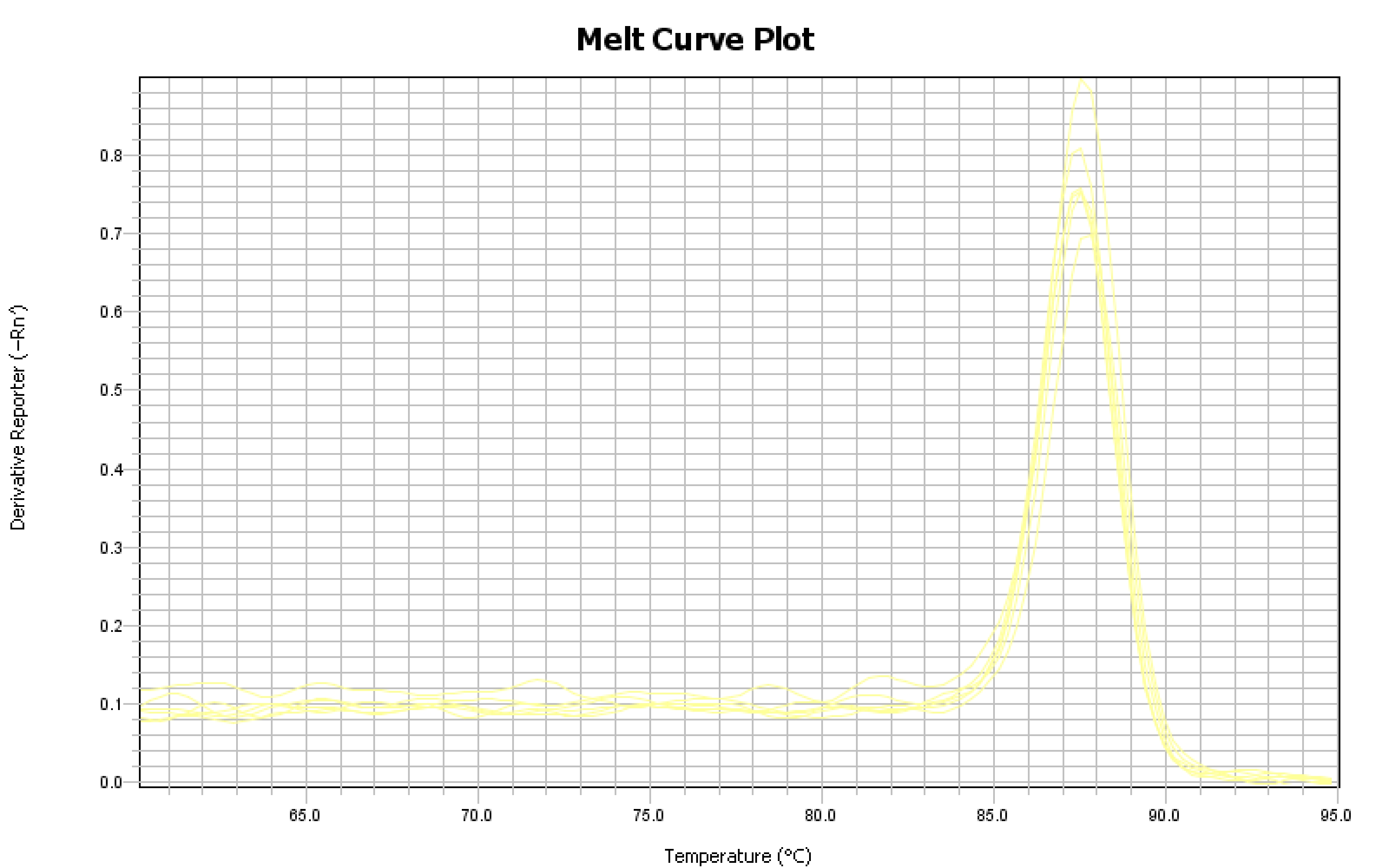

Supplement: Supplementary file 1 [file DataSheet_1.zip › Original data 1/Figure S2B/Melt Curve Plot H-KIF13B.jpg]

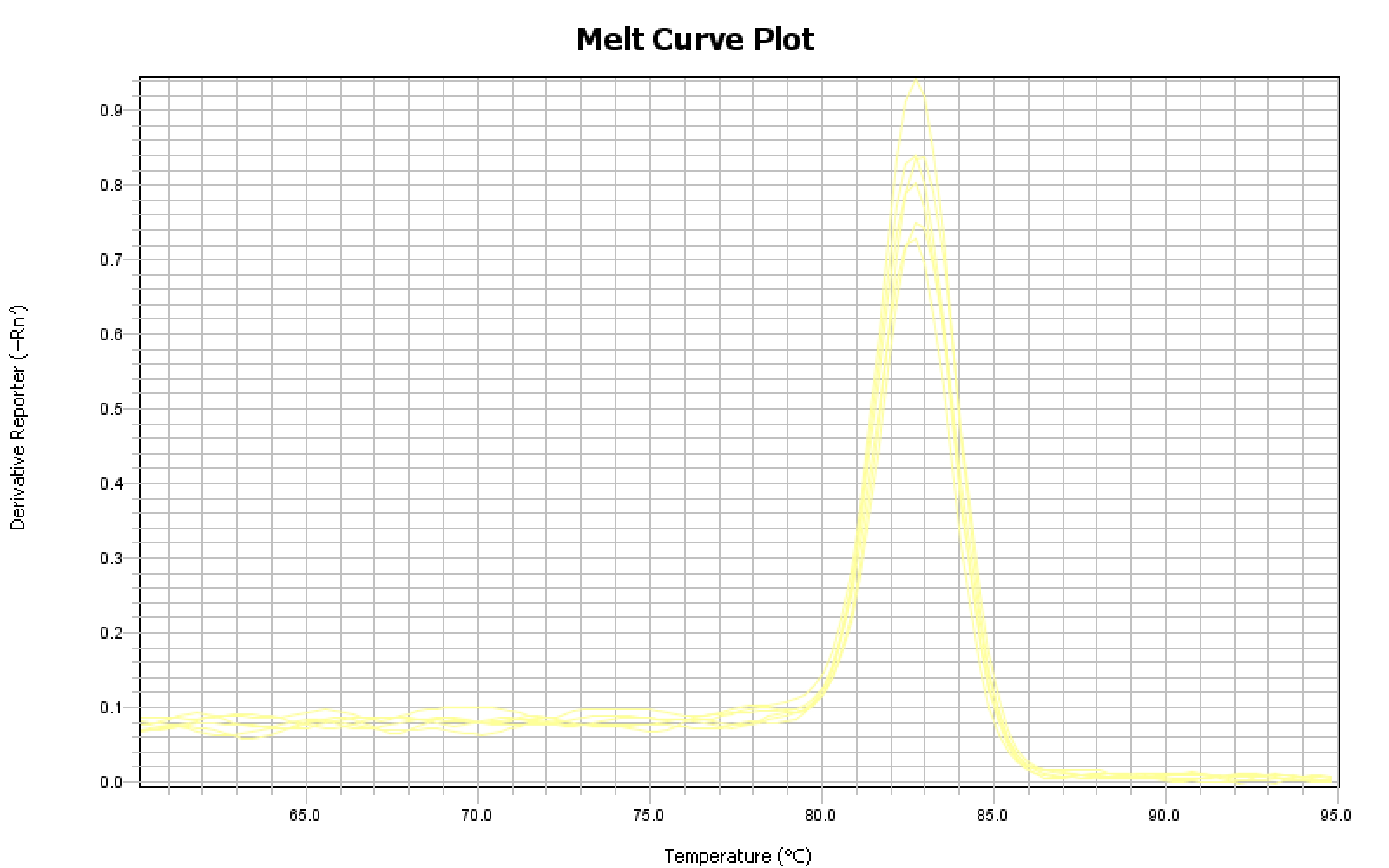

Supplement: Supplementary file 1 [file DataSheet_1.zip › Original data 1/Figure S2B/Melt Curve Plot H-LIMA1.jpg]

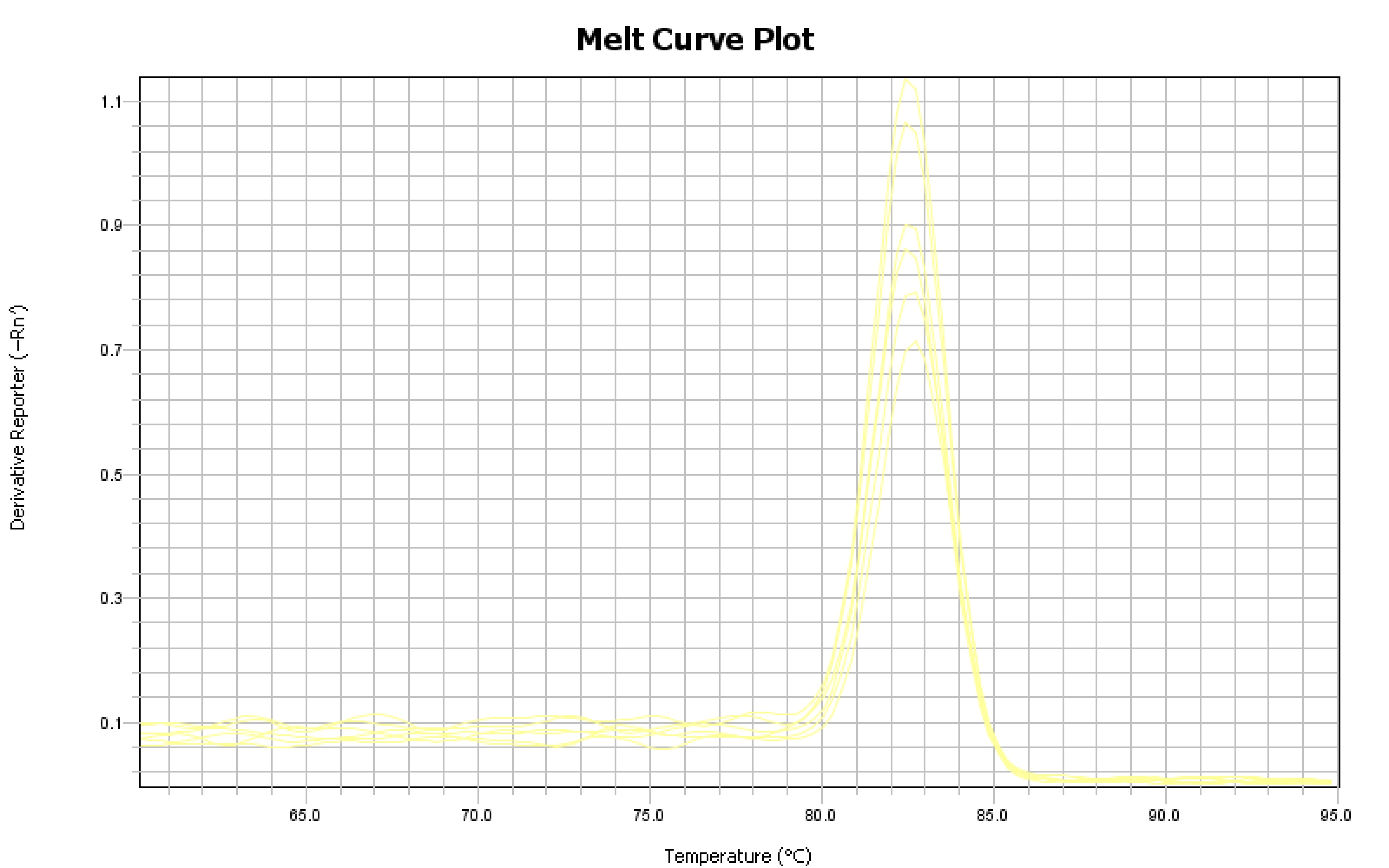

Supplement: Supplementary file 1 [file DataSheet_1.zip › Original data 1/Figure S2B/Melt Curve Plot H-MAPK1.jpg]

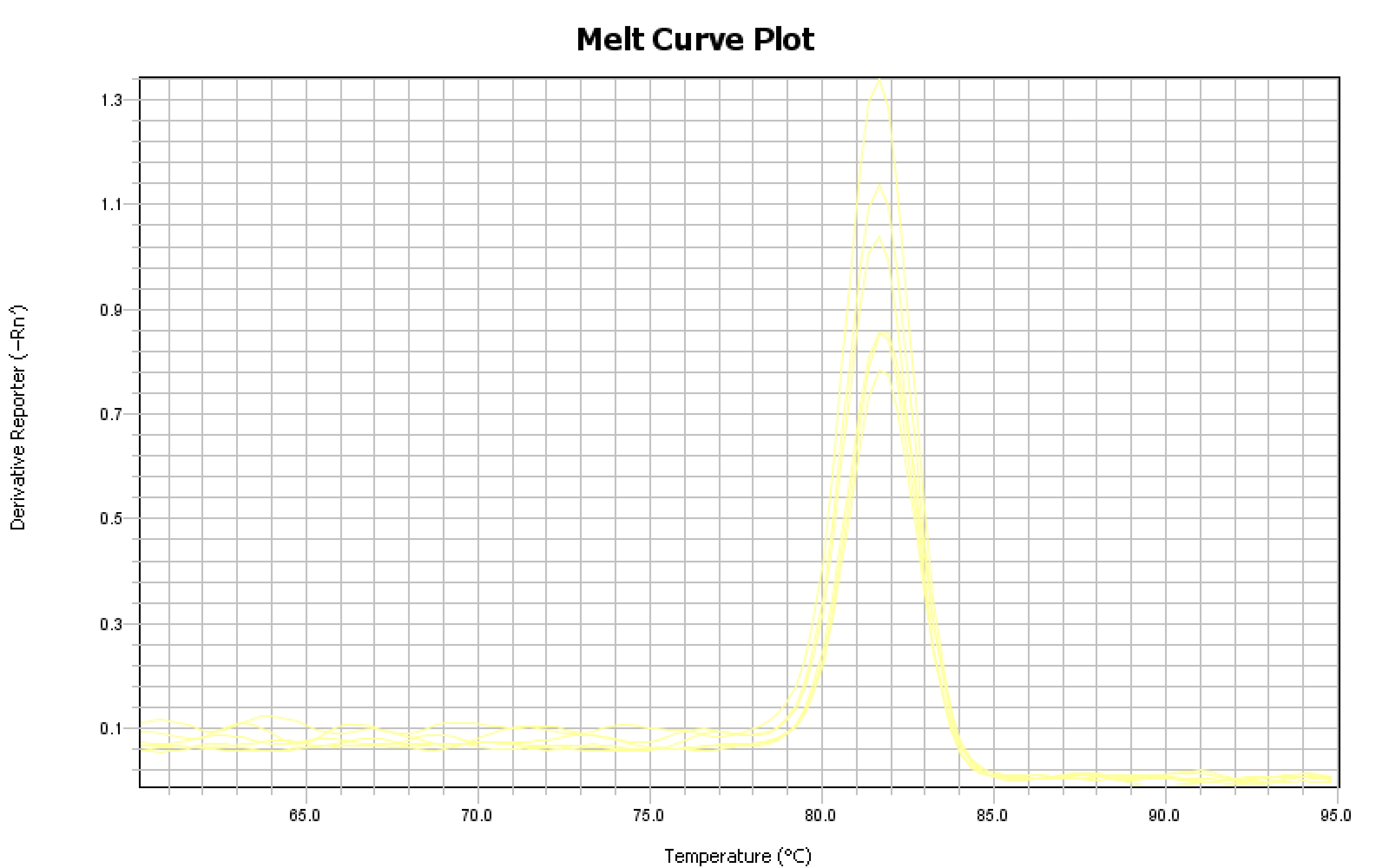

Supplement: Supplementary file 1 [file DataSheet_1.zip › Original data 1/Figure S2B/Melt Curve Plot H-NEK2.jpg]

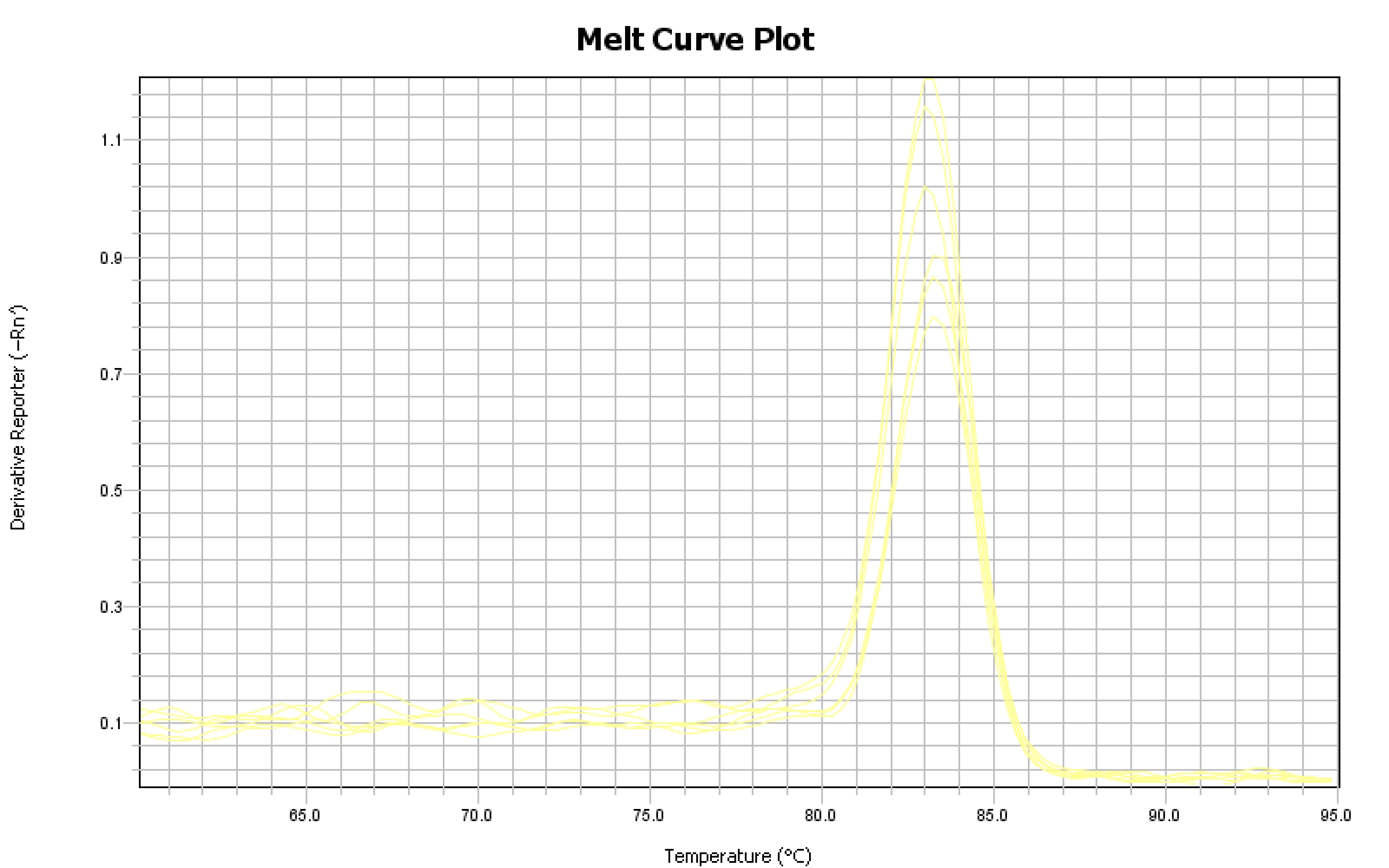

Supplement: Supplementary file 1 [file DataSheet_1.zip › Original data 1/Figure S2B/Melt Curve Plot H-PLK1.jpg]
