# Supplementary figures and images for: DEPDC1B promotes development of cholangiocarcinoma through enhancing the stability of CDK1 and regulating malignant phenotypes (part 1 of 2)
Source: Front Oncol. 2022 Dec 6;12:842205. doi: 10.3389/fonc.2022.842205 (PMC9769124; doi:10.3389/fonc.2022.842205)

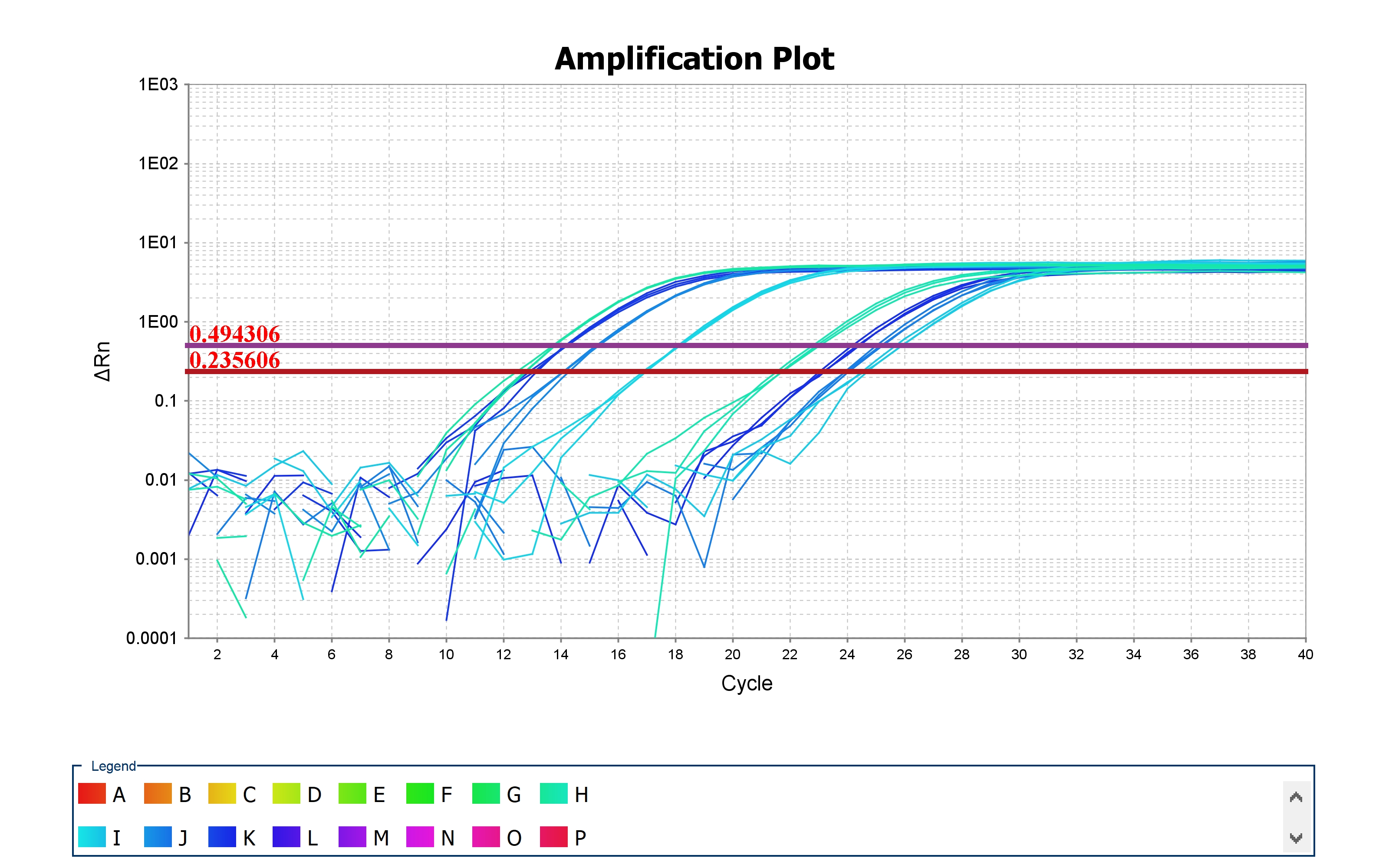

Supplement: Supplementary file 1 [file DataSheet_1.zip › Original data 1/Figure 1B/Amplification Plot.jpg]

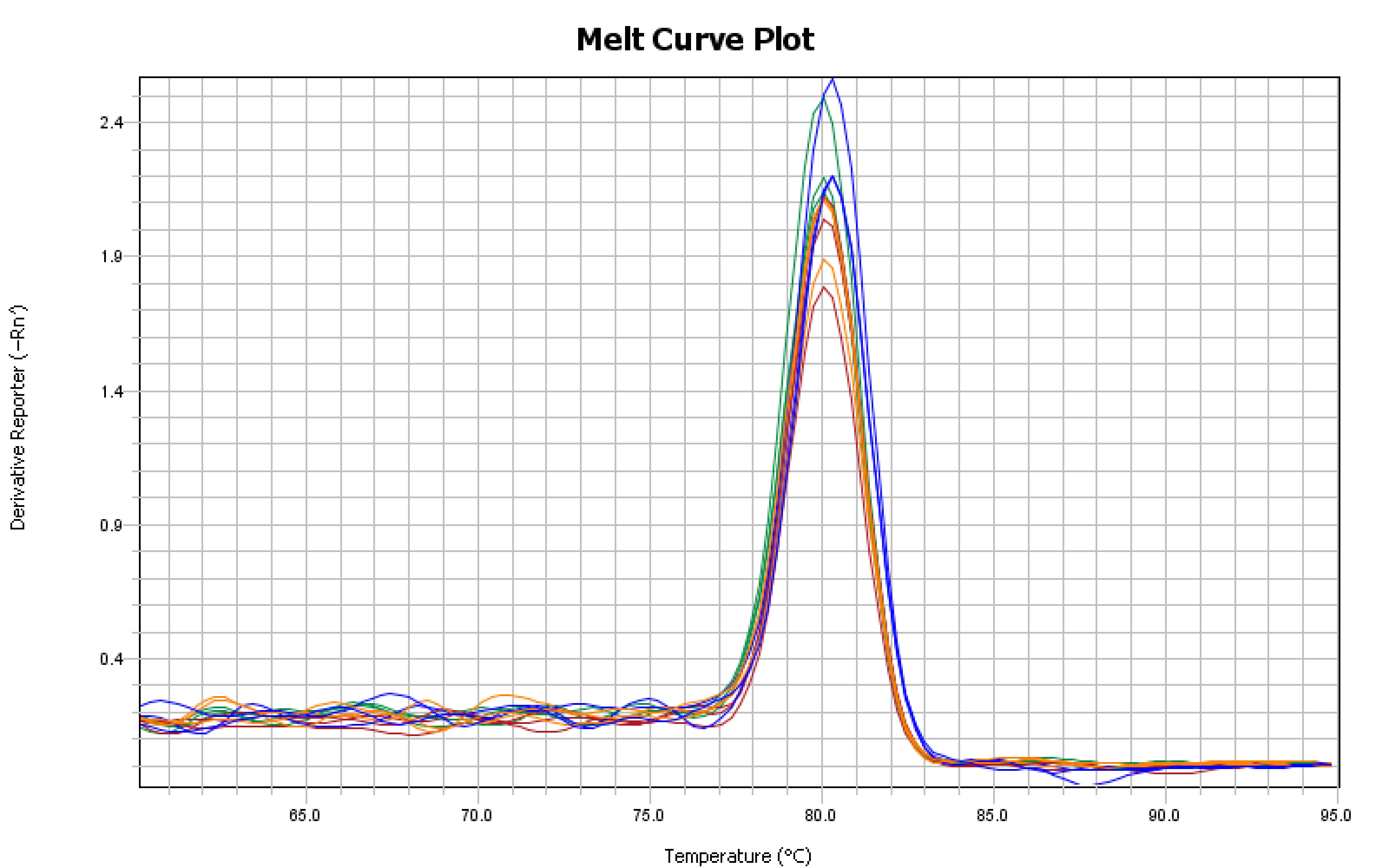

Supplement: Supplementary file 1 [file DataSheet_1.zip › Original data 1/Figure 1B/Melt Curve Plot H-DEPDC1B.jpg]

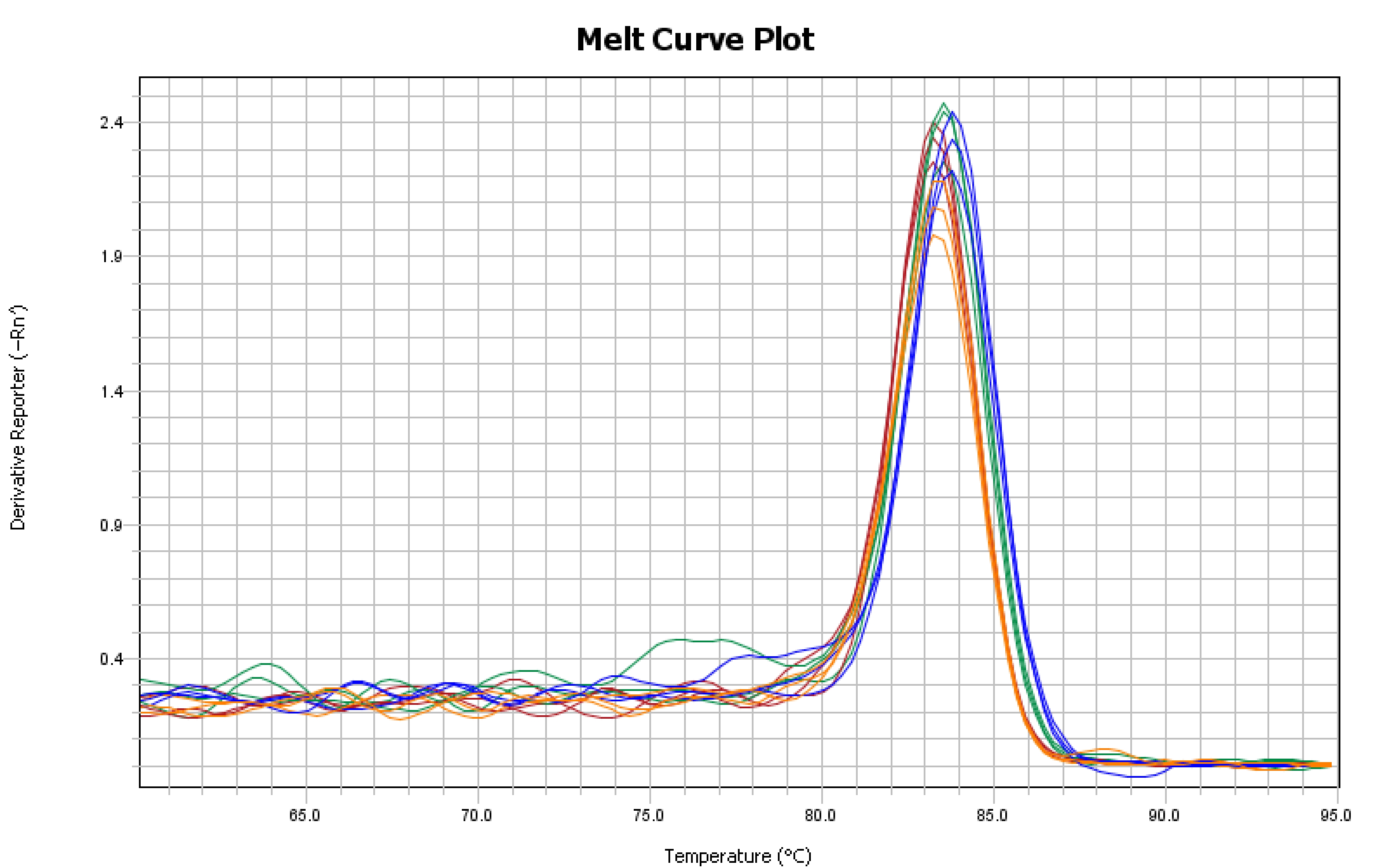

Supplement: Supplementary file 1 [file DataSheet_1.zip › Original data 1/Figure 1B/Melt Curve Plot H-GAPDH.jpg]

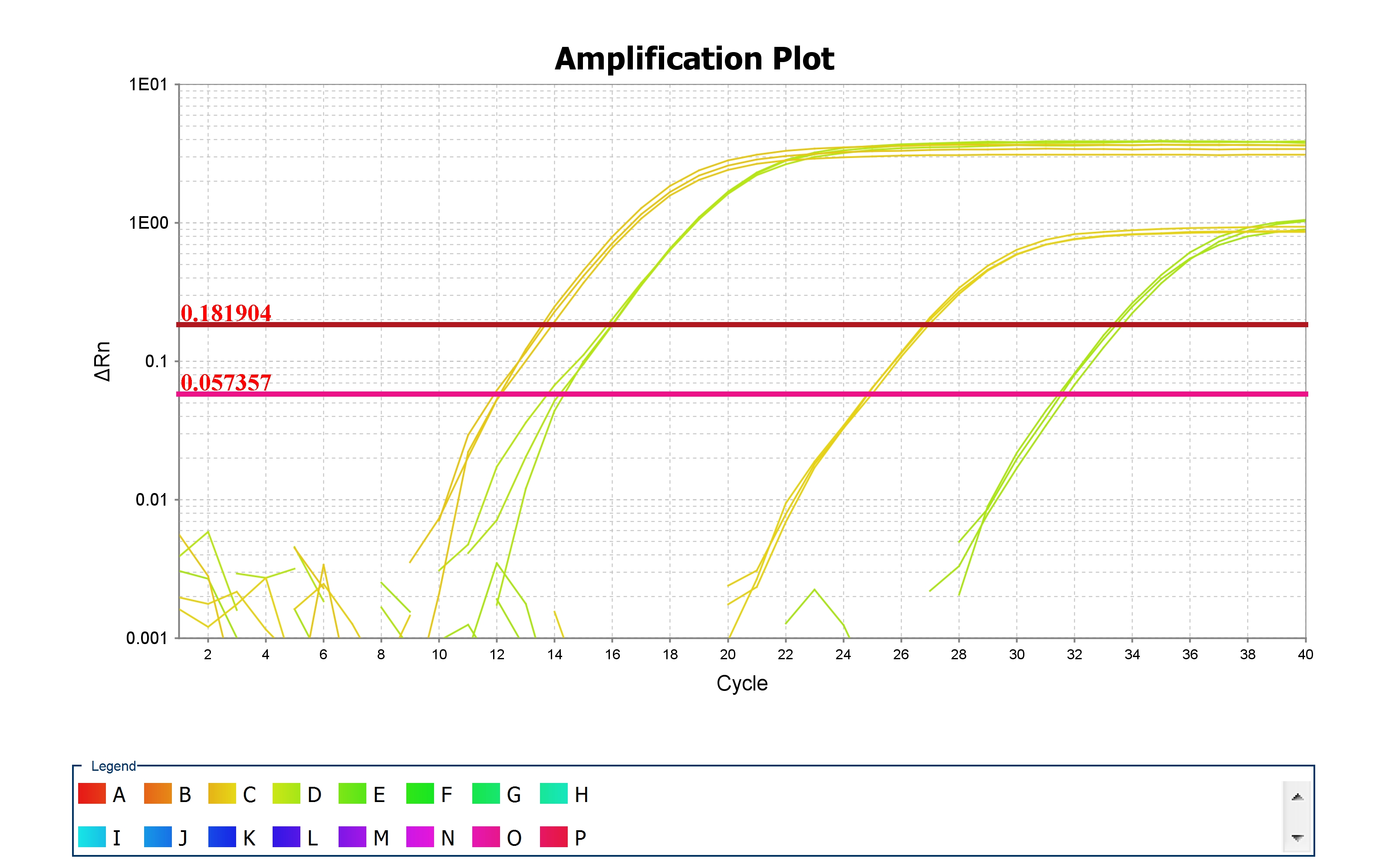

Supplement: Supplementary file 1 [file DataSheet_1.zip › Original data 1/Figure 2A/HCCC9810/Amplification Plot.jpg]

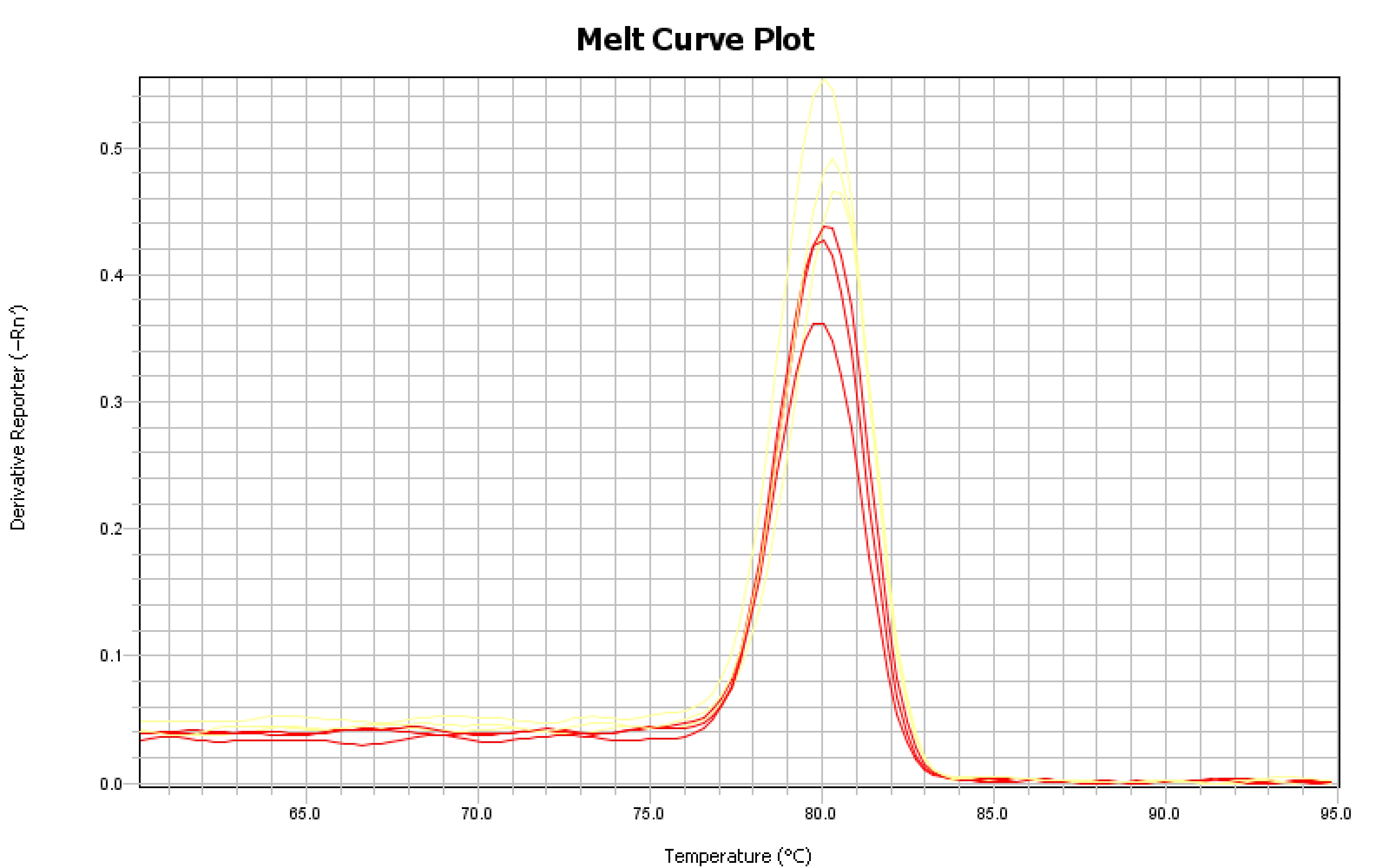

Supplement: Supplementary file 1 [file DataSheet_1.zip › Original data 1/Figure 2A/HCCC9810/Melt Curve Plot H-DEPDC1B.jpg]

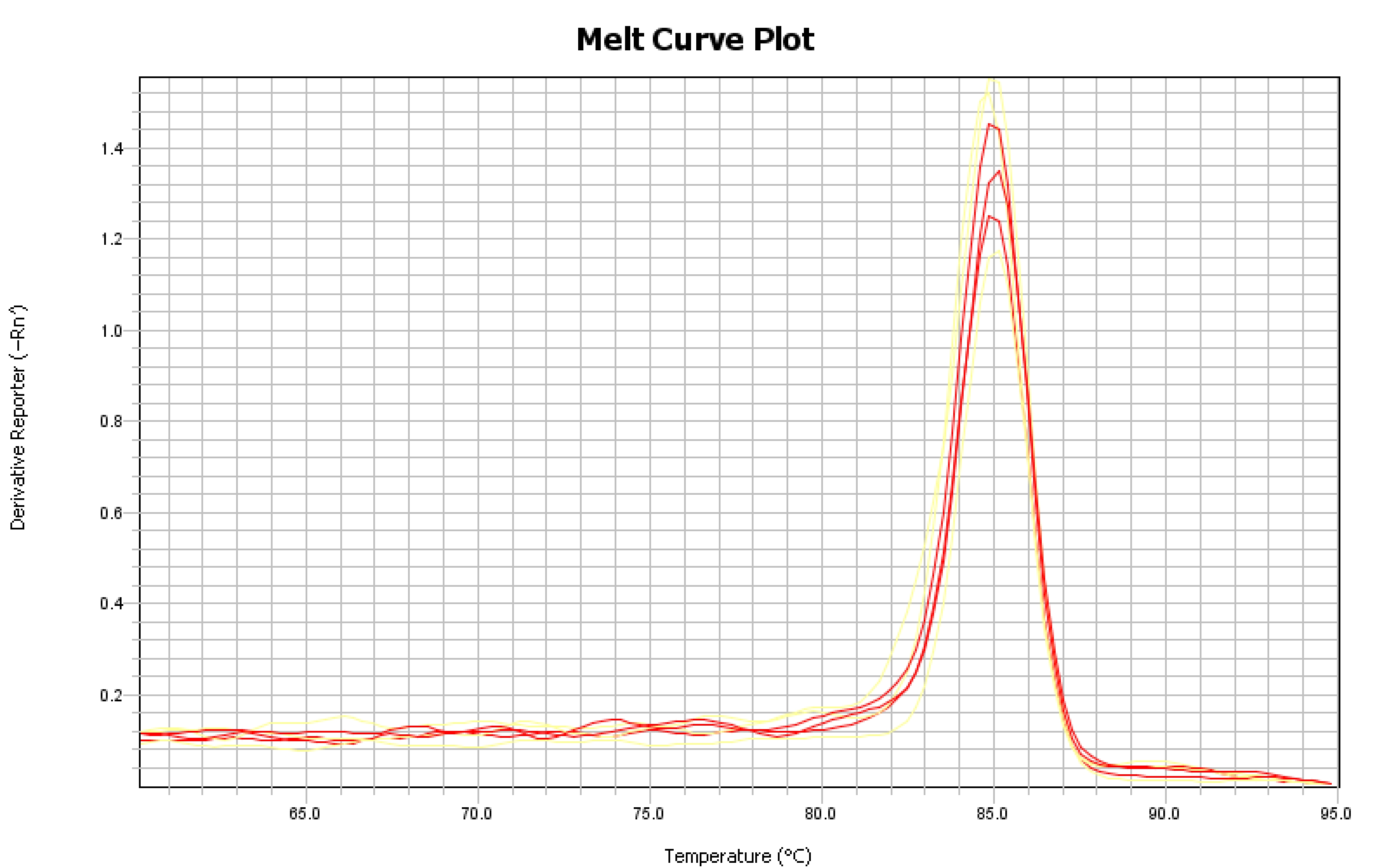

Supplement: Supplementary file 1 [file DataSheet_1.zip › Original data 1/Figure 2A/HCCC9810/Melt Curve Plot H-GAPDH.jpg]

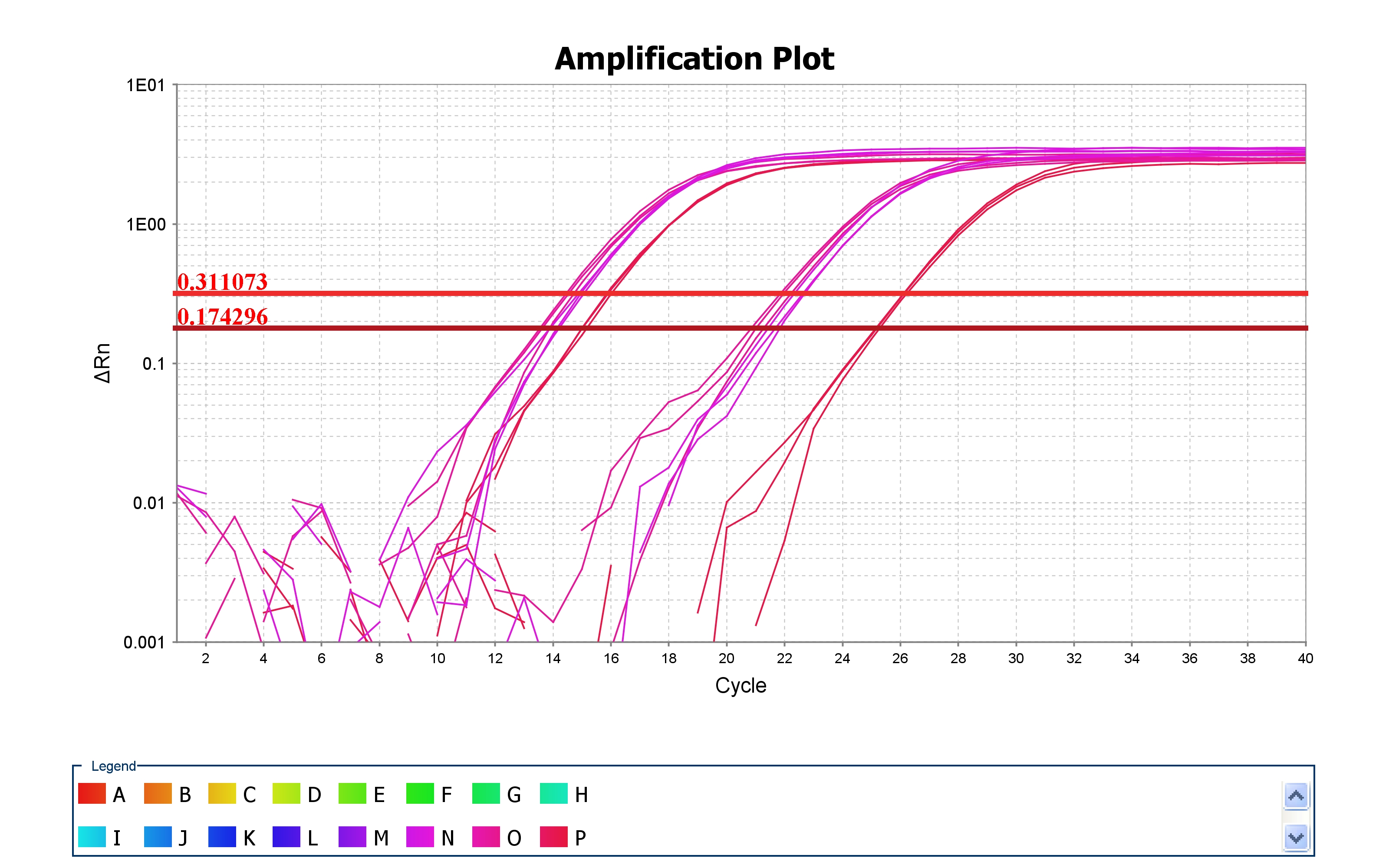

Supplement: Supplementary file 1 [file DataSheet_1.zip › Original data 1/Figure 2A/QBC939/Amplification Plot.jpg]

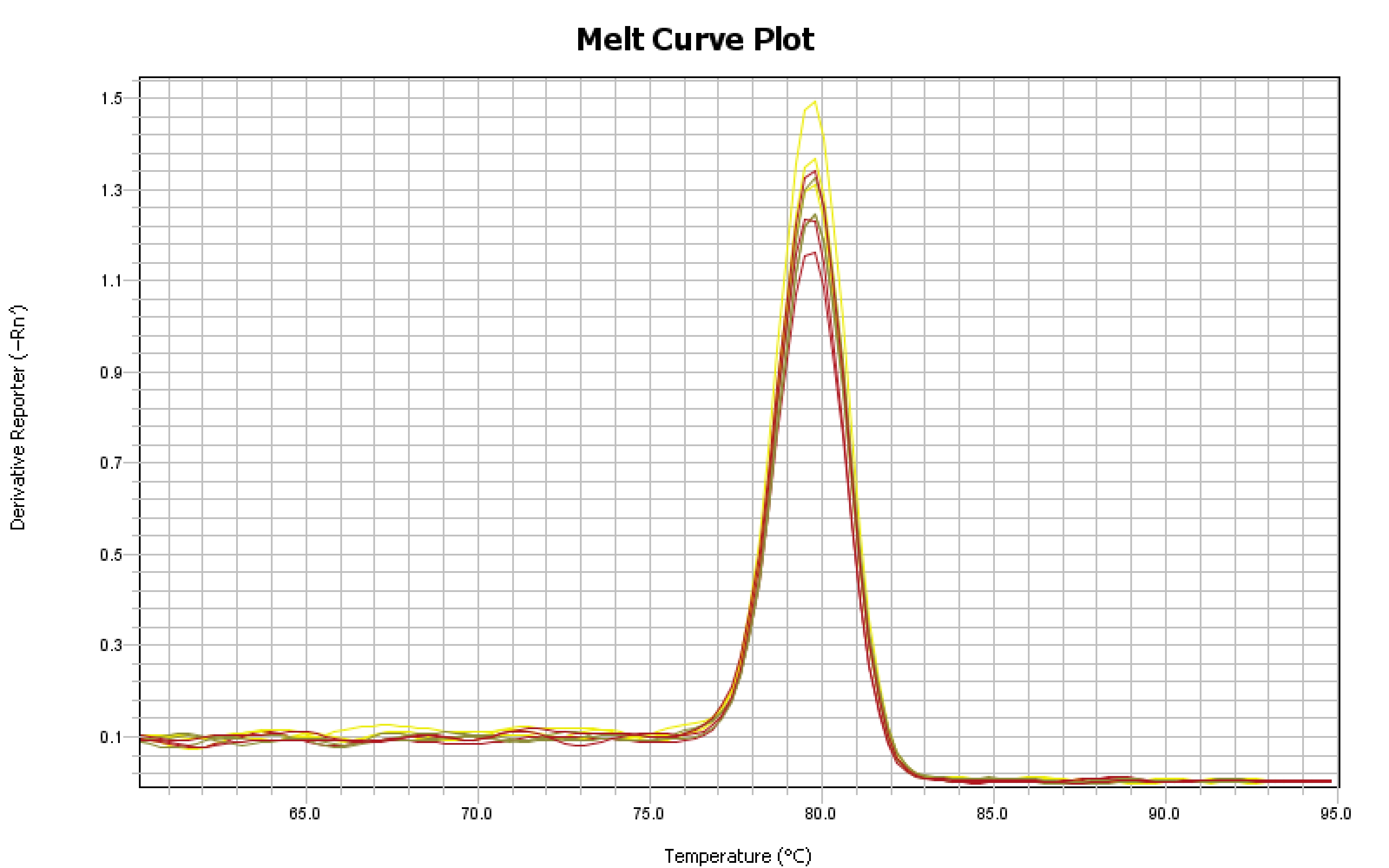

Supplement: Supplementary file 1 [file DataSheet_1.zip › Original data 1/Figure 2A/QBC939/Melt Curve Plot H-DEPDC1B.jpg]

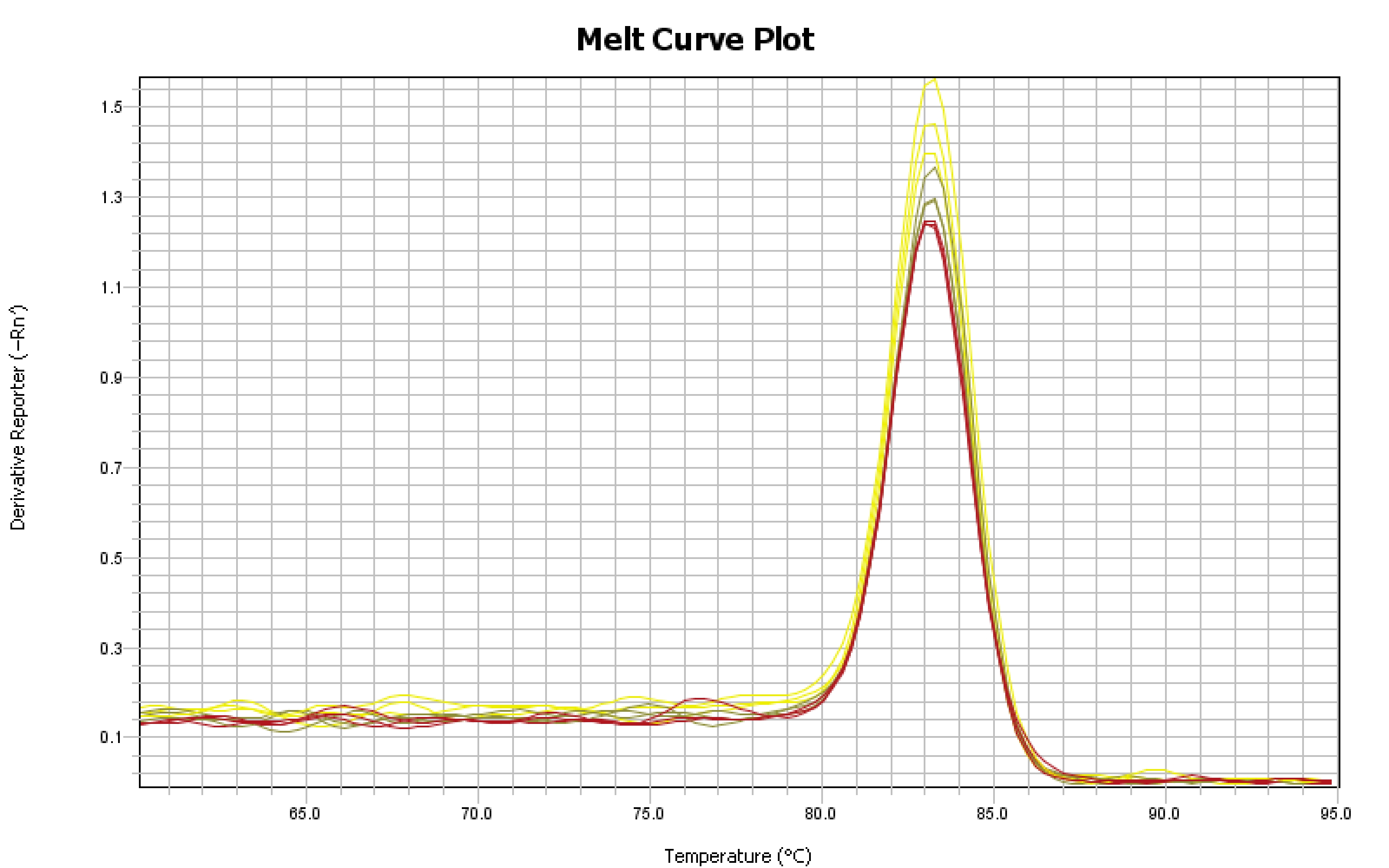

Supplement: Supplementary file 1 [file DataSheet_1.zip › Original data 1/Figure 2A/QBC939/Melt Curve Plot H-GAPDH.jpg]

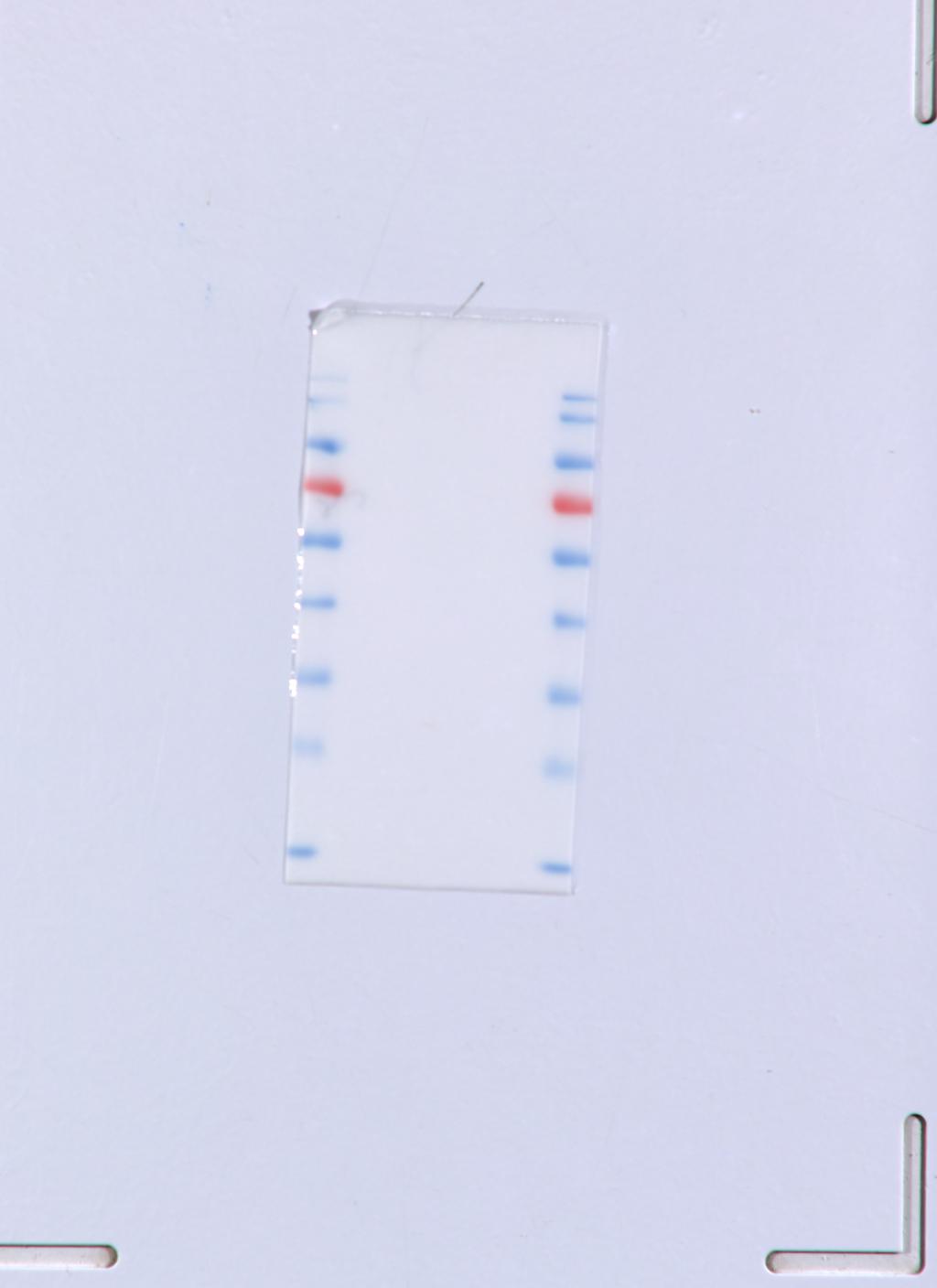

Supplement: Supplementary file 1 [file DataSheet_1.zip › Original data 1/Figure 2B/HCCC-9810 DEPDC1B M/HCCC-9810 DEPDC1B M.jpg]

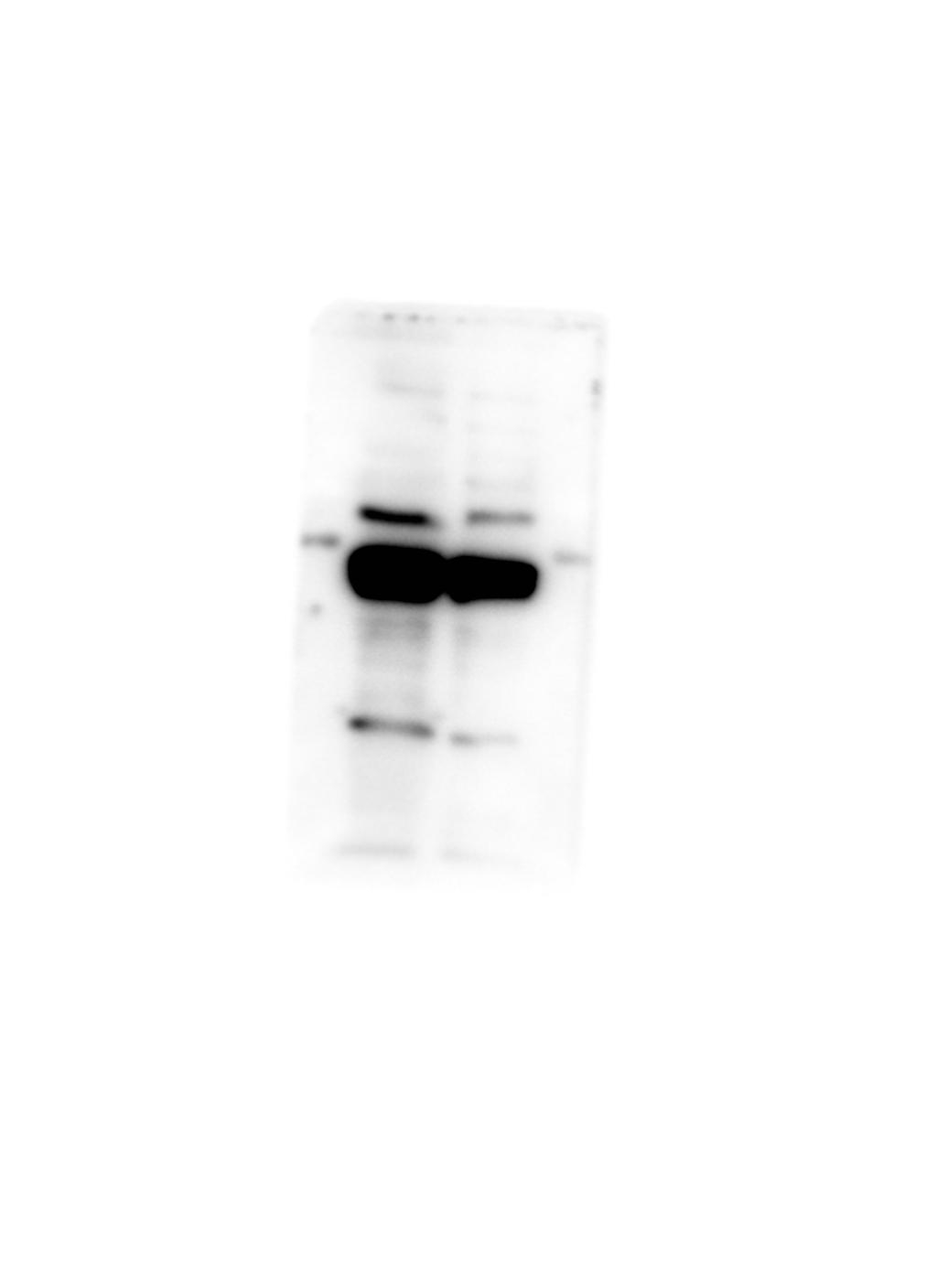

Supplement: Supplementary file 1 [file DataSheet_1.zip › Original data 1/Figure 2B/HCCC-9810 DEPDC1B/HCCC-9810 DEPDC1B.jpg]

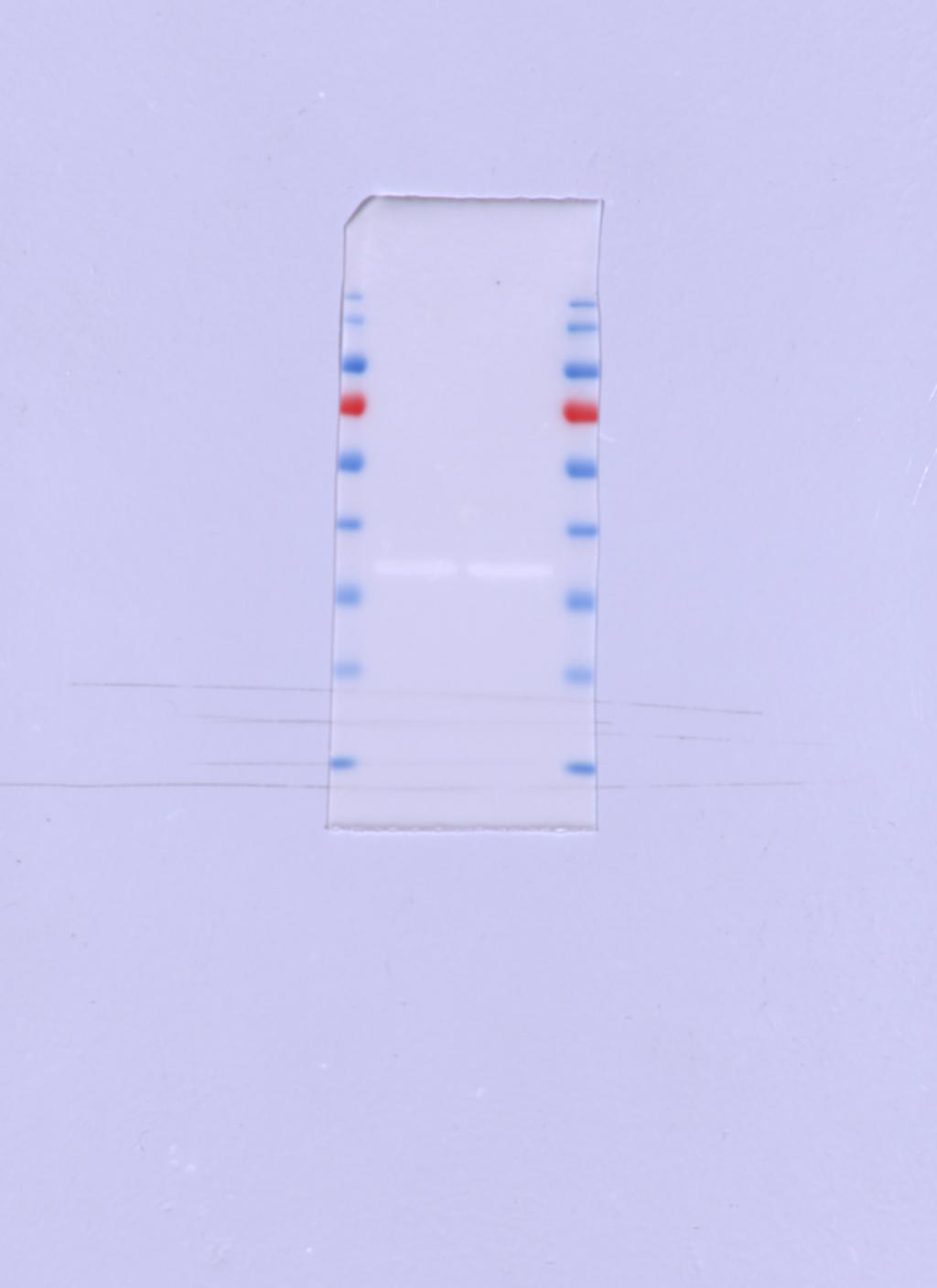

Supplement: Supplementary file 1 [file DataSheet_1.zip › Original data 1/Figure 2B/HCCC-9810 GAPDH M/HCCC-9810 GAPDH M.jpg]

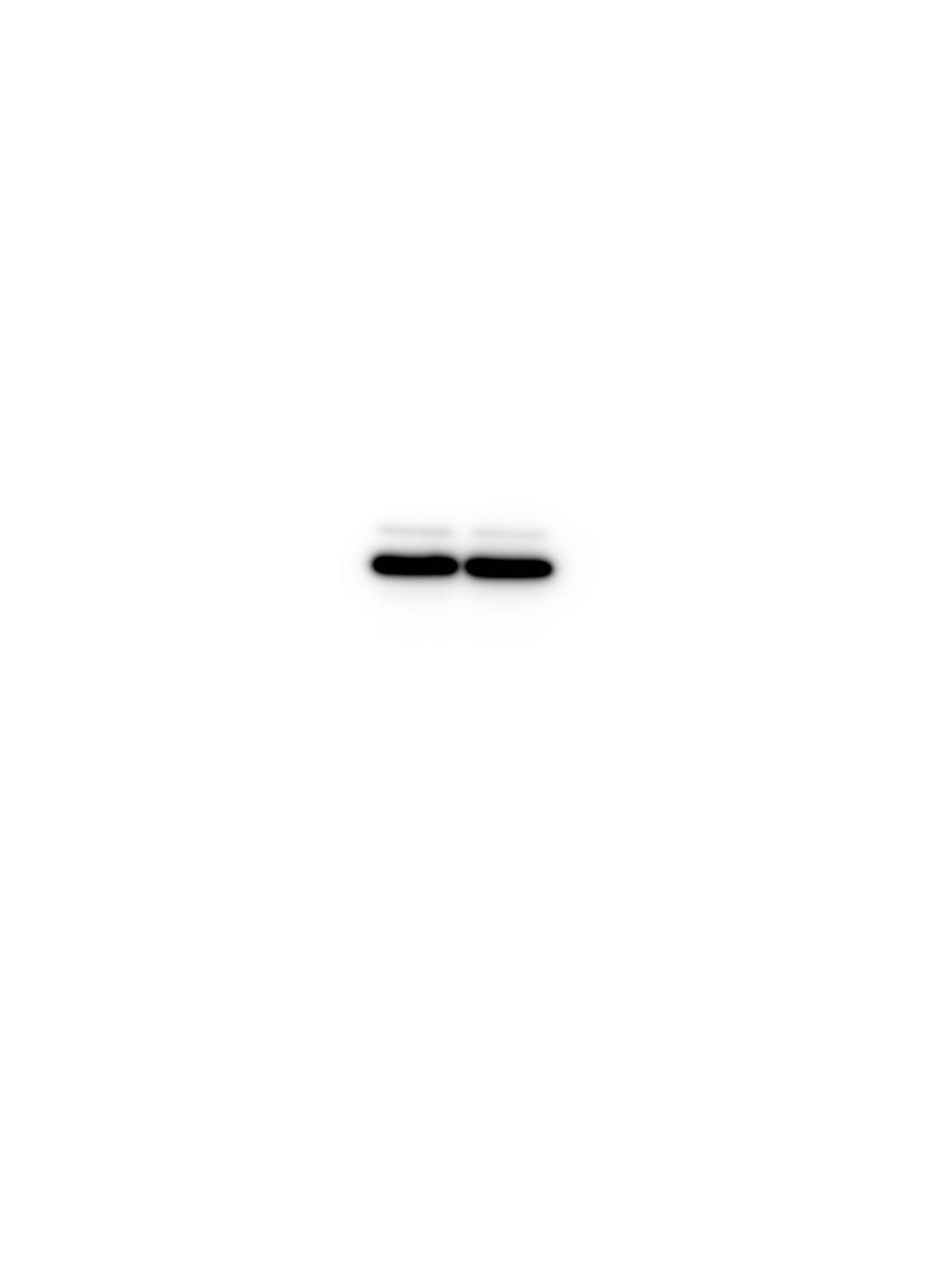

Supplement: Supplementary file 1 [file DataSheet_1.zip › Original data 1/Figure 2B/HCCC-9810 GAPDH/HCCC-9810 GAPDH.jpg]

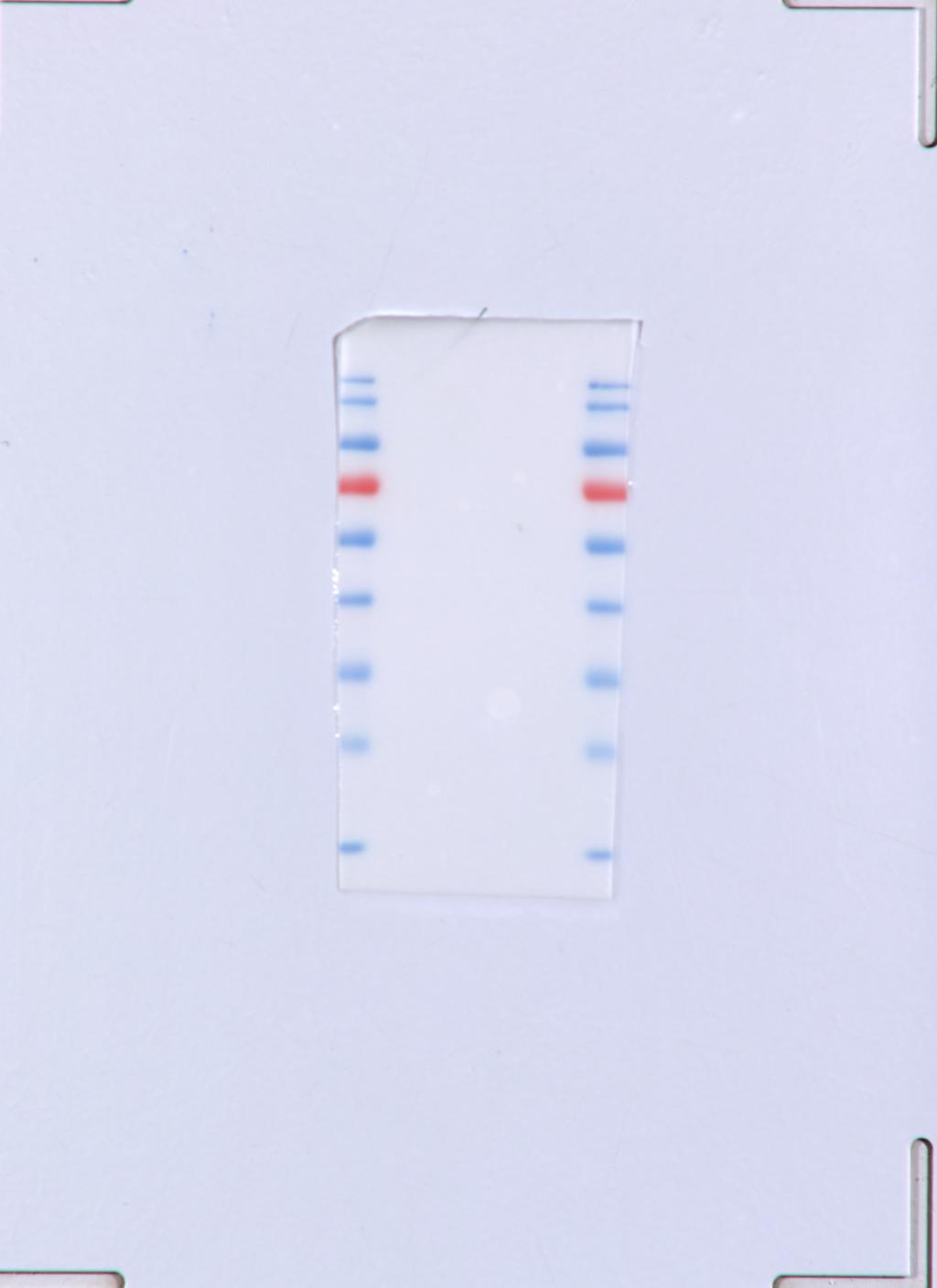

Supplement: Supplementary file 1 [file DataSheet_1.zip › Original data 1/Figure 2B/QBC939 DEPDC1B M/QBC939 DEPDC1B M.jpg]

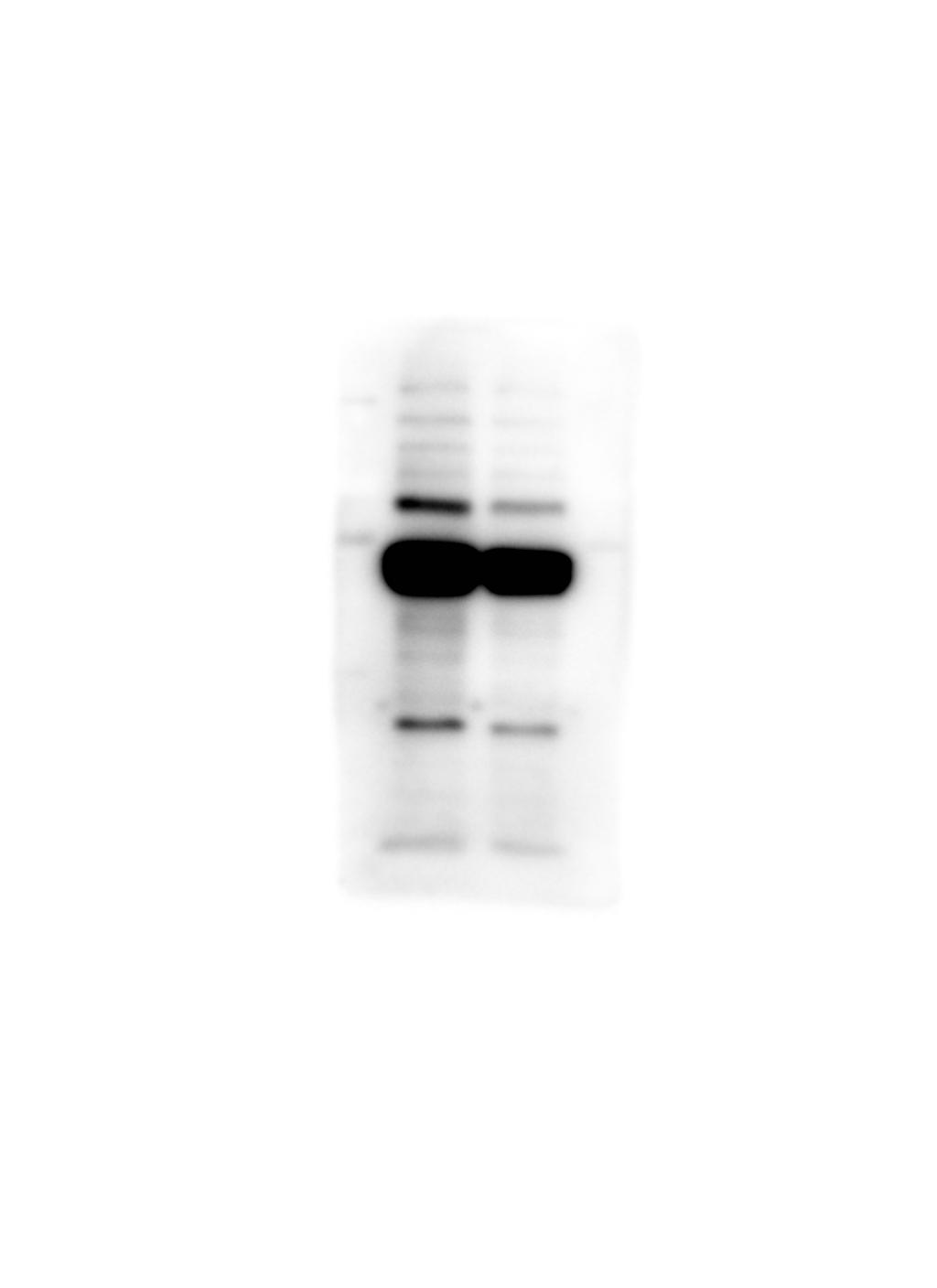

Supplement: Supplementary file 1 [file DataSheet_1.zip › Original data 1/Figure 2B/QBC939 DEPDC1B/QBC939 DEPDC1B.jpg]

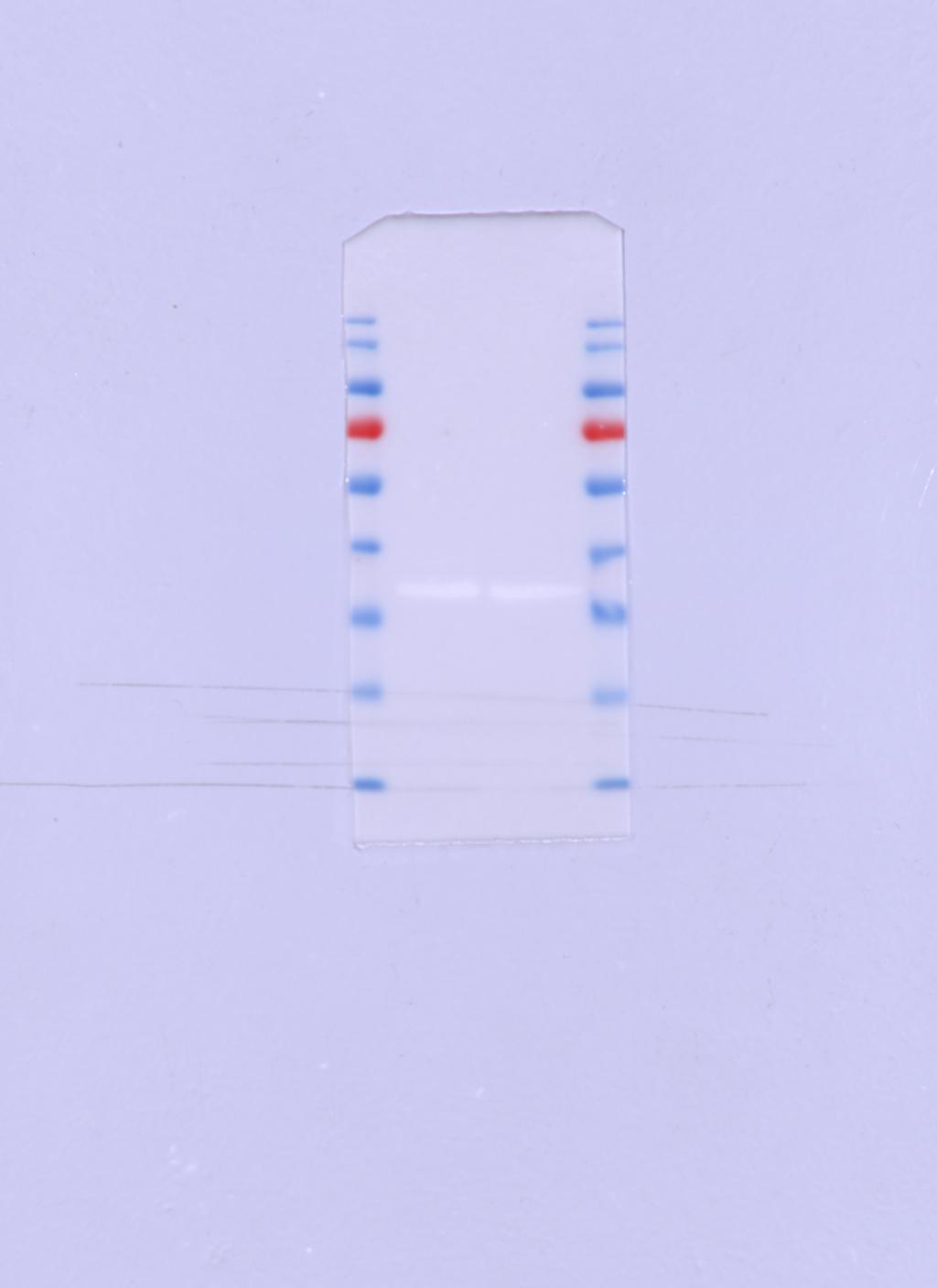

Supplement: Supplementary file 1 [file DataSheet_1.zip › Original data 1/Figure 2B/QBC939 GAPDH M/QBC939 GAPDH M.jpg]

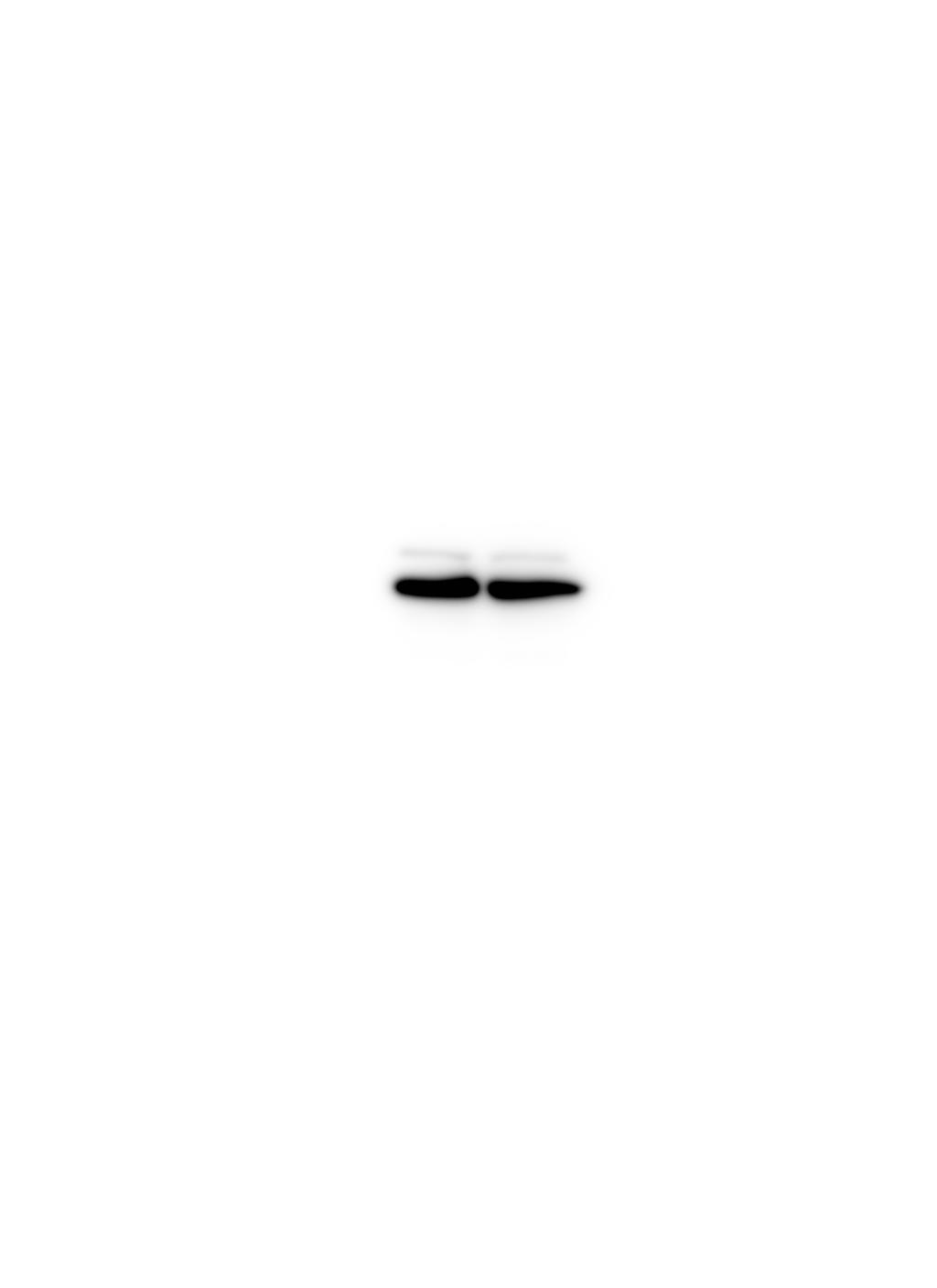

Supplement: Supplementary file 1 [file DataSheet_1.zip › Original data 1/Figure 2B/QBC939 GAPDH/QBC939 GAPDH.jpg]

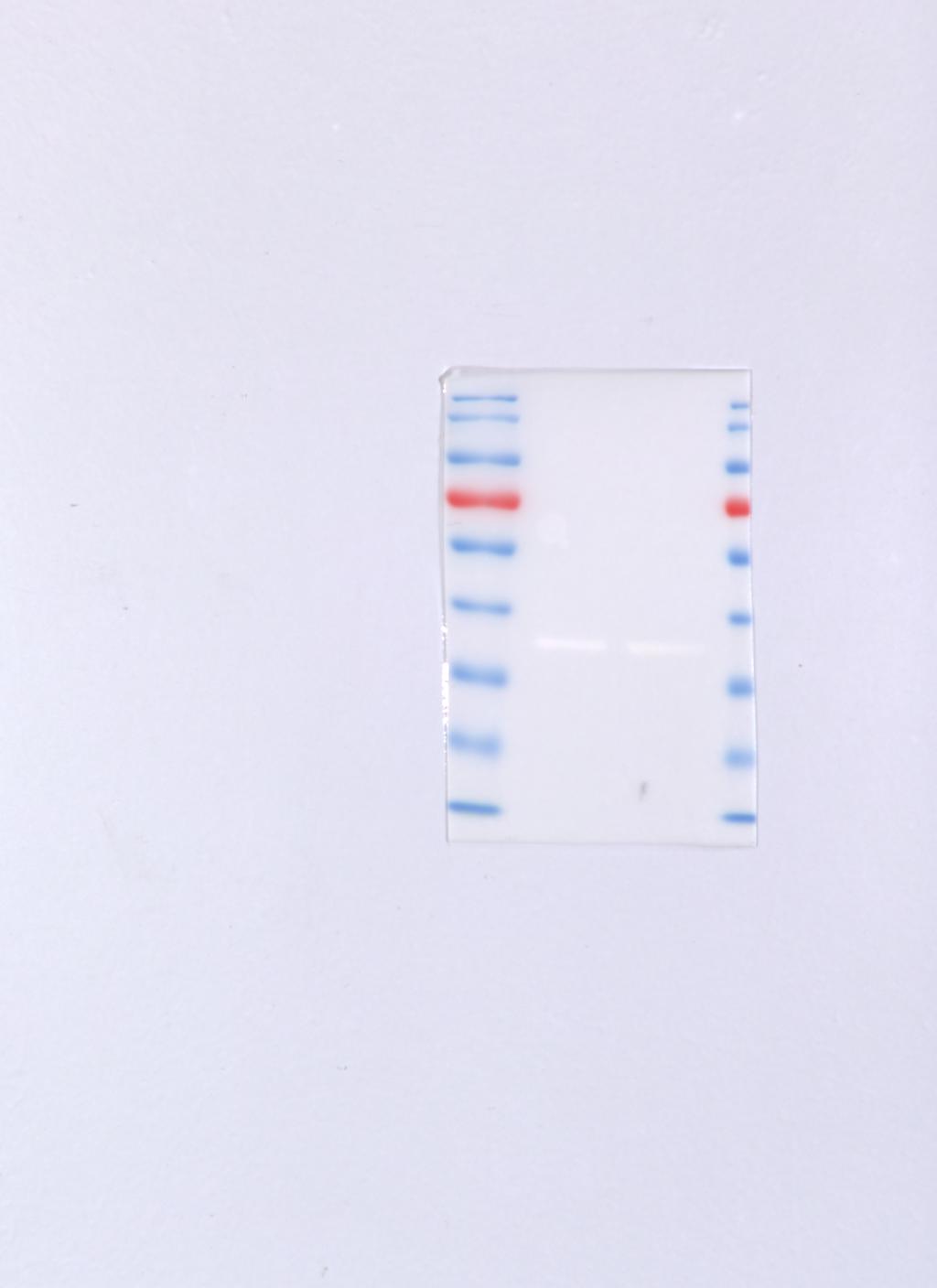

Supplement: Supplementary file 1 [file DataSheet_1.zip › Original data 1/Figure 2H/HCCC-9810/E-Cadherin M/E-Cadherin M.jpg]

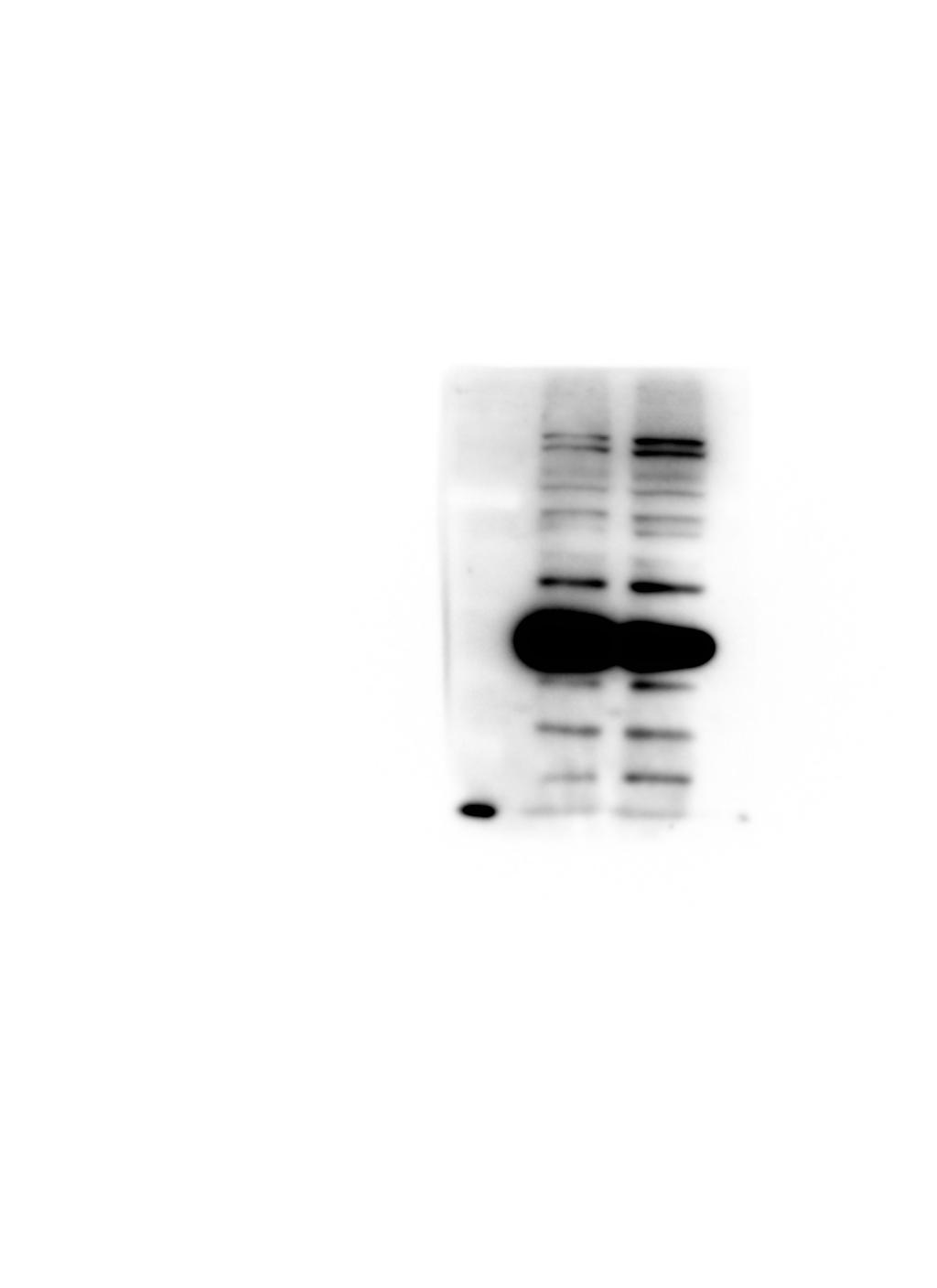

Supplement: Supplementary file 1 [file DataSheet_1.zip › Original data 1/Figure 2H/HCCC-9810/E-Cadherin/E-Cadherin.jpg]

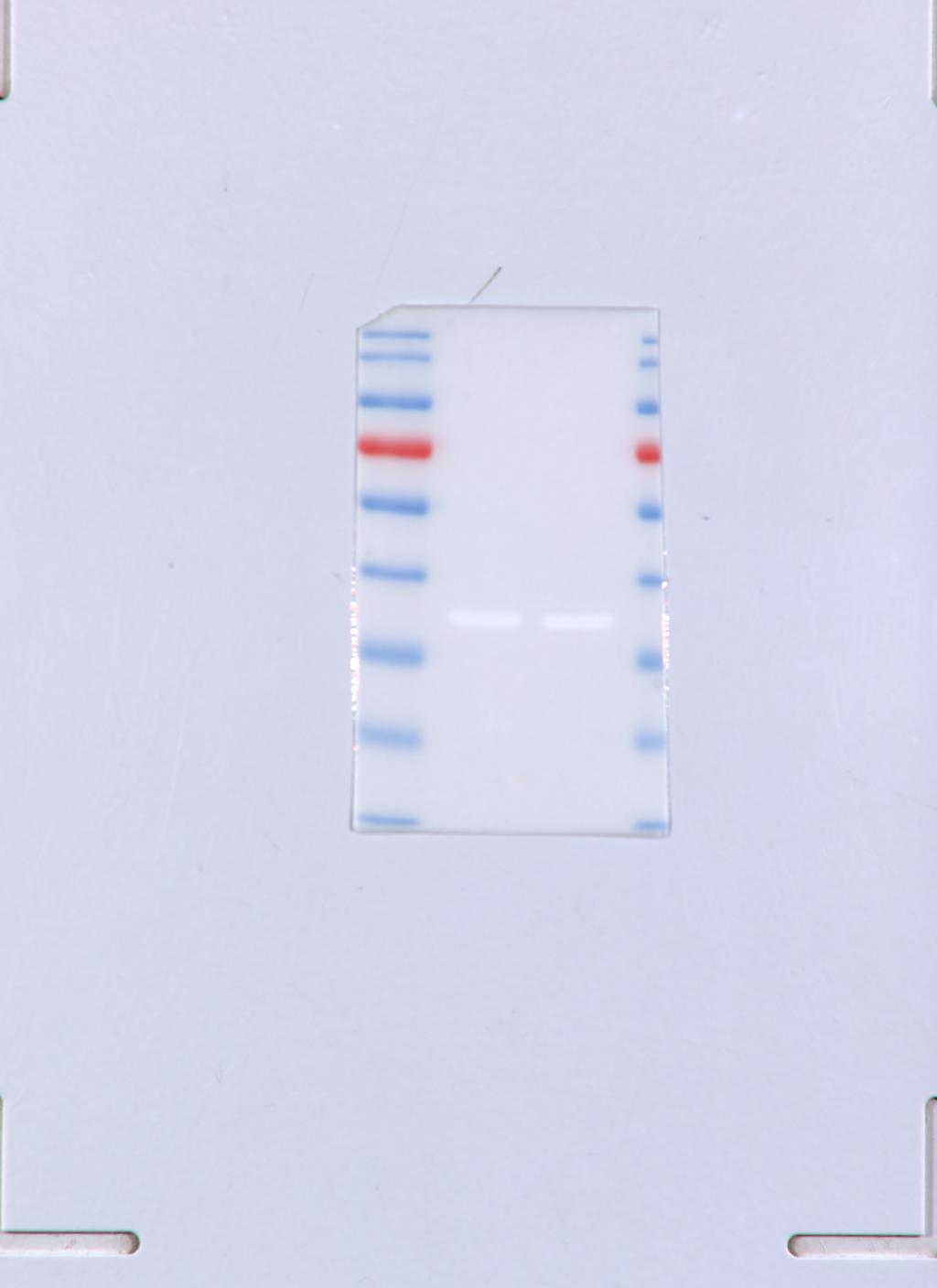

Supplement: Supplementary file 1 [file DataSheet_1.zip › Original data 1/Figure 2H/HCCC-9810/GAPDH M/GAPDH M.jpg]

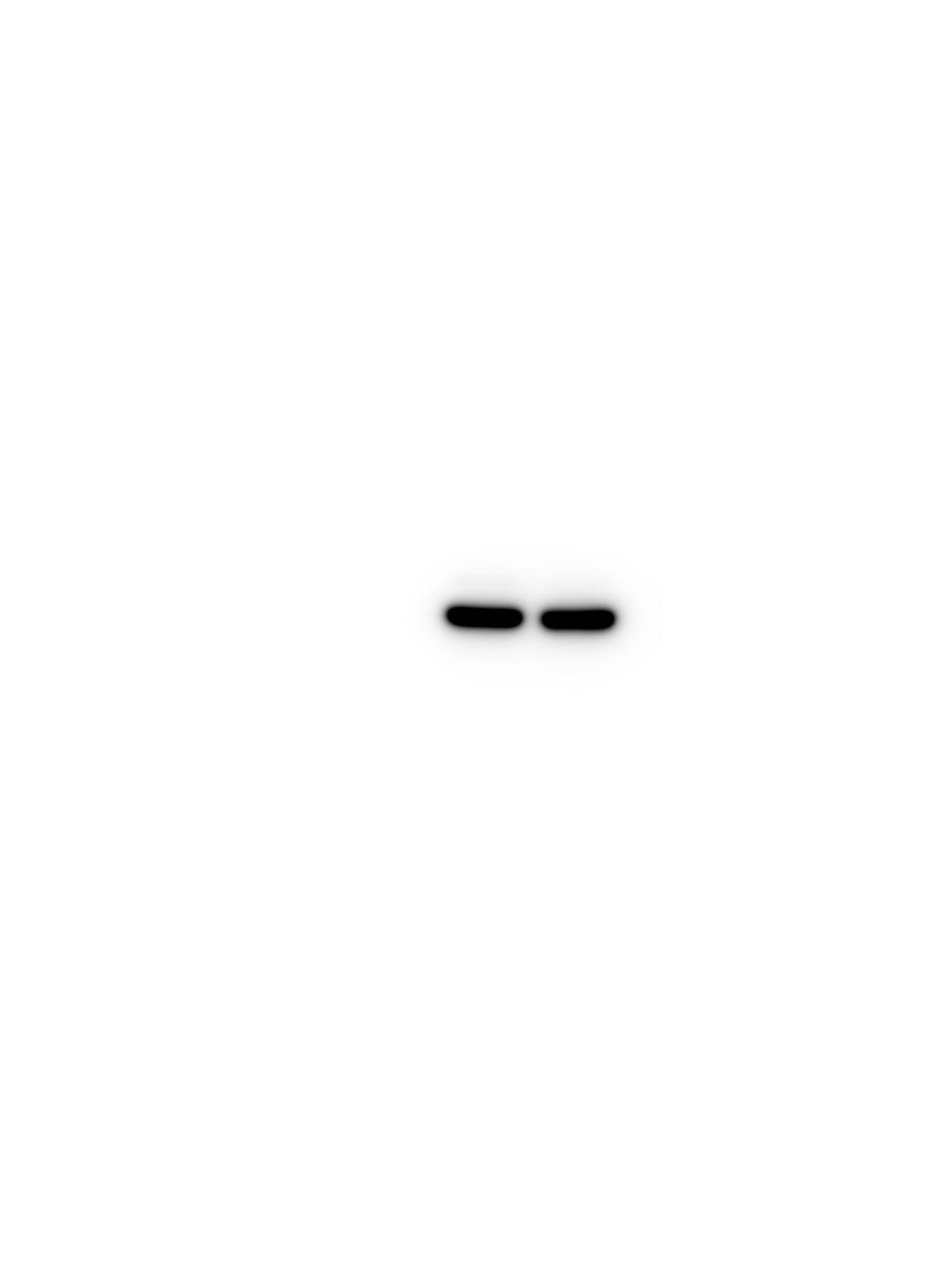

Supplement: Supplementary file 1 [file DataSheet_1.zip › Original data 1/Figure 2H/HCCC-9810/GAPDH/GAPDH.jpg]

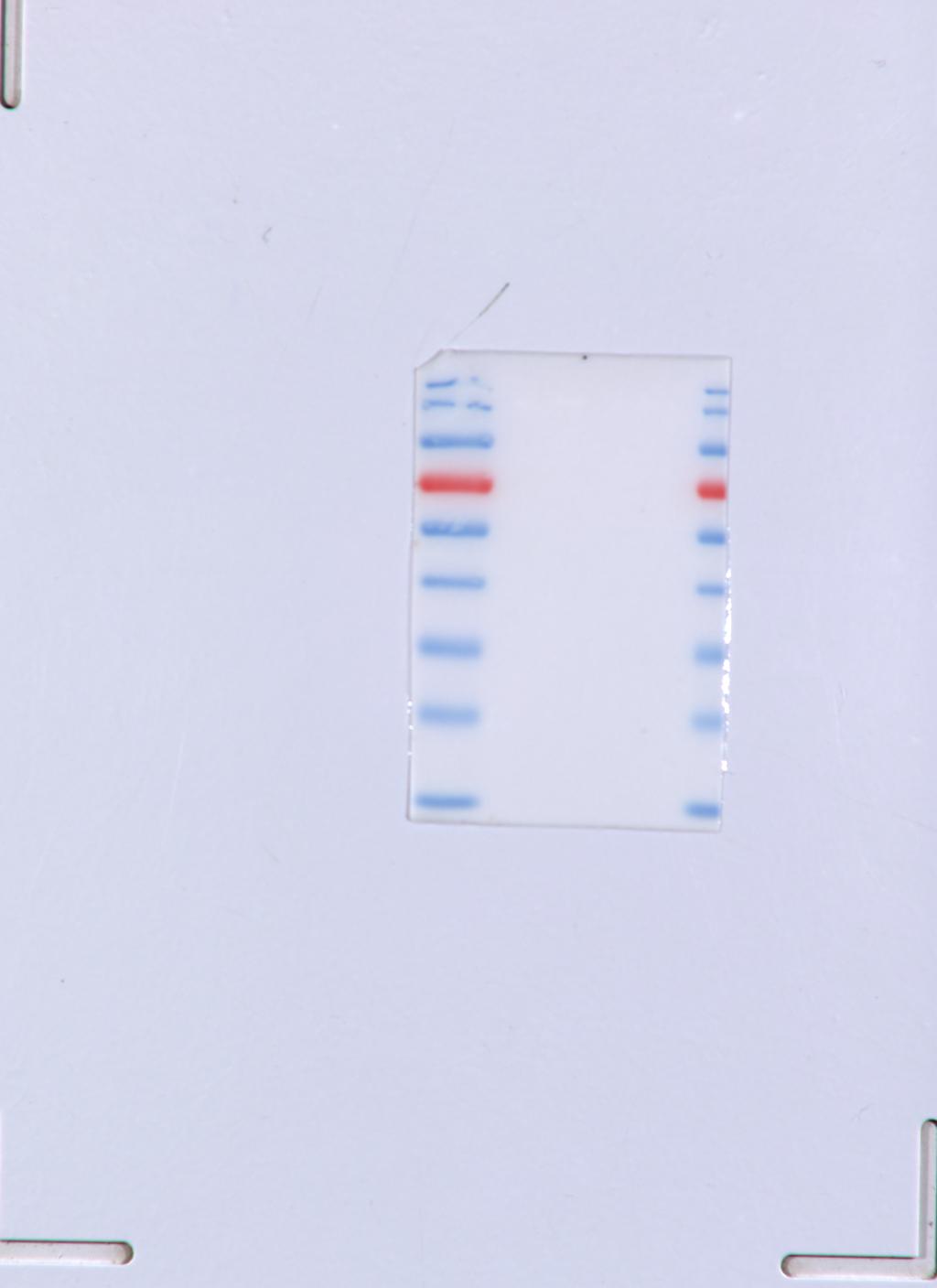

Supplement: Supplementary file 1 [file DataSheet_1.zip › Original data 1/Figure 2H/HCCC-9810/N-cadherin M/N-cadherin M.jpg]

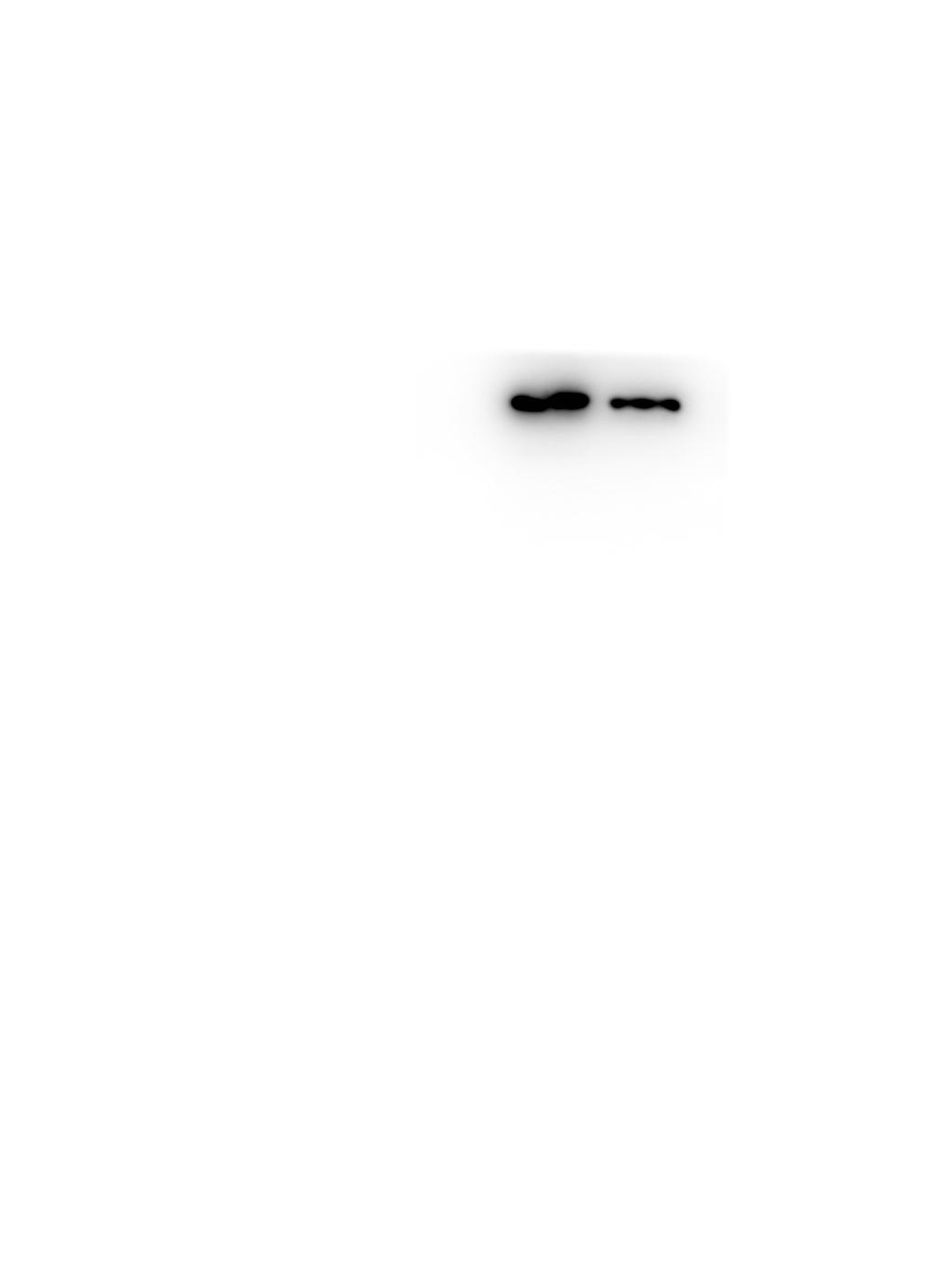

Supplement: Supplementary file 1 [file DataSheet_1.zip › Original data 1/Figure 2H/HCCC-9810/N-cadherin/N-cadherin.jpg]

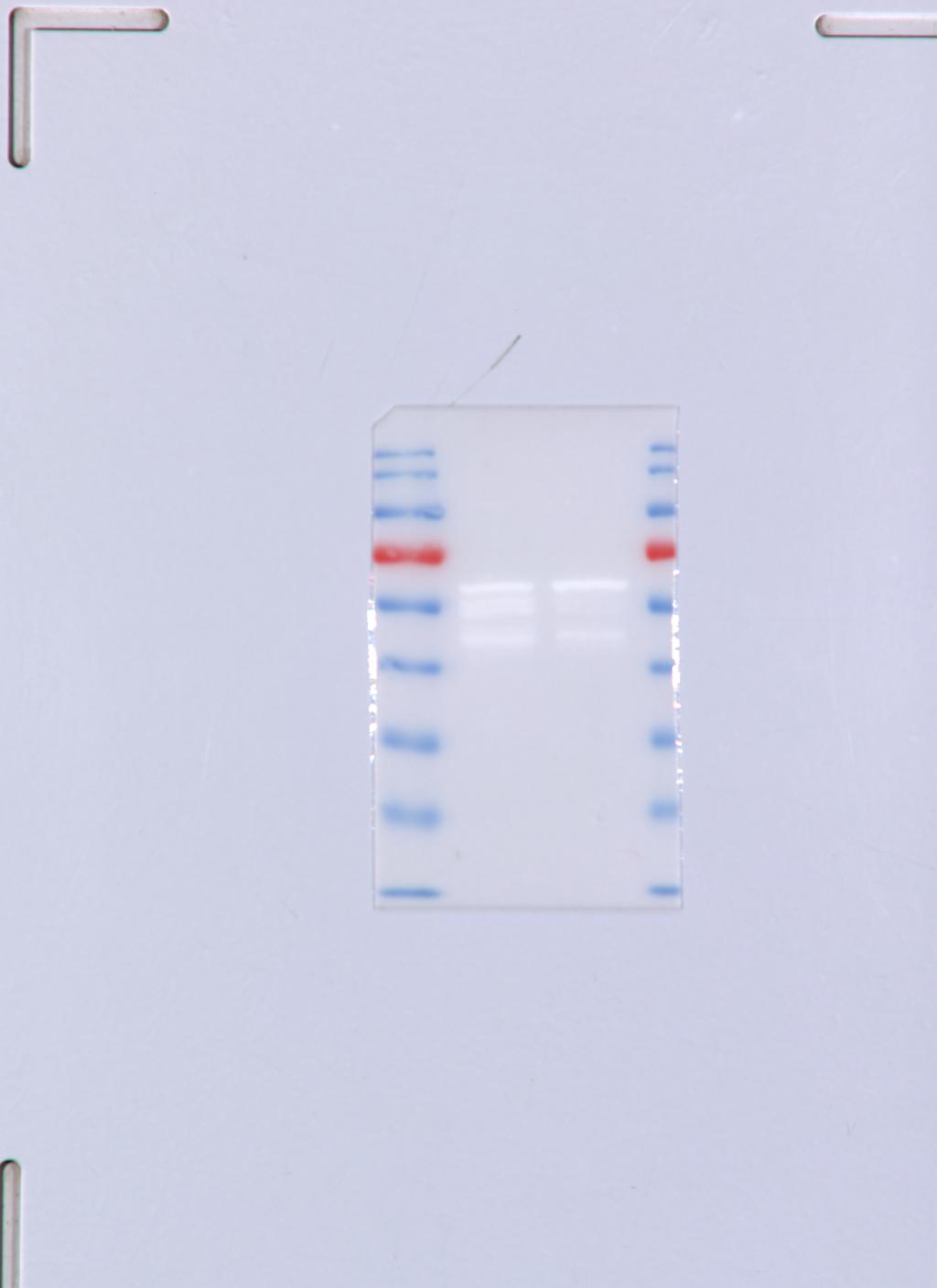

Supplement: Supplementary file 1 [file DataSheet_1.zip › Original data 1/Figure 2H/HCCC-9810/Vimentin M/Vimentin M.jpg]

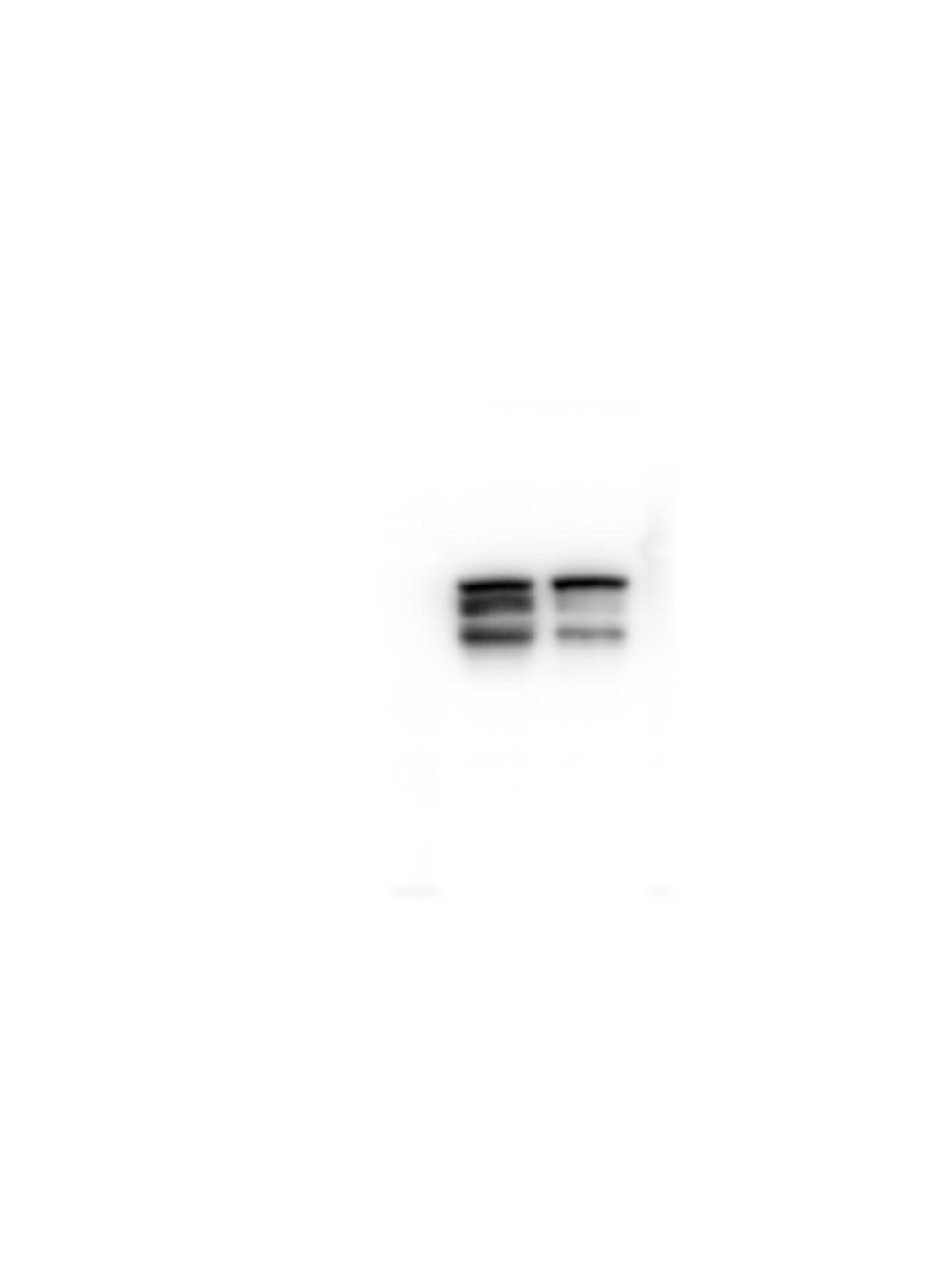

Supplement: Supplementary file 1 [file DataSheet_1.zip › Original data 1/Figure 2H/HCCC-9810/Vimentin/Vimentin.jpg]

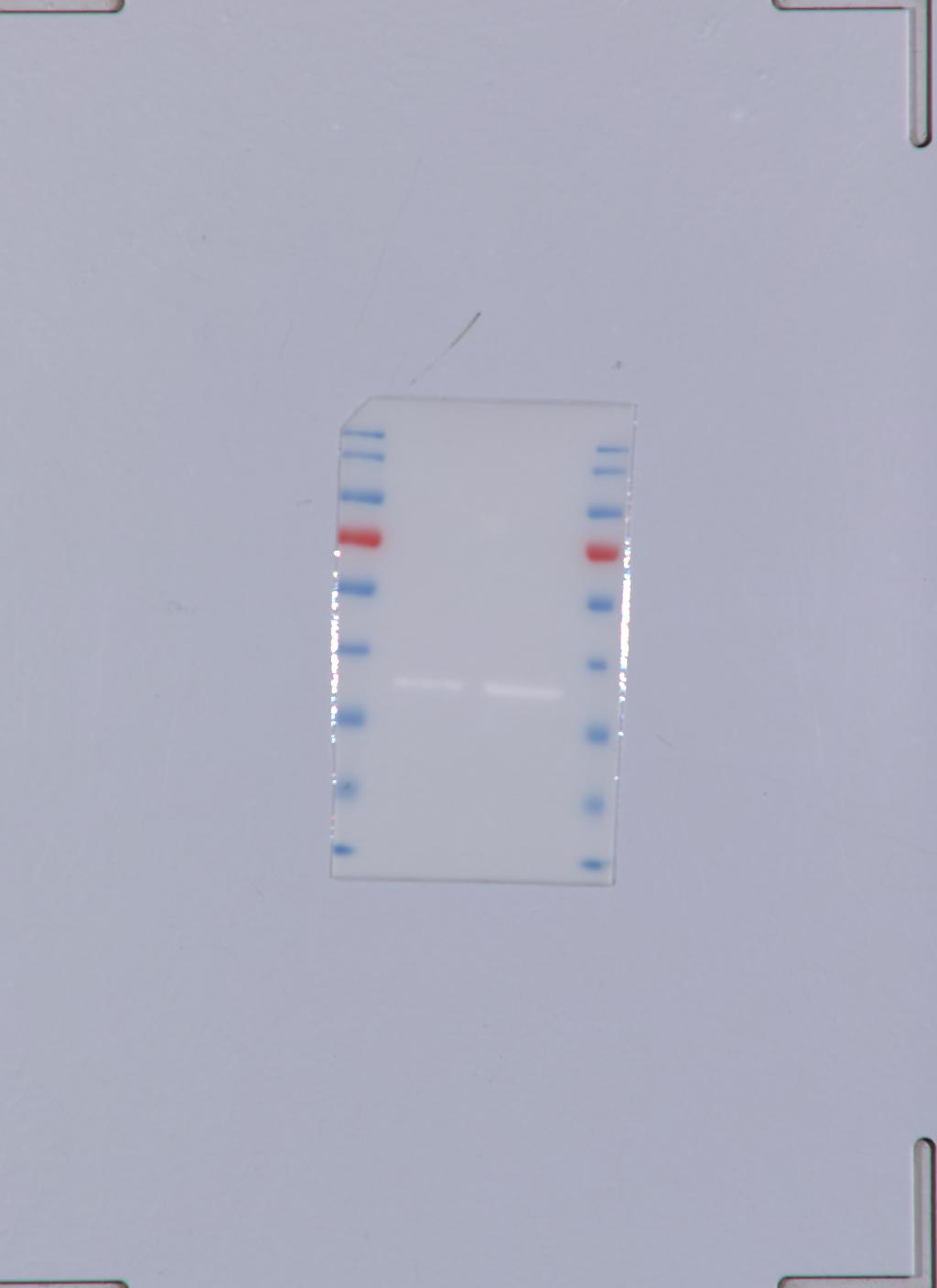

Supplement: Supplementary file 1 [file DataSheet_1.zip › Original data 1/Figure 2H/QBC939/E-Cadherin M/E-Cadherin M.jpg]

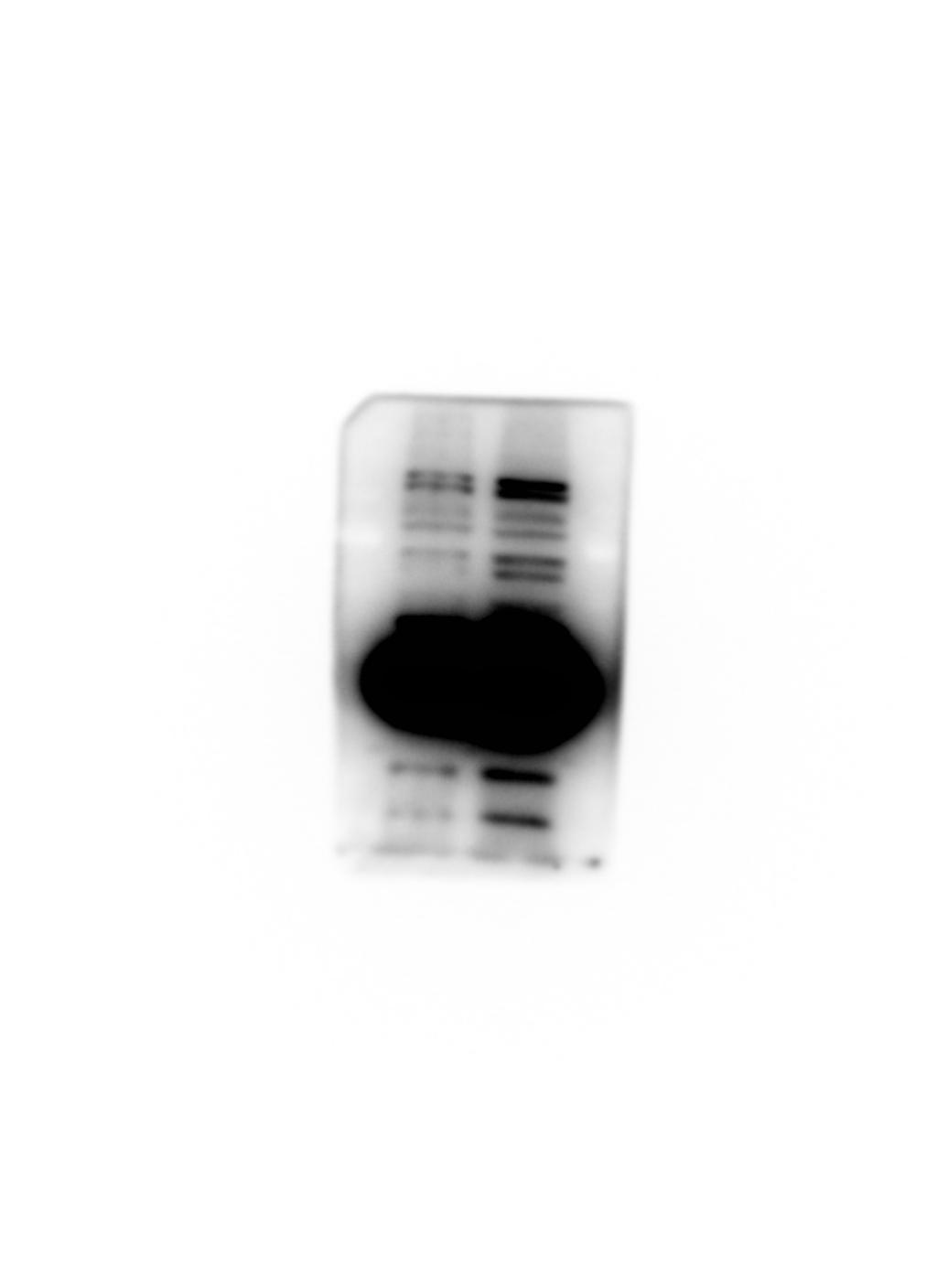

Supplement: Supplementary file 1 [file DataSheet_1.zip › Original data 1/Figure 2H/QBC939/E-Cadherin/E-Cadherin.jpg]

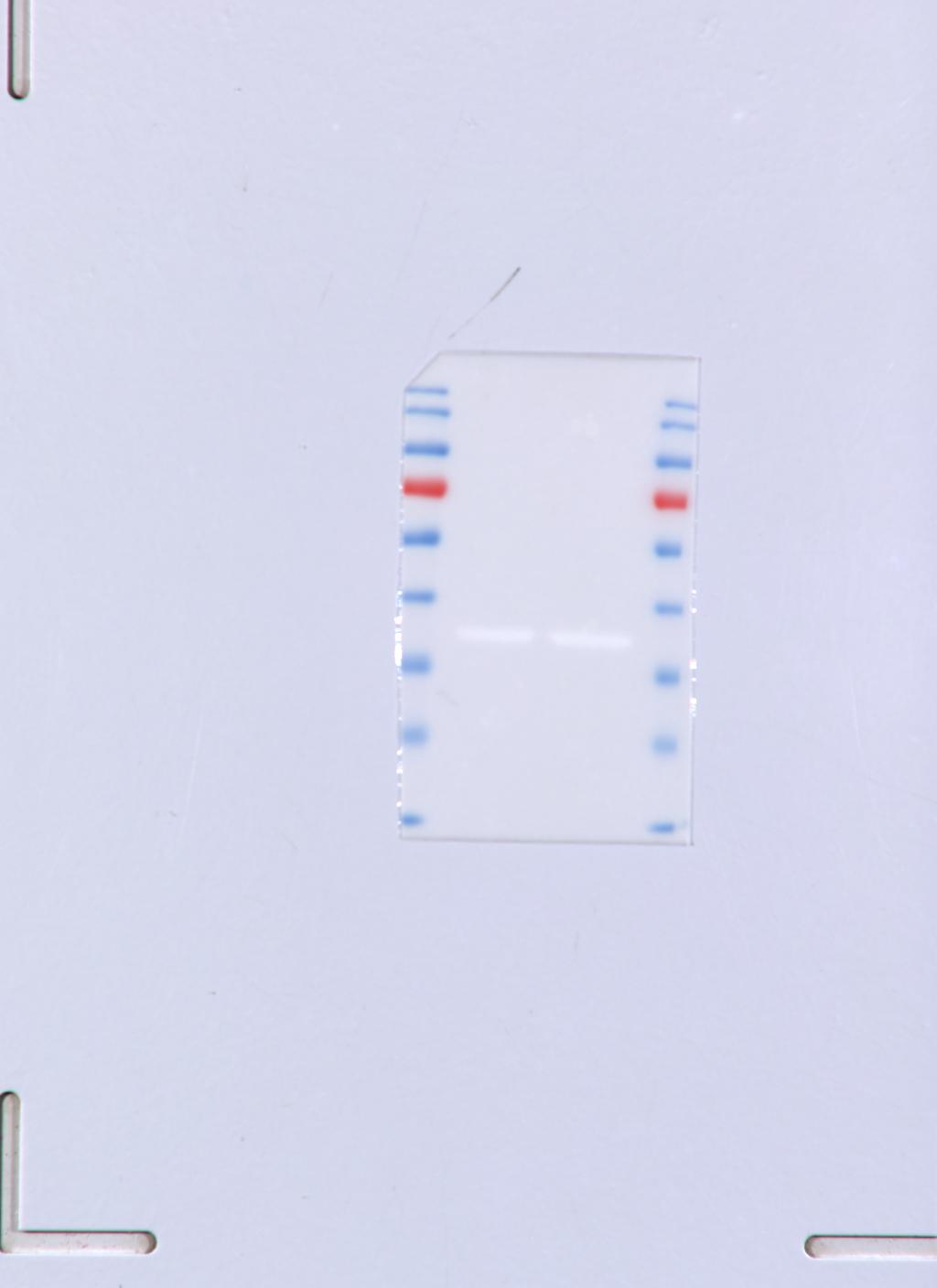

Supplement: Supplementary file 1 [file DataSheet_1.zip › Original data 1/Figure 2H/QBC939/GAPDH M/GAPDH M.jpg]

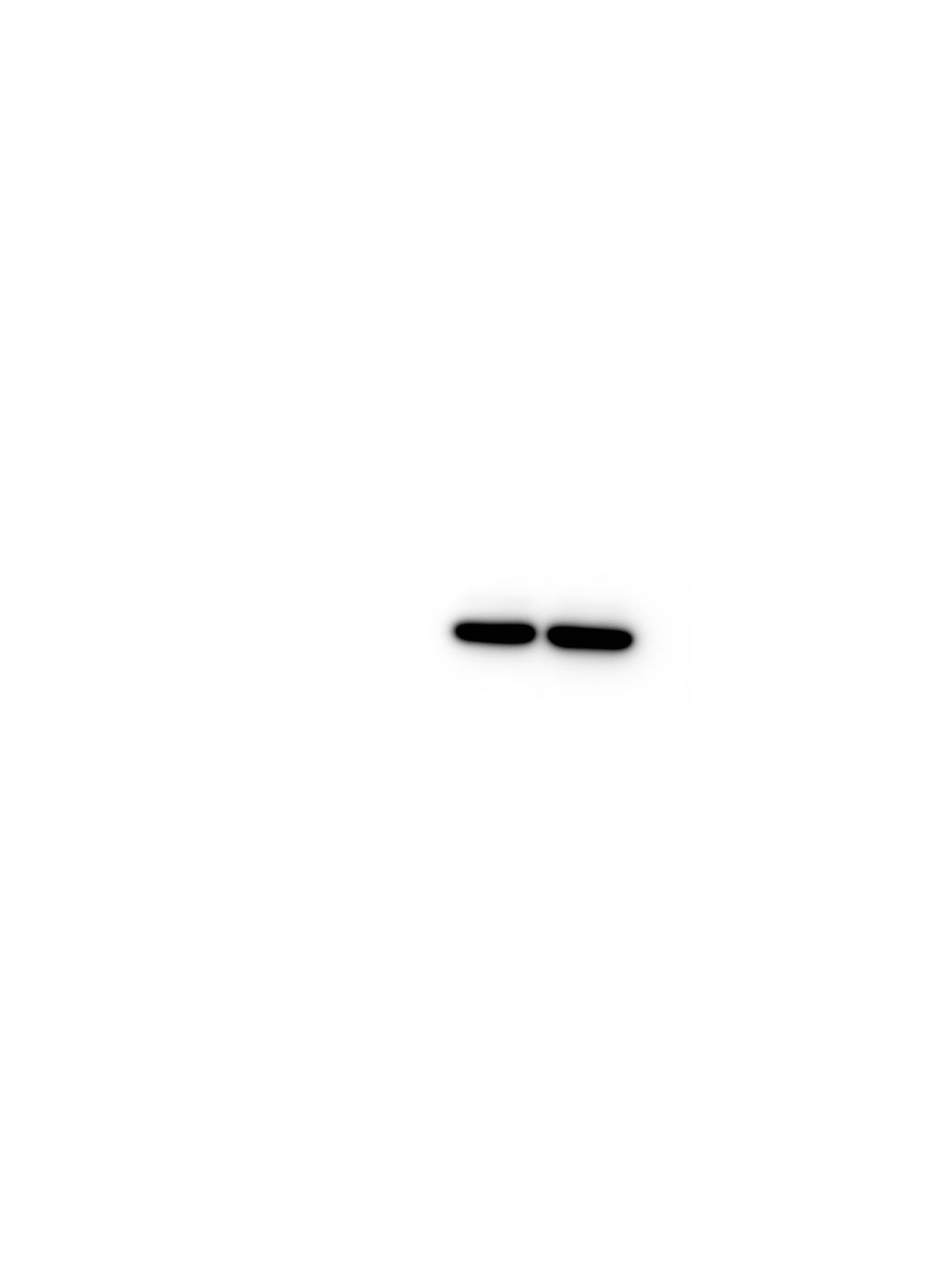

Supplement: Supplementary file 1 [file DataSheet_1.zip › Original data 1/Figure 2H/QBC939/GAPDH/GAPDH.jpg]

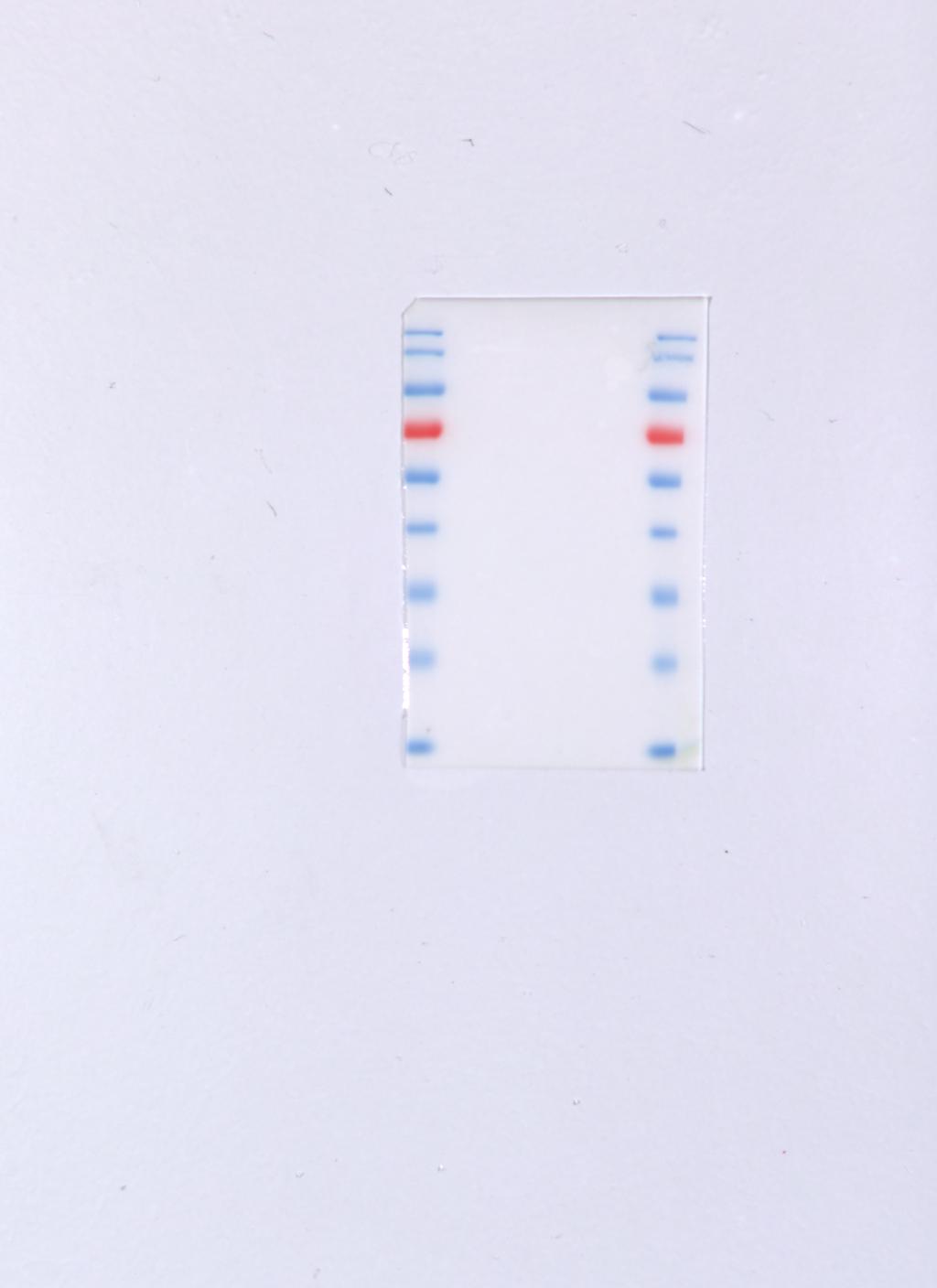

Supplement: Supplementary file 1 [file DataSheet_1.zip › Original data 1/Figure 2H/QBC939/N-cadherin M/N-cadherin M.jpg]

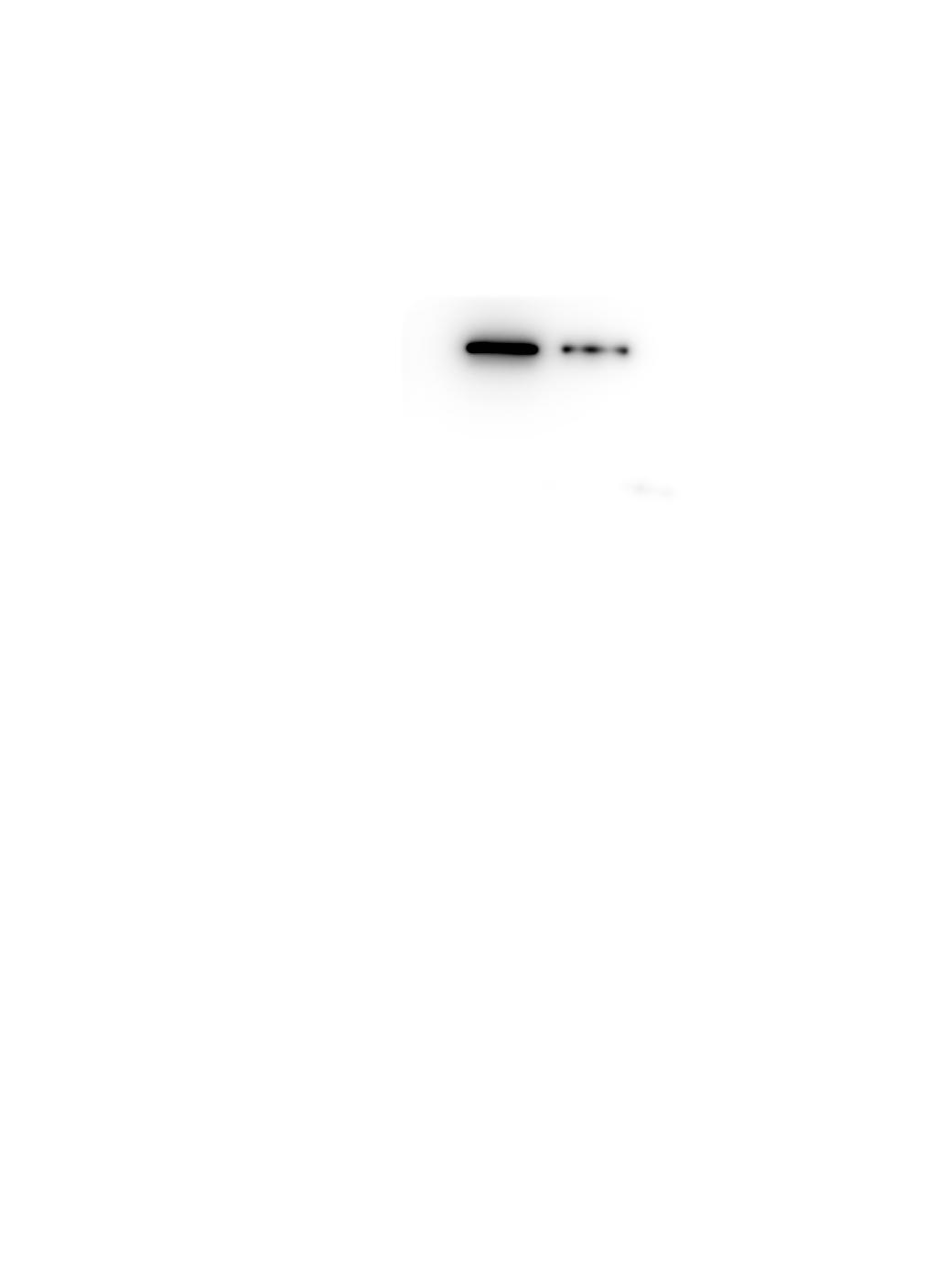

Supplement: Supplementary file 1 [file DataSheet_1.zip › Original data 1/Figure 2H/QBC939/N-cadherin/N-cadherin.jpg]

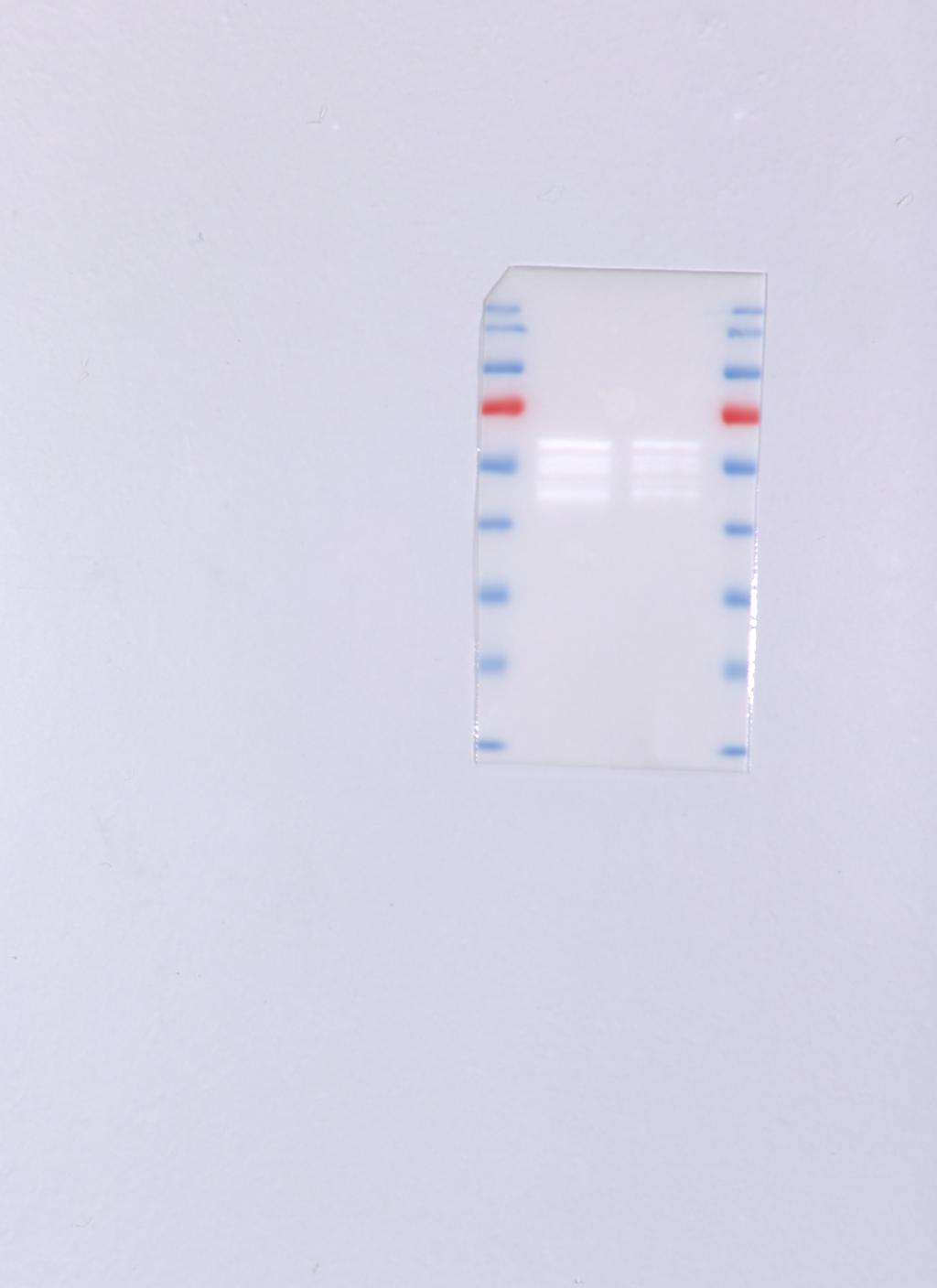

Supplement: Supplementary file 1 [file DataSheet_1.zip › Original data 1/Figure 2H/QBC939/Vimentin M/Vimentin M.jpg]

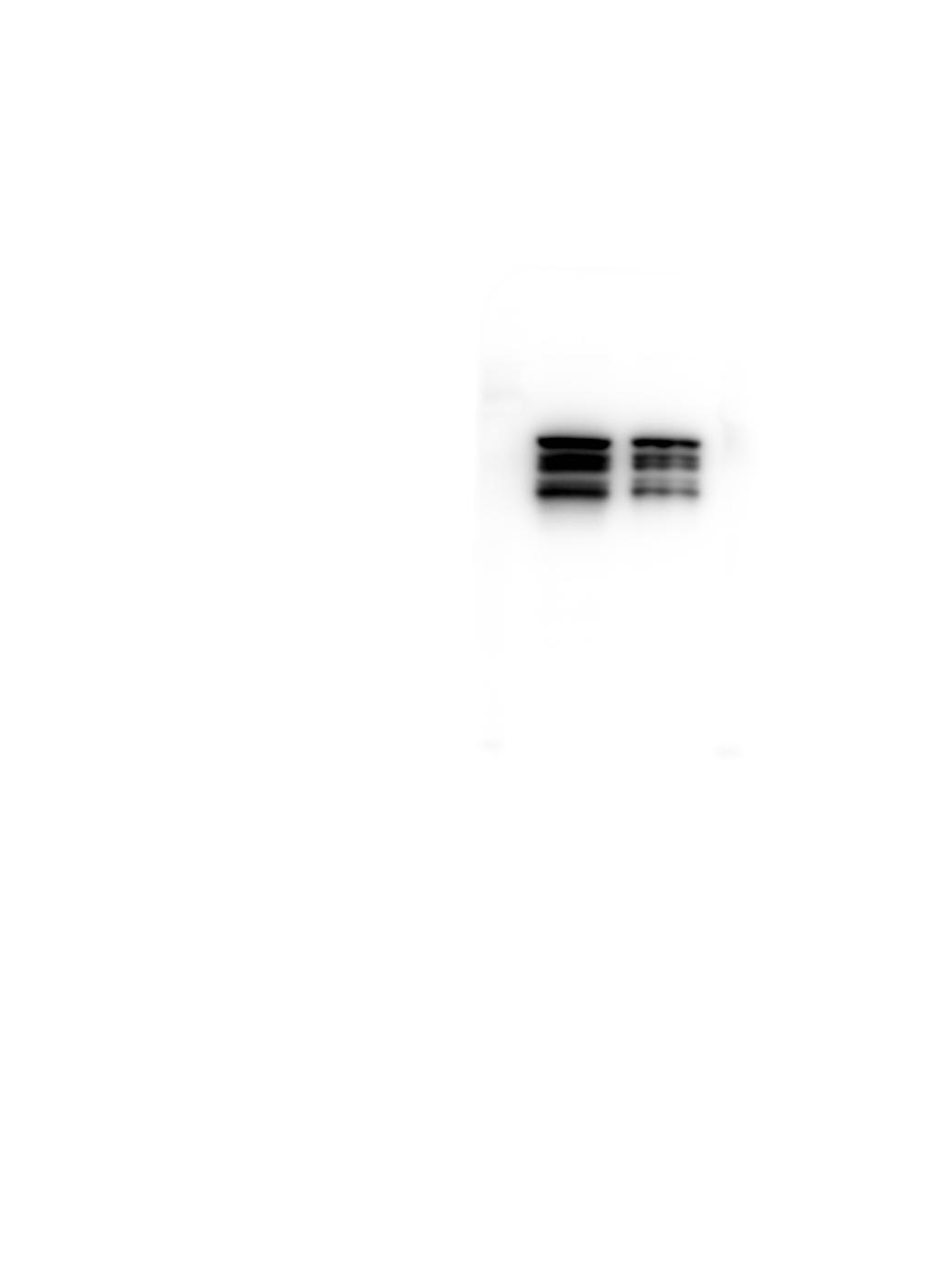

Supplement: Supplementary file 1 [file DataSheet_1.zip › Original data 1/Figure 2H/QBC939/Vimentin/Vimentin.jpg]

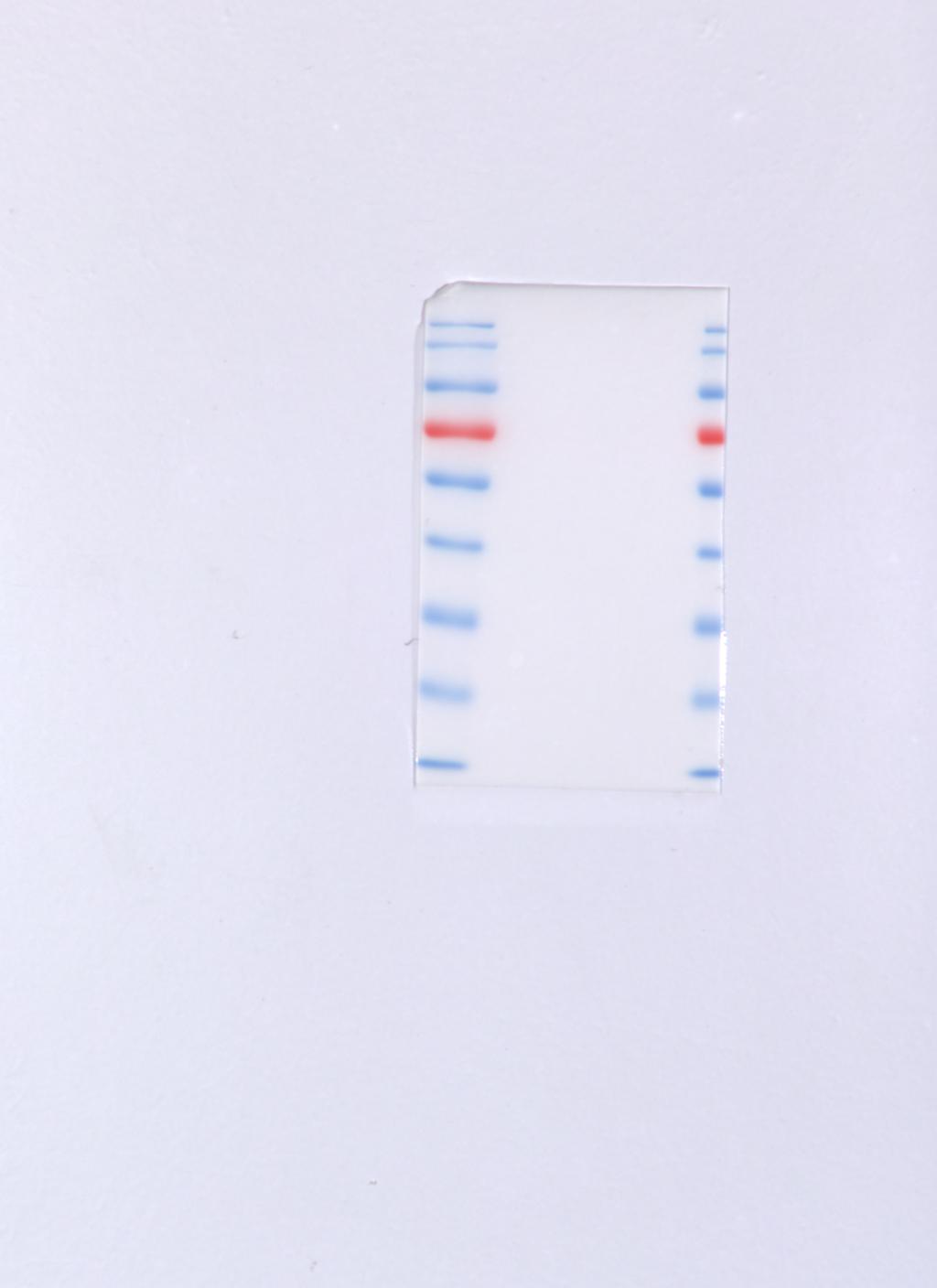

Supplement: Supplementary file 1 [file DataSheet_1.zip › Original data 1/Figure 4D/HCCC-9810/CDK1 M/CDK1 M.jpg]

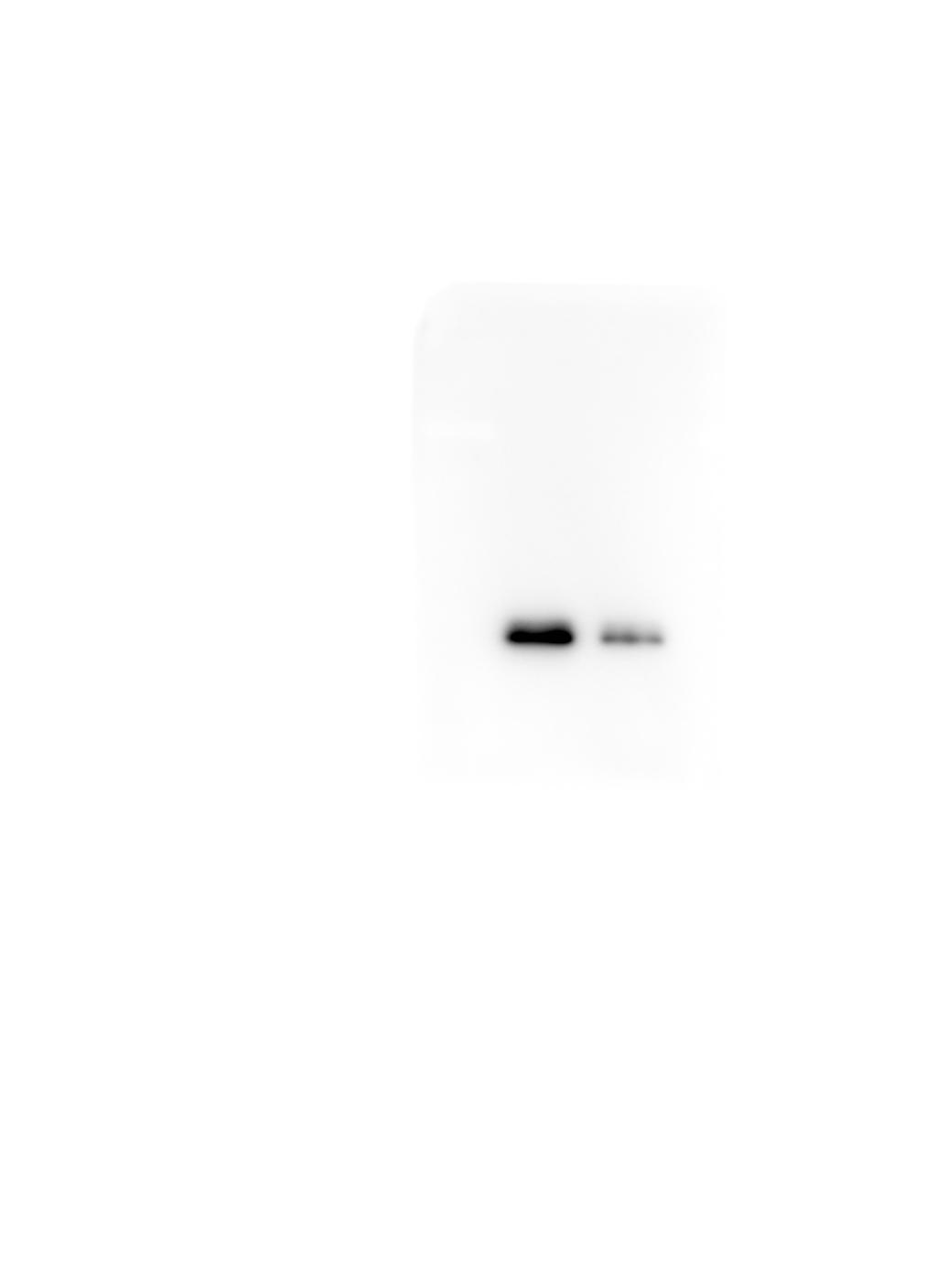

Supplement: Supplementary file 1 [file DataSheet_1.zip › Original data 1/Figure 4D/HCCC-9810/CDK1/CDK1.jpg]

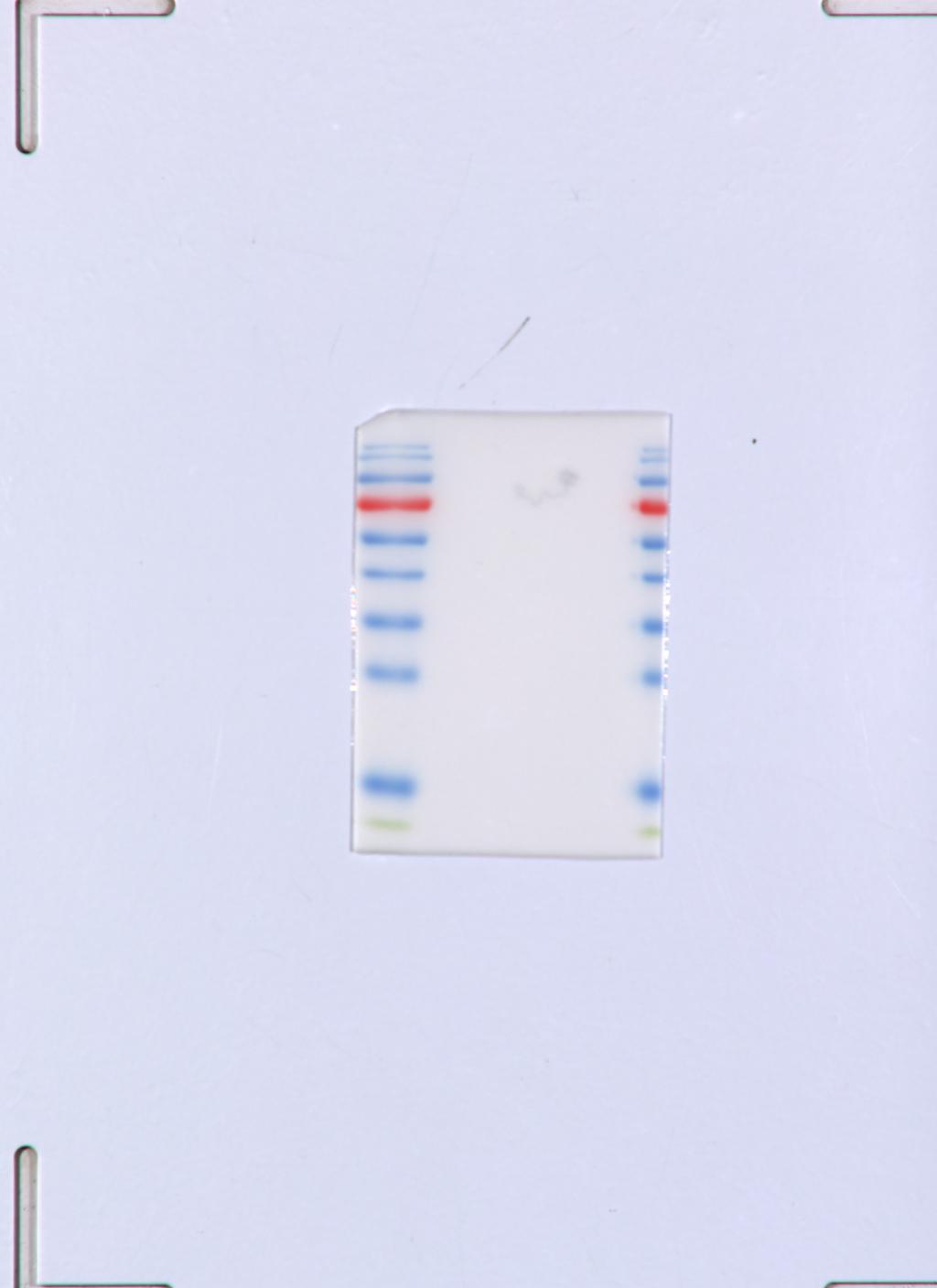

Supplement: Supplementary file 1 [file DataSheet_1.zip › Original data 1/Figure 4D/HCCC-9810/Cyclin E2 M/Cyclin E2 M.jpg]

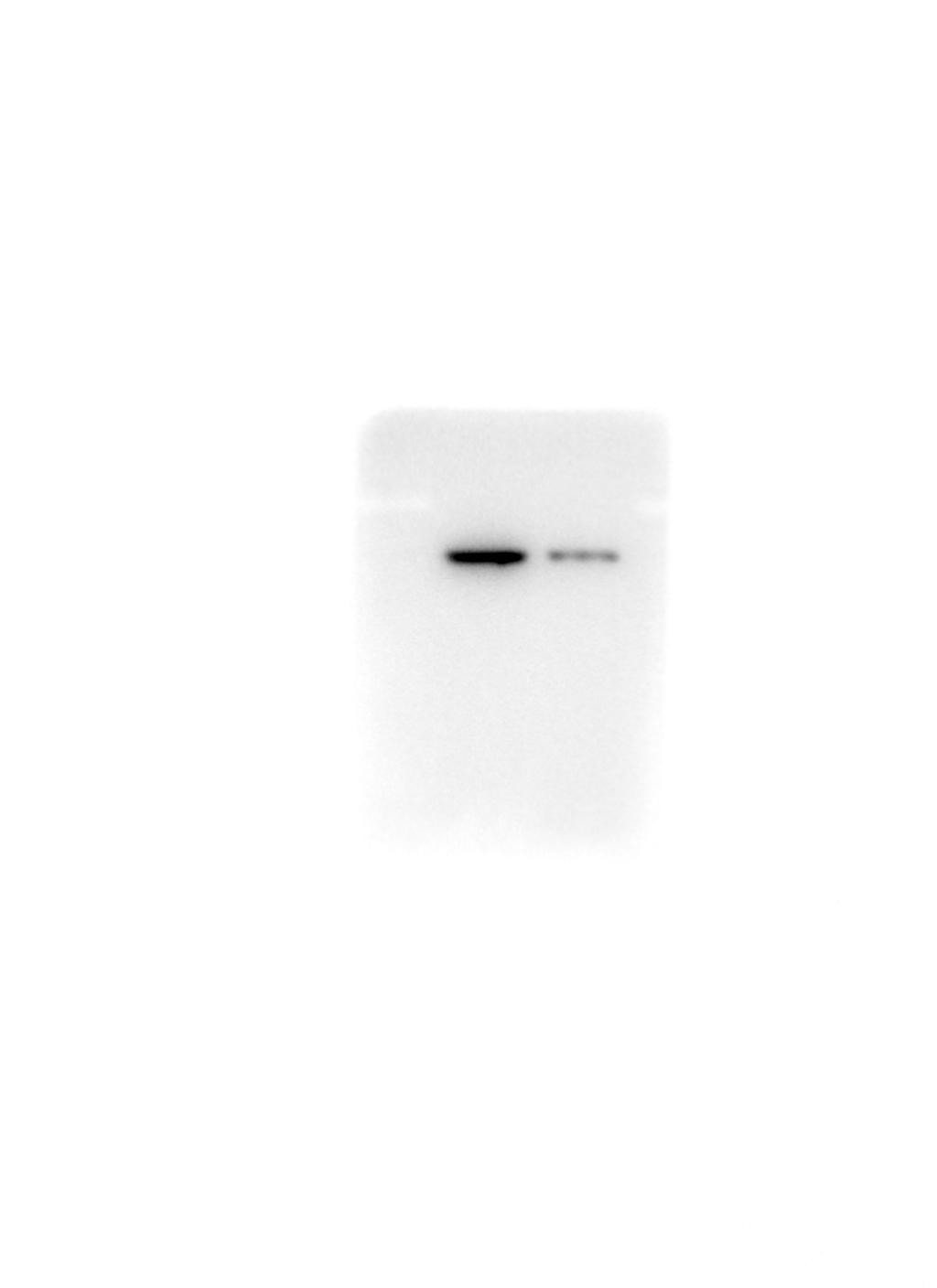

Supplement: Supplementary file 1 [file DataSheet_1.zip › Original data 1/Figure 4D/HCCC-9810/Cyclin E2/Cyclin E2.jpg]

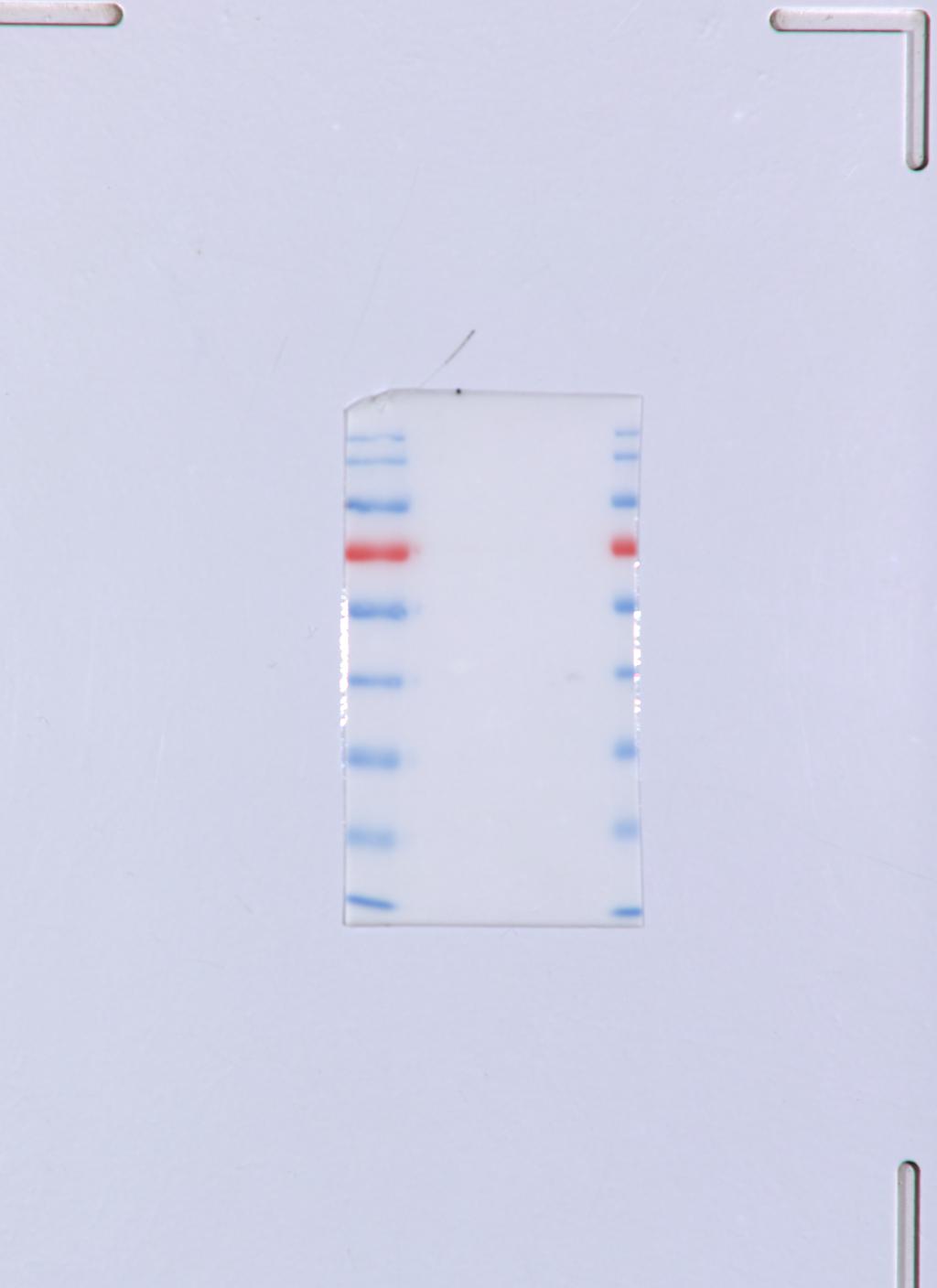

Supplement: Supplementary file 1 [file DataSheet_1.zip › Original data 1/Figure 4D/HCCC-9810/E2F1 M/E2F1 M.jpg]

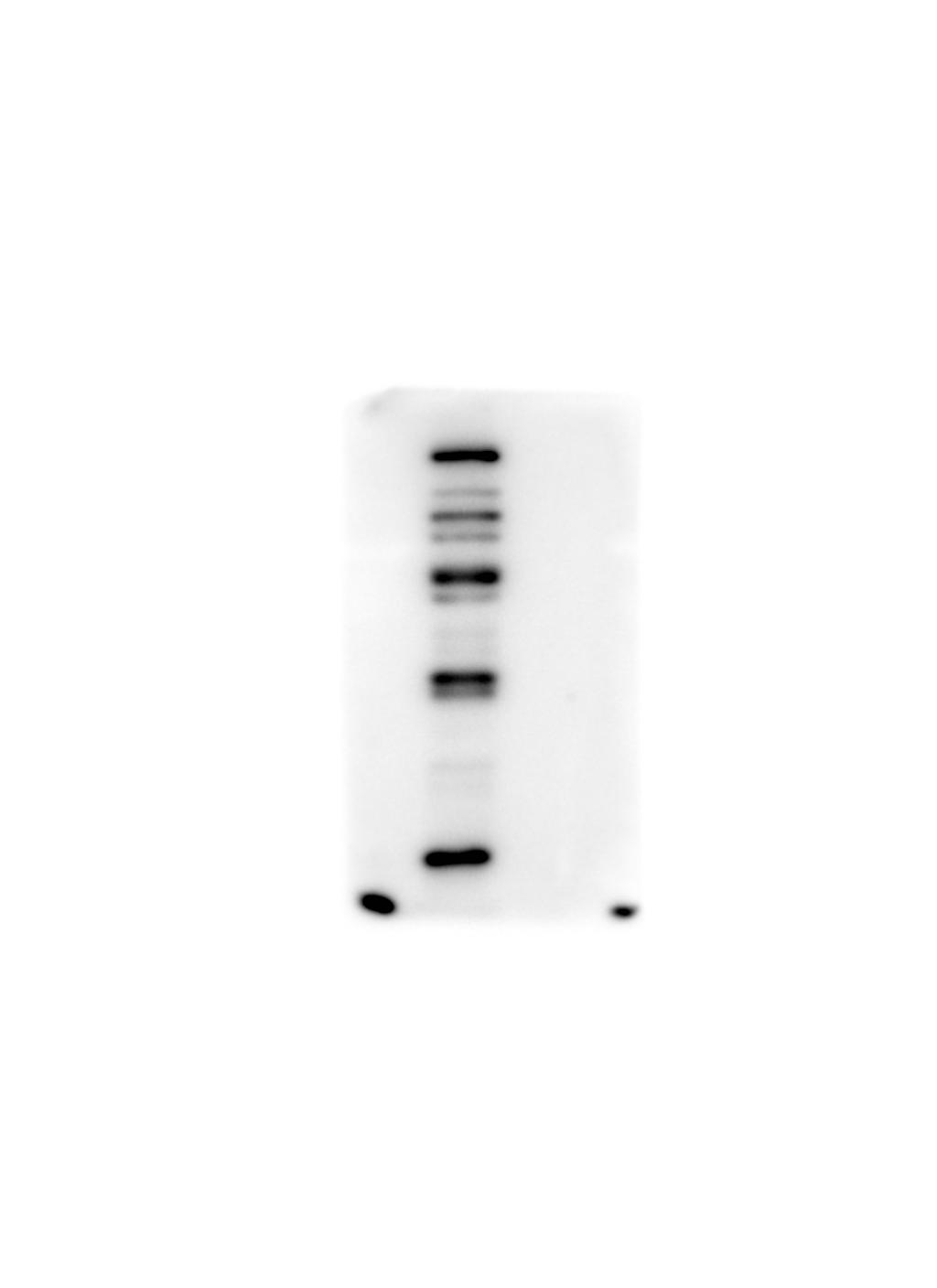

Supplement: Supplementary file 1 [file DataSheet_1.zip › Original data 1/Figure 4D/HCCC-9810/E2F1/E2F1.jpg]

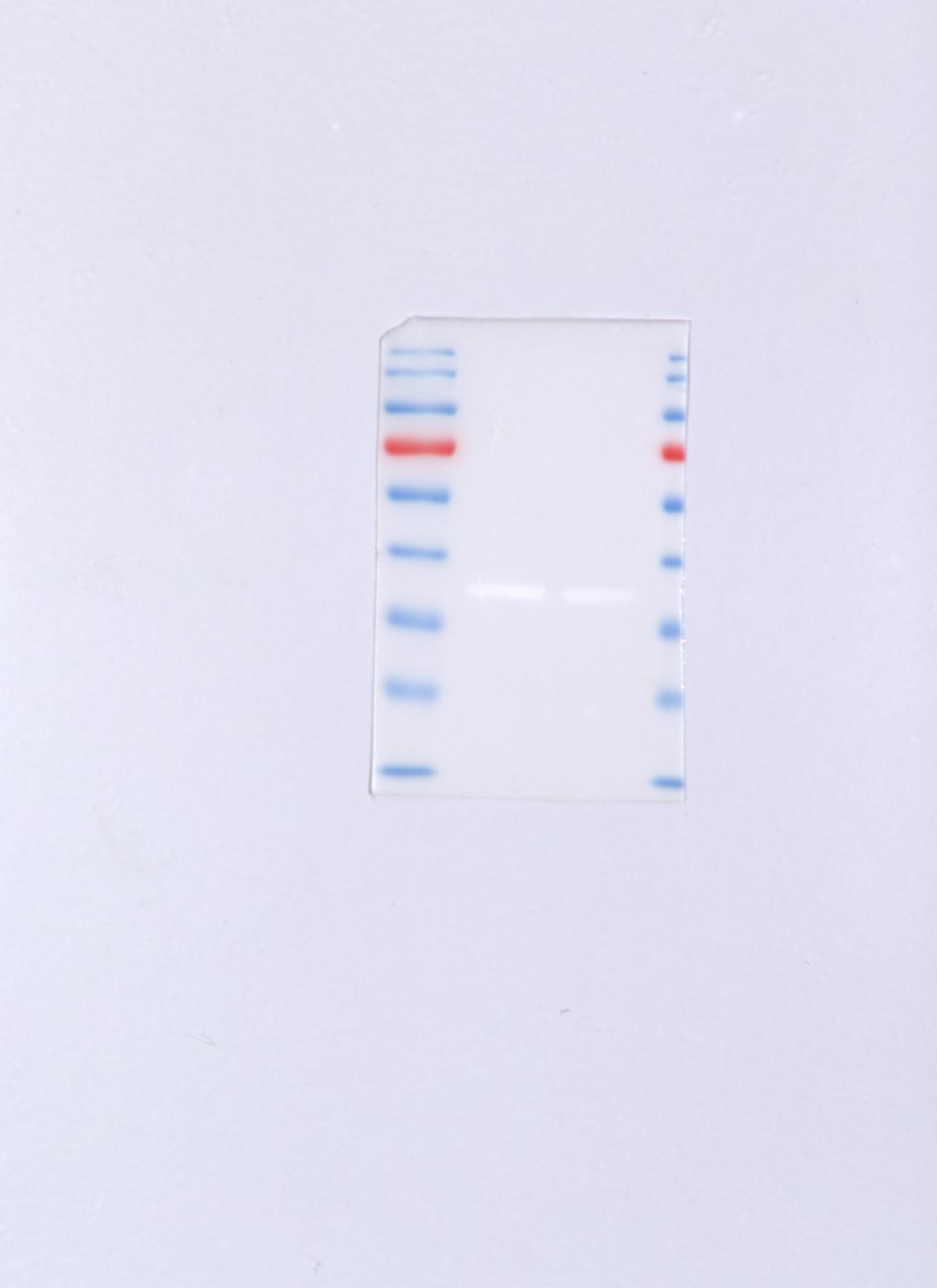

Supplement: Supplementary file 1 [file DataSheet_1.zip › Original data 1/Figure 4D/HCCC-9810/GAPDH M/GAPDH M.jpg]

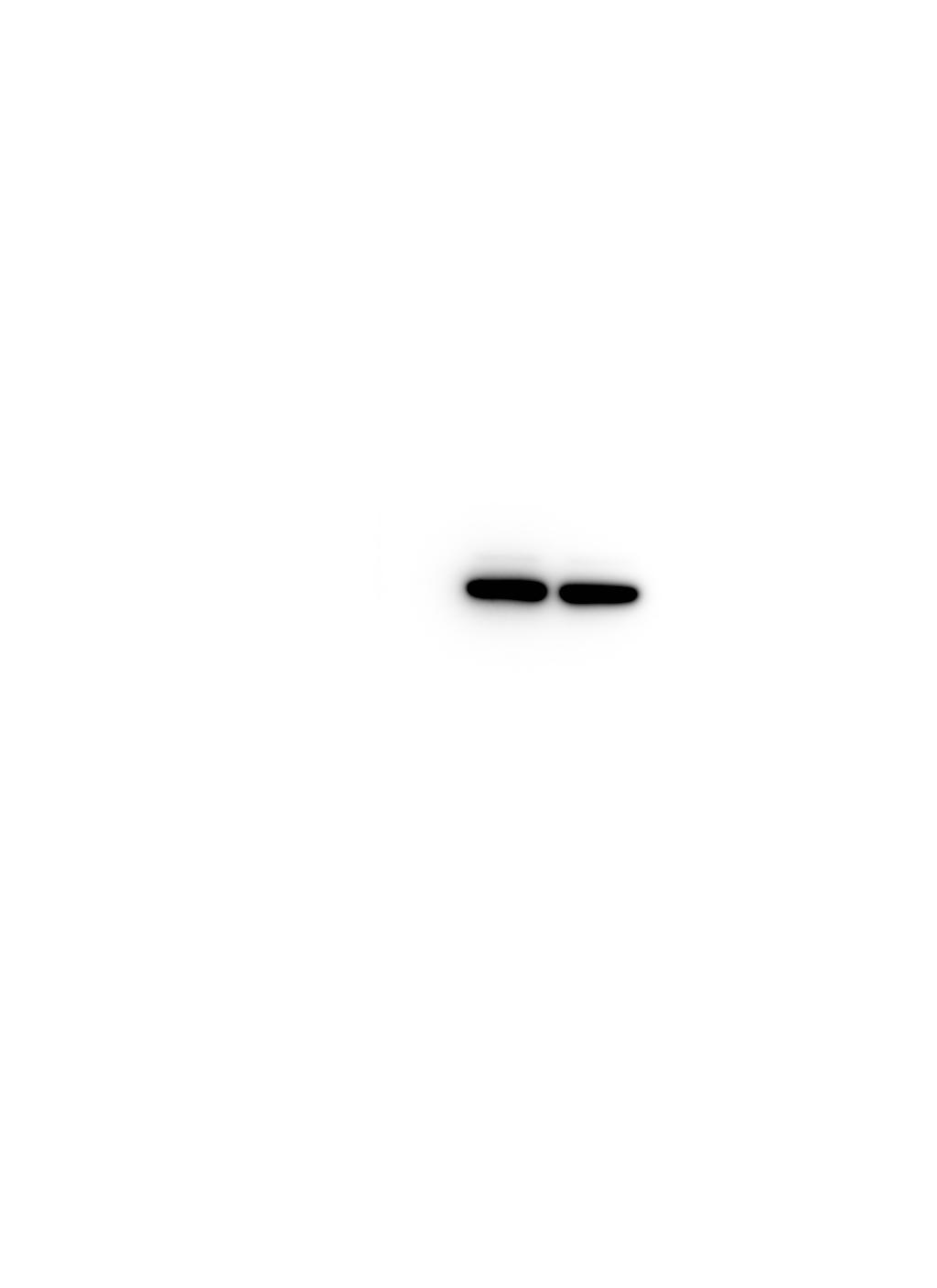

Supplement: Supplementary file 1 [file DataSheet_1.zip › Original data 1/Figure 4D/HCCC-9810/GAPDH/GAPDH.jpg]

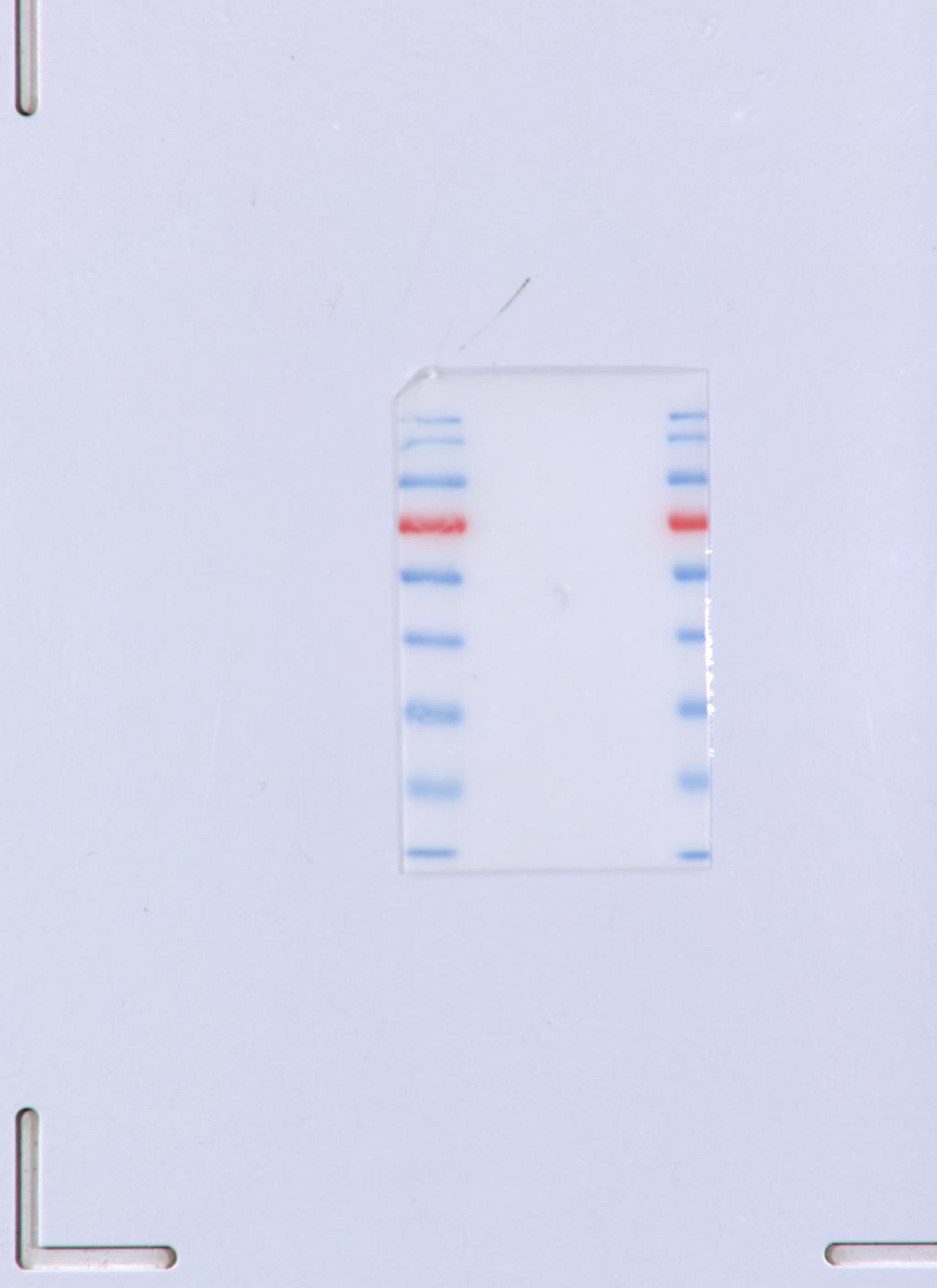

Supplement: Supplementary file 1 [file DataSheet_1.zip › Original data 1/Figure 4D/HCCC-9810/NEK2 M/NEK2 M.jpg]

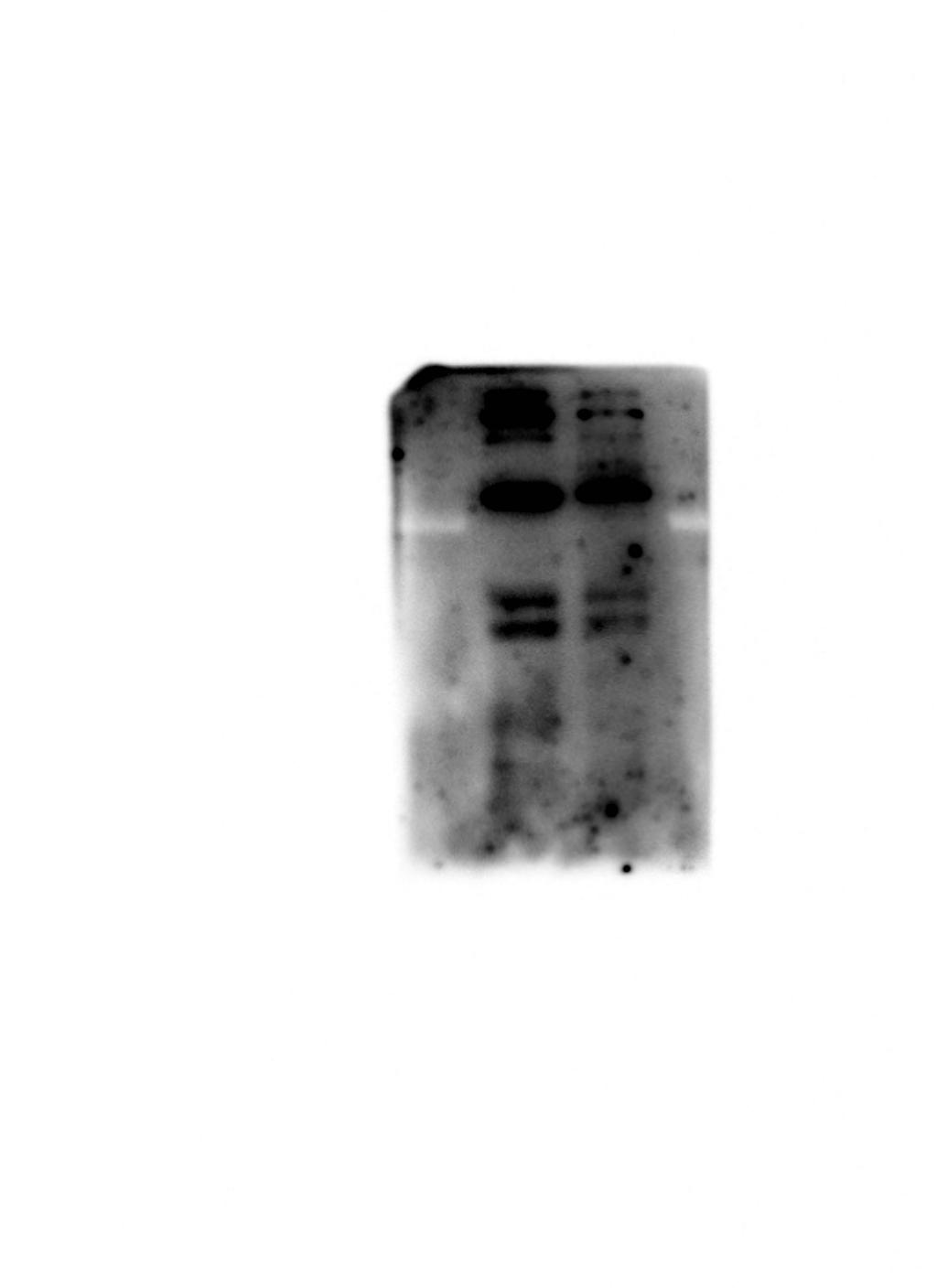

Supplement: Supplementary file 1 [file DataSheet_1.zip › Original data 1/Figure 4D/HCCC-9810/NEK2/NEK2.jpg]

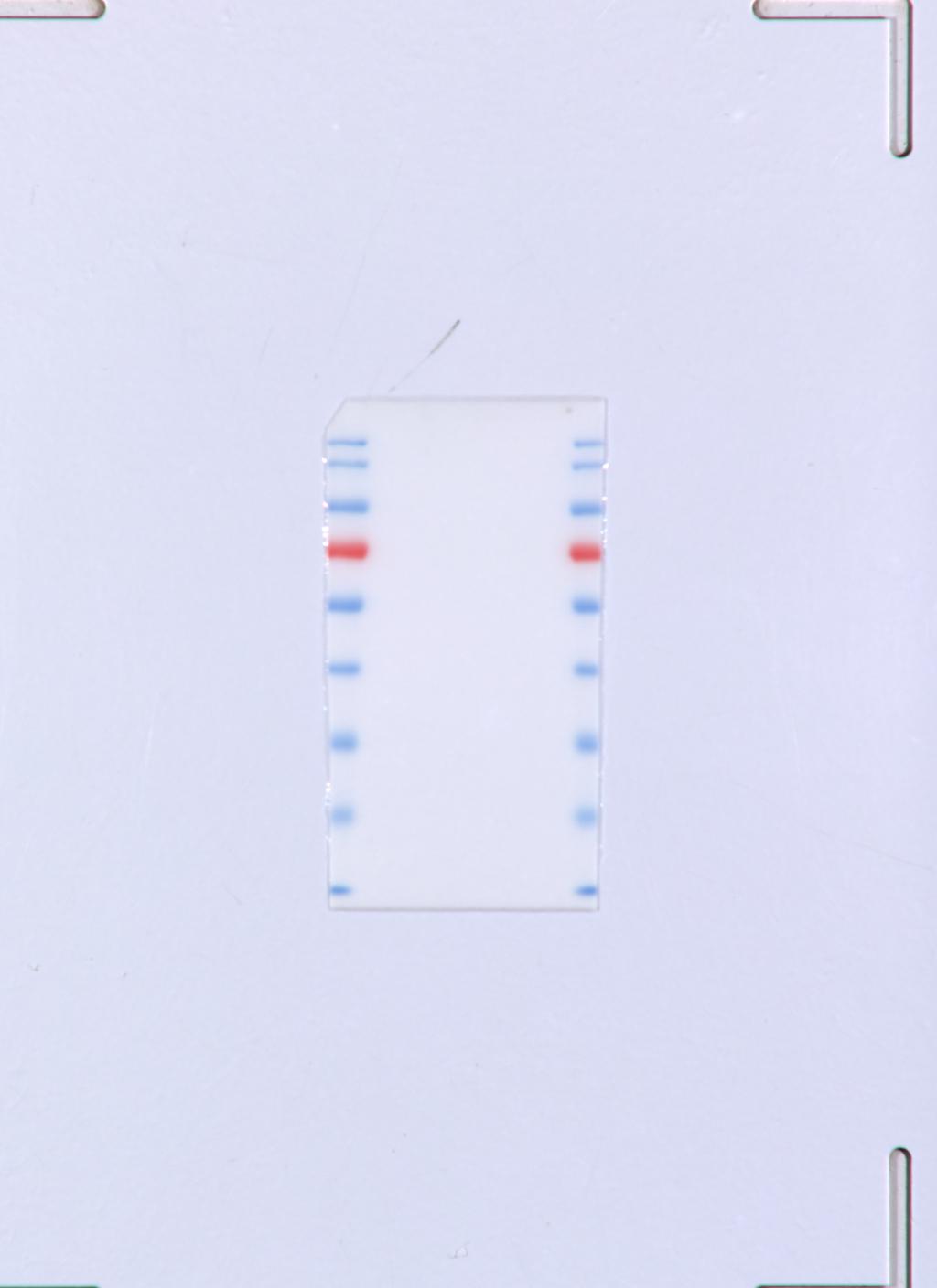

Supplement: Supplementary file 1 [file DataSheet_1.zip › Original data 1/Figure 4D/QBC939/CDK1 M/CDK1 M.jpg]

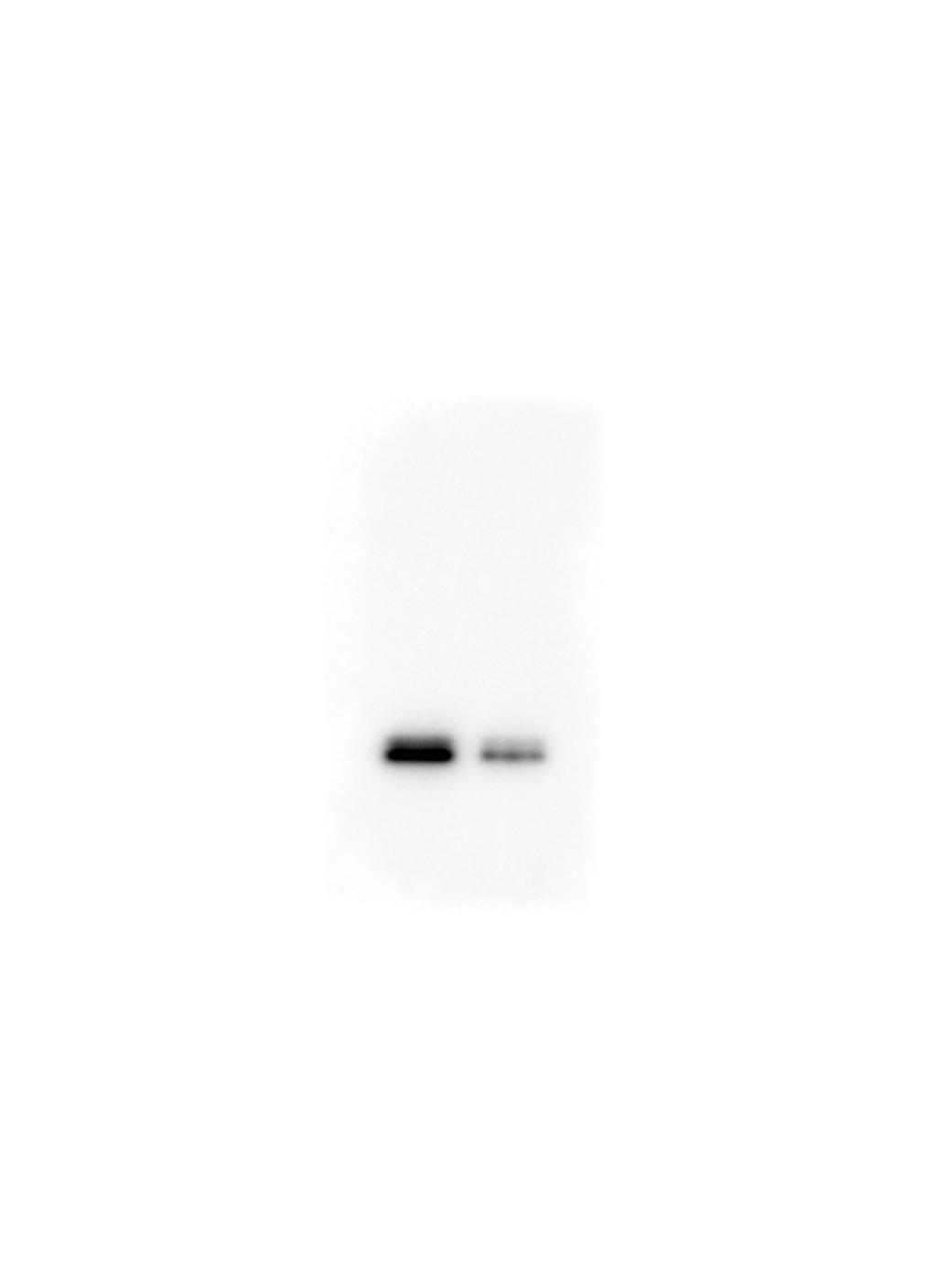

Supplement: Supplementary file 1 [file DataSheet_1.zip › Original data 1/Figure 4D/QBC939/CDK1/CDK1.jpg]

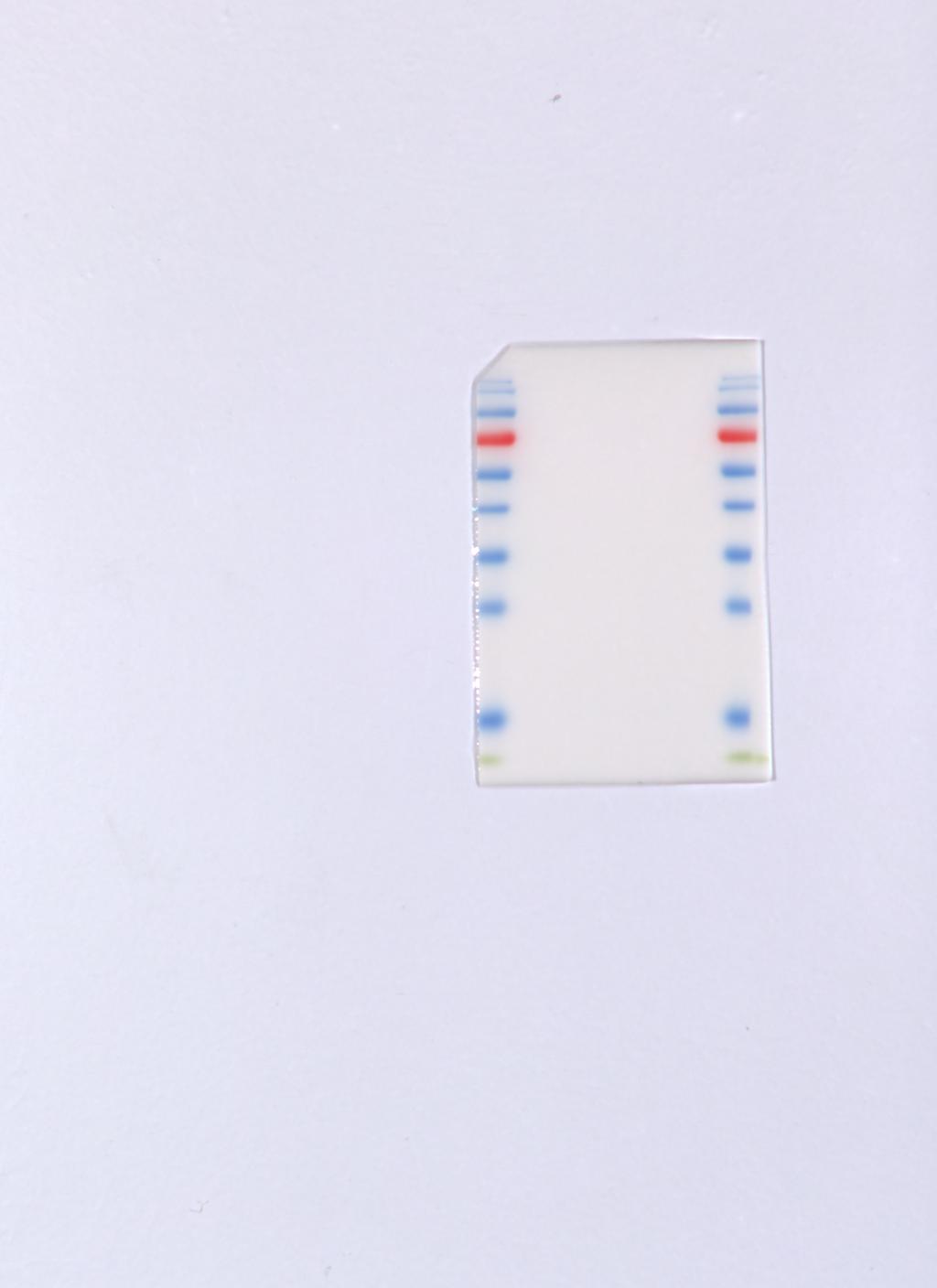

Supplement: Supplementary file 1 [file DataSheet_1.zip › Original data 1/Figure 4D/QBC939/Cyclin E2 M/Cyclin E2 M.jpg]

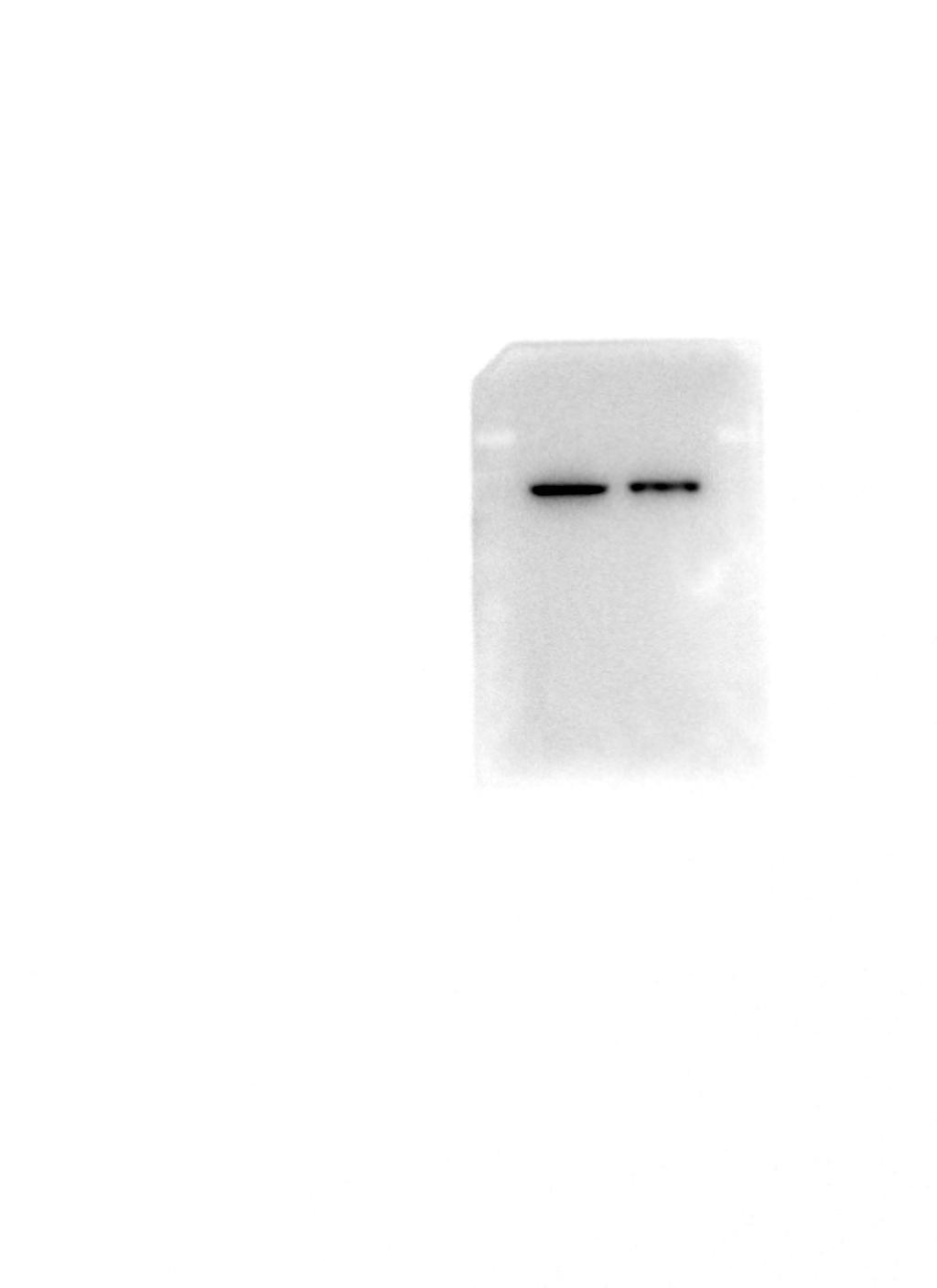

Supplement: Supplementary file 1 [file DataSheet_1.zip › Original data 1/Figure 4D/QBC939/Cyclin E2/Cyclin E2.jpg]

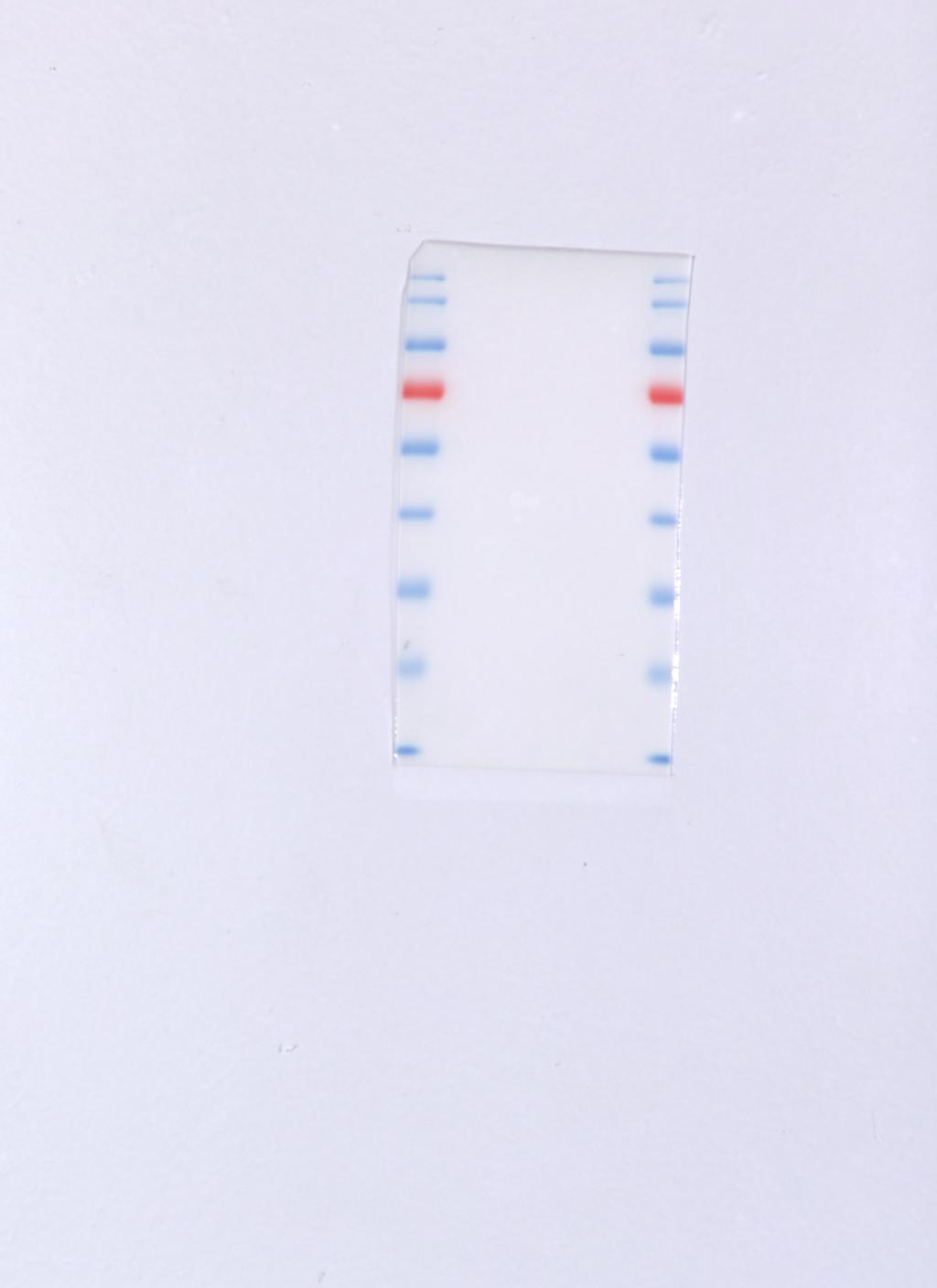

Supplement: Supplementary file 1 [file DataSheet_1.zip › Original data 1/Figure 4D/QBC939/E2F1 M/E2F1 M.jpg]

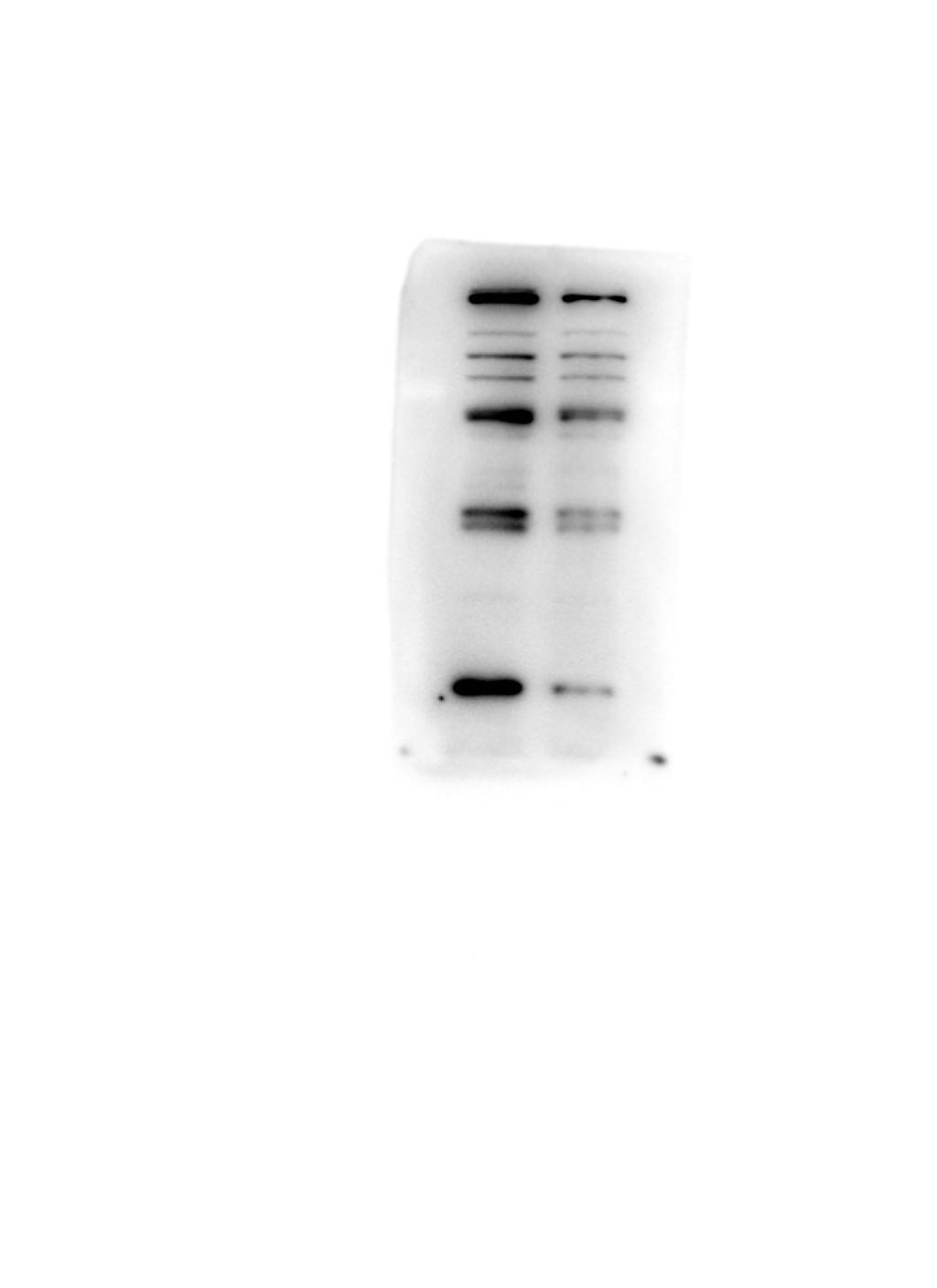

Supplement: Supplementary file 1 [file DataSheet_1.zip › Original data 1/Figure 4D/QBC939/E2F1/E2F1.jpg]

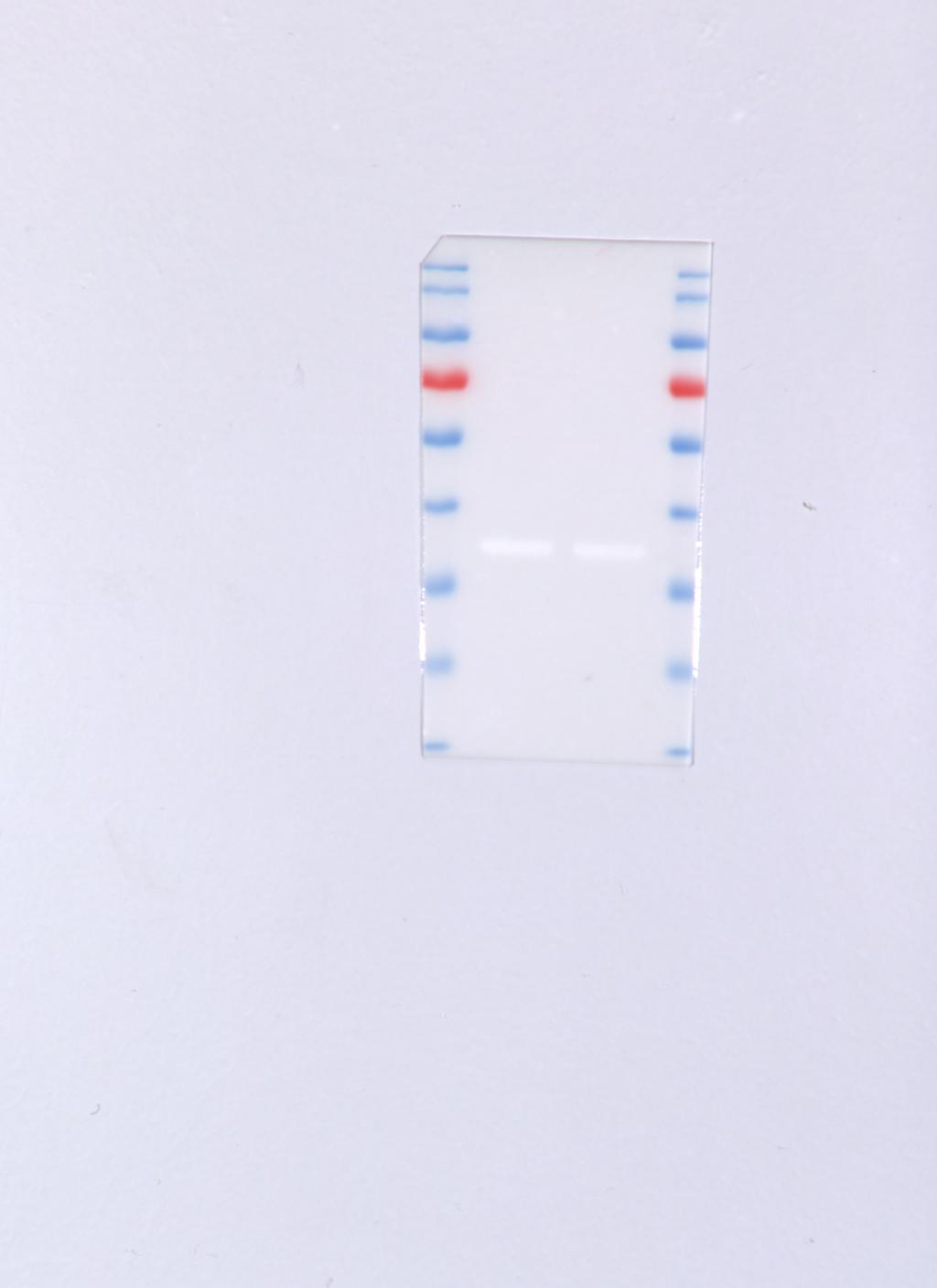

Supplement: Supplementary file 1 [file DataSheet_1.zip › Original data 1/Figure 4D/QBC939/GAPDH M/GAPDH M.jpg]

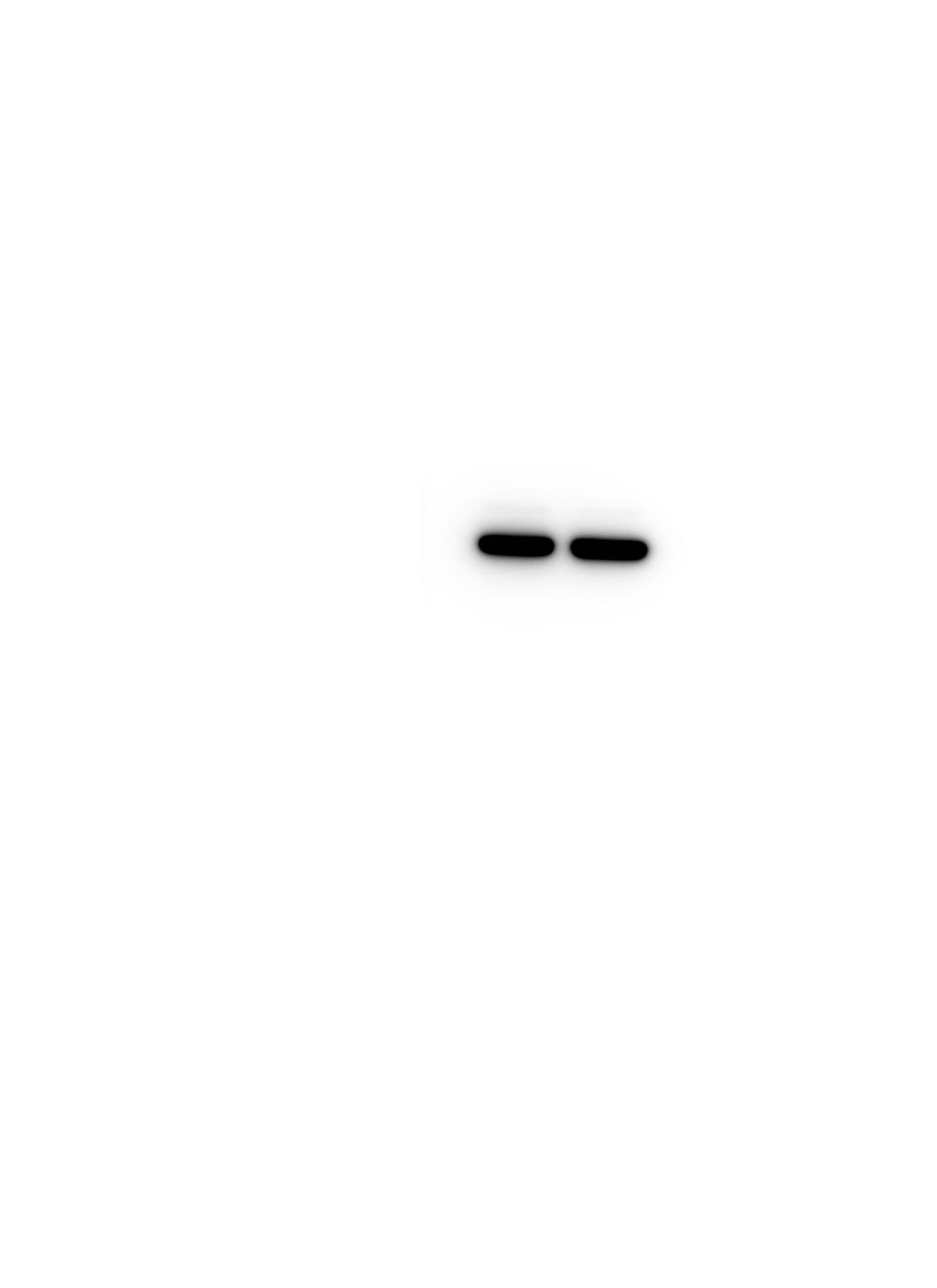

Supplement: Supplementary file 1 [file DataSheet_1.zip › Original data 1/Figure 4D/QBC939/GAPDH/GAPDH.jpg]

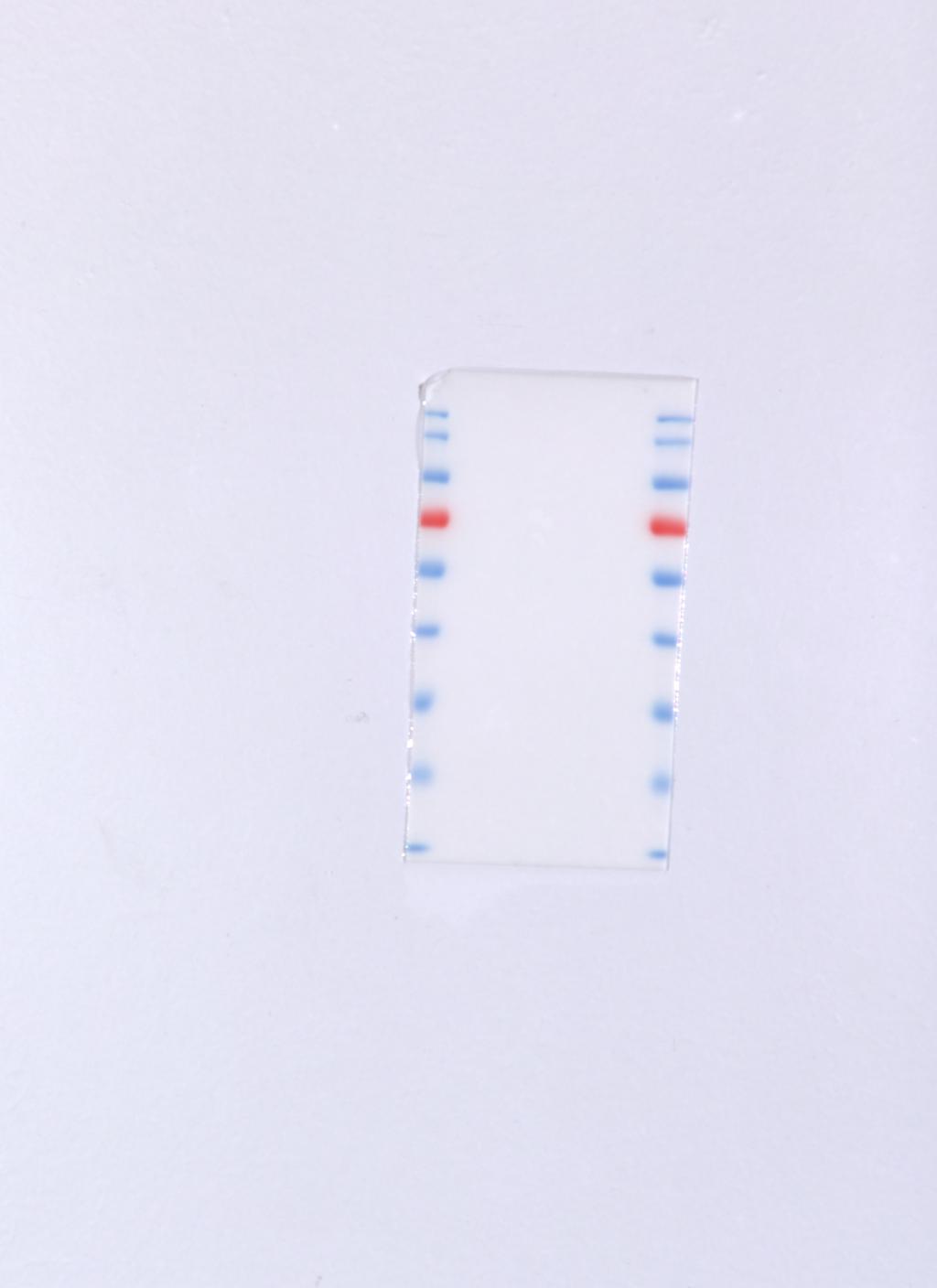

Supplement: Supplementary file 1 [file DataSheet_1.zip › Original data 1/Figure 4D/QBC939/NEK2 M/NEK2 M.jpg]

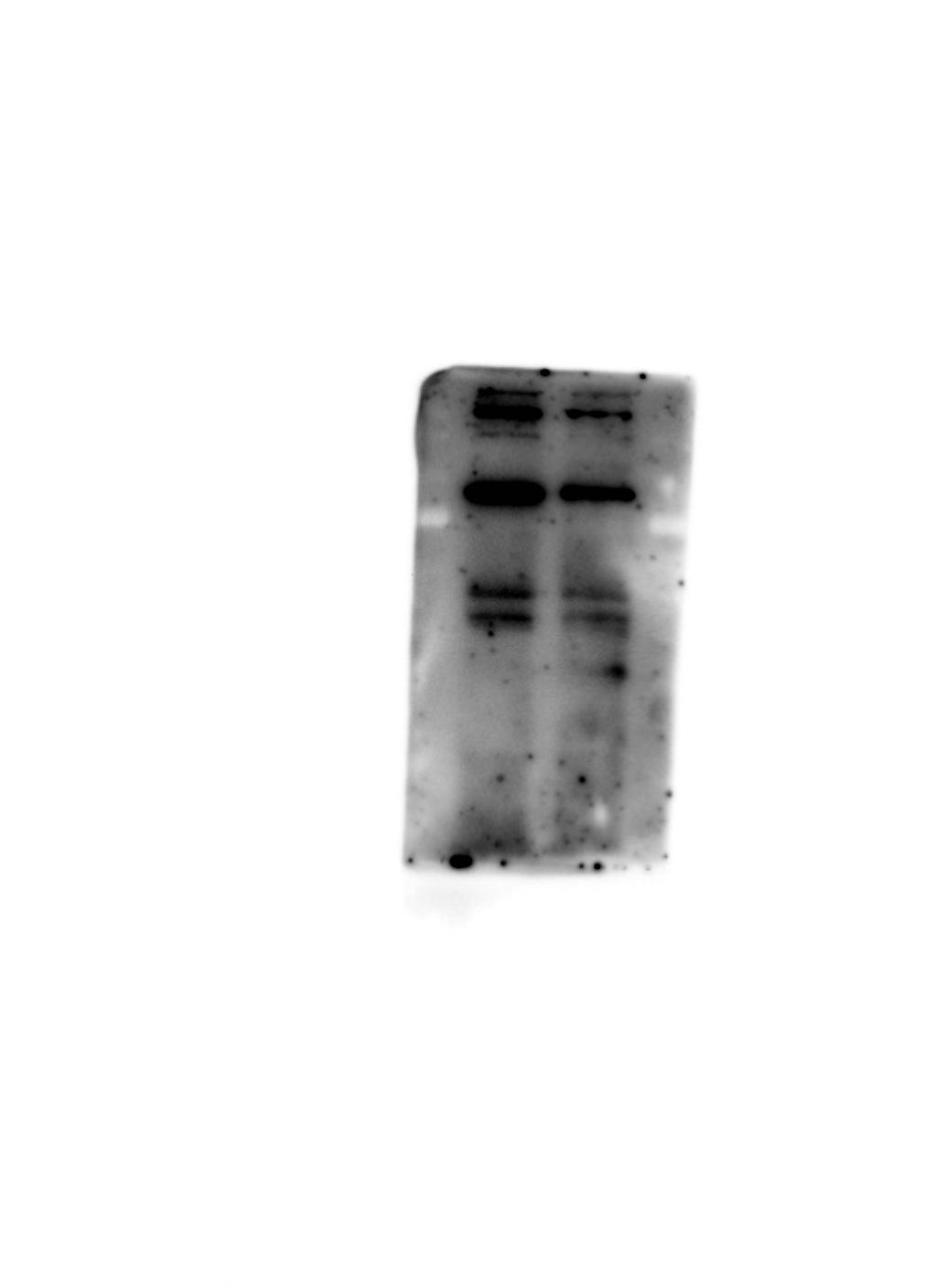

Supplement: Supplementary file 1 [file DataSheet_1.zip › Original data 1/Figure 4D/QBC939/NEK2/NEK2.jpg]

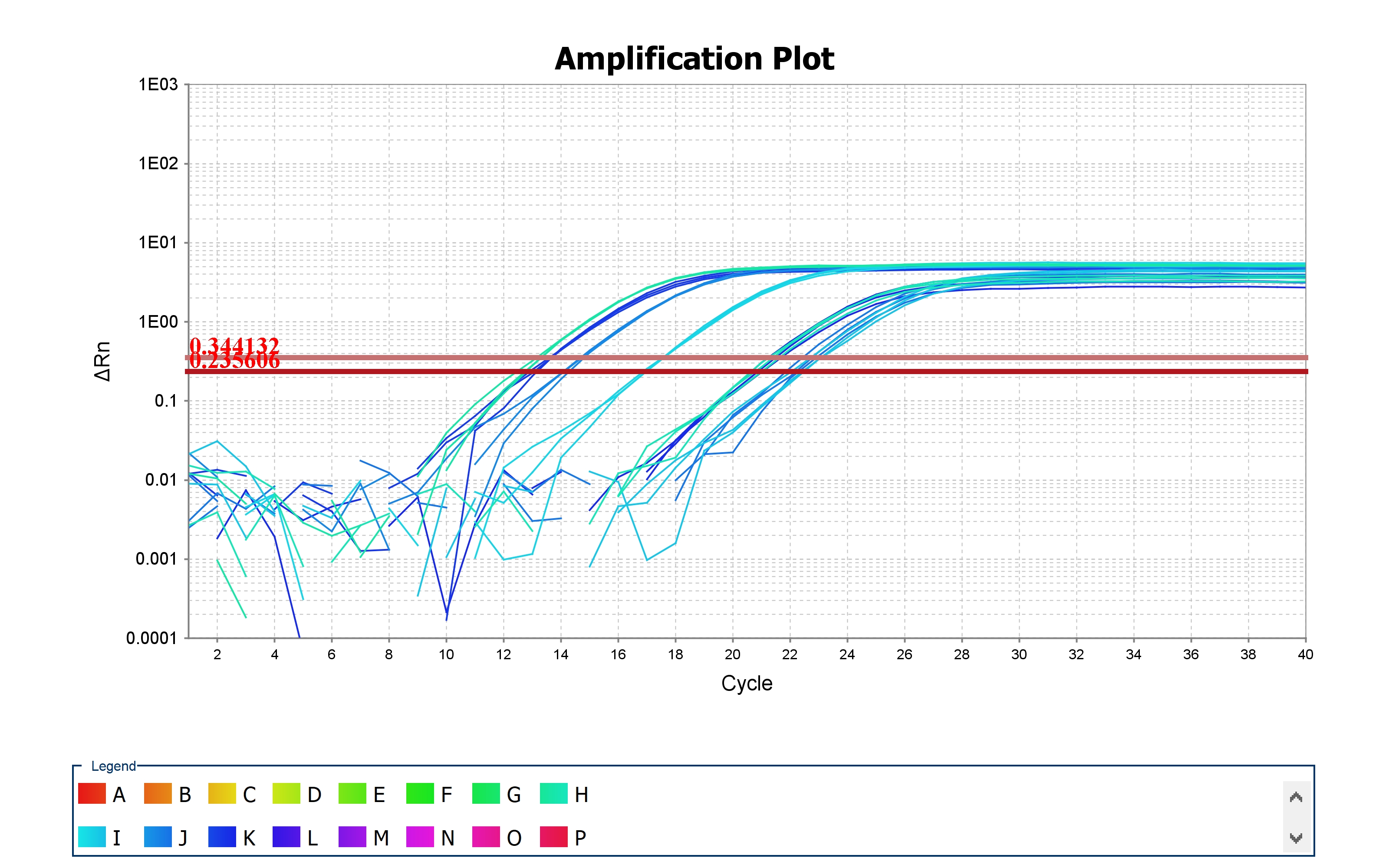

Supplement: Supplementary file 1 [file DataSheet_1.zip › Original data 1/Figure 4G/Amplification Plot.jpg]

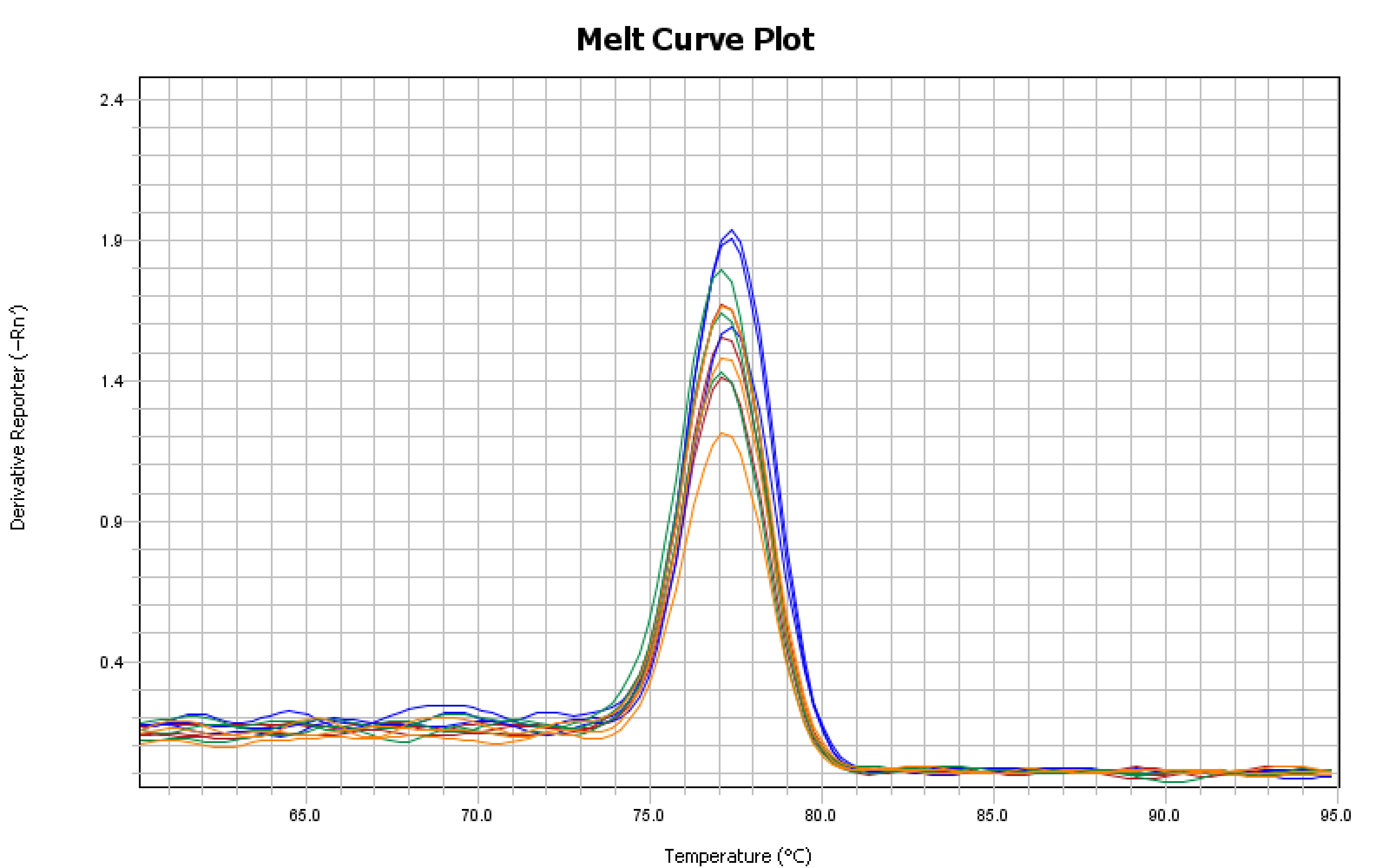

Supplement: Supplementary file 1 [file DataSheet_1.zip › Original data 1/Figure 4G/Melt Curve Plot H-CDK1.jpg]

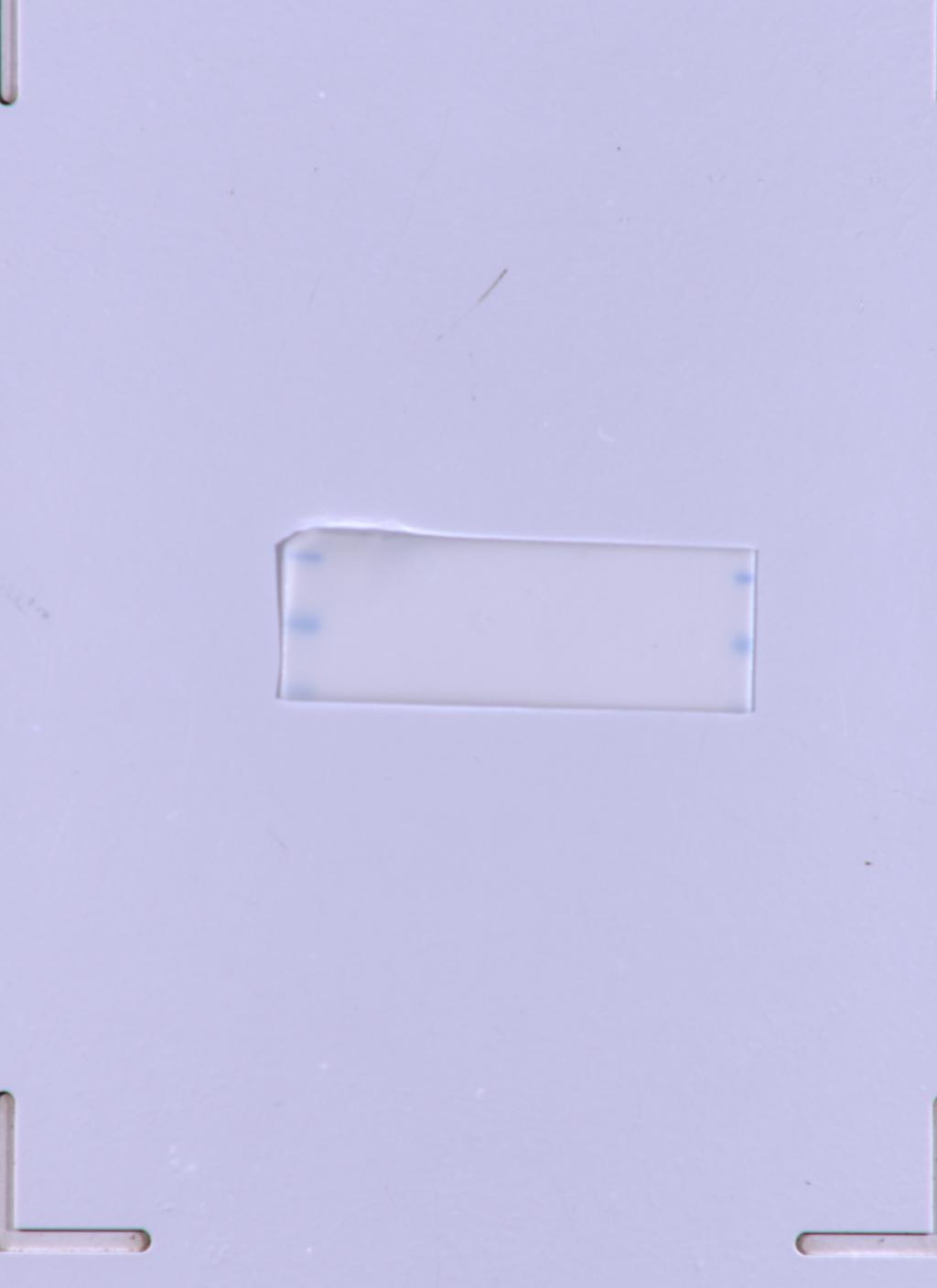

Supplement: Supplementary file 1 [file DataSheet_1.zip › Original data 1/Figure 5A/CDK1-1 M/CDK1-1 M.jpg]

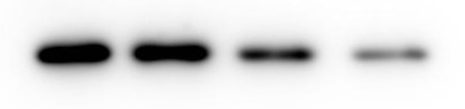

Supplement: Supplementary file 1 [file DataSheet_1.zip › Original data 1/Figure 5A/CDK1-1/CDK1-1.jpg]

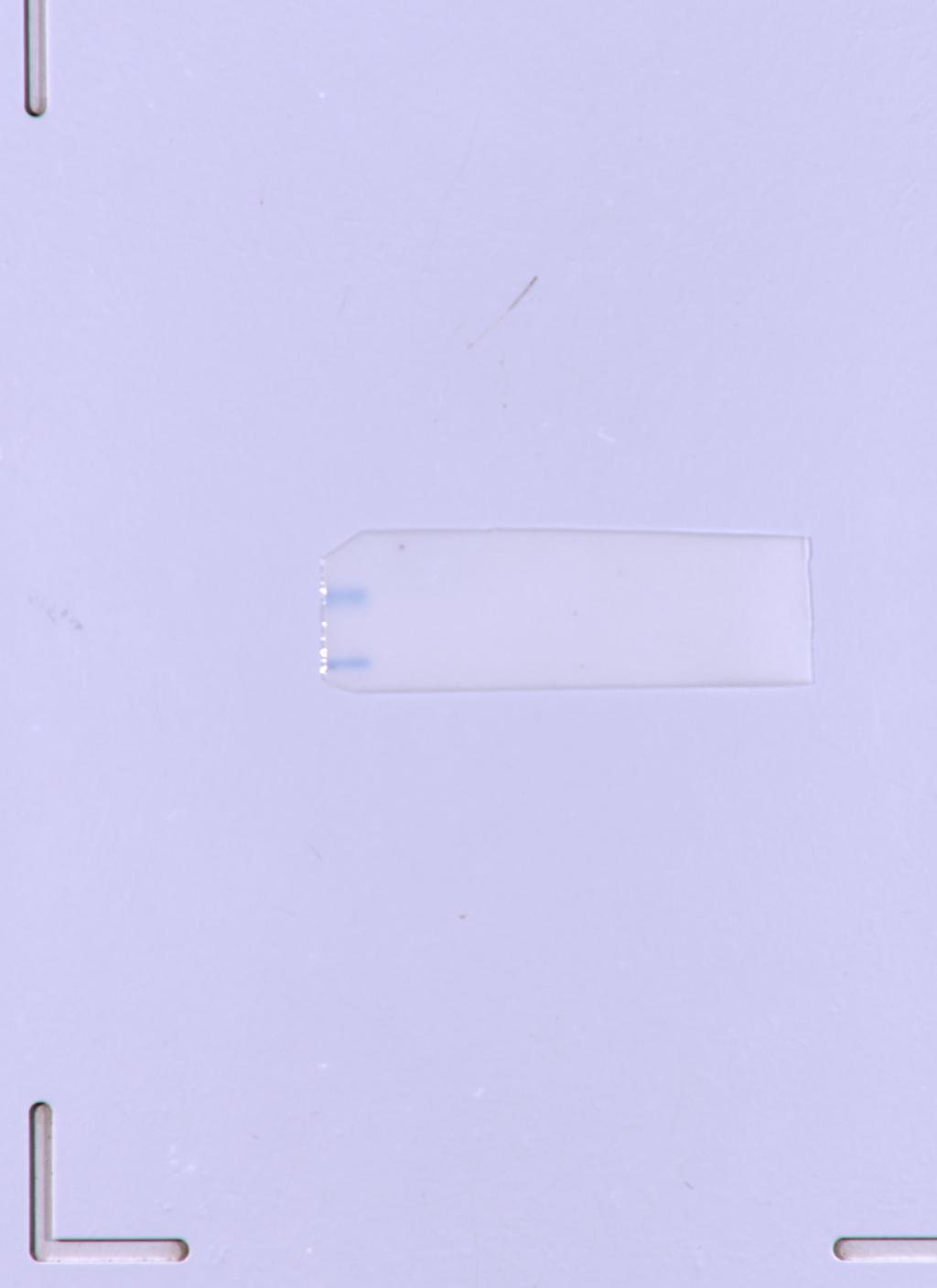

Supplement: Supplementary file 1 [file DataSheet_1.zip › Original data 1/Figure 5A/CDK1-2 M/CDK1-2 M.jpg]

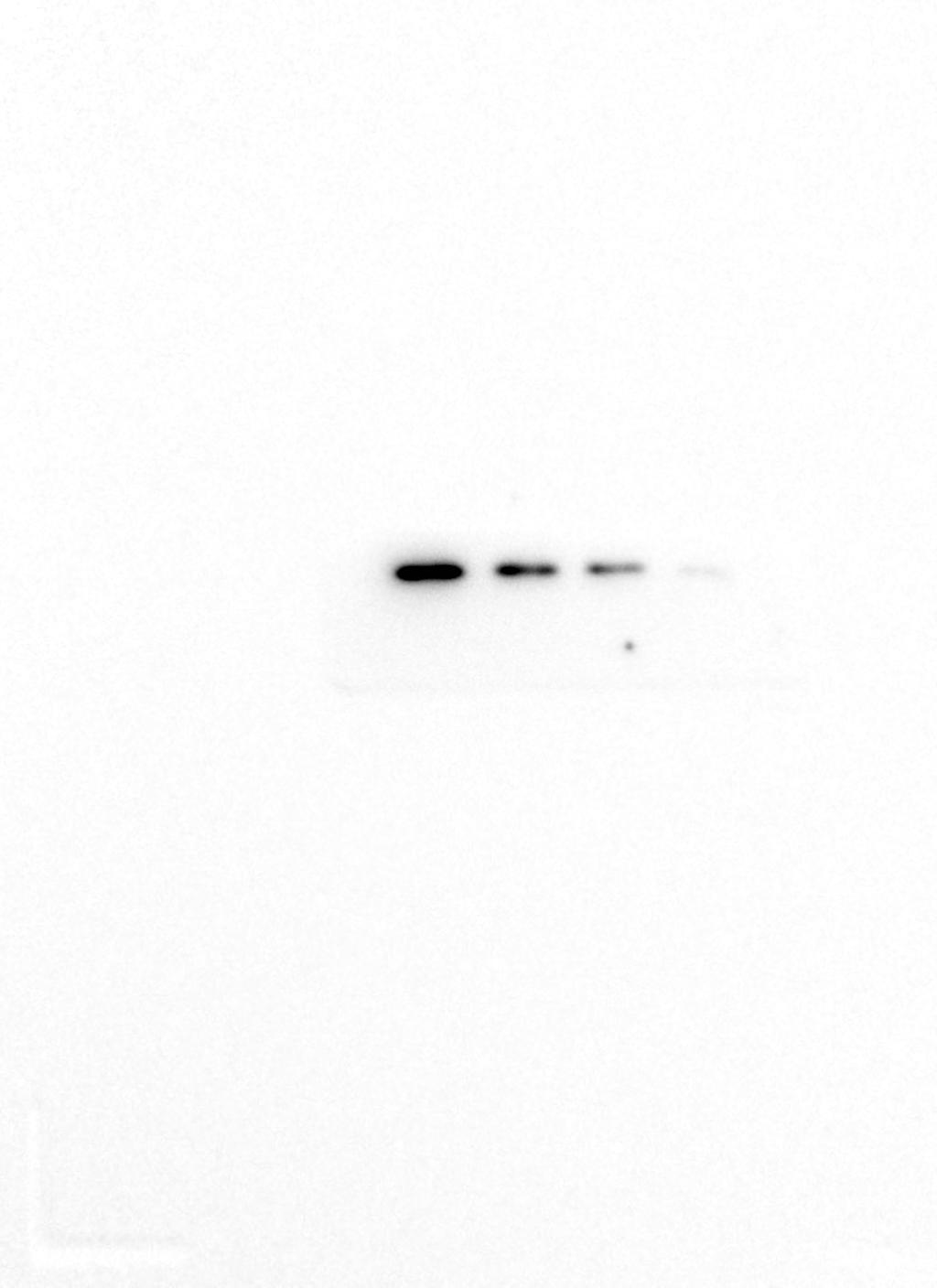

Supplement: Supplementary file 1 [file DataSheet_1.zip › Original data 1/Figure 5A/CDK1-2/CDK1-2.jpg]

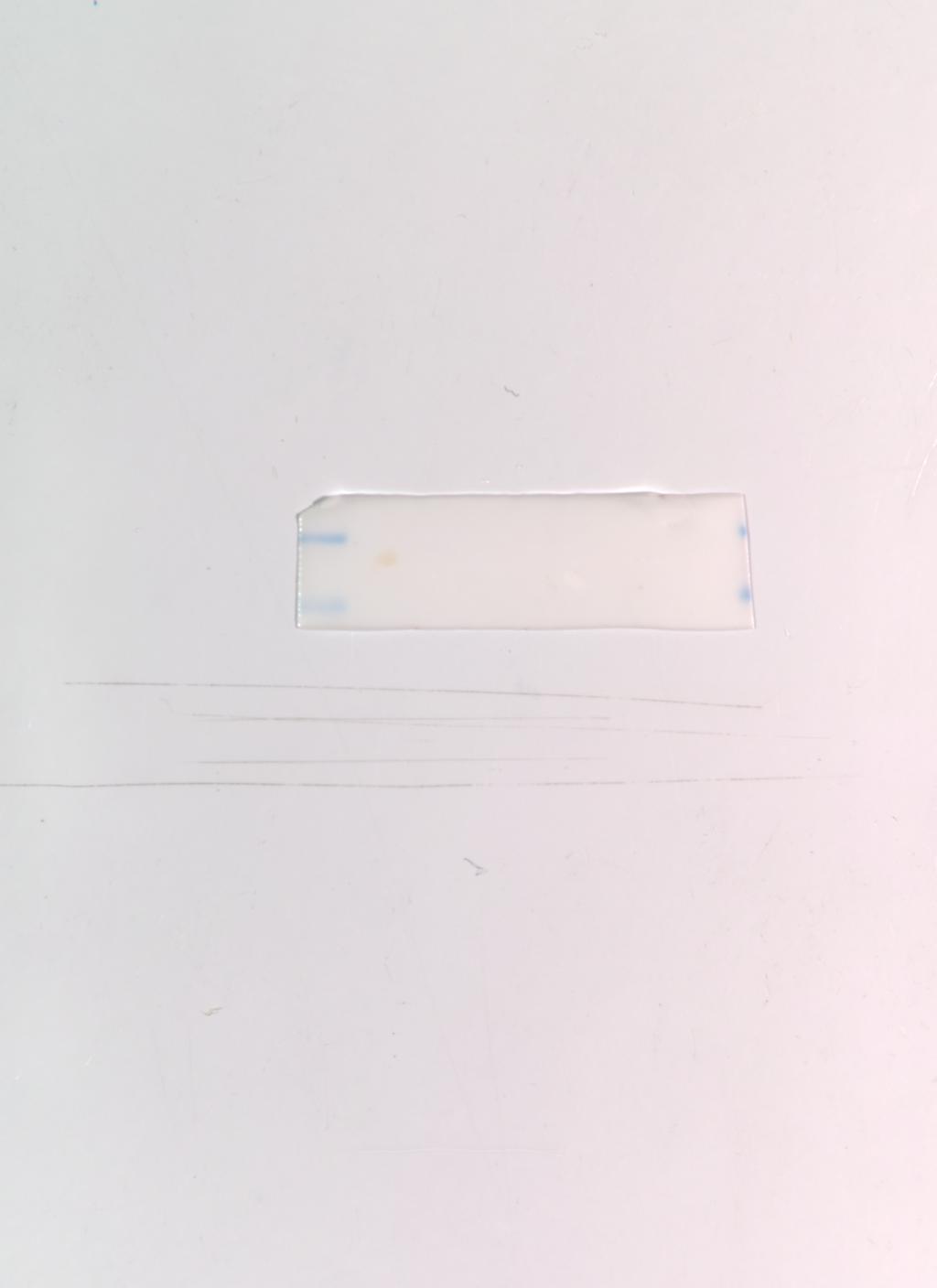

Supplement: Supplementary file 1 [file DataSheet_1.zip › Original data 1/Figure 5A/GAPDH-1 M/GAPDH-1 M.jpg]

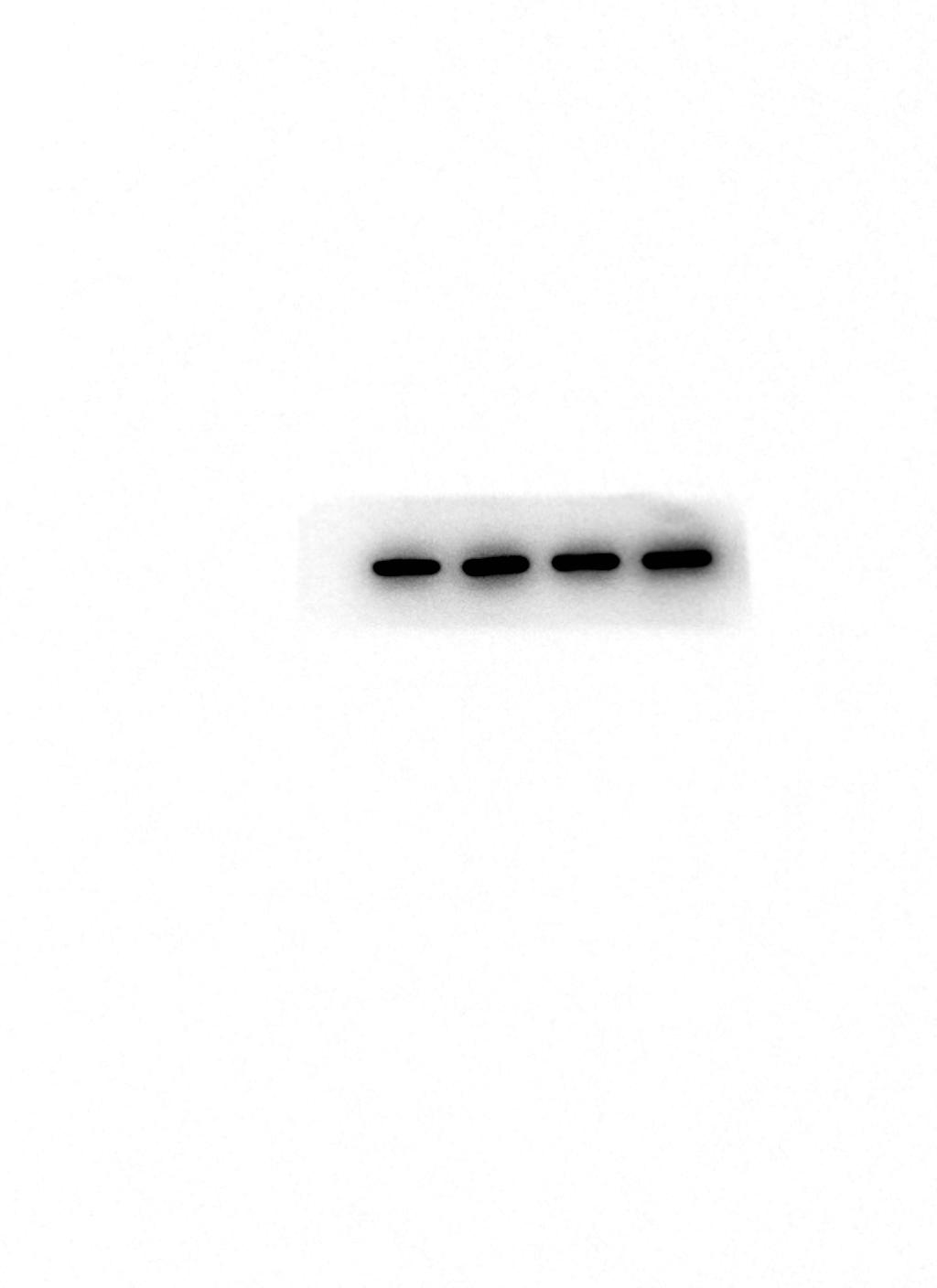

Supplement: Supplementary file 1 [file DataSheet_1.zip › Original data 1/Figure 5A/GAPDH-1/GAPDH-1.jpg]

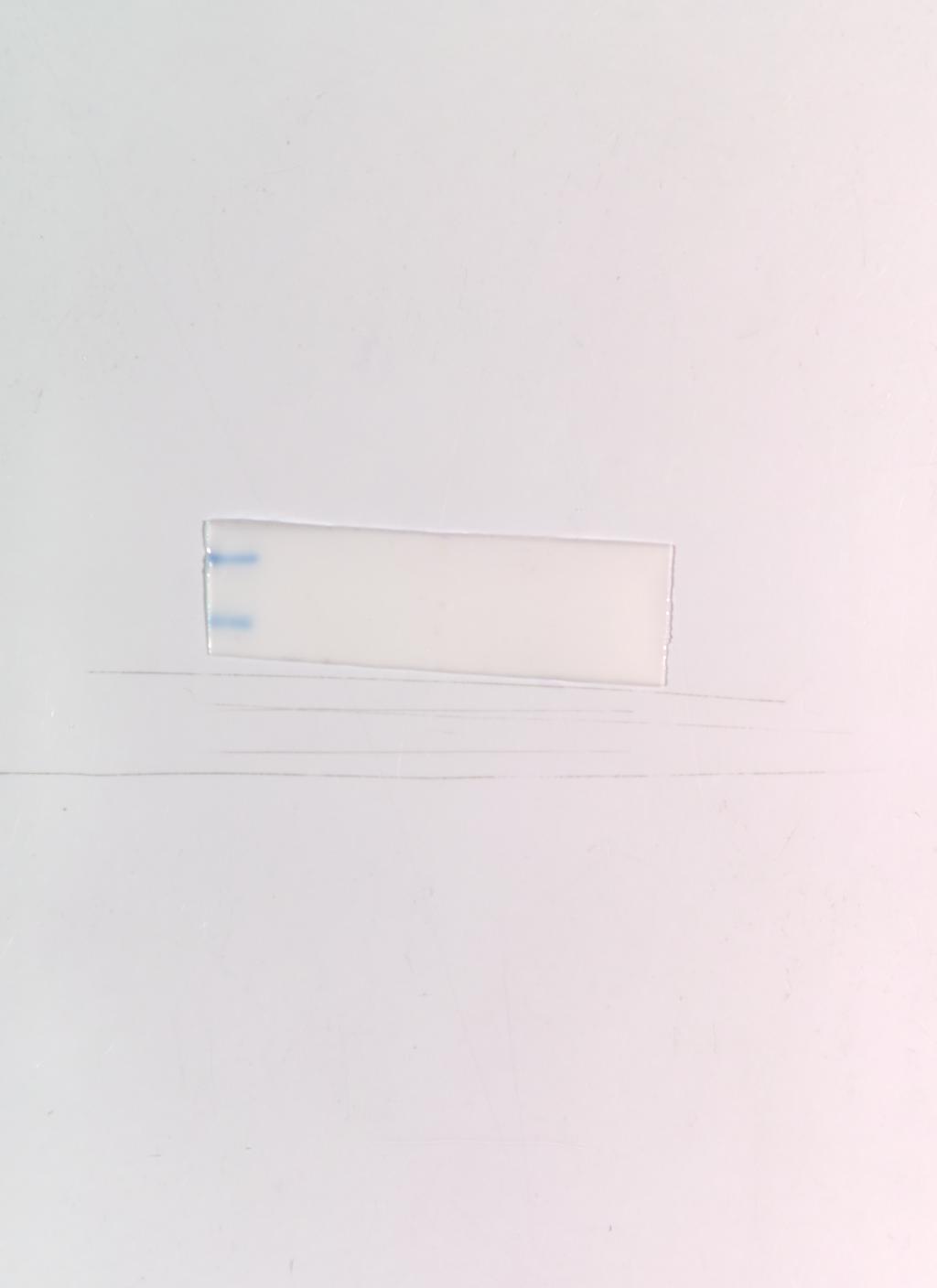

Supplement: Supplementary file 1 [file DataSheet_1.zip › Original data 1/Figure 5A/GAPDH-2 M/GAPDH-2 M.jpg]

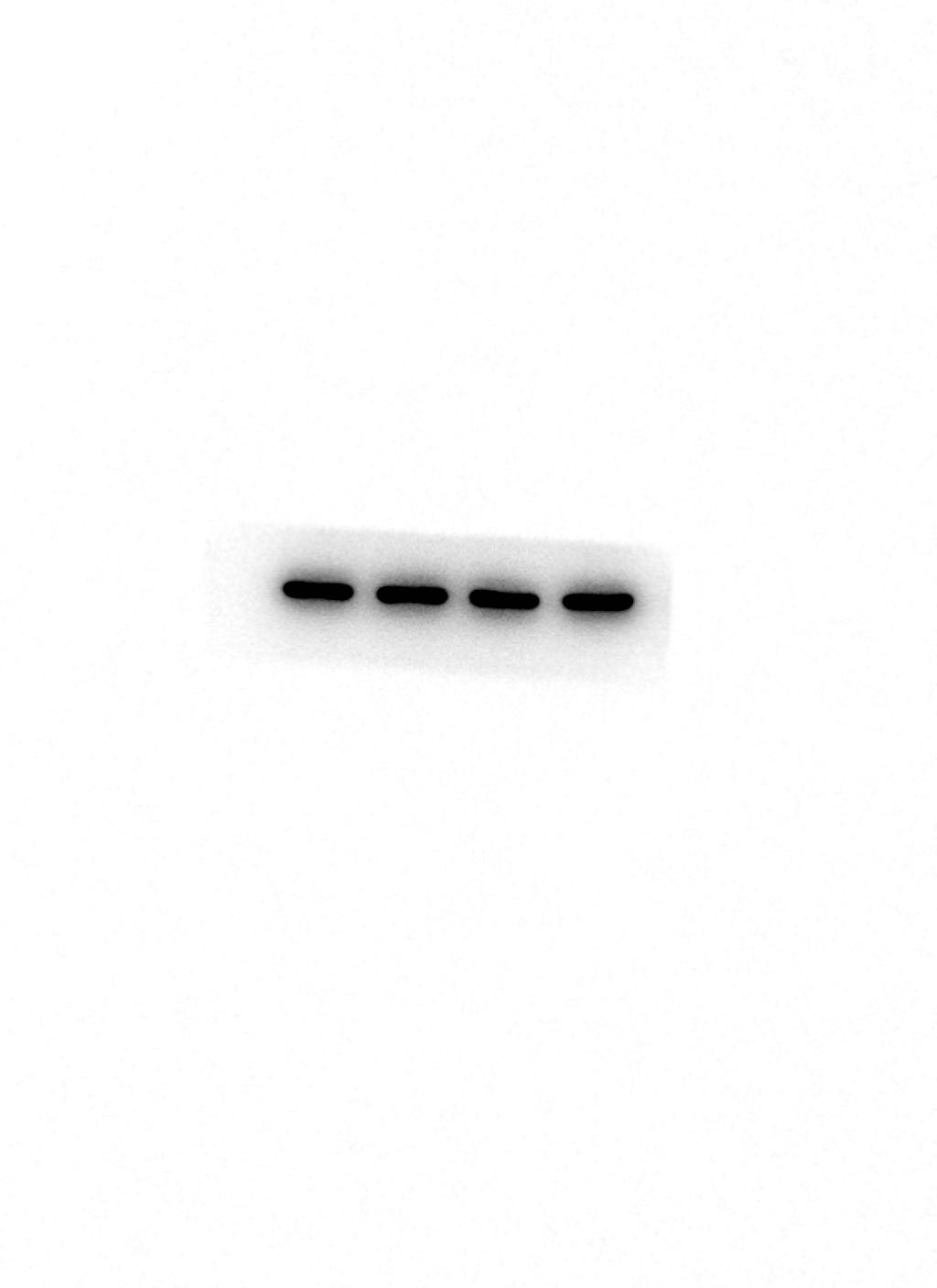

Supplement: Supplementary file 1 [file DataSheet_1.zip › Original data 1/Figure 5A/GAPDH-2/GAPDH-2.jpg]

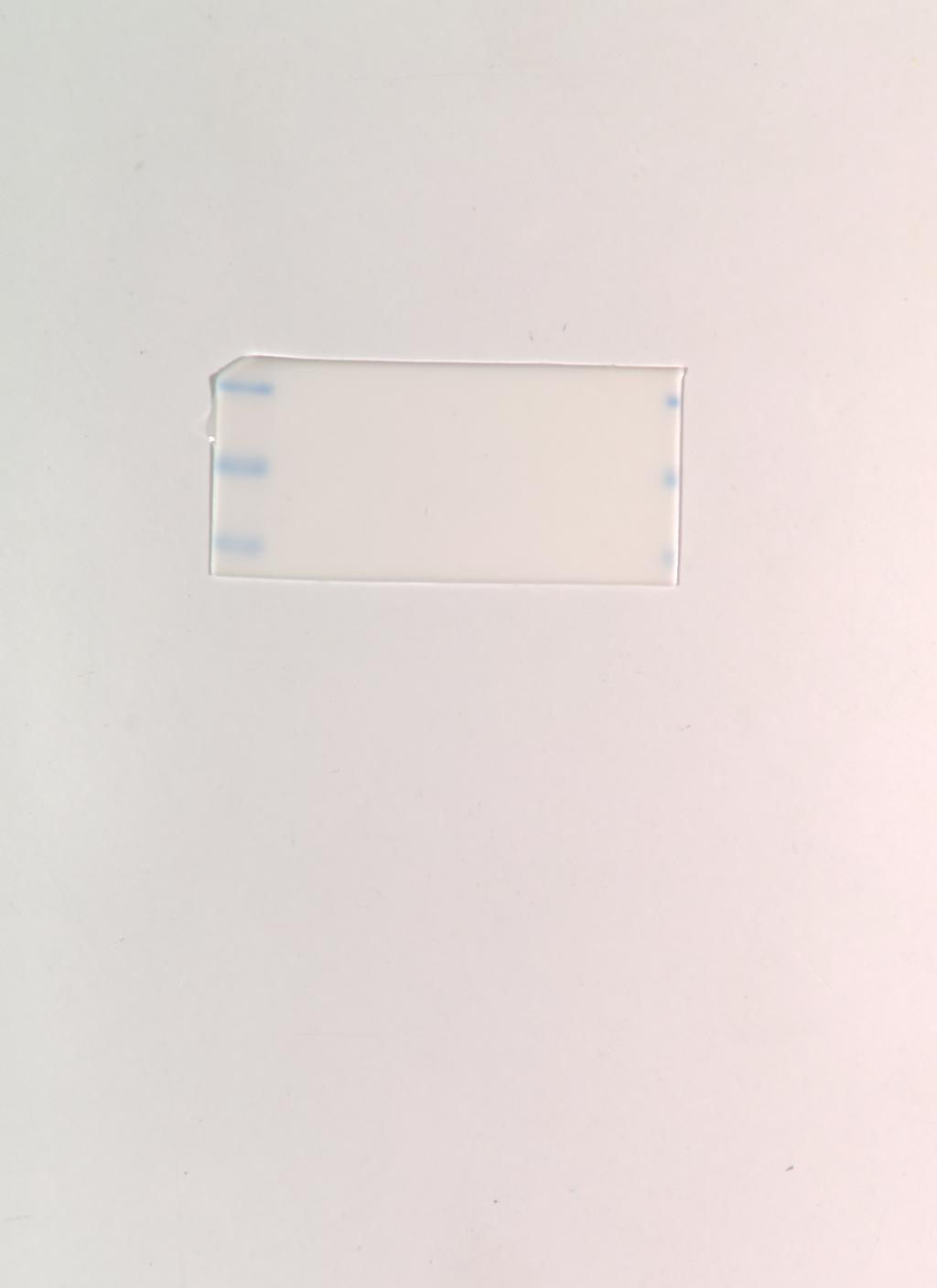

Supplement: Supplementary file 1 [file DataSheet_1.zip › Original data 1/Figure 5C/CDK1-1 M/CDK1-1 M.jpg]

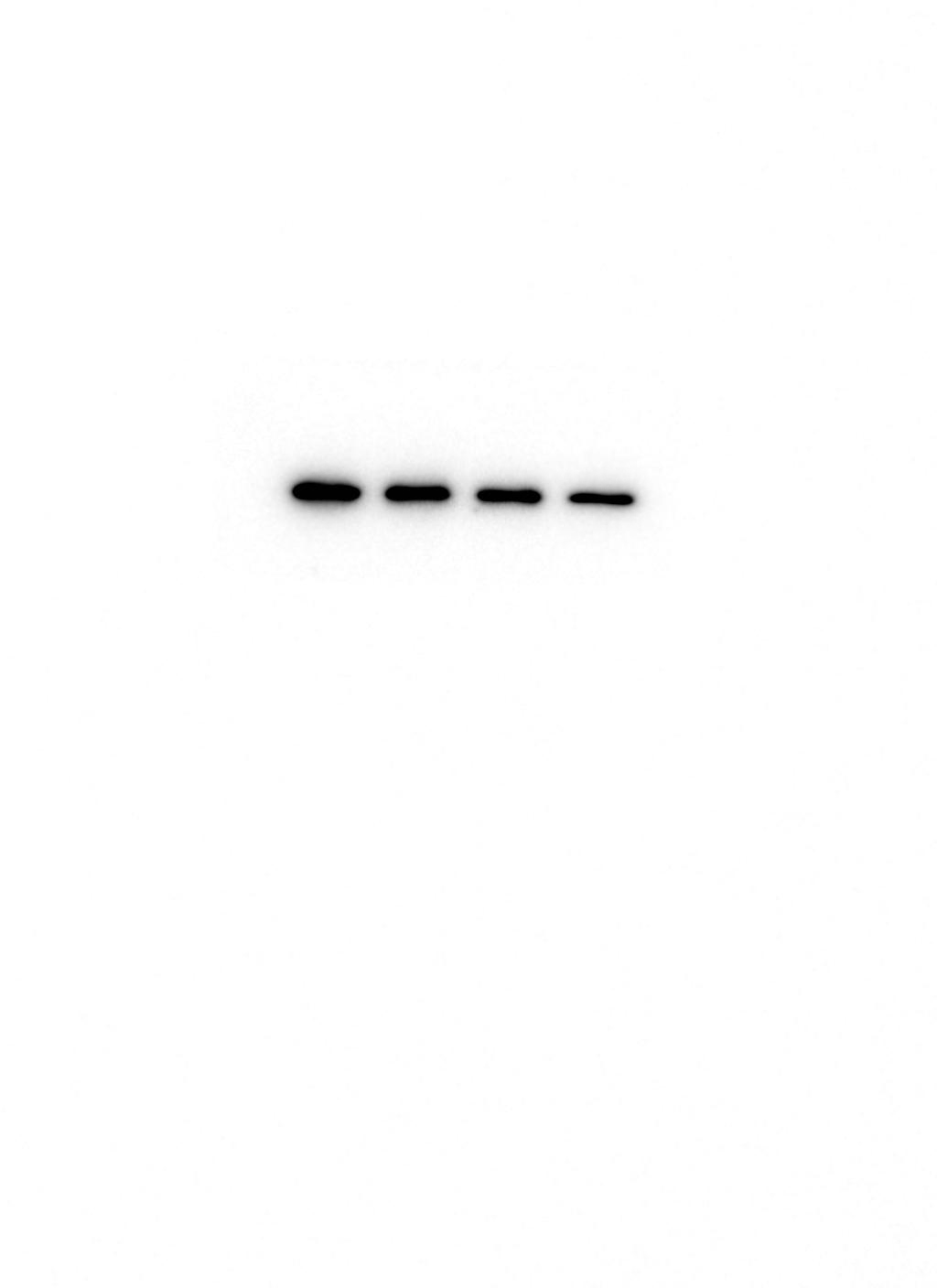

Supplement: Supplementary file 1 [file DataSheet_1.zip › Original data 1/Figure 5C/CDK1-1/CDK1-1.jpg]

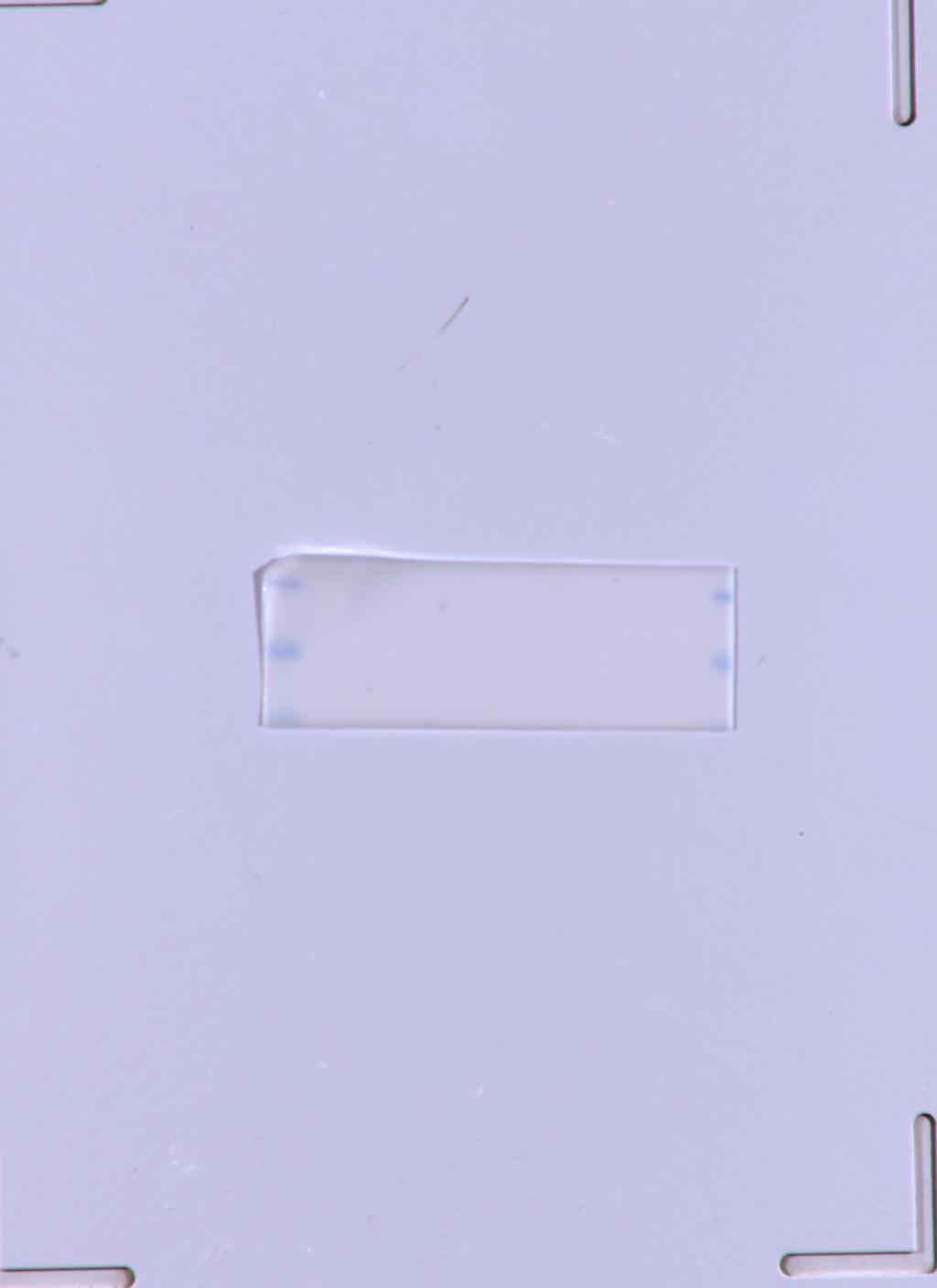

Supplement: Supplementary file 1 [file DataSheet_1.zip › Original data 1/Figure 5C/CDK1-2 M/CDK1-2 M.jpg]

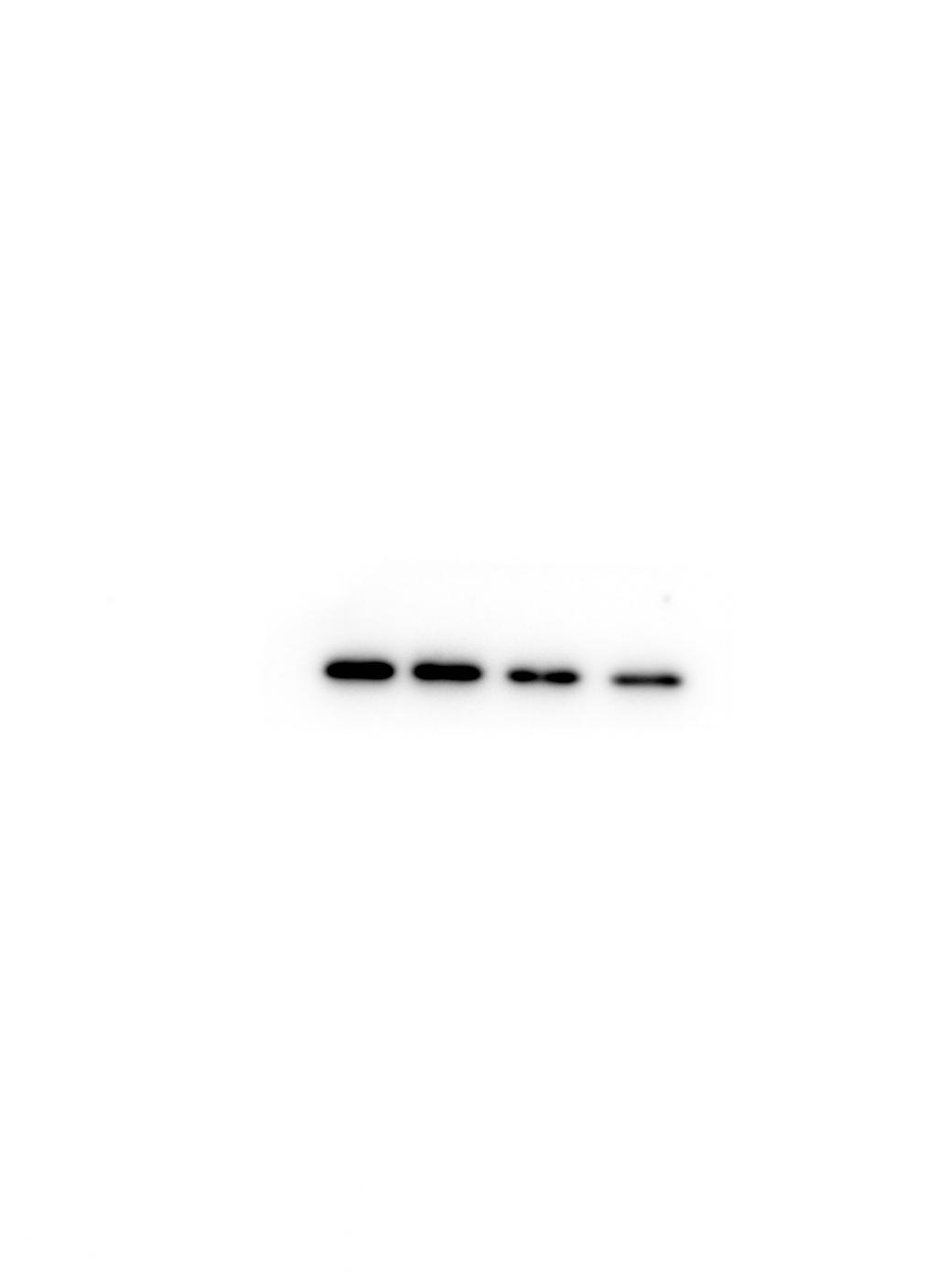

Supplement: Supplementary file 1 [file DataSheet_1.zip › Original data 1/Figure 5C/CDK1-2/CDK1-2.jpg]

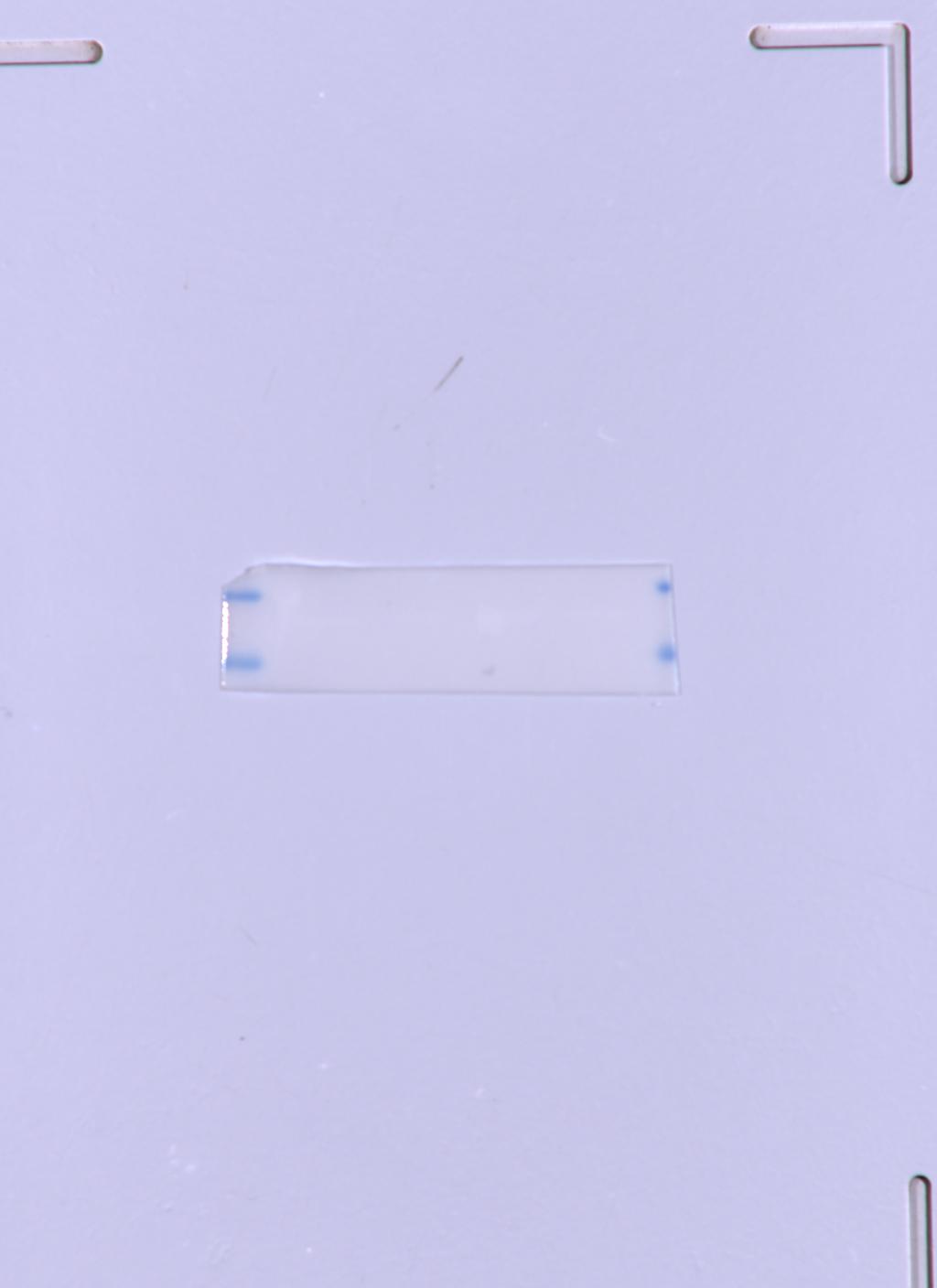

Supplement: Supplementary file 1 [file DataSheet_1.zip › Original data 1/Figure 5C/GAPDH-1 M/GAPDH-1 M.jpg]

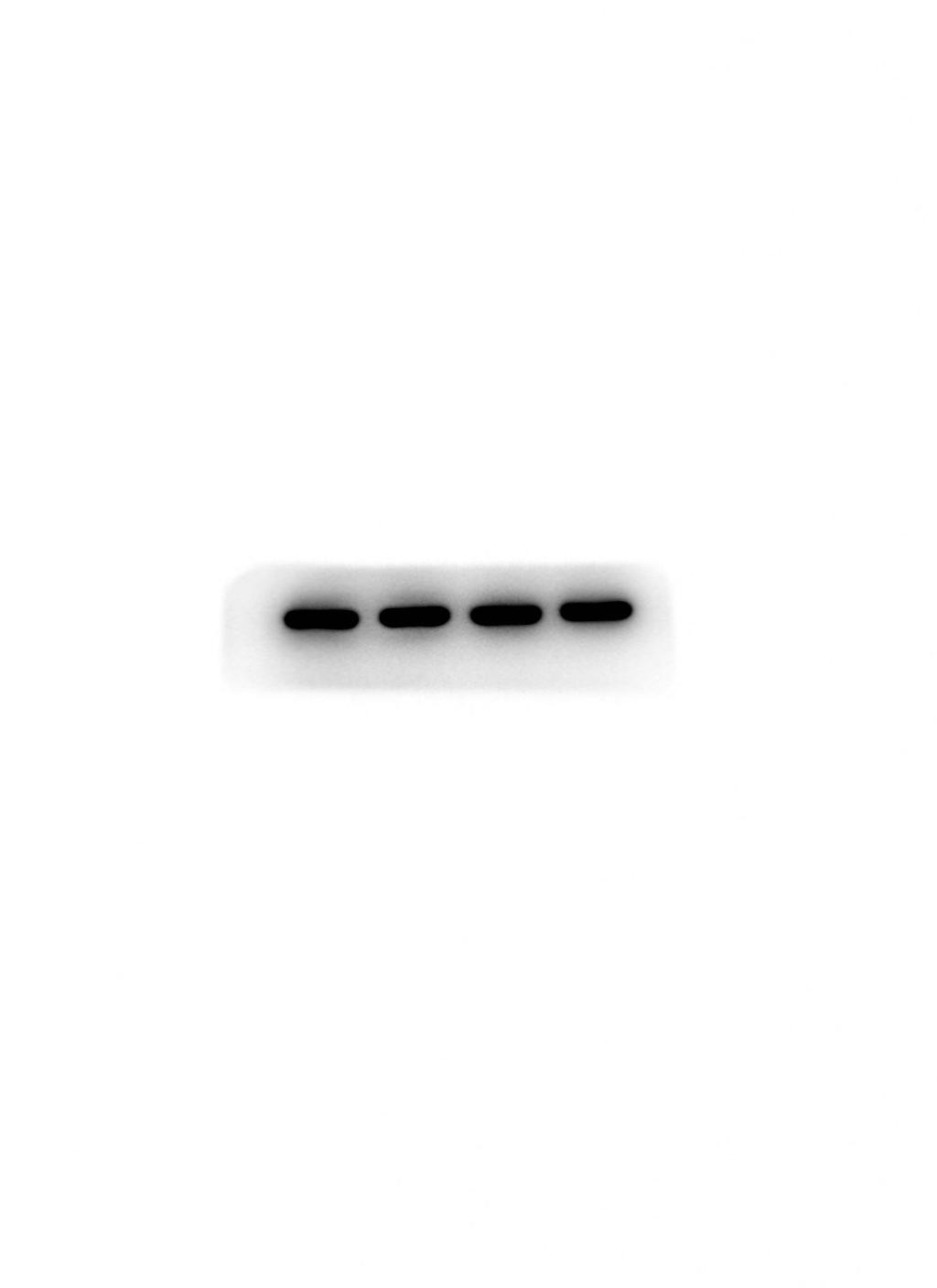

Supplement: Supplementary file 1 [file DataSheet_1.zip › Original data 1/Figure 5C/GAPDH-1/GAPDH-1.jpg]

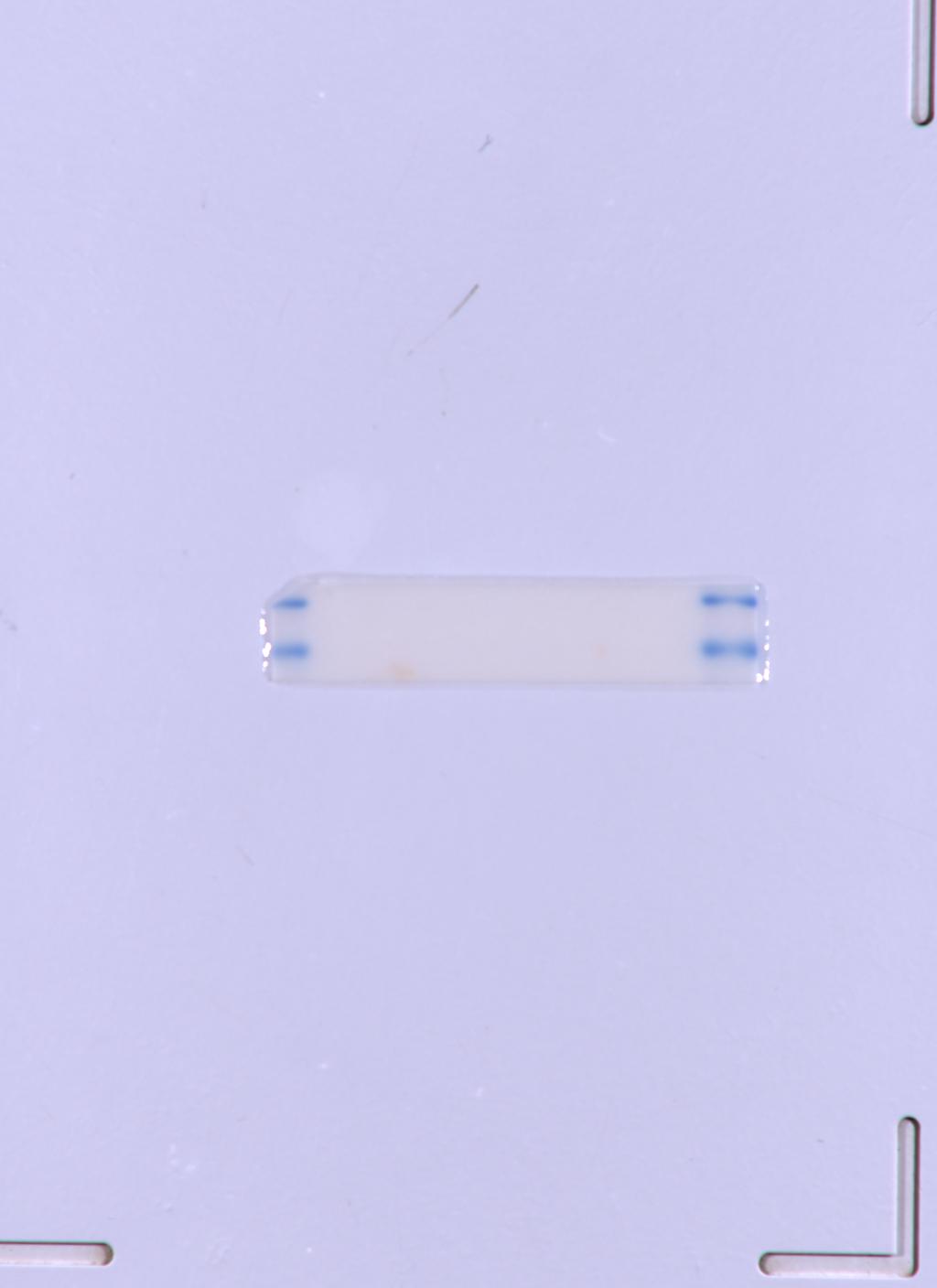

Supplement: Supplementary file 1 [file DataSheet_1.zip › Original data 1/Figure 5C/GAPDH-2 M/GAPDH-2 M.jpg]

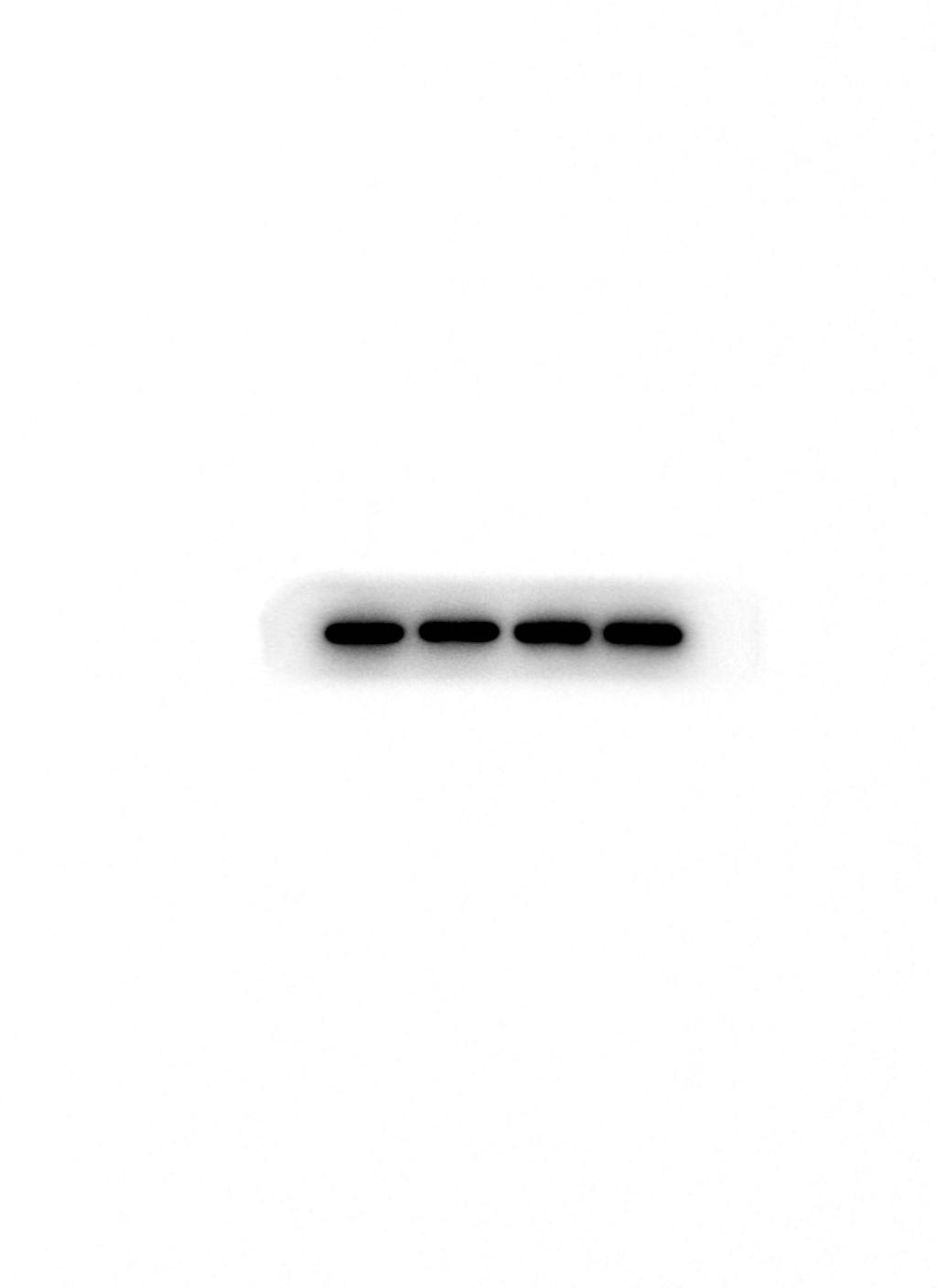

Supplement: Supplementary file 1 [file DataSheet_1.zip › Original data 1/Figure 5C/GAPDH-2/GAPDH-2.jpg]

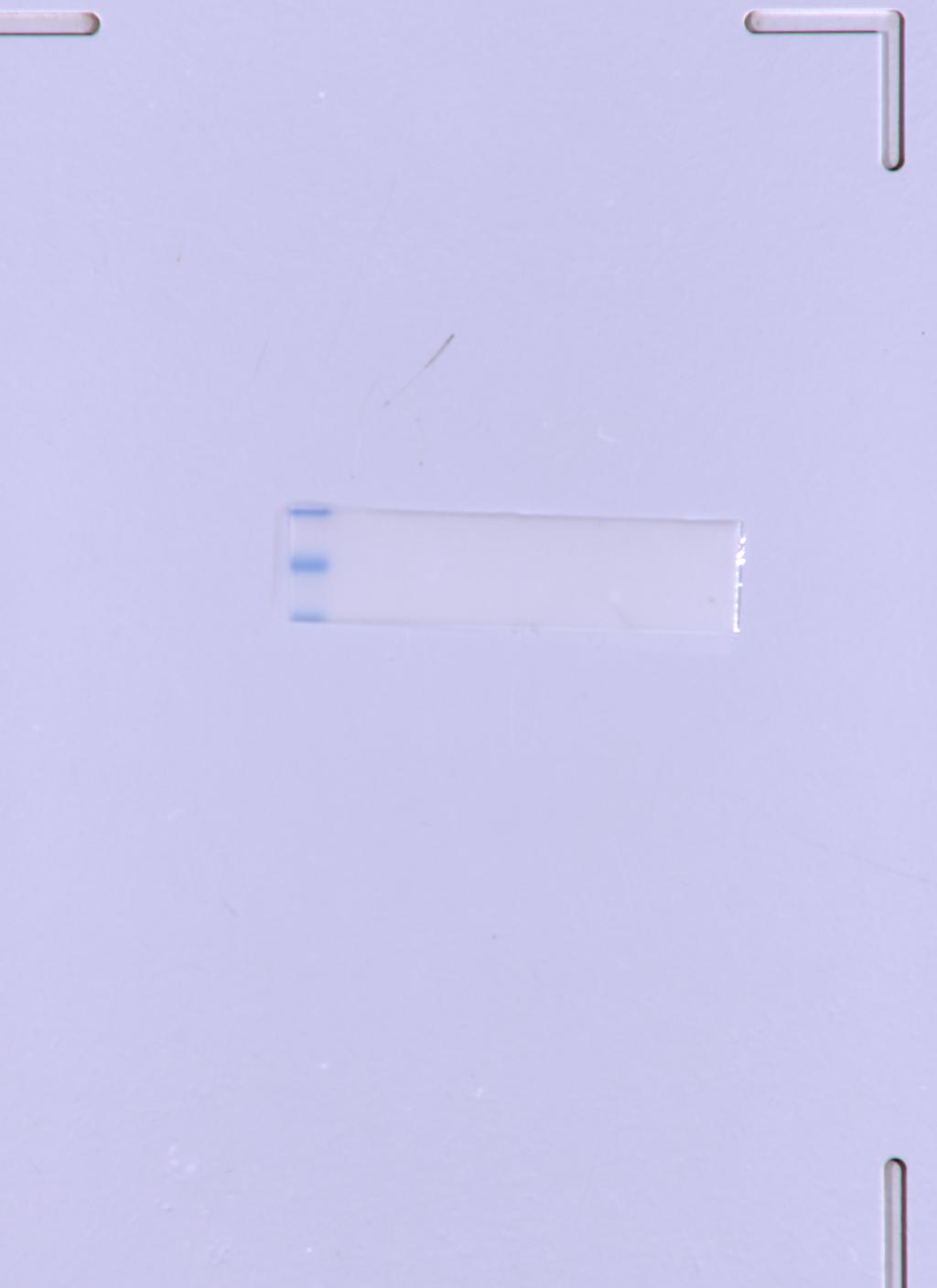

Supplement: Supplementary file 1 [file DataSheet_1.zip › Original data 1/Figure 5D/down/CDK1 M/CDK1 M.jpg]

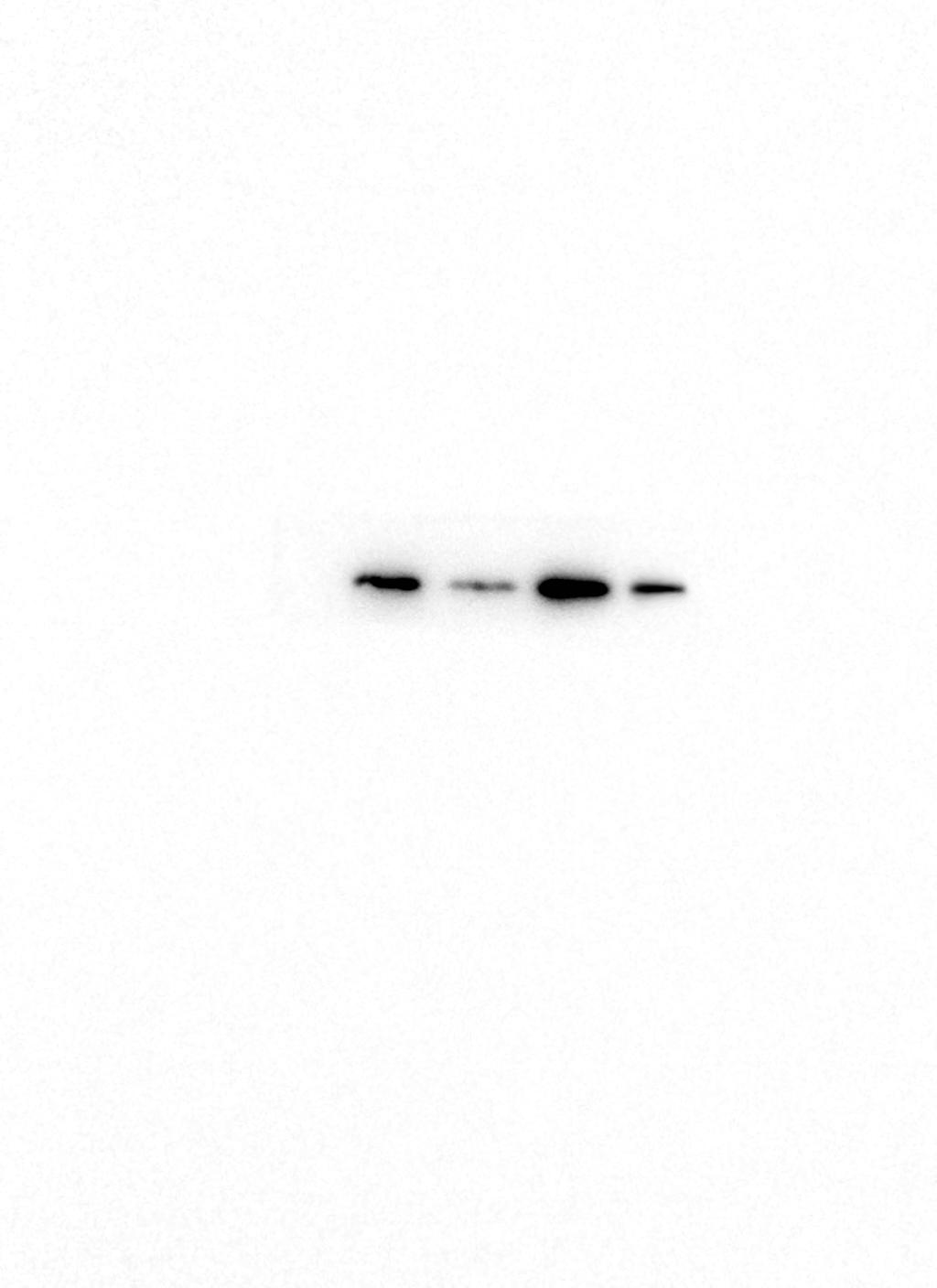

Supplement: Supplementary file 1 [file DataSheet_1.zip › Original data 1/Figure 5D/down/CDK1/CDK1.jpg]

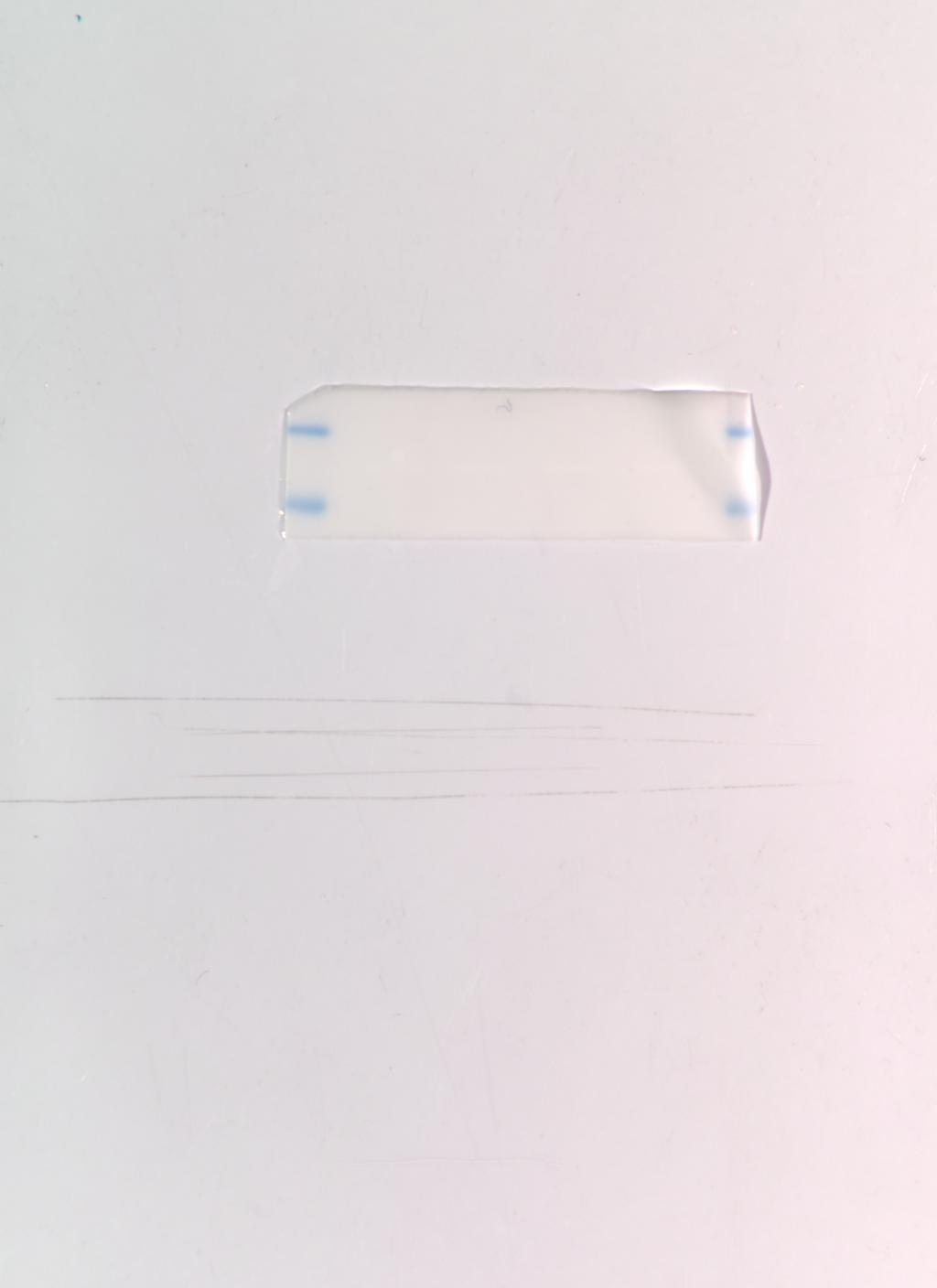

Supplement: Supplementary file 1 [file DataSheet_1.zip › Original data 1/Figure 5D/down/GAPDH M/GAPDH M.jpg]

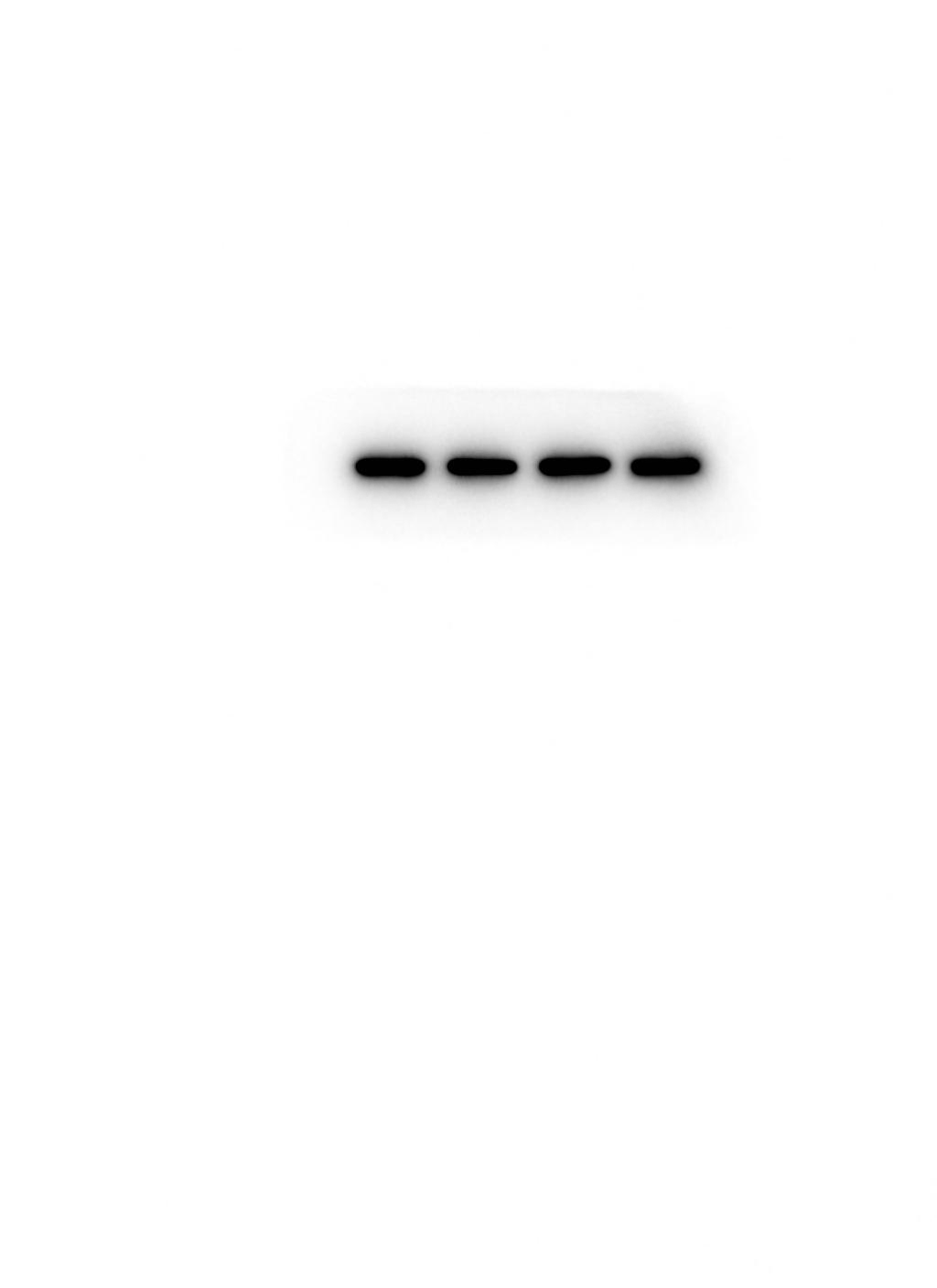

Supplement: Supplementary file 1 [file DataSheet_1.zip › Original data 1/Figure 5D/down/GAPDH/GAPDH.jpg]

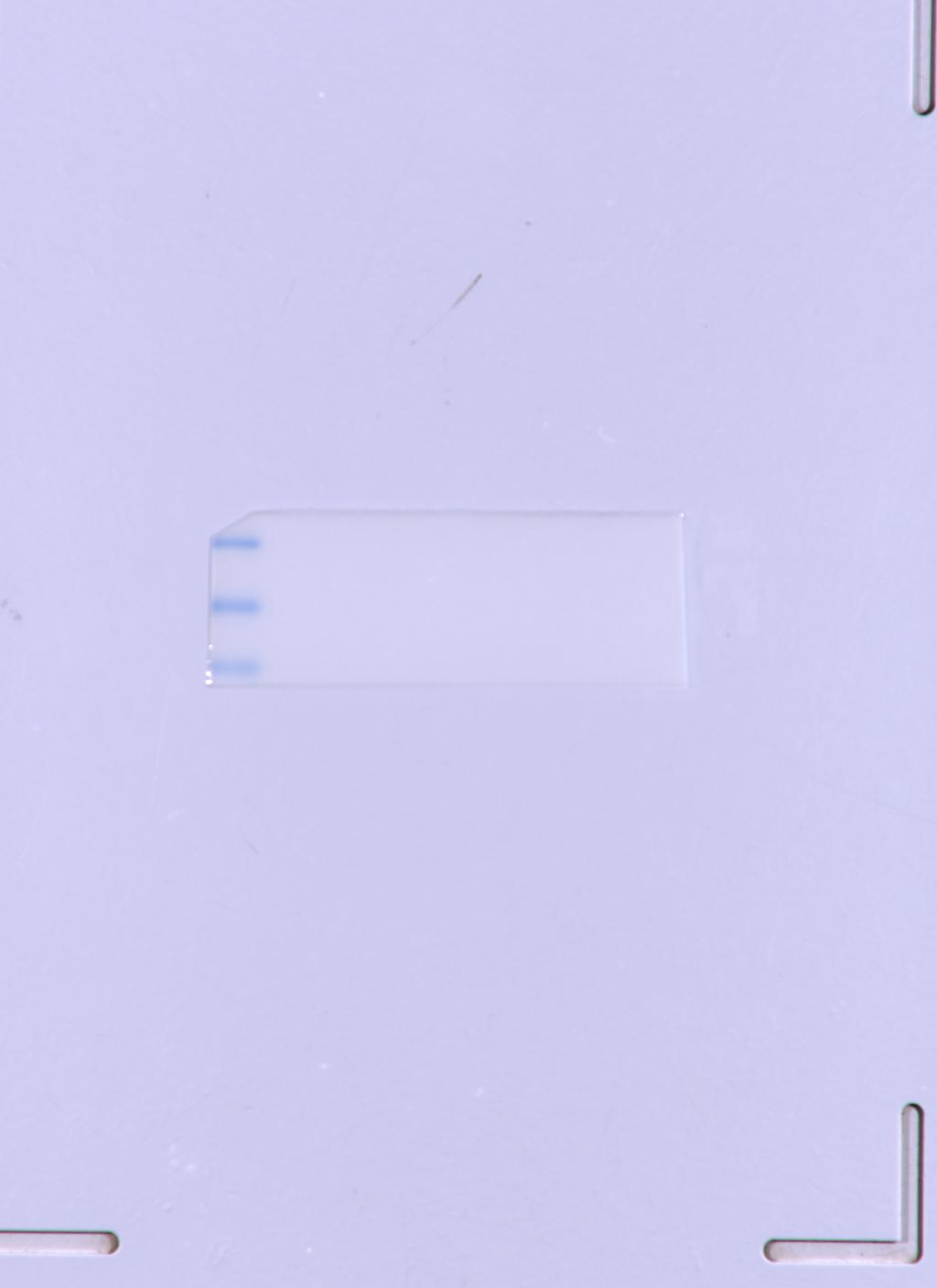

Supplement: Supplementary file 1 [file DataSheet_1.zip › Original data 1/Figure 5D/up/CDK1 M/CDK1 M.jpg]

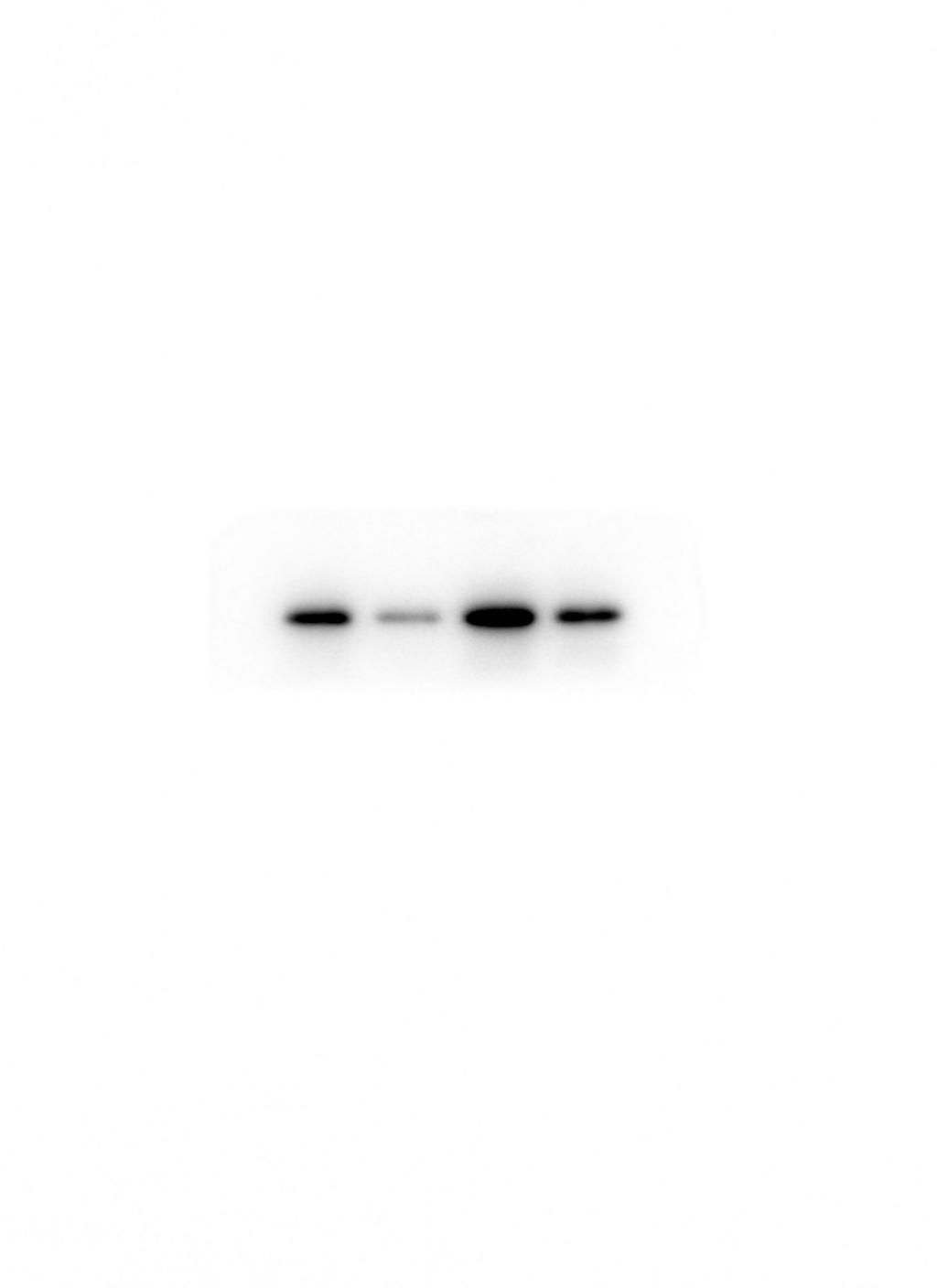

Supplement: Supplementary file 1 [file DataSheet_1.zip › Original data 1/Figure 5D/up/CDK1/CDK1.jpg]

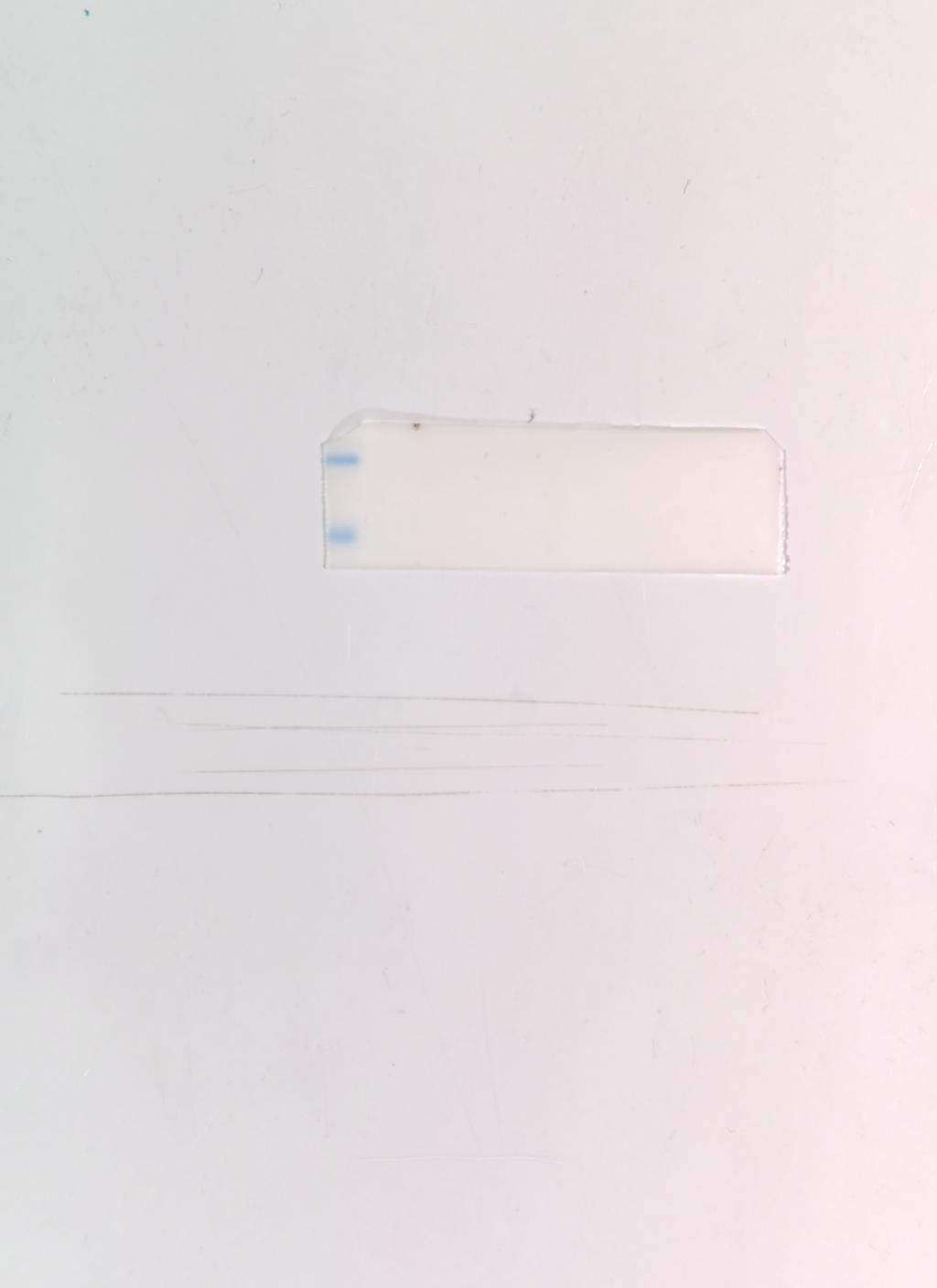

Supplement: Supplementary file 1 [file DataSheet_1.zip › Original data 1/Figure 5D/up/GAPDH M/GAPDH M.jpg]

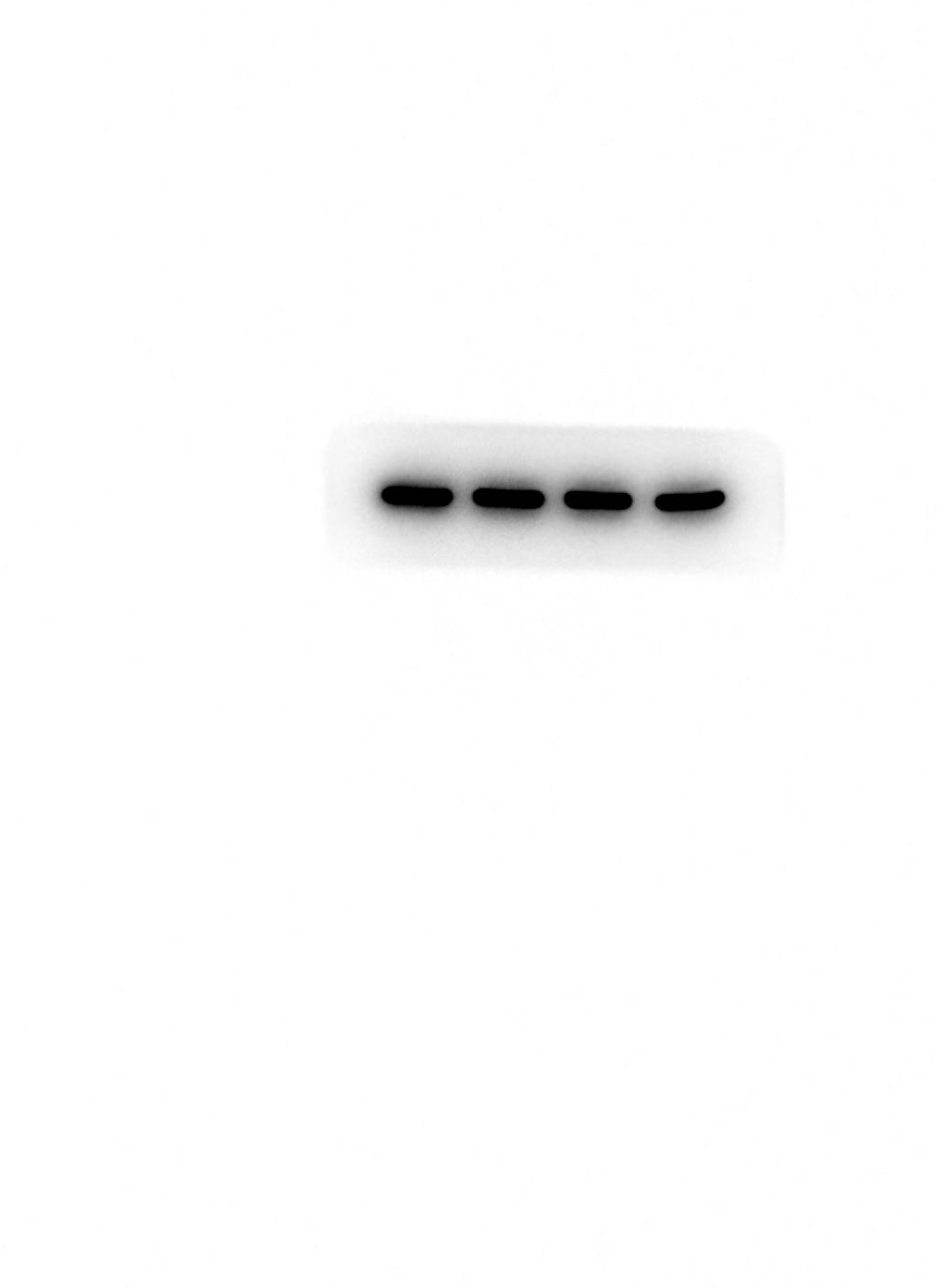

Supplement: Supplementary file 1 [file DataSheet_1.zip › Original data 1/Figure 5D/up/GAPDH/GAPDH.jpg]

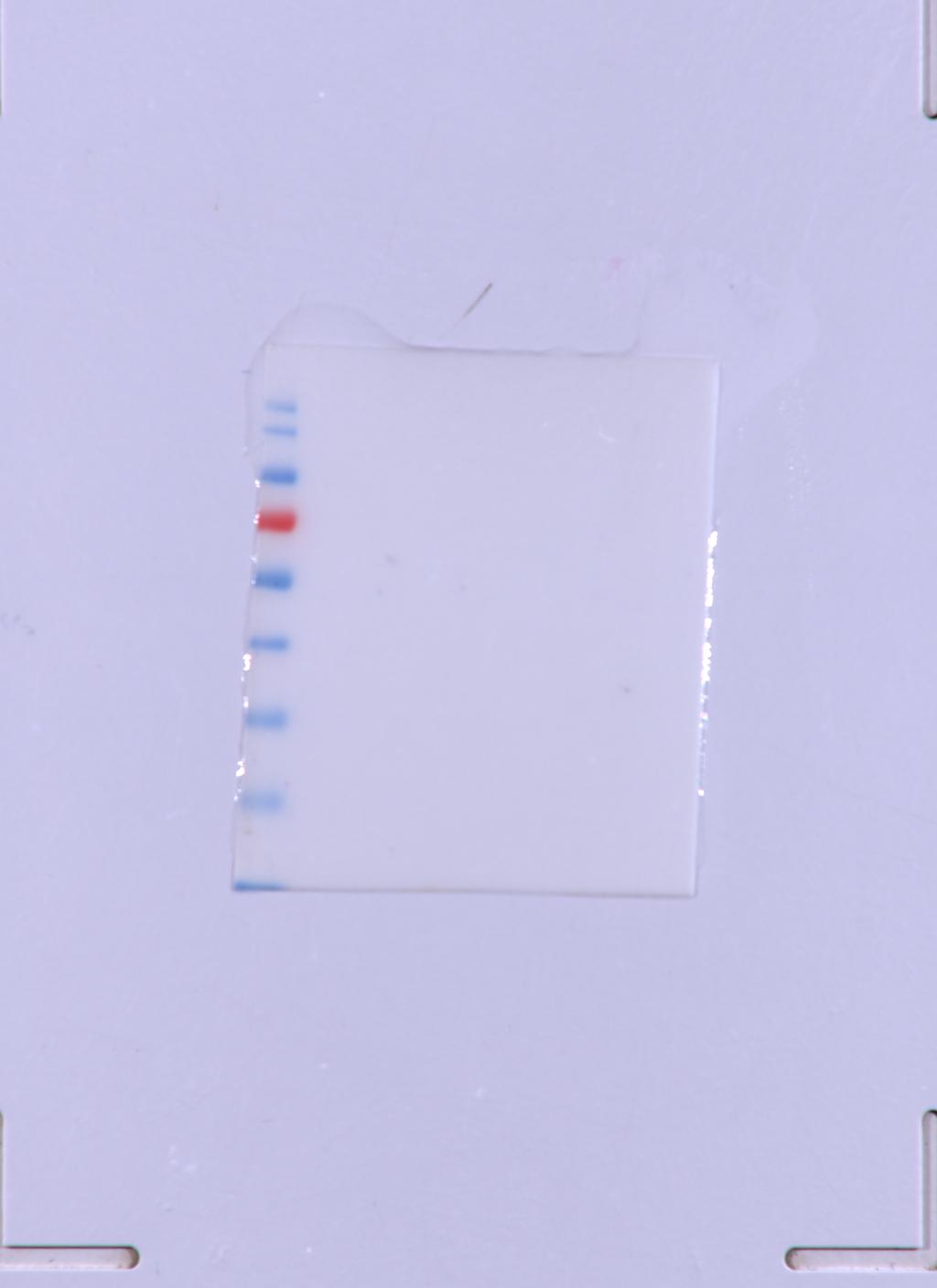

Supplement: Supplementary file 1 [file DataSheet_1.zip › Original data 1/Figure 5E/UB M/UB M.jpg]

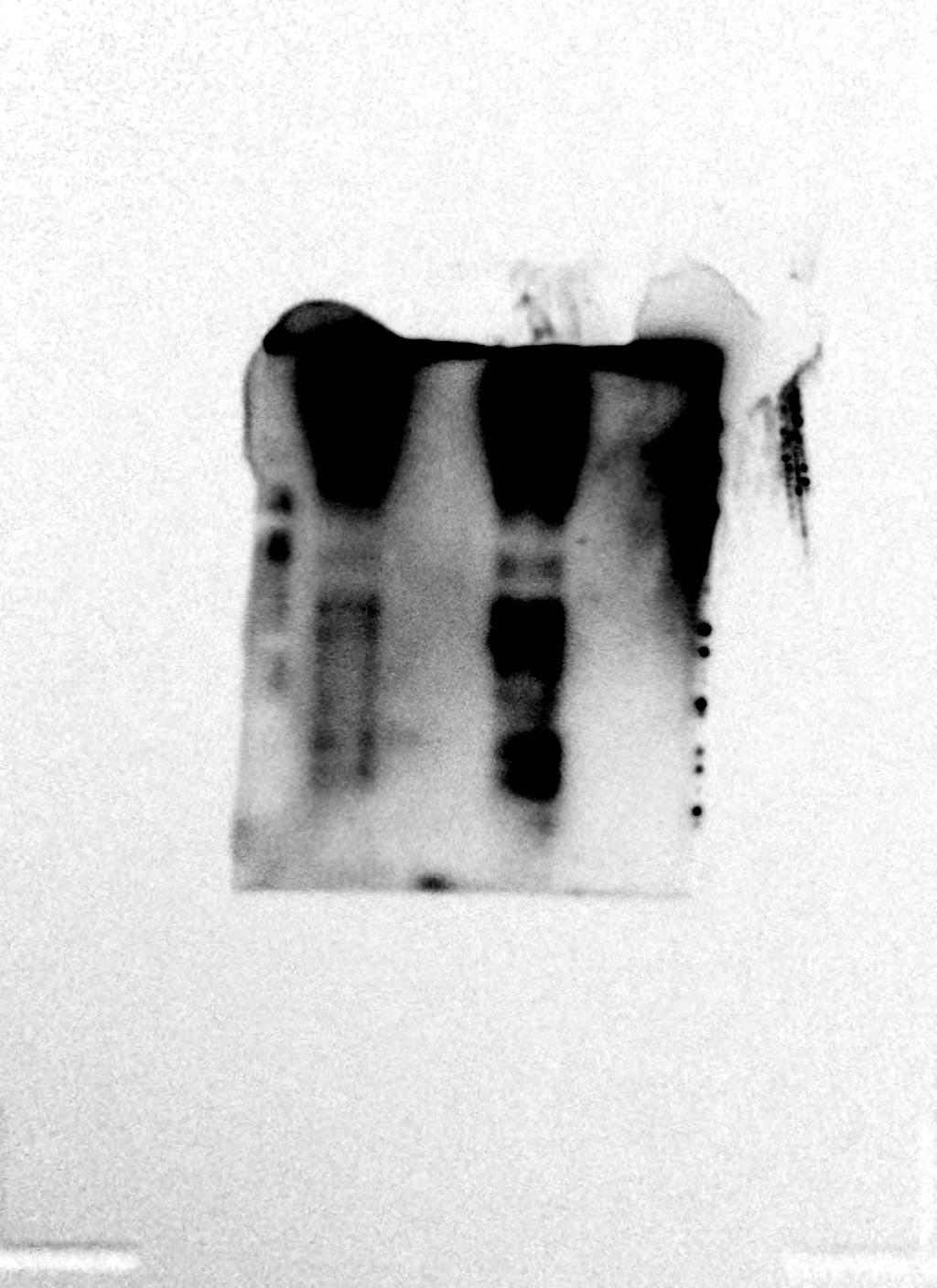

Supplement: Supplementary file 1 [file DataSheet_1.zip › Original data 1/Figure 5E/UB/UB.jpg]

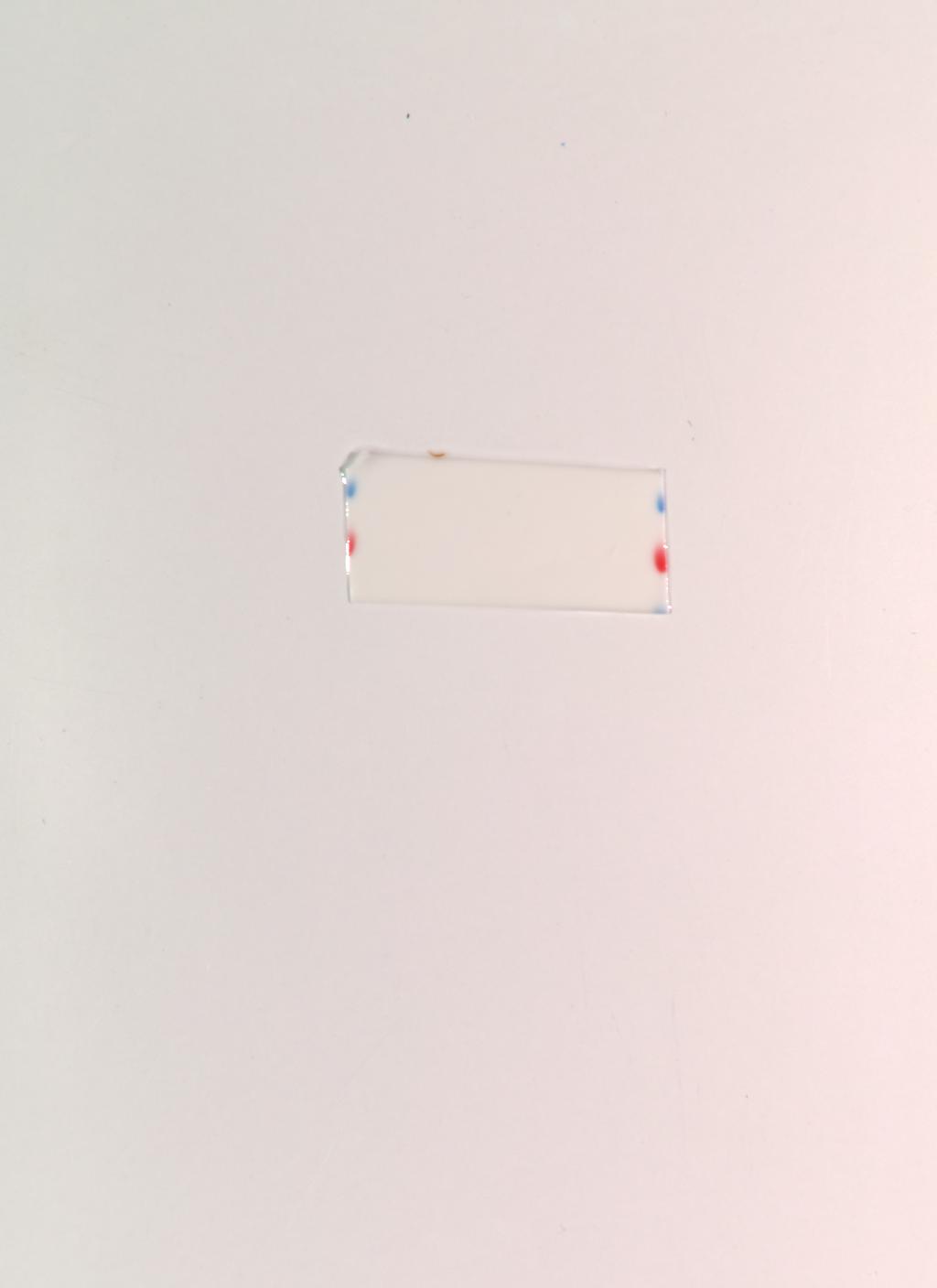

Supplement: Supplementary file 1 [file DataSheet_1.zip › Original data 1/Figure 5F/DEPDC1B M/DEPDC1B M.jpg]

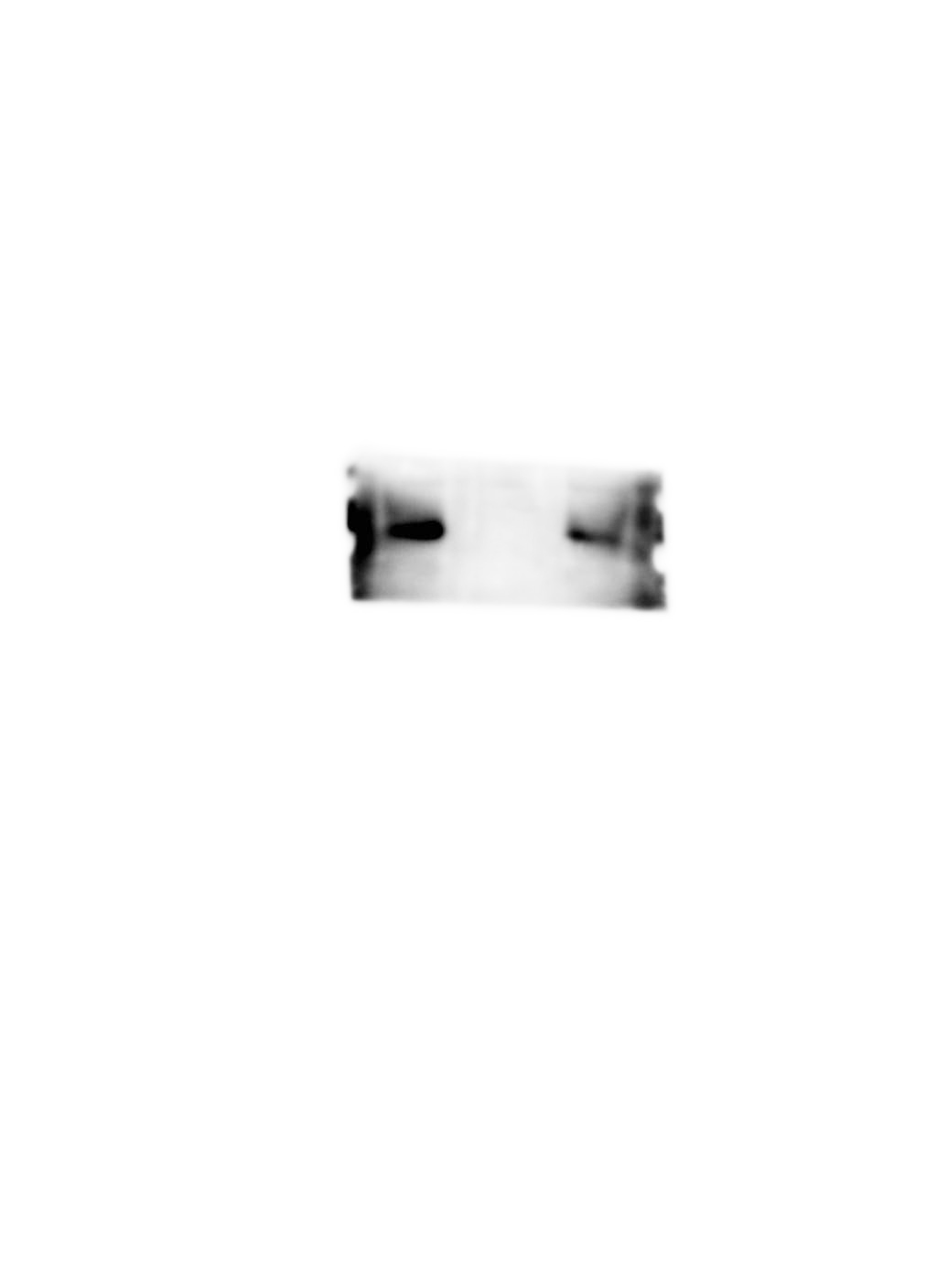

Supplement: Supplementary file 1 [file DataSheet_1.zip › Original data 1/Figure 5F/DEPDC1B/DEPDC1B.jpg]

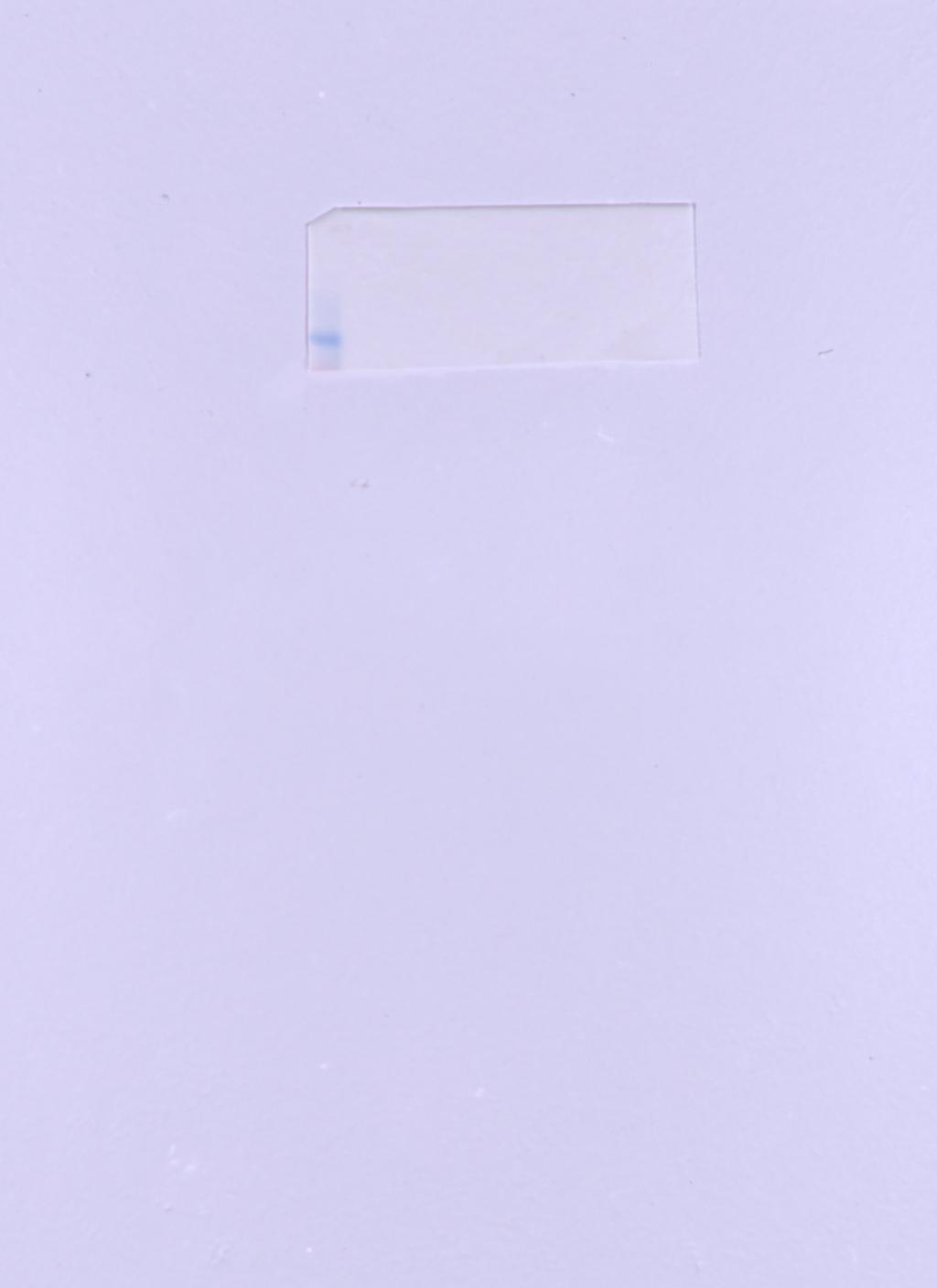

Supplement: Supplementary file 1 [file DataSheet_1.zip › Original data 1/Figure 5F/SMURF1 M/SMURF1 M.jpg]

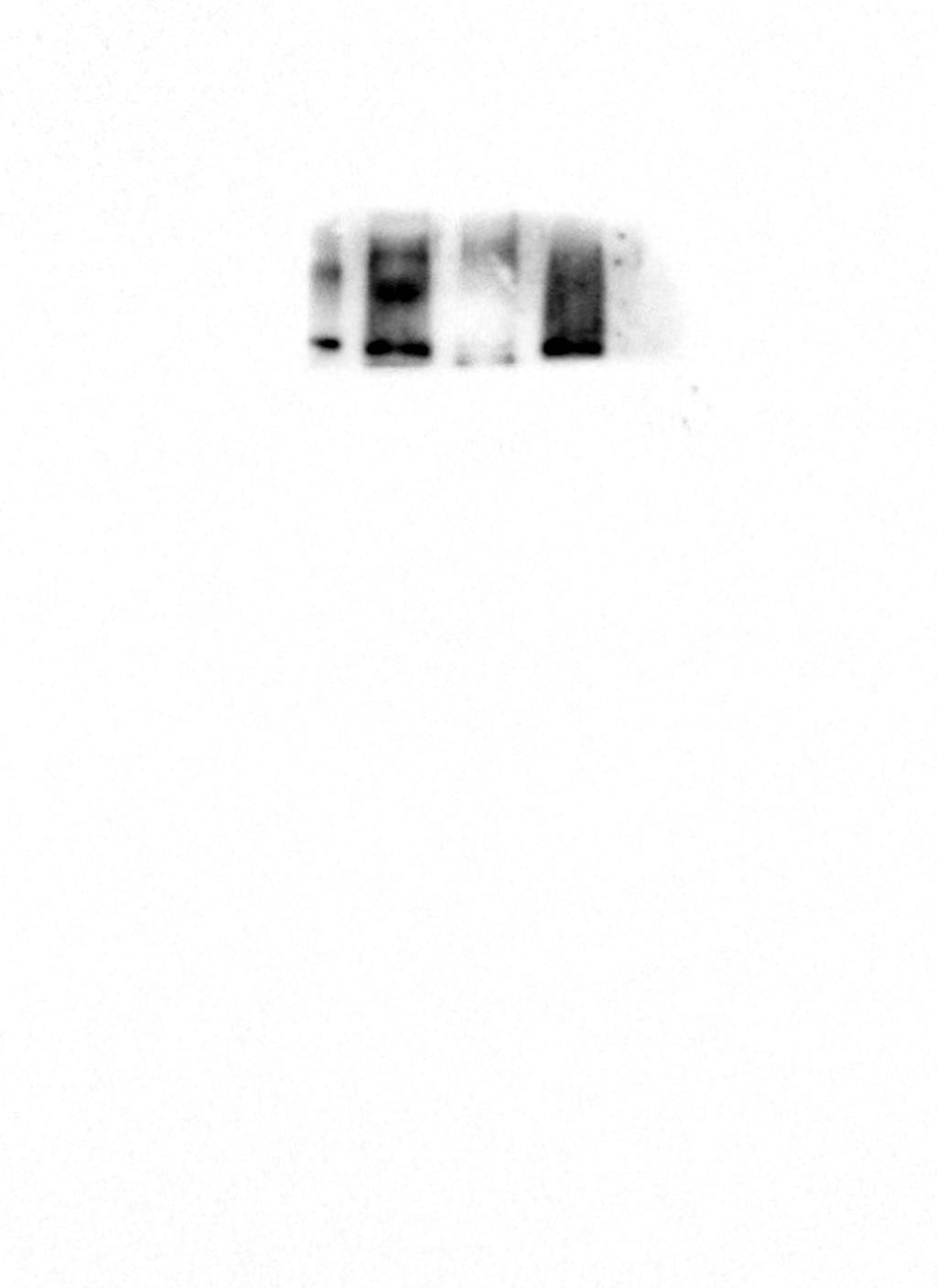

Supplement: Supplementary file 1 [file DataSheet_1.zip › Original data 1/Figure 5F/SMURF1/SMURF1.jpg]

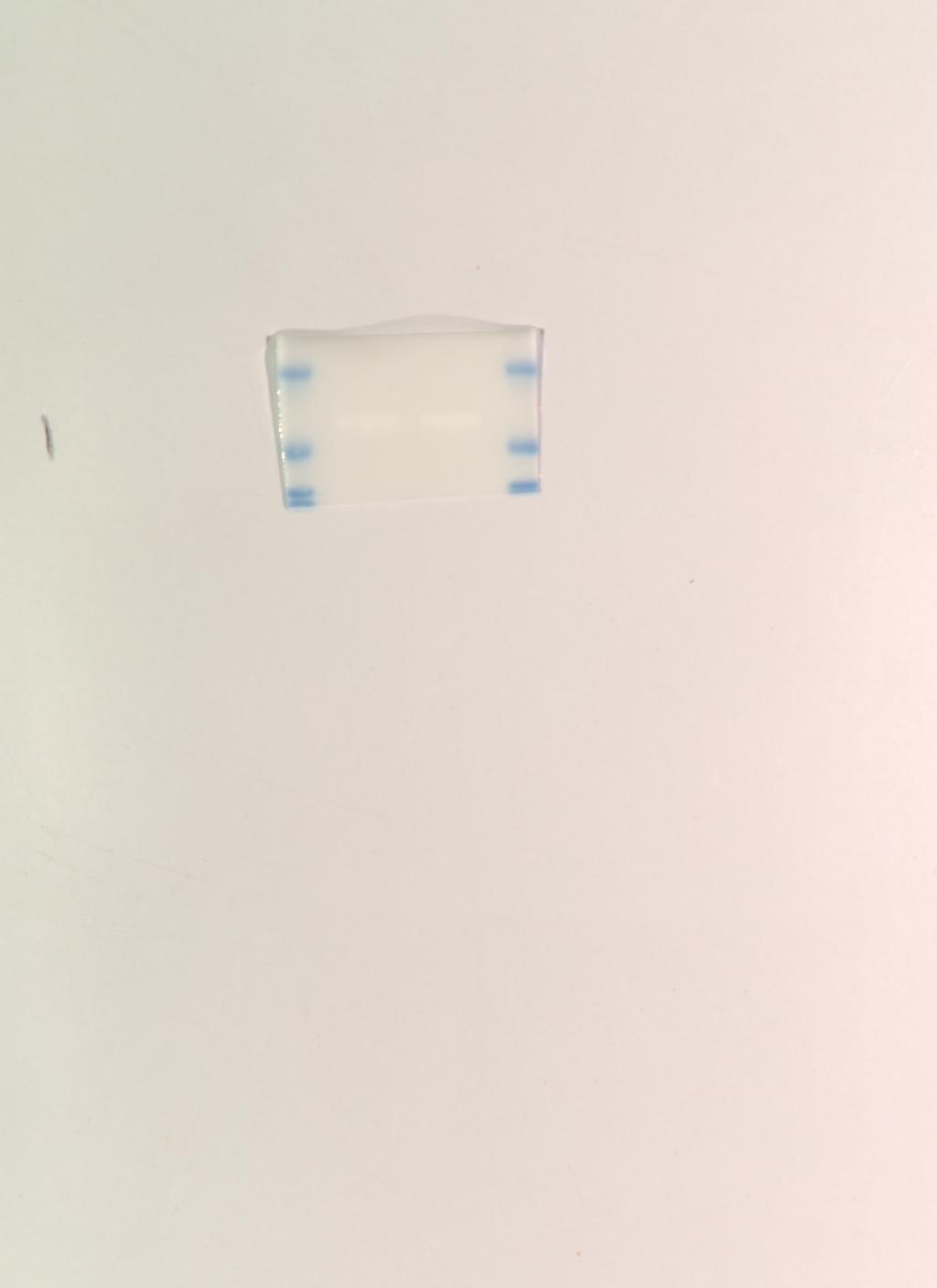

Supplement: Supplementary file 1 [file DataSheet_1.zip › Original data 1/Figure 5G/Input/CDK1 M/CDK1 M.jpg]

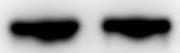

Supplement: Supplementary file 1 [file DataSheet_1.zip › Original data 1/Figure 5G/Input/CDK1.jpg]

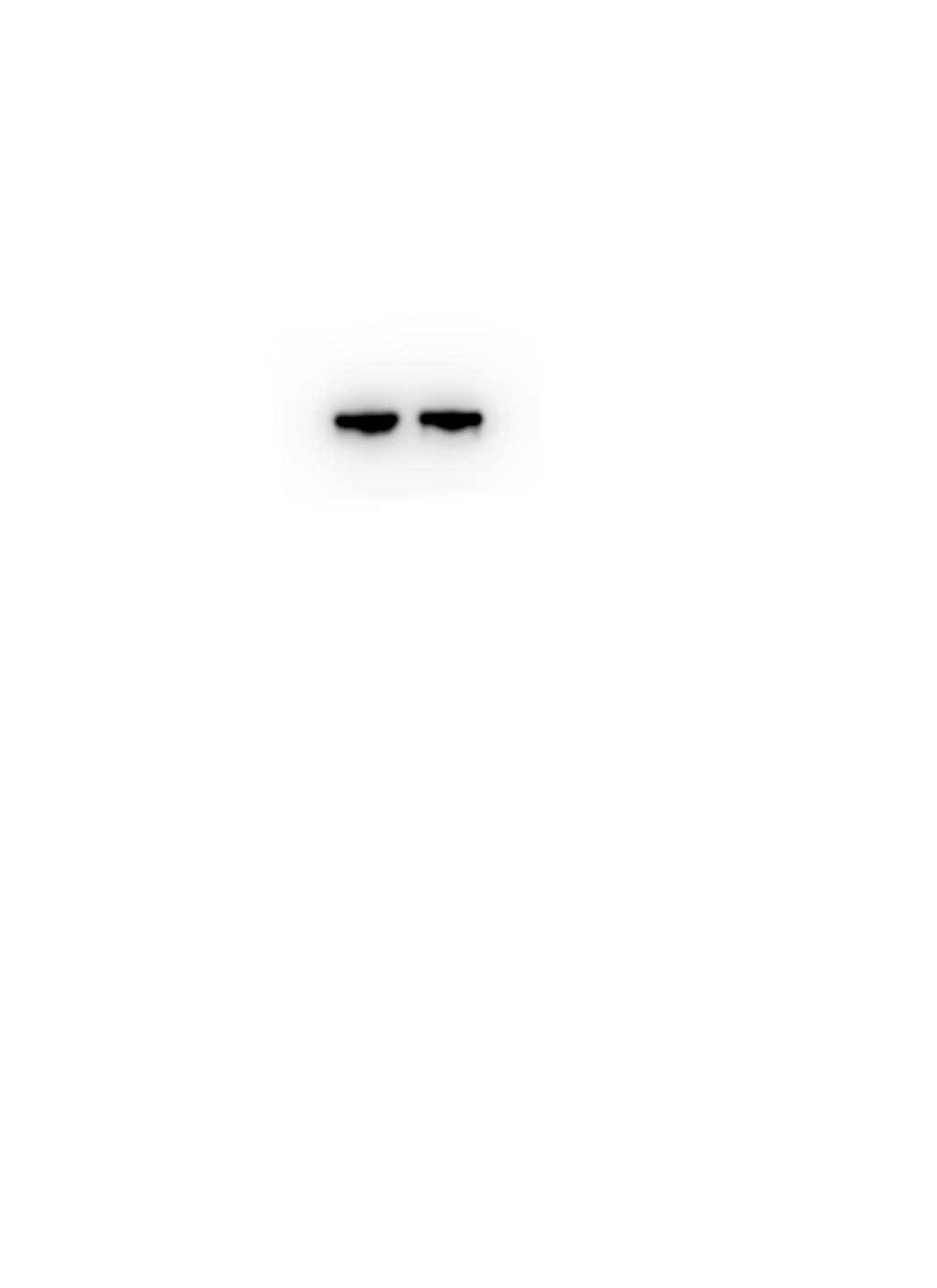

Supplement: Supplementary file 1 [file DataSheet_1.zip › Original data 1/Figure 5G/Input/CDK1/CDK1.jpg]

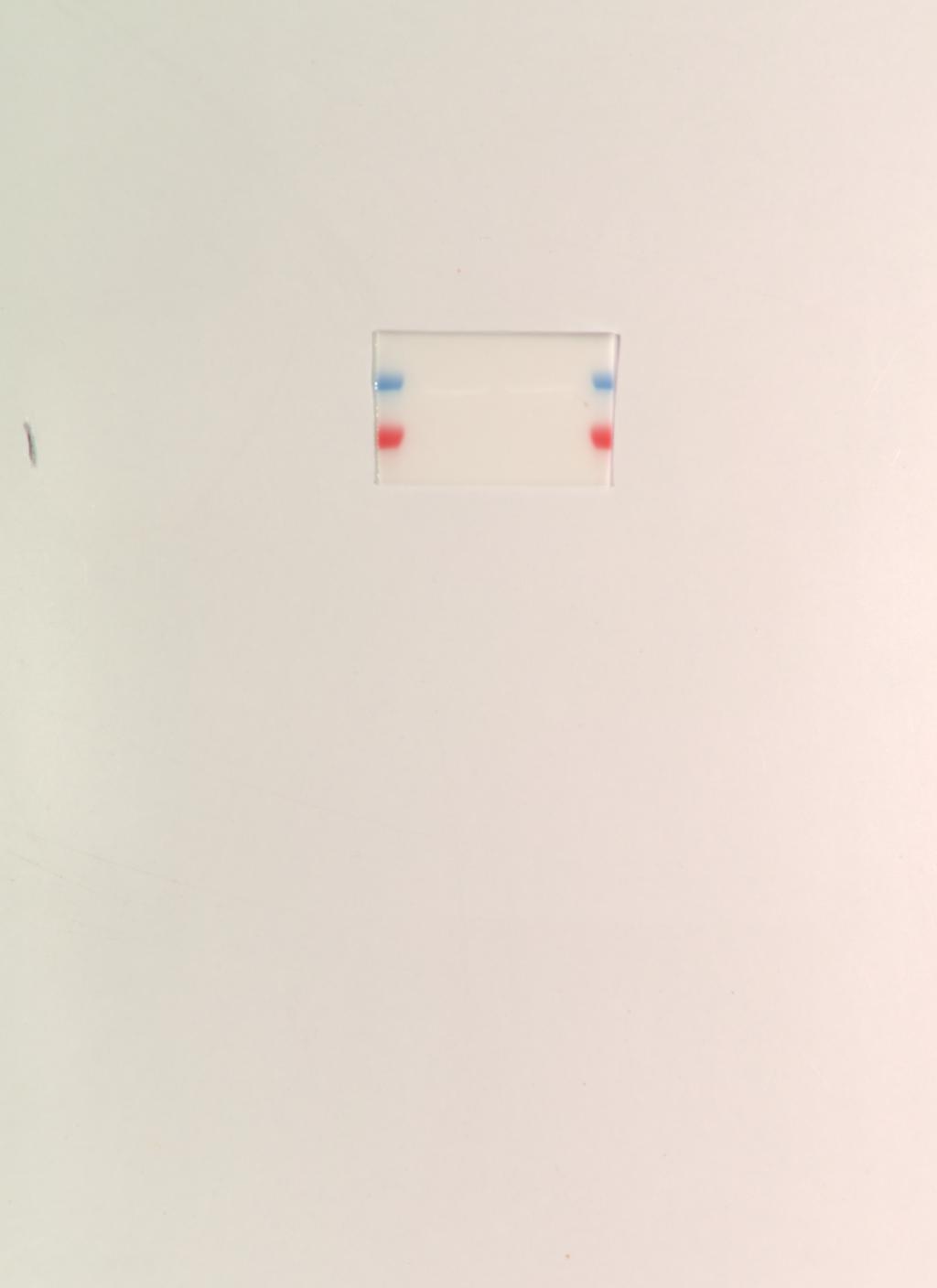

Supplement: Supplementary file 1 [file DataSheet_1.zip › Original data 1/Figure 5G/Input/SMURF1 M/SMURF1 M.jpg]

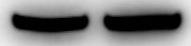

Supplement: Supplementary file 1 [file DataSheet_1.zip › Original data 1/Figure 5G/Input/SMURF1.jpg]

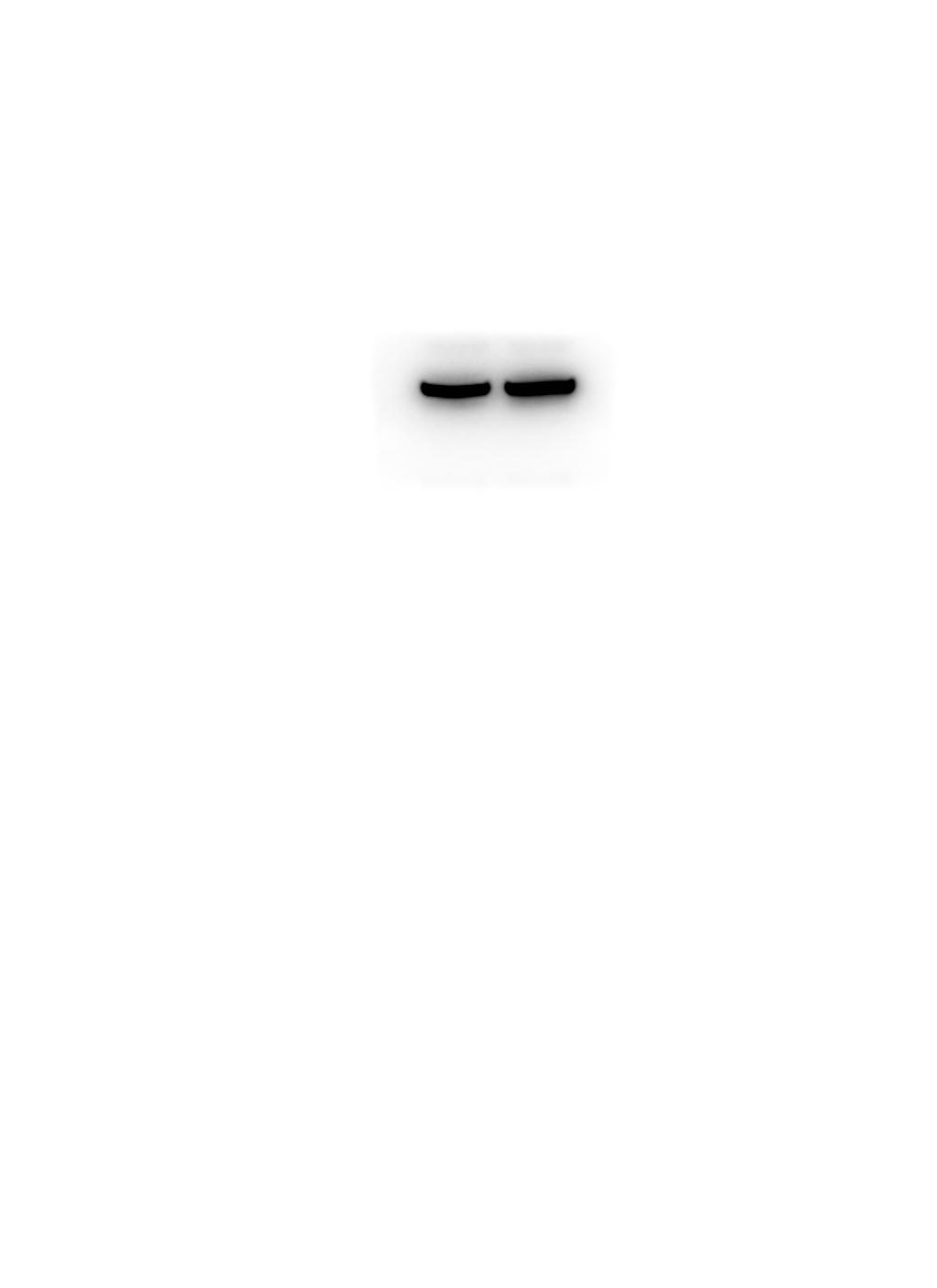

Supplement: Supplementary file 1 [file DataSheet_1.zip › Original data 1/Figure 5G/Input/SMURF1/SMURF1.jpg]

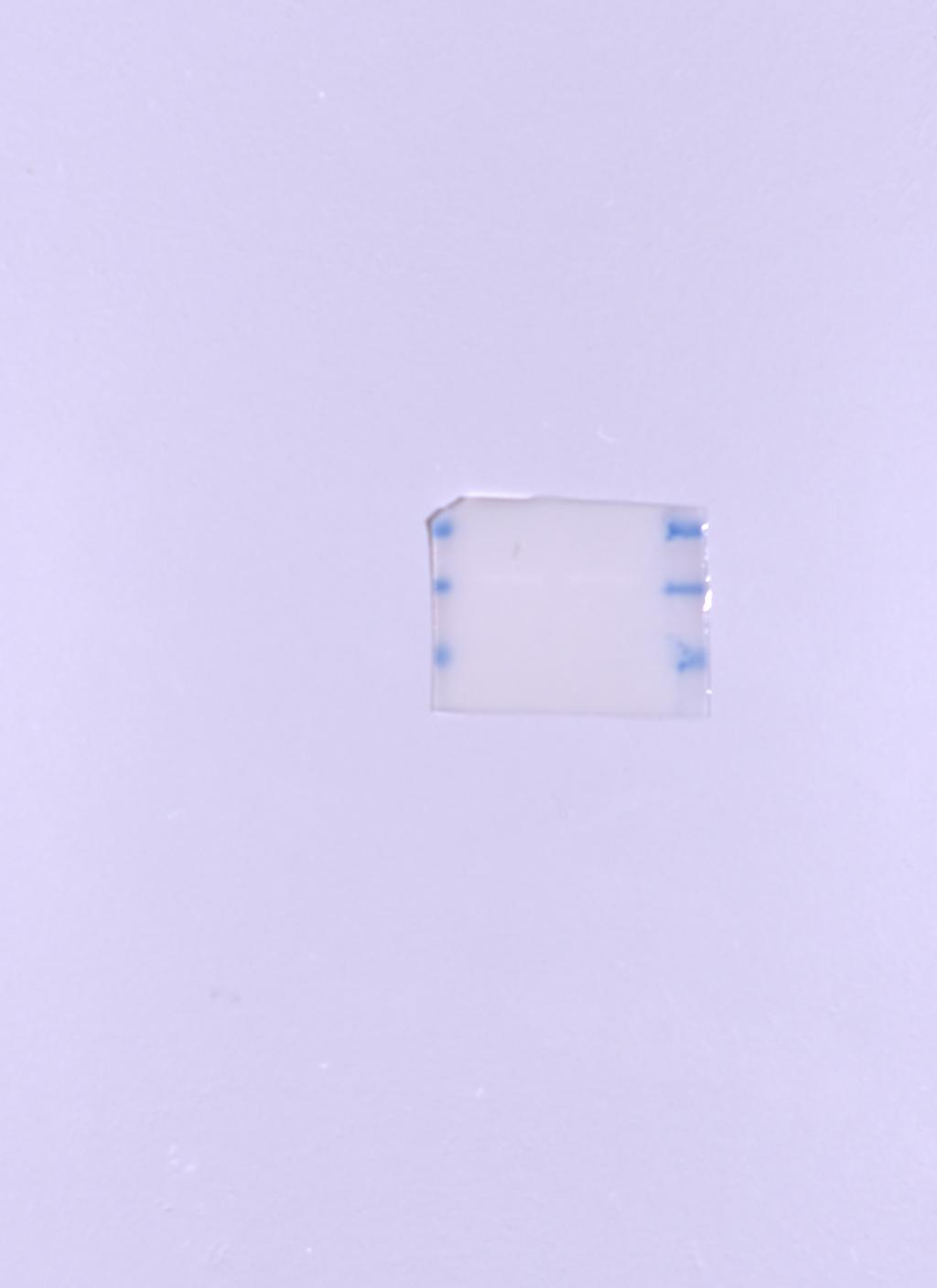

Supplement: Supplementary file 1 [file DataSheet_1.zip › Original data 1/Figure 5G/Input/β-Actin M/β-Actin M.jpg]

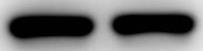

Supplement: Supplementary file 1 [file DataSheet_1.zip › Original data 1/Figure 5G/Input/β-Actin.jpg]

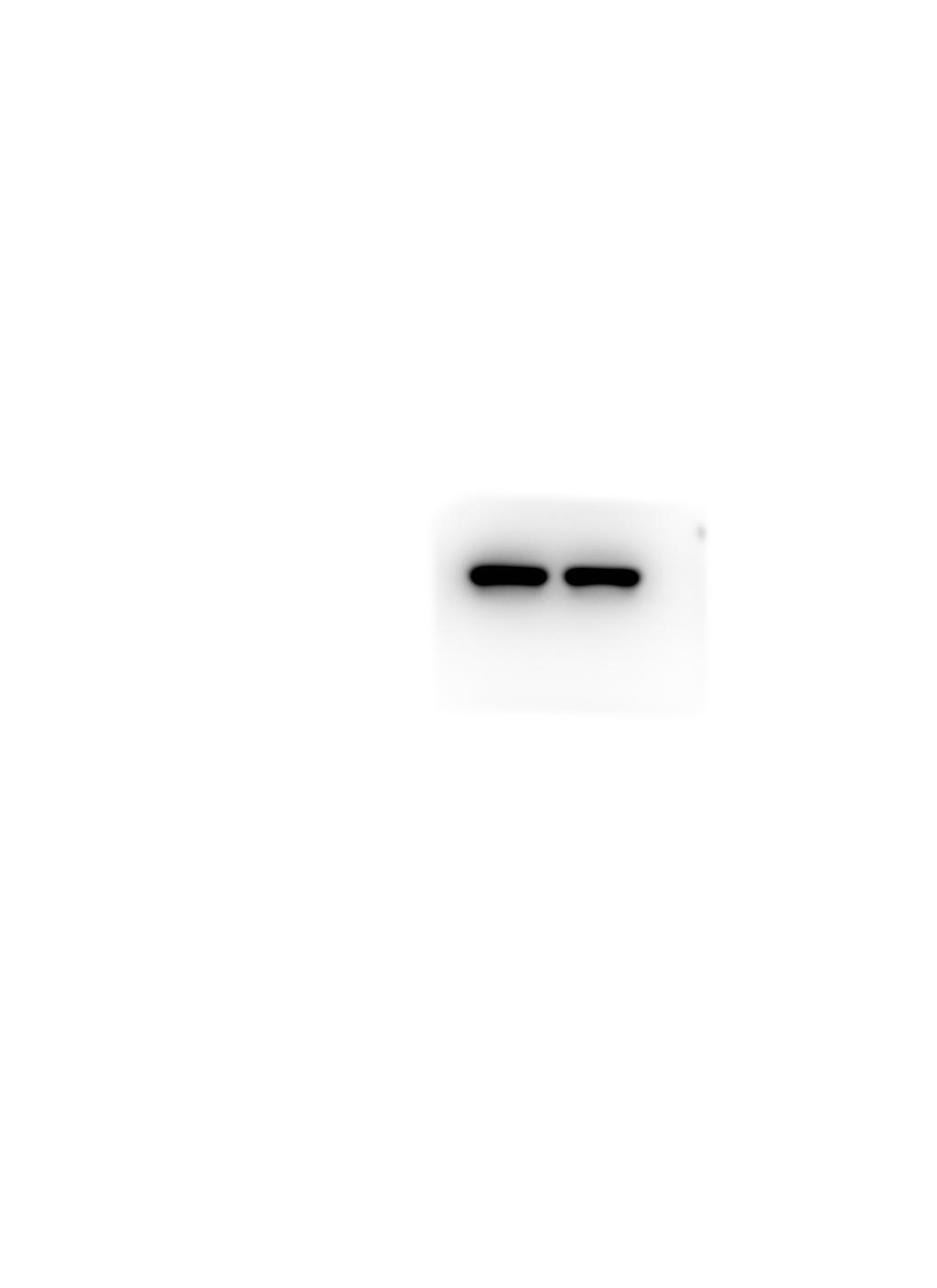

Supplement: Supplementary file 1 [file DataSheet_1.zip › Original data 1/Figure 5G/Input/β-Actin/β-Actin.jpg]

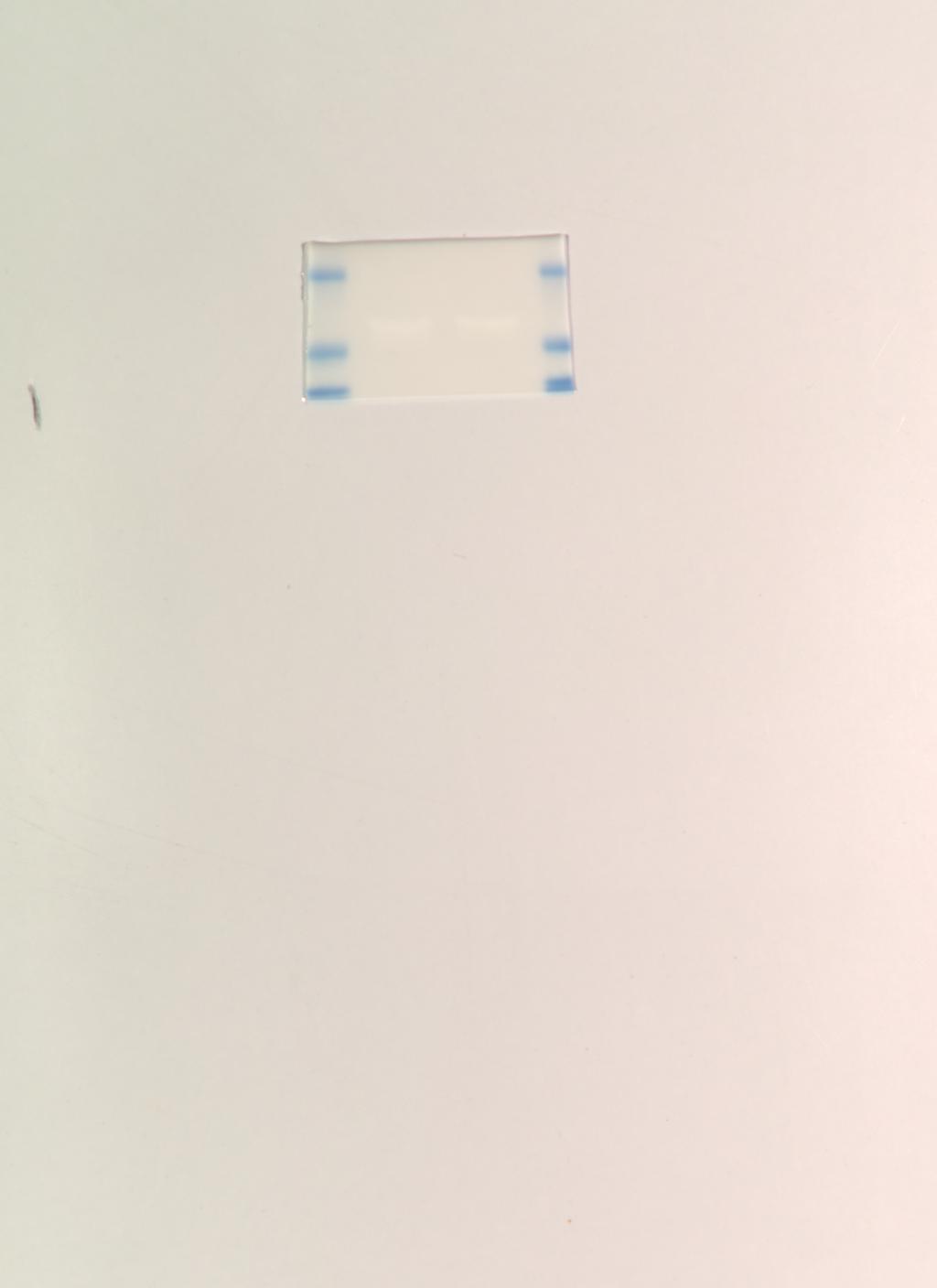

Supplement: Supplementary file 1 [file DataSheet_1.zip › Original data 1/Figure 5G/IP CDK1/CDK1 M/CDK1 M.jpg]

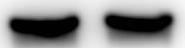

Supplement: Supplementary file 1 [file DataSheet_1.zip › Original data 1/Figure 5G/IP CDK1/CDK1.jpg]

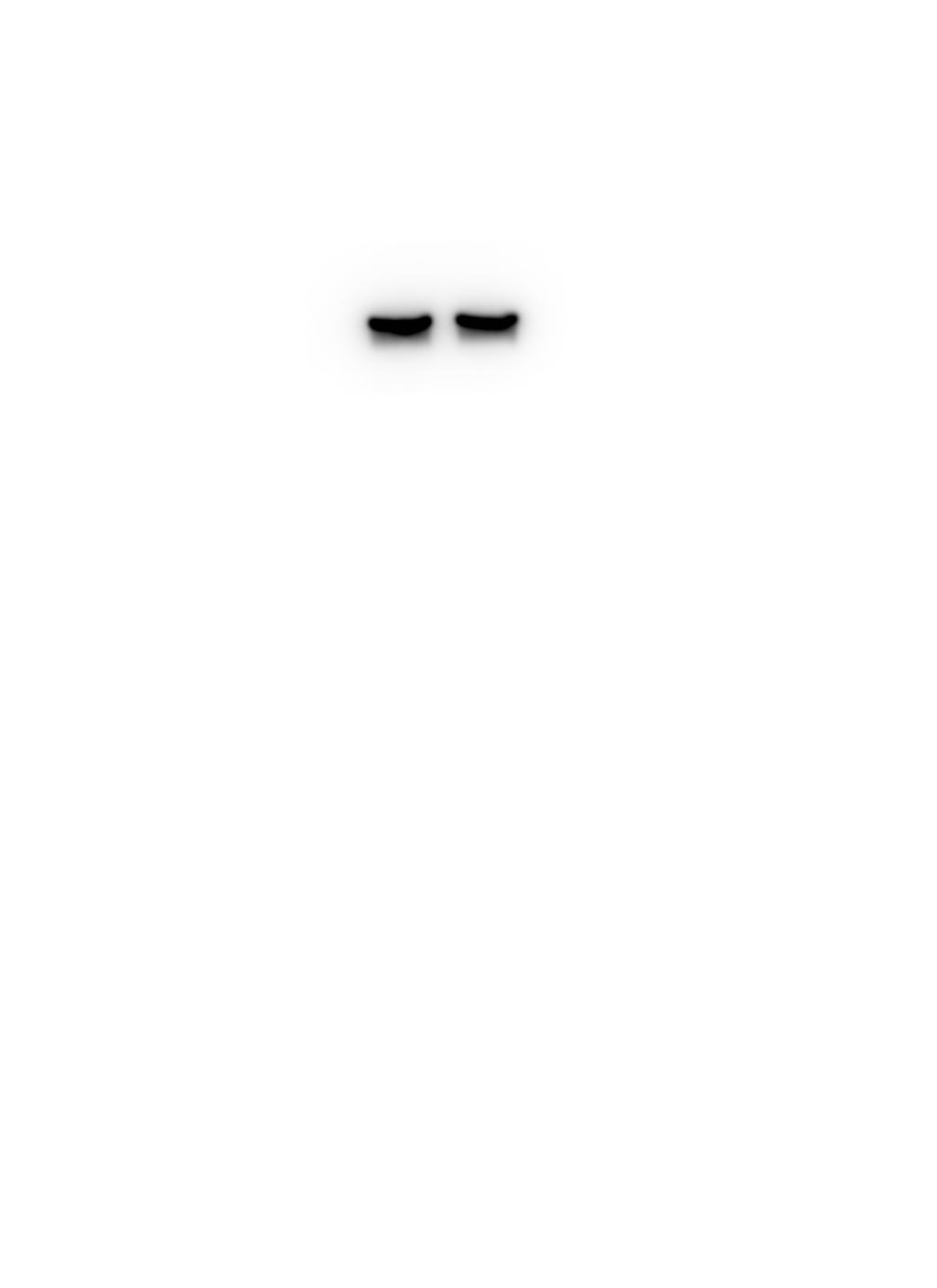

Supplement: Supplementary file 1 [file DataSheet_1.zip › Original data 1/Figure 5G/IP CDK1/CDK1/CDK1.jpg]

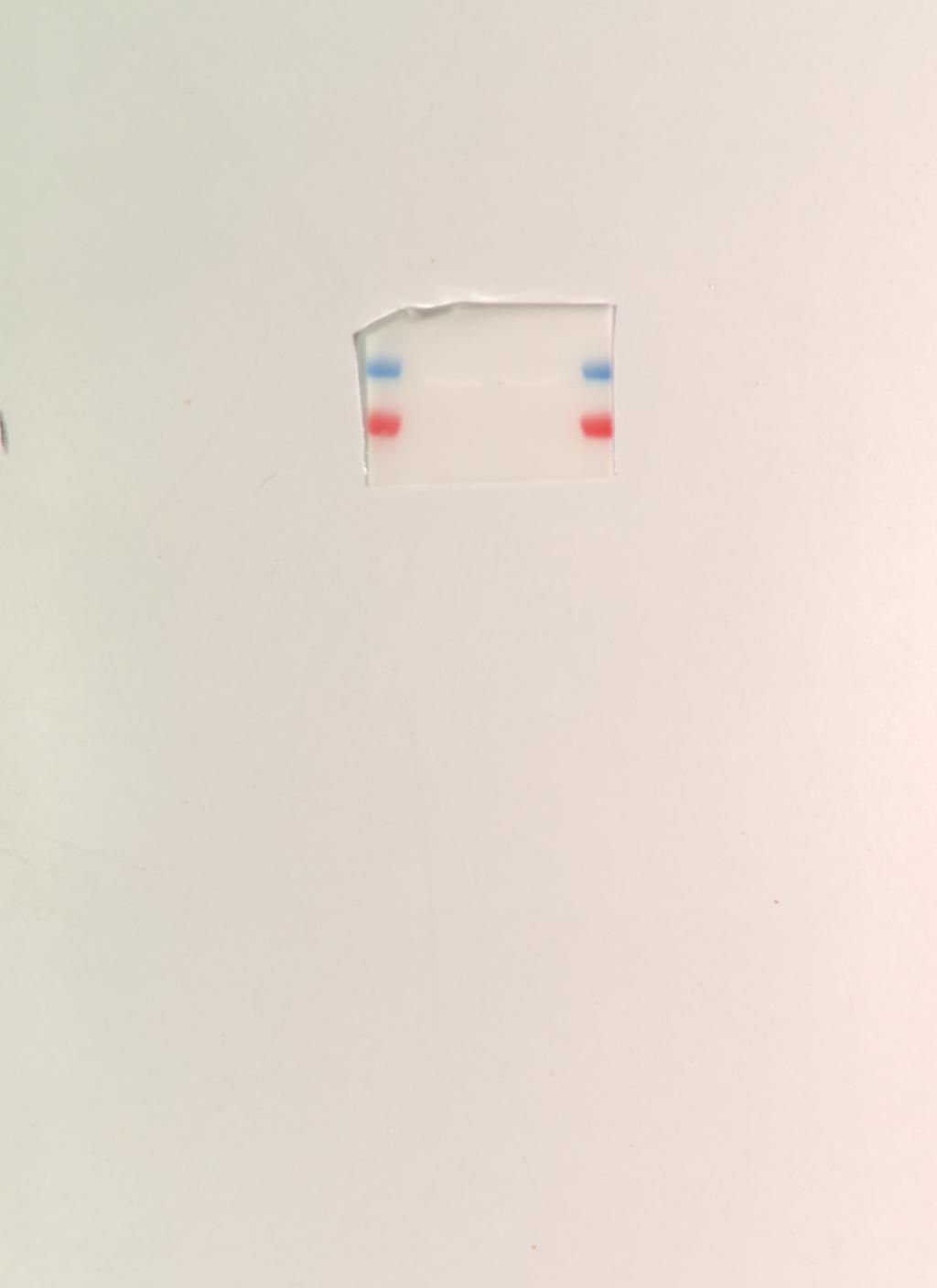

Supplement: Supplementary file 1 [file DataSheet_1.zip › Original data 1/Figure 5G/IP CDK1/SMURF1 M/SMURF1 M.jpg]

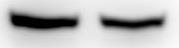

Supplement: Supplementary file 1 [file DataSheet_1.zip › Original data 1/Figure 5G/IP CDK1/SMURF1.jpg]

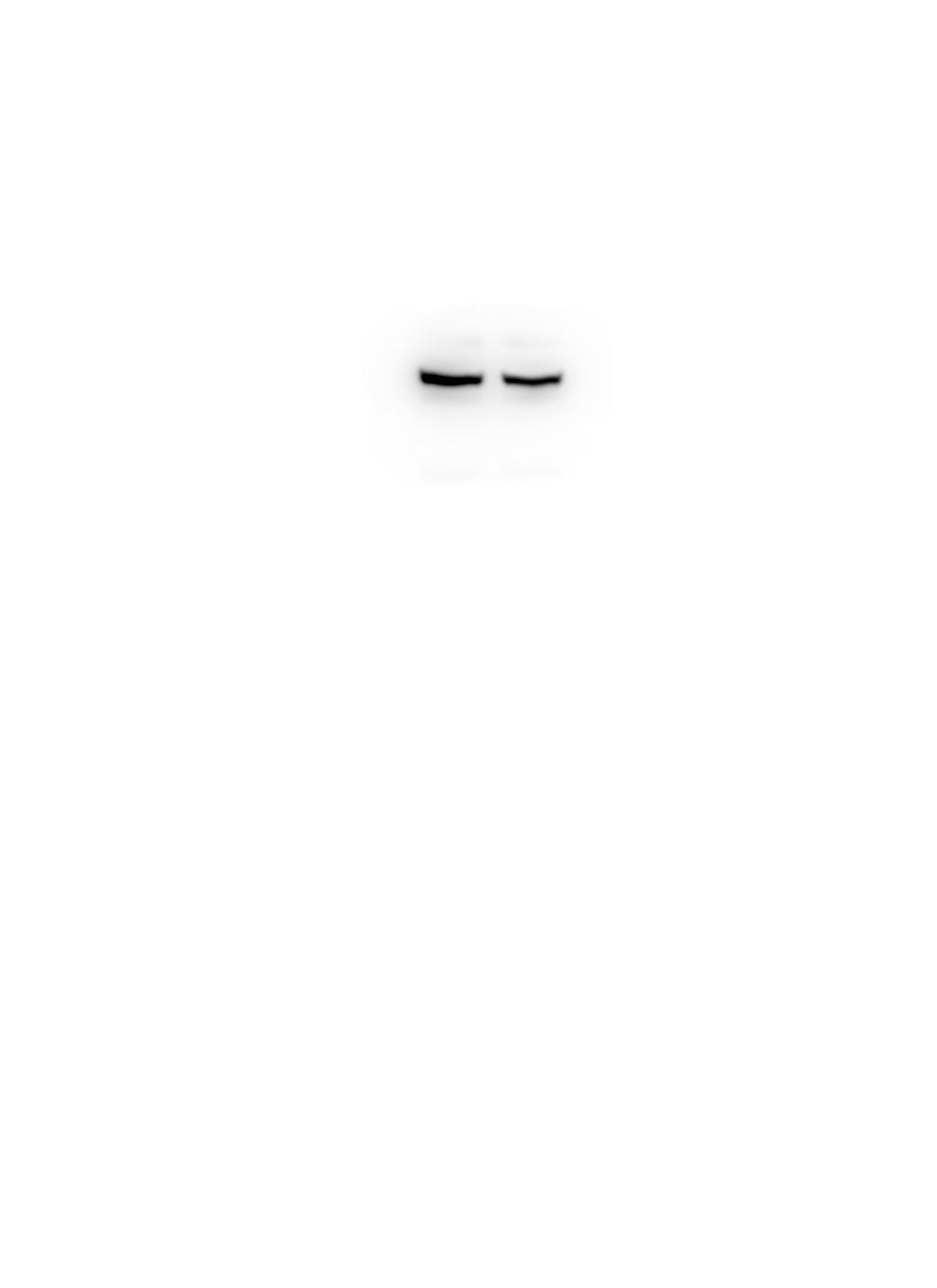

Supplement: Supplementary file 1 [file DataSheet_1.zip › Original data 1/Figure 5G/IP CDK1/SMURF1/SMURF1.jpg]
