# Supplementary material for: DEPDC1B promotes development of cholangiocarcinoma through enhancing the stability of CDK1 and regulating malignant phenotypes
Source: Front Oncol. 2022 Dec 6;12:842205. doi: 10.3389/fonc.2022.842205 (PMC9769124; doi:10.3389/fonc.2022.842205)
Supplement: Supplementary file 4 [file DataSheet_4.pdf]

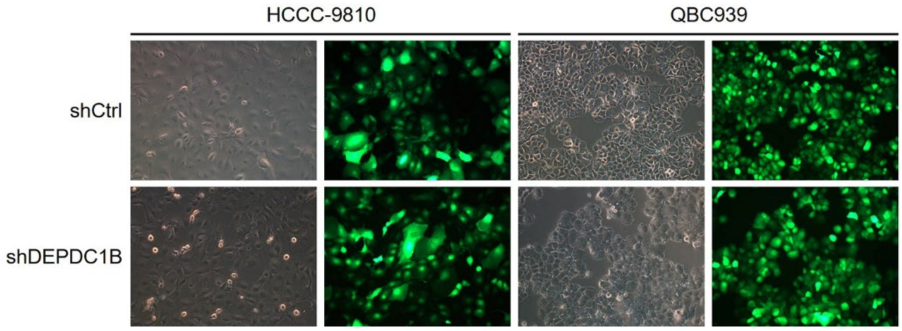

Figure S1. The transfection efficiencies of shDEPDC1B and shCtrl in HCCC-9810 and QBC939 cells were evaluated through observing the fluorescence of GFP on lentivirus vector.

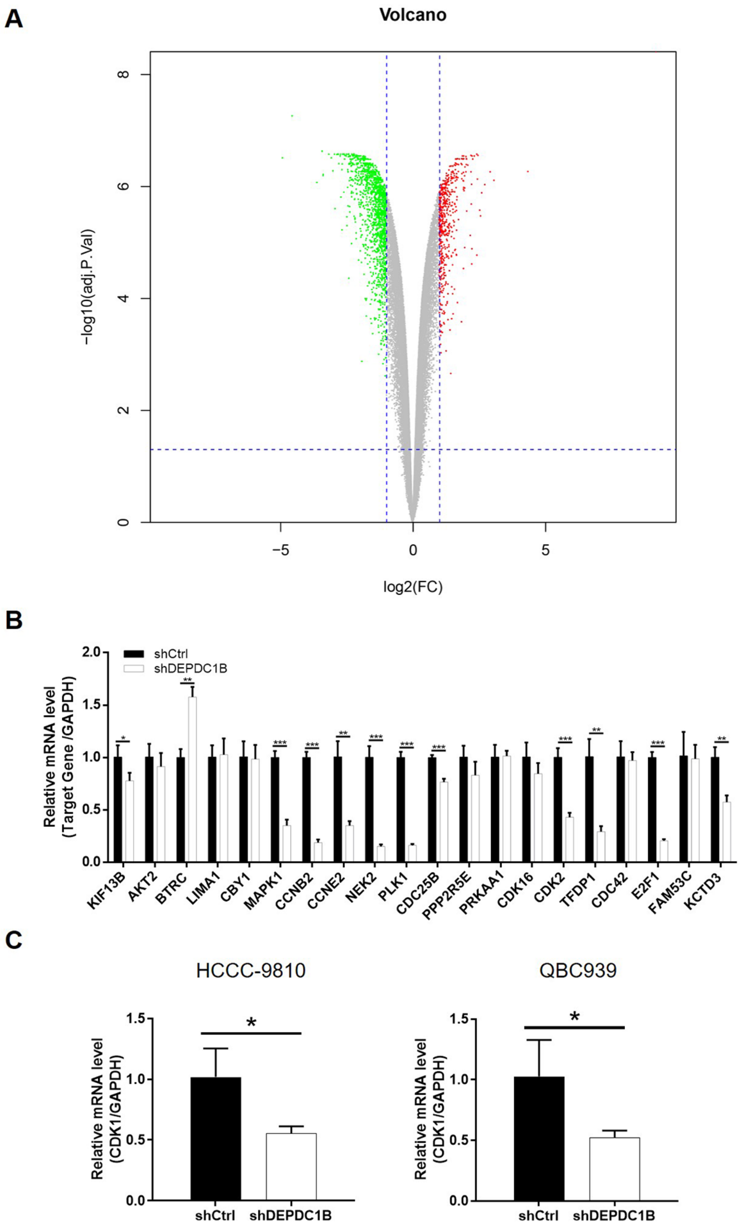

Figure S2. (A) The volcano plot of gene expression profiling in HCCC-9810 cells with or

without DEPDC1B knockdown. Green dots represent the downregulated DEGs, red dots represent the upregulated DEGs. (B) Several DEGs were selected for verification by qPCR. (C) The mRNA expression of CDK1 in HCCC-9810 and QBC939 cells with or without DEPDC1B knockdown was detected by qPCR. Data was shown as mean ± SD (n = 3). *P < 0.05, **P < 0.01, ***P < 0.001

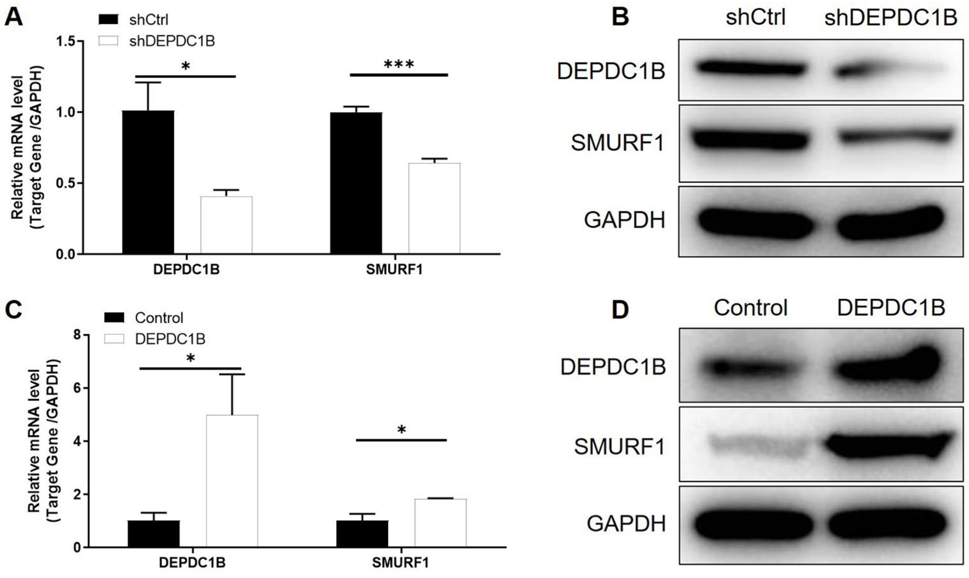

Figure S3. The mRNA (A) and protein (B) levels of DEPDC1B and SMURF1 in HUCCT1 cells with or without DEPDC1B knockdown were detected by qPCR and western blotting, respectively. The mRNA (C) and protein (D) levels of DEPDC1B and SMURF1 in HUCCT1 cells with or without DEPDC1B overexpression were detected by qPCR and western blotting, respectively. Data was shown as mean ± SD (n = 3). *P < 0.05, ***P < 0.001

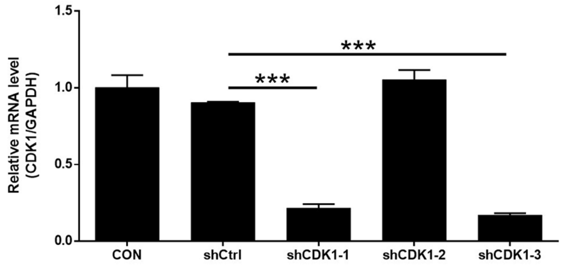

Figure S4. The knockdown efficiencies of 3 shRNAs prepared for silencing CDK1 were evaluated through qPCR. Data was shown as mean ± SD (n = 3). *P < 0.05, **P < 0.01, ***P

< 0.001

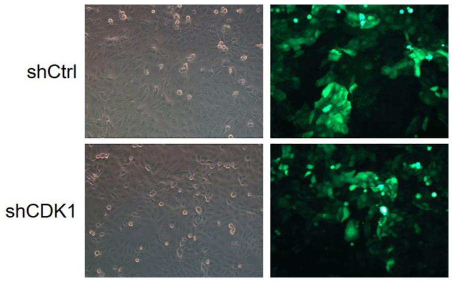

Figure S5. The transfection efficiencies of shCDK1 and shCtrl in HUCCT1 cells were evaluated through observing the fluorescence of GFP on lentivirus vector.

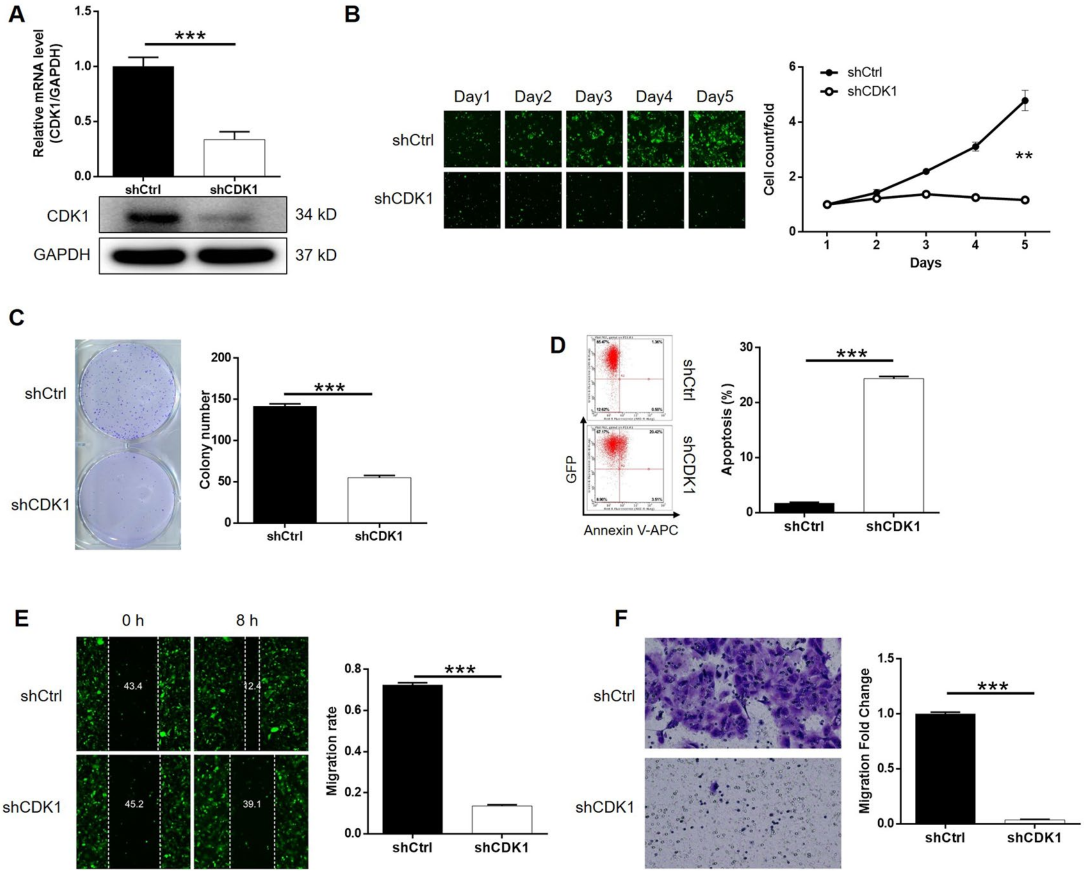

Figure S6. (A) Cell models with or without CDK1 knockdown were constructed by transfecting shCDK1 or shCtrl. The knockdown efficiency of CDK1 in HUCCT1 cells was assessed by qPCR and western blotting, respectively. (B) Celigo cell counting assay was employed to show the effects of CDK1 on cell proliferation of HUCCT1 cells. (C) Colony formation assay was used to evaluate the ability of HUCCT1 cells with or without CDK1

knockdown to form colonies. (D) Flow cytometry was performed to detect cell apoptosis of HUCCT1 cells with or without CDK1 knockdown. (E, F) The effects of CDK1 on cell migration ability of HUCCT1 cells were evaluated by wound-healing assay (E) and Transwell assay (F). The representative images were selected from at least 3 independent experiments. Data was shown as mean ± SD (n = 3). *$P < 0.05$, **$P < 0.01$, ***$P < 0.001$

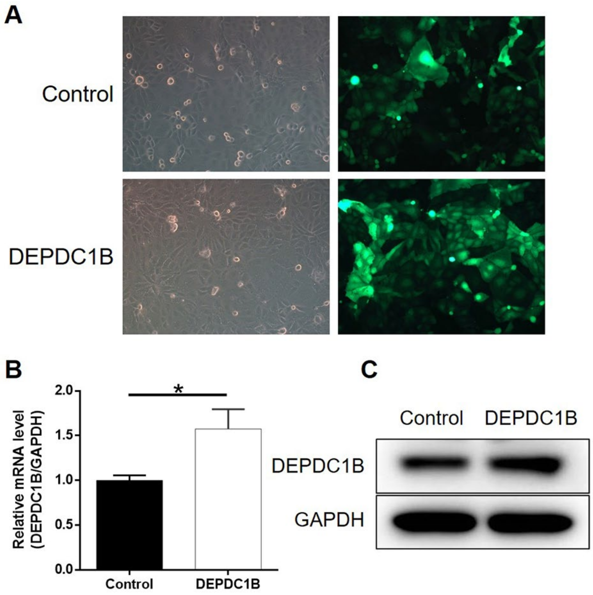

Figure S7. The transfection efficiencies of Control plasmid and DEPDC1B overexpression plasmid in HUCCT1 cells were evaluated through observing the fluorescence of GFP on lentivirus vector. (B, C) The efficiency of DEPDC1B overexpression was detected by qPCR (B) and western blotting (C). Data was shown as mean ± SD (n = 3). *$P < 0.05$, **$P < 0.01$, ***$P < 0.001$

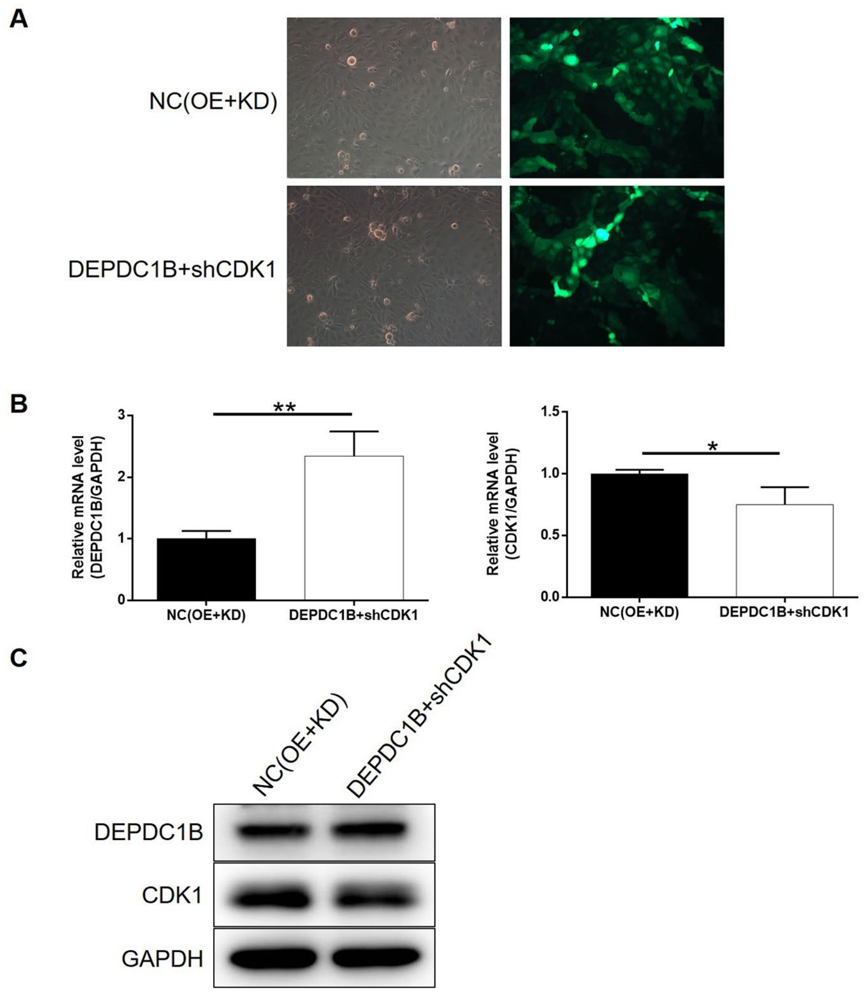

Figure S8. (A) The transfection efficiencies of NC(OE+KD) and DEPDC1B+shCDK1 in HUCCT1 cells were evaluated through observing the fluorescence of GFP on lentivirus vector. (B, C) The mRNA and protein levels of DEPDC1B and CDK1 in HUCCT1 cells transfected with different plasmids were detected by qPCR (B) and western blotting (C), respectively. Data was shown as mean ± SD (n = 3). $^*P < 0.05$, $^{**}P < 0.01$, $^{***}P < 0.001$

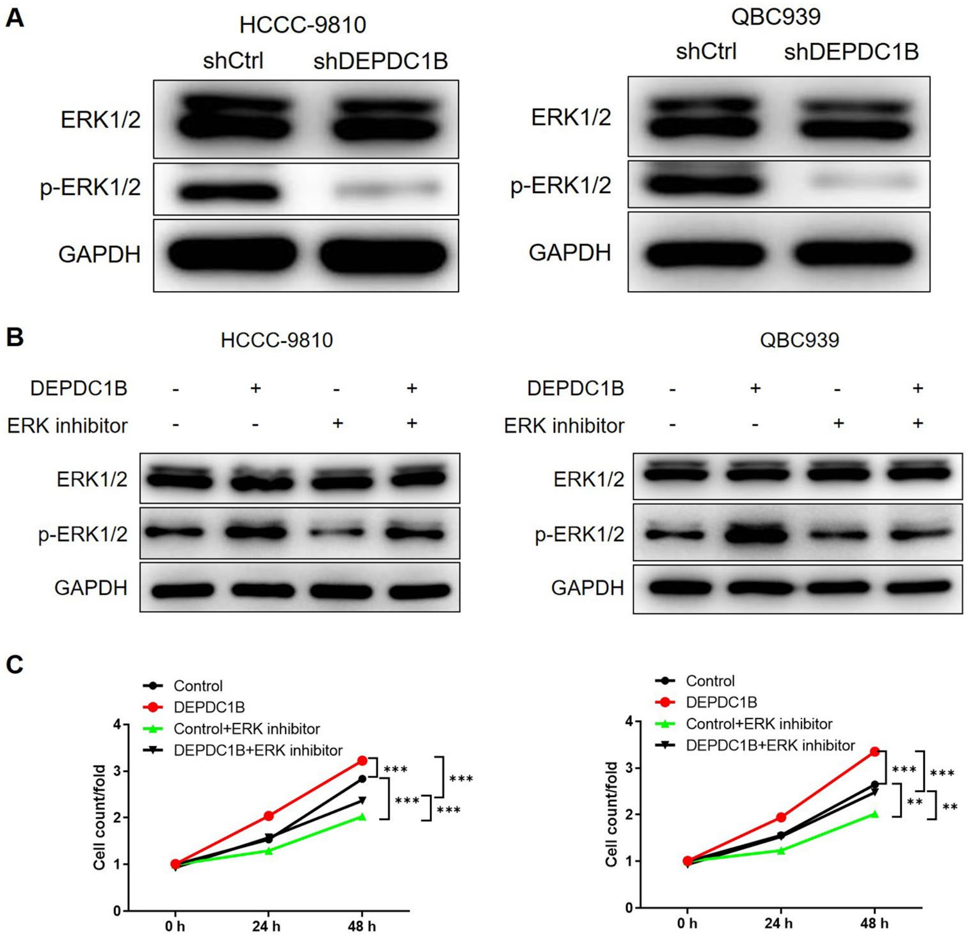

Figure S9. (A) The protein level and phosphorylation level of ERK1/2 were evaluated by western blot in QBC939 and HCCC-9810 cells with or without DEPDC1B knockdown. (B) The protein level and phosphorylation level of ERK1/2 were evaluated by western blot in control and DEPDC1B (overexpression) groups of QBC939 and HCCC-9810 cells with or without treatment of ERK inhibitor (FR 180204, 50 μM). (C) CCK8 assay was performed to assess cell proliferation of control and DEPDC1B (overexpression) groups of QBC939 and HCCC-9810 cells with or without treatment of ERK inhibitor (FR 180204, 50 μM). Data was shown as mean ± SD (n = 3). **$P < 0.01$, ***$P < 0.001$

Table S1 Antibodies used in western blotting and IHC

Primary antibodies	Dilution in WB	Source species	Company	Catalog No.
DEPDC1B	1:1000	Rabbit	abcam	ab124182
GAPDH	1:3000	Rabbit	Bioworld	AP0063
E-cadherin	1:1000	Rabbit	CST	3195
N-cadherin	1:1000	Rabbit	abcam	ab18203
Vimentin	1:1000	Rabbit	abcam	ab92547
SMURF1	1:1000	Rabbit	Abcam	ab38866
CDK1	1:2000	Rabbit	Abcam	ab133327
E2F1	1:1500	Rabbit	Abcam	ab179445
NEK2	1:1000	Rabbit	Abcam	ab115731
ERK	1:3000	Rabbit	CST	4695
p-ERK	1:2000	Rabbit	Proteintech	28733-1-AP
Cyclin E2	1:1000	Rabbit	Abcam	ab40890
DYKDDDDK Tag	1:50/1:1000	Rabbit	CST	14793
**Primary antibodies**	**Dilution in IHC**	**Source species**	**Company**	**Catalog No.**
DEPDC1B	1:100	Rabbit	Bioss	bs-14356R
CDK1	1:100	Rabbit	Abcam	ab133327
Ki67	1:200	Rabbit	Abcam	ab16667

Secondary antibody	Dilution		Company	Catalog No.
HRP Goat Anti-Rabbit IgG (WB)	1:3000		Beyotime	A0208
HRP Goat Anti-Rabbit IgG (IHC)	1:200		Abcam	Ab111909

Table S2 The target sequences and shRNA sequences

Gene	No.	Target sequence (5'-3')	shRNA sequences (5'-3')
DEPDC1B	Pbr-10053-a	gcTGCTAGATTGGTAACGTTT	CcgggcTGCTAGATTGGTAACGTTTctcgagAAACGTTACCAATCTAGCATTTTTg
DEPDC1B	Pbr-10053-b	gcTGCTAGATTGGTAACGTTT	aattcaaaaagcTGCTAGATTGGTAACGTTTctcgagAAACGTTACCAATCTAGCA
DEPDC1B	Pbr-00152-a	GAGGCCAATGTAGAAGAGATA	CcggGAGGCCAATGTAGAAGAGATActcgagTATCTCTTCTACATTGGCCTCTTTTTg
DEPDC1B	Pbr-00152-b	GAGGCCAATGTAGAAGAGATA	aattcaaaaaGAGGCCAATGTAGAAGAGATActcgagTATCTCTTCTACATTGGCCTC
DEPDC1B	Pbr-00153-a	CAGCTATGAAGTGTTTGGCAA	CcggCAGCTATGAAGTGTTTGGCAActcgagTTGCCAAACACTTCATAGCTGTTTTTg
DEPDC1B	Pbr-00153-b	CAGCTATGAAGTGTTTGGCAA	aattcaaaaaCAGCTATGAAGTGTTTGGCAActcgagTTGCCAAACACTTCATAGCTG
CDK1	Pbr-10545-a	TTCCATGGATCTGAAGAAATA	CcggTTCCATGGATCTGAAGAAATActcgagTATTTCTTCAGATCCATGGAATTTTTg
CDK1	Pbr-10545-b	TTCCATGGATCTGAAGAAATA	aattcaaaaaTTCCATGGATCTGAAGAAATActcgagTATTTCTTCAGATCCATGGAA
CDK1	Pbr-10546-a	AGACTAGAAAGTGAAGAGGAA	CcggAGACTAGAAAGTGAAGAGGAActcgagTTCCTCTTCACTTTCTAGTCTTTTTg
CDK1	Pbr-10546-b	AGACTAGAAAGTGAAGAGGAA	aattcaaaaaAGACTAGAAAGTGAAGAGGAActcgagTTCCTCTTCACTTTCTAGTCT
CDK1	Pbr-10547-a	ATGGAGTTGTGTATAAGGGTA	CcggATGGAGTTGTGTATAAGGGTActcgagTACCCTTATACACAACTCCATTTTTg
CDK1	Pbr-10547-b	ATGGAGTTGTGTATAAGGGTA	aattcaaaaaATGGAGTTGTGTATAAGGGTActcgagTACCCTTATACACAACTCCAT

Table S3 Primers used in qPCR

Gene	Forward primer sequence (5’-3’)	Reverse primer sequence (5’-3’)
DEPDC1B	CTGAAGTGACCCGCAAACAAA	CTGGTGGGAGATCATTCCATTC
GAPDH	TGACTTCAACAGCGACACCCA	CACCCTGTTGCTGTAGCCAAA
KIF13B	CTGCGACAAACCTGCCAAAG	TACCCGCTAGAGGCTTCACTCA
AKT2	CCTCATGCTGGACAAAGATGG	GGGGTCCCACAGAAGGTTTT
BTRC	TCTGTCATACCTGGATGCCAAATC	ATGCCATCAGAGGTCACTCG
LIMA1	ACAGCAGCACTGAGATTAGGC	GGTGGATTTGTTCTTCTTGGTC
CBY1	ATACGTTCAGTCCGAAGAAGACAC	TGCCCTGCCAGGTTCATAGT
MAPK1	GACTGGACGTGCTCAGACAT	CCTCCAAACGGCTCAAAGGA
CCNB2	AAGTTCCAGTTCAACCCACCA	GCAGAGCAAGGCATCAGAAAA
CCNE2	AGCTGGTCTGGCGAGGTTTT	GGCCTGGATTATCTGGGCTTC
NEK2	GAAGGAATGCCACAGACGAAGT	CAAGCAGCCCAATGACCAGATA
PLK1	AGAGGAGGAAAGCCCTGACT	TAACTCGGTTTCGGTGCAGG
CDC25B	GACACGCCCGTGCAGAATAA	TGGTCACTGTCCAGGAGGTT
KCTD3	TGGGTGTTCCTGTAGATGCTC	AACCTGCCAGTGCTGAGTGA
PPP2R5E	TCATGGACACGCTATCTGATCT	TGCTCTGTCAAACAGCCTCTGC
PRKAA1	ACAACAGAAATCACCAGGATCC	GGCTTGTCGCCAAATAGAAA
CDK16	CCAGAACCTGCTCATCAACG	CCACAGTGTCACCACCTCATT
CDK2	ATCGCAAATGCTGCACTACG	GGTCACATCCTGGAAGAAAGG
TFDP1	AGAAGGTGCAGAGGAAAGGG	TTGGTAAGATGTGGTTGTCGG
CDC42	GACAGATTACGACCGCTGAGTT	GGAGTCTTTGGACAGTGGTGAG
E2F1	CACTTTCGGCCCTTTTGCTC	GTGCTCTCACCGTCCTACAC
FAM53C	TCTCCAACTGTGGGAACTCTTT	TAGCTGAAGTTGAGGCTGTCC

Table S4 Relationship between DEPDC1B expression and tumor characteristics in patients with CCA analyzed by Spearman rank correlation analysis

Tumor characteristics	index	
Grade	Pearson correlation	0.494
	Significance (two tailed)	<0.001
	n	69
